# Supplementary material for: Comprehensive in silico analyses of fifty-one uncharacterized proteins from Vibrio cholerae
Source: PLoS One. 2024 Oct 4;19(10):e0311301. doi: 10.1371/journal.pone.0311301 (PMC11452002; doi:10.1371/journal.pone.0311301)
Supplement: S1 Table — (DOCX) [file pone.0311301.s001.docx]

**Supplementary Table S1**

**RNA-seq data depicting the expression of genes that were downregulated in *cgtA* knockdown strain of *Vibrio cholerae:*** The p-value and fold change of each protein are recorded and tabulated indicating the alteration in the expression of each protein when *cgtA* is knocked down from *V. cholerae* genome.

| **UniProt ID** | **Gene name** | **Protein Name** | **p-value** | Ψ **Fold Change** |
| --- | --- | --- | --- | --- |
| Q9KRD2 | VC_1710 | EAL domain containing protein | 0.036932969 | 0.15385 |
| Q9KVG3 | VC_0183 | Hypothetical protein | 0.048607587 | 0.15900 |
| Q9KKL8 | VC_A0185 | DUF342 domain containing protein | 0.044541827 | 0.15881 |
| Q9KQX3 | VC_1874 | SpoVR family protein | 9,939512797 | 0.15851 |
| Q9KLK5 | VC_A0738 | Conjugal Transfer protein TraF | 0.048989249 | 0.15904 |
| Q9KT24 | VC_1081 | HDOD domain containing protein | 0.027032121 | 0.15773 |
| Q9KMS2 | VC_A0248 | Uncharacterized protein | 0.041083066 | 0.15862 |
| Q9KMV6 | VC_A0212 | Hypothetical protein | 0.024930907 | 0.15752 |
| Q9KRM9 | VC_1607 | HlyD family secretion system | 0,044233839 | 0.15878 |
| Q9KU75 | VC_0648 | Lipoprotein NlpI | 0.044651939 | 0.15882 |
| Q9KND1 | VC_A0034 | HDOD domain containing protein | 0,016387187 | 0.15718 |
| Q9KTC9 | VC_0973 | Hypothetical protein | 0.049442907 | 0.15911 |
| Q9KS60 | VC­_1400 | Hypothetical protein | 0.015255951 | 0.15710 |
| Q9KKX0 | VC_A0980 | Hypothetical protein | 0.042501822 | 0.15872 |
| Q9KND9 | VC_A0026 | DUF2057 domain containing protein | 0.029802832 | 0.15795 |
| Q9KRJ5 | VC_1645 | HAD domain containing protein | 0.019014478 | 0.15731 |
| Q9KVJ9 | VC_0144 | Uncharacterized protein | 0.025616409 | 0.15760 |
| Q9KSV3 | VC_1153 | Uncharacterized protein | 0.026916239 | 0.15772 |
| Q9KSV6 | VC_1150 | Hypothetical protein | 0.030027089 | 0.15797 |
| Q9KND3 | VC_A0032 | Hypothetical protein | 0.015611256 | 0.15713 |
| Q9KP29 | VC_2550 | YtfJ family protein | 0.048866754 | 0.1590 |
| Q9KMX1 | VC_A0195 | Outer membrane beta-barrel protein | 0.040603845 | 0.15858 |
| Q9KTE5 | VC_0957 | Zinc ribbon containing protein | 0.021055702 | 0.15738 |
| Q9KPD6 | VC_2434 | DUF1249 family protein | 0.028909774 | 0.15787 |
| Q9KPA3 | VC_2470 | Hypothetical proteins | 0.049068537 | 0.15905 |
| Q9KNF4 | VC_A0010 | YibL family ribosome associated protein | 0.018581309 | 0.15725 |
| Q9KT53 | VC_1052 | YbaN family protein | 0.035283002 | 0.0352 |
| Q9KL56 | VC_A0892 | Hypothetical protein | 0.024041417 | 0.15748 |
| Q9KRE6 | VC_1696 | TIGR02647 family protein | 0.028755831 | 0.15785 |
| Q9KLX2 | VC_A0619 | Hypothetical protein | 0.019121829 | 0.15733 |
| Q9KLQ3 | VC_A0689 | Uncharacterized protein | 0.02059642 | 0.15737 |
| Q9KKS6 | VC_A1024 | DUF3081 domain containing protein | 0.047703119 | 0.15897 |
| Q9KPP0 | VC_2326 | YebG family protein | 0.04006664 | 0.15859 |
| Q9KS64 | VC_1396 | Hypothetical protein | 0.013283202 | 0.22565 |
| Q9KN40 | VC_A0125 | DUF3012 domain containing protein | 0.043084743 | 0.15873 |
| Q9KVW5 | VC_0023 | Hypothetical protein | 0.013597271 | 0.22982 |
| Q9KL81 | VC_A0866 | Hypothetical proteins | 0.04781773 | 0.15898 |
| Q9KPA0 | VC_2473 | DUF1107 domain containing protein | 0.033423076 | 0.15816 |
| Q9KL73 | VC_A0874 | Hypothetical protein | 0.028780864 | 0.15786 |
| Q9KNG0 | VC_A0004 | Hypothetical protein | 0.014994607 | 0.22411 |
| Q9KSJ4 | VC_1262 | Hypothetical protein | 0.043414662 | 0.15874 |
| Q9KPZ1 | VC_2221 | DUF3149 domain containing protein | 0.014379399 | 0.23495 |
| Q9KNI6 | VC_2753 | Hypothetical protein | 0.027266299 | 0.15776 |
| Q9KVT0 | VC_0059 | Hypothetical protein | 0.013211102 | 0.21170 |
| Q9KST0 | VC_1176 | Hypothetical protein | 0.028136678 | 0.15780 |

Ψ the cut-off log_2_(fold change) value for downregulated genes is ≤-1.
