# Supplementary material for: Comprehensive in silico analyses of fifty-one uncharacterized proteins from Vibrio cholerae
Source: PLoS One. 2024 Oct 4;19(10):e0311301. doi: 10.1371/journal.pone.0311301 (PMC11452002; doi:10.1371/journal.pone.0311301)
Supplement: S2 Table — (DOCX) [file pone.0311301.s002.docx]

**Table S2**

**Physicochemical properties of the 51 uncharacterized proteins:** Physicochemical properties like molecular weight, isoelectric point, aliphatic index(thermostability), GRAVY (solubility), molar extinction coefficient, instability index (Stability in solution) and number of positive and negative residues were assessed.

| **UniProt ID** | **Gene name** | **M.W**  **(KDa)** | **pI** | **Length** | **‡ Aliphatic index** | **$ GRAVY** | **Molar Extinction Coefficient**  **M^-1^ cm^-1^ (280 nm)** | | **† Instability index** | **No. of Positive residues** | **No. of Negative residues** |
| --- | --- | --- | --- | --- | --- | --- | --- | --- | --- | --- | --- |
|  |  |  |  |  |  |  | **Cystines** | **Reduced Cysteine** |  |  |  |
| **Q9KRD2** | **VC_1710** | 91.831 | 6.02 | 818 | 97.97 | -0.172 | 89645 | 89270 | 37.10  (stable) | 78 | 92 |
| **Q9KVG3** | **VC_0183** | 81.883 | 8.51 | 703 | 79.35 | -0.534 | 116170 | 115170 | 40.14  (unstable) | 97 | 89 |
| **Q9KT38** | **VC_1067** | 79.308 | 6.81 | 693 | 99.19 | -0.169 | 88380 | 88130 | 39.47  (stable) | 77 | 79 |
| **Q9KKL8** | **VC_A0185** | 60.476 | 7.65 | 556 | 94.87 | -0.233 | 17670 | 17420 | 31  (Stable) | 72 | 71 |
| **Q9KQX3** | **VC_1874** | 60.882 | 5.96 | 516 | 78.28 | -0.577 | 98585 | 98210 | 49.21  (unstable) | 64 | 74 |
| **Q9KLK5** | **VC_A0738** | 44.672 | 4.45 | 413 | 89.52 | -0.109 | 47330 | 47330 | 17.60  (stable) | 32 | 50 |
| **Q9KT24** | **VC_1081** | 42.950 | 5.11 | 380 | 103.95 | -0.030 | 35910 | 35410 | 40.31  (unstable) | 37 | 54 |
| **Q9KMS2** | **VC_A0248** | 40.466 | 5.45 | 355 | 79.32 | -0.339 | 85745 | 85370 | 37.67  (stable) | 33 | 45 |
| **Q9KMV6** | **VC_A0212** | 39.620 | 5.89 | 349 | 97.25 | -0.198 | 86860 (No cysteines present) | | 35.78  (stable) | 34 | 40 |
| **Q9KRM9** | **VC_1607** | 35.772 | 5.24 | 324 | 82.87 | -0.280 | 47900 (No cysteines present) | | 36.37  (stable) | 33 | 40 |
| **Q9KU75** | **VC_0648** | 35.150 | 4.79 | 303 | 93.80 | -0.315 | 57300 | 57300 | 44.16  (unstable) | 28 | 43 |
| **Q9KND1** | **VC_A0034** | 32.410 | 5.93 | 290 | 10.62 | -0.077 | 26470 (No cysteines present) | | 30.99  (stable) | 30 | 35 |
| **Q9KTC9** | **VC_0973** | 30.380 | 5.01 | 271 | 79.15 | -0.203 | 40005 | 39880 | 28.71  (Stable) | 24 | 31 |
| **Q9KSQ9** | **VC_1197** | 25.832 | 4.63 | 231 | 89.44 | -.0.268 | 6085 | 5960 | 43.59  (unstable) | 19 | 33 |
| **Q9KS60** | **VC­_1400** | 24.616 | 4.61 | 218 | 114.59 | -0.108 | 8605 | 8480 | 58.78  (unstable) | 14 | 33 |
| **Q9KKX0** | **VC_A0980** | 23.868 | 6.16 | 214 | 116.17 | 0.224 | 18575 | 18450 | 21.78  (stable) | 22 | 23 |
| **Q9KND9** | **VC_A0026** | 23.633 | 5.36 | 211 | 78.29 | -0.299 | 18575 | 18450 | 33.25  (stable) | 24 | 27 |
| **Q9KRJ5** | **VC_1645** | 23.579 | 5.96 | 209 | 87.75 | -0.146 | 25440 | 25440 | 32.18  (stable) | 23 | 19 |
| **Q9KVJ9** | **VC_0144** | 23.315 | 8.86 | 209 | 135.36 | 1.019 | 51450 | 51450 | 26.72  (stable) | 9 | 7 |
| **Q9KSV3** | **VC_1153** | 23.579 | 6.53 | 202 | 80.10 | -0.506 | 23045 | 22920 | 51.64  (unstable) | 28 | 29 |
| **Q9KSV6** | **VC_1150** | 22.815 | 8.97 | 201 | 83.53 | -0.493 | 30035 | 29910 | 38.64  (stable) | 25 | 21 |
| **Q9KND3** | **VC_A0032** | 20.388 | 4.19 | 186 | 91.77 | -0.259 | 23950 (No cysteines present) | | 36.50  (Stable) | 11 | 26 |
| **Q9KP29** | **VC_2550** | 19.815 | 5.69 | 183 | 100.27 | 0.017 | 24980 | 24980 | 47.16  (unstable) | 15 | 17 |
| **Q9KMX1** | **VC_A0195** | 20.331 | 5.11 | 182 | 71.26 | -0.086 | 30495 | 30370 | 29.95  (stable) | 13 | 15 |
| **Q9KTE5** | **VC_0957** | 17.414 | 4.71 | 156 | 83.78 | -0.340 | 18700 | 18450 | 42.57  (unstable) | 14 | 25 |
| **Q9KPD6** | **VC_2434** | 17.017 | 5.58 | 148 | 88.85 | -0.311 | 19160 | 18910 | 43.77  (Unstable) | 14 | 17 |
| **Q9KPA3** | **VC_2470** | 17.336 | 9.62 | 148 | 114.66 | 0.375 | 50085 | 49960 | 50.70  (unstable) | 14 | 9 |
| **Q9KNF4** | **VC_A0010** | 16.370 | 9.84 | 145 | 82.07 | -0.477 | 3105 | 2980 | 38.86  (stable) | 28 | 17 |
| **Q9KT53** | **VC_1052** | 15.312 | 11.33 | 132 | 121.14 | 0.662 | 1490 (No cysteines present) | | 51.77  (unstable) | 11 | 2 |
| **Q9KL56** | **VC_A0892** | 14.658 | 9.41 | 132 | 90.08 | 0.022 | 14105 | 13980 | 30.32  (stable) | 10 | 7 |
| **Q9KRE6** | **VC_1696** | 14.059 | 8.31 | 126 | 109.92 | 0.006 | 3230 | 2980 | 36.27  (stable) | 15 | 13 |
| **Q9KLX2** | **VC_A0619** | 14.150 | 5.02 | 122 | 92.62 | -0.102 | 19605 | 19480 | 46.09  (unstable) | 9 | 15 |
| **Q9KLQ3** | **VC_A0689** | 13.135 | 5.50 | 115 | 82.26 | -0.470 | 4470 (No cysteines present) | | 37.81  (stable) | 15 | 16 |
| **Q9KKS6** | **VC_A1024** | 13.051 | 6.19 | 113 | 70.88 | -0.511 | 17420 | 17420 | 30.01 (Stable) | 14 | 16 |
| **Q9KN87** | **VC_A0078** | 12.886 | 5.18 | 110 | 64.73 | -0.475 | 15595 | 15470 | 30.24  (stable) | 15 | 18 |
| **Q9KU58** | **VC_0666** | 12.084 | 11.40 | 104 | 105.87 | -0.319 | 13980 (No cysteines present) | | 73.99  (Unstable) | 19 | 10 |
| **Q9KPP0** | **VC_2326** | 11.128 | 4.41 | 102 | 78.63 | -0.436 | 2980 | 2980 | 43.88  (unstable) | 14 | 24 |
| **B1B1N2** | **VC_A0594** | 10.582 | 4.75 | 93 | 65.91 | -0.642 | 13075 | 12950 | 28.44  (Stable) | 8 | 13 |
| **Q9K2J6** | **VC_A0319** | 9.683 | 6.21 | 90 | 110.78 | 0.040 | 1490 (No cysteines present) | | 29.64  (stable) | 11 | 11 |
| **Q9KS64** | **VC_1396** | 9.5950 | 6.04 | 86 | 97.33 | -.0158 | 13980 | 13980 | 29.82  (stable) | 8 | 10 |
| **Q9KN40** | **VC_A0125** | 9.7831 | 5.06 | 86 | 53.37 | -0.595 | 25230 | 24980 | 20.93  (stable) | 11 | 14 |
| **Q9KVW5** | **VC_0023** | 9.732 | 6.04 | 85 | 118.12 | 0.355 | 13980 (No cysteines present) | | 57.17  (unstable) | 7 | 8 |
| **Q9KL81** | **VC_A0866** | 9.214 | 10.01 | 79 | 101.01 | -0.315 | 2980 | 2980 | 43.28  (unstable) | 14 | 6 |
| **Q9KPA0** | **VC_2473** | 9.223 | 10.12 | 77 | 65.84 | -0.783 | 5960 | 5960 | 36.75 (Stable) | 18 | 8 |
| **Q9KL73** | **VC_A0874** | 7.869 | 9.27 | 67 | 55.37 | -0.967 | 4470 | 4470 | 42.81  (stable) | 14 | 10 |
| **Q9KNG0** | **VC_A0004** | 7.533 | 5.09 | 65 | 90.15 | -0.871 | 5500 (No cysteines present) | | 101.35  (Unstable) | 10 | 14 |
| **Q9KSJ4** | **VC_1262** | 6.687 | 5.64 | 58 | 94.14 | -0.502 | 12490 (No cysteines present) | | 58.86  (unstable) | 6 | 7 |
| **Q9KPZ1** | **VC_2221** | 5.130 | 5.28 | 46 | 116.52 | 1.002 | 8480 (No cysteines present) | | 24.98  (Stable) | 2 | 3 |
| **Q9KNI6** | **VC_2753** | 5.1038 | 5.14 | 46 | 89.13 | -0.226 | 1490 (No cysteines present) | | 50.43  (unstable) | 6 | 7 |
| **Q9KVT0** | **VC_0059** | 5.115 | 6.13 | 46 | 97.61 | -0.150 | 1490 | 1490 | 44.29  (unstable) | 7 | 7 |
| **Q9KST0** | **VC_1176** | 5.4111 | 5.30 | 45 | 58.67 | -0.444 | 28990 (No cysteines present) | | 30.65  (stable) | 2 | 4 |

**†** Instability index < 40 and > 40 is predicted the protein as stable and unstable, respectively.

**‡** Aliphatic index (alanine, valine, isoleucine, and leucine) is regarded as a positive factor for the increase of thermostability of globular proteins.

**$** GRAVY is calculated as the sum of hydropathy values of all the amino acids, divided by the number of residues in the sequence. Negative and positive GRAVY values indicate that the protein is non-polar and polar, respectively.
