# Supplementary material for: Comprehensive in silico analyses of fifty-one uncharacterized proteins from Vibrio cholerae
Source: PLoS One. 2024 Oct 4;19(10):e0311301. doi: 10.1371/journal.pone.0311301 (PMC11452002; doi:10.1371/journal.pone.0311301)
Supplement: S3 Table — (DOCX) [file pone.0311301.s003.docx]

**Table S3**

**Amino acid composition (%) of the uncharacterized and hypothetical proteins**

| UniProt ID | **C** | **S** | **T** | **P** | **A** | **G** | **N** | **D** | **E** | **Q** | **H** | **R** | **K** | **M** | **I** | **L** | **V** | **F** | **Y** | **W** |
| --- | --- | --- | --- | --- | --- | --- | --- | --- | --- | --- | --- | --- | --- | --- | --- | --- | --- | --- | --- | --- |
| Q9KRD2 | 0.9 | 6.6 | 6.0 | 3.8 | 7.5 | 5.9 | 3.1 | 5.6 | 5.6 | 7.6 | 3.2 | 4.9 | 4.6 | 1.3 | 4.9 | 12.6 | 7.7 | 4.3 | 2.8 | 1.2 |
| Q9KVG3 | 2.4 | 7.3 | 4.4 | 2.7 | 4.8 | 5.4 | 4.4 | 4.8 | 7.8 | 5.1 | 3.6 | 5.3 | 8.5 | 2.6 | 8.0 | 8.0 | 4.3 | 4.3 | 4.7 | 1.7 |
| Q9KT38 | 0.7 | 6.6 | 4.9 | 2.9 | 7.6 | 4.6 | 4.5 | 4.9 | 6.5 | 5.3 | 2.5 | 5.9 | 5.2 | 2.0 | 6.2 | 11.7 | 7.5 | 4.2 | 5.3 | 0.9 |
| Q9KKL8 | 0.9 | 4.9 | 5.2 | 3.2 | 8.8 | 9.2 | 4.3 | 4.1 | 8.6 | 4.7 | 2.0 | 4.7 | 8.3 | 2.5 | 6.8 | 8.8 | 8.6 | 2.7 | 1.4 | 0.2 |
| Q9KQX3 | 1.4 | 5.6 | 3.9 | 4.7 | 6.6 | 3.5 | 4.7 | 5.2 | 9.1 | 5.2 | 3.3 | 5.8 | 6.6 | 2.5 | 6.8 | 8.1 | 4,7 | 4.8 | 5.6 | 1.9 |
| Q9KLK5 | 0.2 | 7.5 | 5.3 | 1.7 | 10.7 | 8.5 | 6.8 | 9.0 | 3.1 | 5.1 | 0.2 | 3.4 | 4.4 | 1.9 | 3.6 | 9.9 | 9.0 | 4.6 | 4.1 | 1.0 |
| Q9KT24 | 2.4 | 6.1 | 3.4 | 4.5 | 8.4 | 4.2 | 3.7 | 6.3 | 7.9 | 4.2 | 2.9 | 6.1 | 3.7 | 2.6 | 7.6 | 11.6 | 7.1 | 3.9 | 2.4 | 1.1 |
| Q9KMS2 | 1.7 | 5.6 | 5.1 | 4.2 | 5.9 | 7.3 | 5.1 | 7.0 | 5.6 | 4.5 | 3.4 | 3.9 | 5.4 | 2.8 | 5.4 | 7.6 | 7.9 | 4.5 | 3.7 | 3.4 |
| Q9KMV6 | 0.0 | 4.9 | 2.6 | 4.6 | 8.0 | 8.0 | 4.0 | 4.6 | 6.9 | 6.3 | 2.6 | 4.0 | 5.7 | 2.3 | 6.9 | 10.9 | 6.9 | 3.4 | 4.0 | 3.4 |
| Q9KRM9 | 0.0 | 4.0 | 7.4 | 2.8 | 13.9 | 6.2 | 1.9 | 4.3 | 8.0 | 9.6 | 0.9 | 4.0 | 6.2 | 2.5 | 3.1 | 7.7 | 9.3 | 3.4 | 3.1 | 1.9 |
| Q9KU75 | 0.3 | 5.0 | 3.6 | 4.0 | 10.6 | 4.3 | 2.6 | 4.6 | 9.6 | 8.3 | 1.3 | 6.6 | 2.6 | 2.0 | 5.0 | 13.2 | 4.3 | 4.0 | 6.6 | 1.7 |
| Q9KND1 | 0.0 | 7.6 | 6.2 | 3.8 | 9.3 | 4.1 | 4.5 | 5.5 | 6.6 | 4.1 | 2.8 | 4.8 | 5.5 | 4.5 | 8.6 | 10.7 | 5.5 | 3.4 | 1.0 | 1.4 |
| Q9KTC9 | 1.1 | 8.1 | 6.6 | 4.1 | 10.3 | 2.2 | 6.3 | 4.8 | 6.6 | 5.9 | 0.7 | 1.5 | 7.4 | 3.0 | 4.4 | 5.5 | 10.3 | 5.2 | 4.4 | 1.5 |
| Q9KSQ9 | 0.9 | 6.9 | 8.2 | 2.6 | 6.1 | 3.9 | 6.9 | 6.9 | 7.4 | 6.5 | 1.7 | 2.2 | 6.1 | 4.3 | 4.3 | 11.3 | 7.8 | 4.3 | 1.7 | 0.0 |
| Q9KS60 | 0.9 | 5.5 | 4.1 | 5.0 | 9.2 | 1.8 | 4.6 | 7.8 | 7.3 | 8.7 | 4.1 | 5.0 | 1.4 | 1.4 | 10.1 | 12.8 | 5.5 | 3.2 | 0.9 | 0.5 |
| Q9KKX0 | 1.4 | 7.0 | 8.4 | 3.7 | 7.0 | 3.7 | 4.7 | 4.2 | 6.5 | 2.8 | 0.9 | 3.3 | 7.0 | 1.4 | 7.9 | 14.5 | 7.5 | 4.7 | 2.3 | 0.9 |
| Q9KND9 | 0.9 | 5.2 | 5.2 | 3.3 | 12.8 | 4.7 | 3.8 | 5.7 | 7.1 | 8.1 | 0.5 | 4.3 | 7.1 | 4.3 | 5.7 | 7.6 | 4.7 | 5.7 | 2.4 | 0.9 |
| Q9KRJ5 | 0.5 | 3.8 | 3.8 | 3.3 | 9.6 | 7.2 | 3.3 | 5.7 | 5.3 | 8.6 | 3.3 | 3.3 | 5.7 | 3.3 | 3.8 | 10.5 | 7.7 | 6.7 | 2.9 | 1.4 |
| Q9KVJ9 | 0.5 | 10.5 | 3.8 | 2.9 | 10.5 | 4.8 | 1.0 | 1.0 | 2.4 | 4.3 | 3.3 | 2.4 | 1.9 | 3.3 | 9.1 | 17.2 | 7.7 | 7.2 | 2.4 | 3.8 |
| Q9KSV3 | 1.0 | 5.4 | 3.5 | 2.5 | 5.4 | 6.4 | 2.5 | 5.9 | 8.4 | 7.4 | 2.5 | 7.4 | 6.4 | 4.0 | 5.0 | 9.4 | 6.4 | 5.4 | 4.0 | 1.0 |
| Q9KSV6 | 1.0 | 8.5 | 6.0 | 3.5 | 10.0 | 3.0 | 5.5 | 4.5 | 6.0 | 5.5 | 35 | 5.0 | 7.5 | 2.0 | 3.0 | 11.4 | 6.0 | 2.5 | 4.5 | 1.5 |
| Q9KND3 | 0.0 | 8.6 | 5.9 | 2.7 | 9.7 | 4.8 | 5.9 | 7.0 | 7.0 | 9.7 | 0.5 | 05 | 5.4 | 2.7 | 7.5 | 7.5 | 8.1 | 2.2 | 2.7 | 1.6 |
| Q9KP29 | 0.5 | 8.7 | 4.9 | 3.3 | 12.0 | 5.5 | 5.5 | 4.4 | 4.9 | 7.7 | 1.1 | 1.6 | 6.6 | 2.2 | 7.1 | 9.8 | 7.7 | 3.3 | 1.1 | 2.2 |
| Q9KMX1 | 1.6 | 11.0 | 4.4 | 3.8 | 4.9 | 11.0 | 4.4 | 3.8 | 4.4 | 5.5 | 0.0 | 3.3 | 3.8 | 2.7 | 5.5 | 8.2 | 4.4 | 8.8 | 7.1 | 1.1 |
| Q9KTE5 | 2.6 | 8.3 | 4.5 | 4.5 | 7.1 | 5.8 | 2.6 | 6.4 | 9.6 | 5.8 | 2.6 | 1.3 | 7.7 | 1.9 | 5.1 | 10.3 | 5.8 | 3.8 | 3.2 | 1.3 |
| Q9KPD6 | 2.7 | 6.1 | 6.1 | 6.1 | 6.1 | 2.7 | 3.4 | 4.7 | 6.8 | 8.1 | 2.0 | 5.4 | 4.1 | 3.4 | 1.4 | 12.8 | 9.5 | 2.0 | 6.1 | 0.7 |
| Q9KPA3 | 1.4 | 8.1 | 2.7 | 3.4 | 7.4 | 4.7 | 1.4 | 2.0 | 4.1 | 5.4 | 3.4 | 6.8 | 2.7 | 3.4 | 7.4 | 12.5 | 6.1 | 6.1 | 2.7 | 5.4 |
| Q9KNF4 | 1.4 | 8.3 | 6.9 | 3.4 | 6.9 | 4.1 | 3.4 | 6.9 | 4.8 | 3.4 | 0.7 | 6.9 | 12.4 | 4.1 | 4.8 | 10.3 | 5.5 | 4.1 | 1.4 | 0.0 |
| Q9KT53 | 1.5 | 8.3 | 5.3 | 6.1 | 6.1 | 3.8 | 3.0 | 0.0 | 1.5 | 4.5 | 3.0 | 5.3 | 3.0 | 2.3 | 8.3 | 16.7 | 6.1 | 9.1 | 0.8 | 5.3 |
| Q9KL56 | 1.5 | 8.3 | 9.1 | 7.6 | 6.8 | 4.5 | 4.5 | 1.5 | 3.8 | 5.3 | 4.5 | 3.8 | 3.8 | 3.0 | 3.8 | 11.4 | 8.3 | 5.3 | 1.5 | 1.5 |
| Q9KRE6 | 4.0 | 4.0 | 4.8 | 3.2 | 9..5 | 4.0 | 2.4 | 6.3 | 4.0 | 7.1 | 4.8 | 4.8 | 7.1 | 2.4 | 4.0 | 15.9 | 7.9 | 2.4 | 1.6 | 0.0 |
| Q9KLX2 | 1.6 | 5.7 | 6.6 | 3.3 | 3.3 | 5.7 | 3.3 | 5.7 | 6.6 | 8.2 | 3.3 | 4.1 | 3.3 | 3.3 | 4.1 | 13.9 | 6.6 | 7.4 | 1.6 | 2.5 |
| Q9KLQ3 | 0.0 | 7.0 | 8.7 | 1.7 | 7.0 | 1.7 | 7.0 | 5.2 | 8.7 | 7.0 | 0.0 | 0.9 | 12.2 | 3.5 | 3.5 | 11.3 | 6.1 | 6.1 | 2.6 | 0.0 |
| Q9KKS6 | 0.9 | 6.2 | 4.4 | 1.8 | 8.0 | 7.1 | 5.3 | 5.3 | 8.8 | 0.9 | 4.4 | 3.5 | 8.8 | 4.4 | 5.3 | 8.8 | 2.7 | 5.3 | 7.1 | 0.9 |
| Q9KN87 | 2.7 | 3.6 | 5.5 | 4.5 | 7.3 | 2.7 | 3.6 | 7.3 | 9.1 | 5.5 | 0.9 | 2.7 | 10.9 | 3.6 | 3.6 | 6.4 | 6.4 | 9.1 | 2.7 | 1.8 |
| Q9KU58 | 0.0 | 8.7 | 4.8 | 5.8 | 5.8 | 3.8 | 2.9 | 3.8 | 5.8 | 1.9 | 2.9 | 15.4 | 2.9 | 1.9 | 6.7 | 12.5 | 8.7 | 1.9 | 1.9 | 1.9 |
| Q9KPP0 | 1.0 | 3.9 | 2.9 | 2.9 | 16.7 | 3.9 | 2.0 | 11.8 | 11.8 | 2.0 | 0.0 | 2.0 | 11.8 | 4.9 | 2.0 | 8.8 | 6.9 | 2.9 | 2.0 | 0.0 |
| BIB1N2 | 3.2 | 3.2 | 9.7 | 2.2 | 5.4 | 6.5 | 6.5 | 2.2 | 11.8 | 9.7 | 1.1 | 3.2 | 5.4 | 4.3 | 1.1 | 6.5 | 10.8 | 1.1 | 5.4 | 1.1 |
| Q9K2J6 | 0.0 | 6.7 | 5.6 | 2.2 | 18.9 | 1.1 | 4.4 | 5.6 | 6.7 | 5.6 | 0.0 | 4.4 | 7.8 | 3.3 | 4.4 | 13.3 | 7.8 | 1.1 | 1.1 | 0.0 |
| Q9KS64 | 1.2 | 5.8 | 4.7 | 5.8 | 7.0 | 4.7 | 8.1 | 2.3 | 9.3 | 1.2 | 4.7 | 2.3 | 7.0 | 3.5 | 2.3 | 10.5 | 14.0 | 1.2 | 2.3 | 2.3 |
| Q9KN40 | 5.8 | 10.5 | 7.0 | 1.2 | 7.0 | 4.7 | 4.7 | 7.0 | 9.3 | 1.2 | 1.2 | 1.2 | 11.6 | 5.8 | 2.3 | 7.0 | 3.5 | 2.3 | 2.3 | 4.7 |
| Q9KVW5 | 0.0 | 4.7 | 7.1 | 3.5 | 7.1 | 4.7 | 2.4 | 2.4 | 7.1 | 4.7 | 2.4 | 5.9 | 2.4 | 8.2 | 4.7 | 17.6 | 8.2 | 2.4 | 2.4 | 2.4 |
| Q9KL81 | 1.3 | 12.7 | 3.8 | 6.3 | 1.3 | 0.0 | 3.8 | 0.0 | 7.6 | 3.8 | 5.1 | 5.1 | 12.7 | 3.8 | 10.1 | 8.9 | 8.9 | 2.5 | 2.5 | 0.0 |
| Q9KPA0 | 1.3 | 2.6 | 2.6 | 3.9 | 5.2 | 6.5 | 2.6 | 3.9 | 6.5 | 5.2 | 2.6 | 10.4 | 13.0 | 5.2 | 5.2 | 6.5 | 5.2 | 6.5 | 5.2 | 0.0 |
| Q9KL73 | 1.5 | 4.5 | 1.5 | 1.5 | 9.0 | 4.5 | 4.5 | 1.5 | 13.4 | 6.0 | 3.0 | 3.0 | 17.9 | 4.5 | 1.5 | 6.0 | 6.0 | 6.0 | 4.5 | 0.0 |
| Q9KNG0 | 0.0 | 9.2 | 6.2 | 1.5 | 6.2 | 3.1 | 6.2 | 6.2 | 15.4 | 1.5 | 3.1 | 9.2 | 6.2 | 3.1 | 12.3 | 9.2 | 0.0 | 0.0 | 0.0 | 1.5 |
| Q9KSJ4 | 0.0 | 12.1 | 5.2 | 3.4 | 6.9 | 0.0 | 3.4 | 3.4 | 8.6 | 12.1 | 1.7 | 1.7 | 8.6 | 1.7 | 3.4 | 13.8 | 6.9 | 1.7 | 1.7 | 3.4 |
| Q9KPZ1 | 0.0 | 6.5 | 2.2 | 2.2 | 4.3 | 13.0 | 2.2 | 6.5 | 0.0 | 0.0 | 2.2 | 0.0 | 4.3 | 8.7 | 6.5 | 17.4 | 6.5 | 10.9 | 4.3 | 2.2 |
| Q9KNI6 | 0.0 | 6.5 | 6.5 | 0.0 | 10.9 | 6.5 | 4.3 | 4.3 | 10.9 | 4.3 | 0.0 | 4.3 | 8.7 | 4.3 | 6.5 | 8.7 | 6.5 | 4.3 | 2.2 | 0.0 |
| Q9KVT0 | 2.2 | 6.5 | 6.5 | 0.0 | 10.9 | 4.3 | 4.3 | 4.3 | 10.9 | 2.2 | 0.0 | 4.3 | 10.9 | 4.3 | 6.5 | 10.9 | 6.5 | 2.2 | 2.2 | 0.0 |
| Q9KST0 | 0.0 | 8.9 | 6.7 | 6.7 | 8.9 | 2.2 | 6.7 | 4.4 | 4.4 | 4.4 | 4.4 | 2.2 | 2.2 | 4.4 | 0.0 | 11.1 | 2.2 | 6.7 | 2.2 | 11.1 |

C: Cysteine (sulfhydrl group); STPAG: Serine, threonine, proline, alanine, glycine (small hydrophilic), respectively; NDEQ: Asparagine, aspartic acid, glutamic acid, glutamine (acid, acid amide and hydrophilic), respectively; HRK: Histidine, arginine, lysine (basic, positive hydrophilic), respectively; MILV: Methionine, Isoleucine, Leucine, Valine (small hydrophobic), respectively; FYW: Phenylalanine, Tyrosine, Tryptophan (aromatic), respectively.
