# Supplementary material for: Comprehensive in silico analyses of fifty-one uncharacterized proteins from Vibrio cholerae
Source: PLoS One. 2024 Oct 4;19(10):e0311301. doi: 10.1371/journal.pone.0311301 (PMC11452002; doi:10.1371/journal.pone.0311301)
Supplement: S4 Table — (DOCX) [file pone.0311301.s004.docx]

**Table S4**

**Subcellular localization of the uncharacterized proteins: -** The subcellular location of 51 uncharacterized proteins were assessed using PSORTb and PSLPred.

| **UniProt ID** | **Gene name** | **Subcellular localization (PSORTb)** | **PSORTb score** | **Subcellular localization (PSLpred)** | **Expected accuracy (PSLpred)** |
| --- | --- | --- | --- | --- | --- |
| Q9KRD2 | VC_1710 | Cytoplasmic Membrane | 7.88 | Cytoplasmic | 71.1% |
| Q9KVG3 | VC_0183v | Cytoplasmic membrane | 8.96 | Cytoplasmic | 71.1% |
| Q9KT38 | VC_1067 | Cytoplasmic | 9.59 | Cytoplasmic | 68.1% |
| Q9KKL8 | VC_A0185 | Cytoplasmic | 8.96 | Cytoplasmic | 90.2% |
| Q9KQX3 | VC_1874 | Cytoplasmic | 9.97 | Cytoplasmic | 68.1% |
| Q9KLK5 | VC_A0738 | Unknown | - | Outer-Membrane | 68.1% |
| Q9KT24 | VC_1081 | Cytoplasmic | 8.96 | Cytoplasmic | 98.1% |
| Q9KMS2 | VC_A0248 | Cytoplasmic | 9.97 | Cytoplasmic | 71.1% |
| Q9KMV6 | VC_A0212 | Cytoplasmic | 8.96 | Cytoplasmic | 71.1% |
| Q9KRM9 | VC_1607 | Unknown | -- | Periplasmic protein | 53.1% |
| Q9KU75 | VC_0648 | Cytoplasmic Membrane | 7.88 | Cytoplasmic | 68.1% |
| Q9KND1 | VC_A0034 | Cytoplasmic | 8.96 | Cytoplasmic | 71.1% |
| Q9KTC9 | VC_0973 | Unknown | -- | Periplasmic protein | 68.1% |
| Q9KSQ9 | VC_1197 | Unknown | -- | Periplasmic protein | 53.2% |
| Q9KS60 | VC­_1400 | Cytoplasmic | 8.96 | Cytoplasmic | 90.2% |
| Q9KKX0 | VC_A0980 | Cytoplasmic | 8.96 | Cytoplasmic | 53.1% |
| Q9KND9 | VC_A0026 | Unknown | -- | Periplasmic protein | 53.1% |
| Q9KRJ5 | VC_1645 | Cytoplasmic | 8.96 | Cytoplasmic | 71.1% |
| Q9KVJ9 | VC_0144 | Cytoplasmic Membrane | 10.00 | Inner Membrane | 90.2 |
| Q9KSV3 | VC_1153 | Cytoplasmic | 8.96 | Cytoplasmic | 71.1% |
| Q9KSV6 | VC_1150 | Unknown | -- | Periplasmic protein | 71.1% |
| Q9KND3 | VC_A0032 | Unknown | -- | Inner-Membrane | 68.1% |
| Q9KP29 | VC_2550 | Cytoplasmic Membrane | 9.86 | Inner-Membrane | 53.1% |
| Q9KMX1 | VC_A0195 | Outer Membrane | 9.52 | Outer Membrane | 53.1% |
| Q9KTE5 | VC_0957 | Cytoplasmic | 8.96 | Cytoplasmic | 71.1% |
| Q9KPD6 | VC_2434 | Cytoplasmic | 8.96 | Periplasmic protein | 53.1% |
| Q9KPA3 | VC_2470 | Cytoplasmic membrane | 9.82 | Inner membrane | 90.2% |
| Q9KNF4 | VC_A0010 | Cytoplasmic | 8.96 | Cytoplasmic/ Periplasmic | 53.1% |
| Q9KT53 | VC_1052 | Cytoplasmic membrane | 10.00 | Inner-membrane | 71.1% |
| Q9KL56 | VC_A0892 | Unknown | -- | Periplasmic protein | 53.1% |
| Q9KRE6 | VC_1696 | Unknown | -- | Cytoplasmic | 90.2% |
| Q9KLX2 | VC_A0619 | Unknown | -- | Cytoplasmic | 68.1% |
| Q9KLQ3 | VC_A0689 | Unknown | -- | Periplasmic protein | 53.1% |
| Q9KKS6 | VC_A1024 | Unknown | - | Cytoplasmic | 68.1% |
| Q9KN87 | VC_A0078 | Unknown | -- | Cytoplasmic | 68.1% |
| Q9KU58 | VC_0666 | Unknown | -- | Inner-membrane | 53.1% |
| Q9KPP0 | VC_2326 | Unknown | -- | Periplasmic protein | 68.1% |
| B1B1N2 | VC_A0594 | Unknown | - | Periplasmic protein | 53.1% |
| Q9K2J6 | VC_A0319 | Unknown | -- | Periplasmic protein | 68.1% |
| Q9KS64 | VC_1396 | Unknown | -- | Cytoplasmic | 71.1% |
| Q9KN40 | VC_A0125 | Unknown | -- | Periplasmic protein | 53.1% |
| Q9KVW5 | VC_0023 | Cytoplasmic Membrane | 9.82 | Inner Membrane | 71.1% |
| Q9KL81 | VC_A0866 | Unknown | -- | Cytoplasmic | 53.1% |
| Q9KPA0 | VC_2473 | Unknown | - | Cytoplasmic | 53.1% |
| Q9KL73 | VC_A0874 | Unknown | -- | Outer Membrane | 53.1% |
| Q9KNG0 | VC_A0004 | Unknown | - | Cytoplasmic | 53.1% |
| Q9KSJ4 | VC_1262 | Unknown | -- | Inner Membrane | 53.1% |
| Q9KPZ1 | VC_2221 | Cytoplasmic membrane | 9.82 | Inner-membrane | 68.1% |
| Q9KNI6 | VC_2753 | Unknown | - | Cytoplasmic | 68.1% |
| Q9KVT0 | VC_0059 | Unknown | -- | Cytoplasmic | 68.1% |
| Q9KST0 | VC_1176 | Unknown | -- | Extracellular protein | 53.1% |
