# Supplementary material for: Comprehensive in silico analyses of fifty-one uncharacterized proteins from Vibrio cholerae
Source: PLoS One. 2024 Oct 4;19(10):e0311301. doi: 10.1371/journal.pone.0311301 (PMC11452002; doi:10.1371/journal.pone.0311301)
Supplement: S5 Table — (DOCX) [file pone.0311301.s005.docx]

**Table S5**

**Identification of domains of 51 uncharacterized proteins using InterPro, SMART and PROSITE.**

| **UniProt ID** | **Gene name** | **Pfam/InterPro** | | **SMART** | | **PROSITE** | |
| --- | --- | --- | --- | --- | --- | --- | --- |
|  |  | **Domain** | **Residue** | **Domain** | **Residue** | **Domain** | **Residue** |
| Q9KRD2 | VC_1710 | EAL | 422-656 | EAL | 414-661 | EAL | 415-670 |
|  |  | PAS | 138-235 | PAS | 126-192 | PAS | 124-168 |
| Q9KVG3 | VC_0183 |  | |  | |  | |
| Q9KT38 | VC_1067 | Bacterial extracellular solute-binding protein | 66-247 | Bacterial  Peri-  plasmic  Substrate binding protein | 50-251 | GGDEF | 557-686 |
|  |  |  | 276-465 |  | 270-517 |  |  |
|  |  | Diguanylate cyclase, GGDEF domain | 533-676 | GGDEF | 522-683 |  |  |
| Q9KKL8 | VC_A0185 | Flagellar assembly Protein A beta solenoid domain | 267-543 |  | |  | |
|  |  | Flagellar Assembly Protein A N-terminal domain | 80-260 |  |  |  |  |
| Q9KQX3 | VC_1874 | SpoVR like protein | 43-453 |  | |  | |
| Q9KLK5 | VC_A0738 | F plasmid transfer operon, TraF | 76-330 |  | |  | |
| Q9KT24 | VC_1081 | Response regulator receiver domain | 8-113 | CheY-homo-logous receiver domain | 6-117 | Response regulator receiver domain | 7-121 |
|  |  | HDOD | 148-278 |  |  | HDOD | 142-326 |
| Q9KMS2 | VC_A0248 | Beta-lactamase superfamily domain | 42-282 |  | |  | |
| Q9KMV6 | VC_A0212 |  | |  | |  | |
| Q9KRM9 | VC_1607 | Barrel-sandwich domain of CusB or HlyD membrane fusion | 40-275 |  | |  | |
| Q9KU75 | VC_0648 | Tetra-tricopeptide repeat | 101-133 | Tetra-  tricopeptide repeat | 66-99 | Tetra-tricopeptide repeat | 66-99 |
|  |  |  |  |  | 100-133 |  |  |
|  |  |  |  |  | 134-167 |  | 100-133 |
|  |  |  |  |  | 237-270 |  | 237-270 |
| Q9KND1 | VC_A0034 | HDOD | 27-217 |  | | HDOD | 27-218 |
| Q9KTC9 | VC_0973 |  | |  | |  | |
| Q9KSQ9 | VC_1197 | Domain of Unknown Function | 7-231 |  | |  | |
| Q9KS60 | VC­_1400 | HEAT repeats | 129-192 |  | |  | |
| Q9KKX0 | VC_A0980 |  | |  | |  | |
| Q9KND9 | VC_A0026 | Domain of Unknown Function | 24-206 |  | |  | |
| Q9KRJ5 | VC_1645 | Haloacid dehalogenase-like hydrolase | 5-178 |  | |  | |
| Q9KVJ9 | VC_0144 | YhhN domain | 28-207 |  | |  | |
| Q9KSV3 | VC_1153 | TfoX N-terminal domain | 15-106 |  | |  | |
|  |  | TfoX C-terminal domain | 119-197 |  |  |  |  |
| Q9KSV6 | VC_1150 |  | |  | |  | |
| Q9KND3 | VC_A0032 |  | |  | |  | |
| Q9KP29 | VC_2550 | Domain of Unknown Function | 24-182 |  | |  | |
| Q9KMX1 | VC_A0195 |  | |  | |  | |
| Q9KTE5 | VC_0957 | Zinc Ribbon containing domain | 14-153 |  | |  | |
| Q9KPD6 | VC_2434 | Domain of Unknown Function | 27-139 |  | |  | |
| Q9KPA3 | VC_2470 |  | |  | |  | |
| Q9KNF4 | VC_A0010 | Domain of Unknown Function | 92-143 |  | |  | |
| Q9KT53 | VC_1052 | Domain of Unknown Function | 7-121 |  | |  | |
| Q9KL56 | VC_A0892 |  | |  | |  | |
| Q9KRE6 | VC_1696 | Domain of Unknown Function | 50-125 |  | |  | |
| Q9KLX2 | VC_A0619 |  | |  | |  | |
| Q9KLQ3 | VC_A0689 | Phasin protein | 8-108 |  | |  | |
| Q9KKS6 | VC_A1024 | Domain of Unknown Function | 35-110 |  | |  | |
| Q9KN87 | VC_A0078 |  | |  | |  | |
| Q9KU58 | VC_0666 |  | |  | |  | |
| Q9KPP0 | VC_2326 | YebG | 1-72 |  | |  | |
| B1B1N2 | VC_A0594 | Domain of Unknown Function | 43-89 |  | |  | |
| Q9K2J6 | VC_A0319 | Domain of Unknown Function | 7-86 |  | |  | |
| Q9KS64 | VC_1396 |  | |  | |  | |
| Q9KN40 | VC_A0125 | Domain of Unknown Function | 20-51 |  | |  | |
| Q9KVW5 | VC_0023 |  | |  | |  | |
| Q9KL81 | VC_A0866 |  | |  | |  | |
| Q9KPA0 | VC_2473 | Domain of unknown function | 10-72 |  | |  | |
| Q9KL73 | VC_A0874 |  | |  | |  | |
| Q9KNG0 | VC_A0004 |  | |  | |  | |
| Q9KSJ4 | VC_1262 |  | |  | |  | |
| Q9KPZ1 | VC_2221 | Domain of Unknown function | 4-43 |  | |  | |
| Q9KNI6 | VC_2753 |  | |  | |  | |
| Q9KVT0 | VC_0059 |  | |  | |  | |
| Q9KST0 | VC_1176 |  | | Tryptophan operon leader peptide | | 10-45 | |
