# Supplementary material for: Comprehensive in silico analyses of fifty-one uncharacterized proteins from Vibrio cholerae
Source: PLoS One. 2024 Oct 4;19(10):e0311301. doi: 10.1371/journal.pone.0311301 (PMC11452002; doi:10.1371/journal.pone.0311301)
Supplement: S6 Table — (DOCX) [file pone.0311301.s006.docx]

**Table S6**

**Prediction of soluble and transmembrane protein and determination of transmembrane region present within the uncharacterized proteins**

| **UniProt ID** | **Soluble/**  **Membrane protein**  **(SOUSUI)** | **Transmembrane Region**  **(SOSUI)** | | | **Deep-**  **THMM**  **Prediction** | **Transmembrane Region**  **(DeepTMHMM)** | | | | | **HMMTOP Prediction** | **Transmembrane Region**  **(HMMTOP)** | |
| --- | --- | --- | --- | --- | --- | --- | --- | --- | --- | --- | --- | --- | --- |
|  |  | **Sequence** | **Region** | **Type** |  | **Inner** | **TM-**  **helix** | **Beta-**  **barrel** | **Outer** | **Periplasm** |  | **Sequence** | **Region** |
| **Q9KRD2** | Soluble |  | | | Globula**r** | 1-818 |  |  |  |  | Soluble |  | |
| **Q9KVG3** | Soluble |  | | | Globular | 1-703 |  |  |  |  | Soluble |  | |
| **Q9KT38** | Membrane | VFLWLG  WLLISTSSLAATSTTYK | 30-52 | Secondary | Trans-  Membrane  helix | 530-693 | 513-529 |  | 46-512 |  | Trans-membrane helix | IAIGFAKNPLGATLAPLFS | 458-476 |
|  |  | WLFAMAFIVLFMVAFYLHGISHT | 513-535 | Primary |  |  |  |  |  |  |  | TQWLFAMAFIVLFMVAFYL | 511-529 |
| **Q9KKL8** | Soluble |  | | | Globular | 1-556 |  |  |  |  | Soluble |  | |
| **Q9KQX3** | Soluble |  | | | Globular | 1-516 |  |  |  |  | Soluble |  | |
| **Q9KLK5** | Soluble |  | | | Beta-barrel |  |  | 94-101 | 102-153 | 51-93 | Soluble |  | |
|  |  |  |  |  |  |  |  | 154-164 | 178-213 | 165-168 |  |  |  |
|  |  |  |  |  |  |  |  | 169-177 | 244-267 | 224-231 |  |  |  |
|  |  |  |  |  |  |  |  | 214-223 | 294-311 | 279-283 |  |  |  |
|  |  |  |  |  |  |  |  | 232-243 | 331-345 | 319-323 |  |  |  |
|  |  |  |  |  |  |  |  | 268-278 | 368-374 | 354-358 |  |  |  |
|  |  |  |  |  |  |  |  | 284-293 | 398-402 | 384-388 |  |  |  |
|  |  |  |  |  |  |  |  | 312-318 |  | 412-413 |  |  |  |
|  |  |  |  |  |  |  |  | 324-330 |  |  |  |  |  |
|  |  |  |  |  |  |  |  | 346-353 |  |  |  |  |  |
|  |  |  |  |  |  |  |  | 359-367 |  |  |  |  |  |
|  |  |  |  |  |  |  |  | 375-383 |  |  |  |  |  |
|  |  |  |  |  |  |  |  | 389-397 |  |  |  |  |  |
|  |  |  |  |  |  |  |  | 403-411 |  |  |  |  |  |
| **Q9KT24** | Soluble |  | | | Globular | 1-380 |  |  |  |  | Soluble |  | |
| **Q9KMS2** | Soluble |  | | | Globular | 1-355 |  |  |  |  | Soluble |  | |
| **Q9KMV6** | Soluble |  | | | Globular | 43-349 |  |  |  |  | Trans  membrane helix | WMVIVWLLSGQAWAANVLVI | 29-48 |
| **Q9KRM9** | Soluble |  | | | Globular | 28-324 |  |  |  |  | Trans-  membrane helix | TTLMT LTAVAVVGWI GY | 6-22 |
| **Q9KU75** | Membrane | WFRFPIAVIGLVLLSGCATQNE | 3-24 | Primary | Globular |  |  |  | 19-303 |  | Soluble |  | |
| **Q9KND1** | Soluble |  | | | Globular | 1-290 |  |  |  |  | Soluble |  | |
| **Q9KTC9** | Soluble |  | | | Globular | 20-271 |  |  |  |  | Soluble |  | |
| **Q9KSQ9** | Soluble |  | | | Globular | 1-231 |  |  |  |  | Soluble |  | |
| **Q9KS60** | Soluble |  | | | Globular | 1-218 |  |  |  |  | Soluble |  | |
| **Q9KKX0** | Membrane | KVASIVQIVFAVCAVLLALTVFE | 6-28 | Primary | Trans-membrane  helix | 1-12 | 13-27 |  | 28-163 |  | Soluble | VASIVQIVFAVCAVLLALTV | 7-26 |
|  |  | LLKVLILAILIPCMLFIILNFSV | 164-186 | Primary |  | 185-214 | 164-287 |  |  |  |  | VLIL AILIPCMLFI ILNFSVL | 167-187 |
| **Q9KND9** | Soluble |  | | | Globular |  |  |  | 22-211 |  |  | LTCLLALCFAASAS AKVTM | 7-25 |
| **Q9KRJ5** | Soluble |  | | | Globular | 1-209 |  |  |  |  |  |  | |
| **Q9KVJ9** | Membrane | \| MSMLSW  ISVVLSG  FISISAYENQ \| \| --- \| | 1-23 | Primary | Trans-membrane  helix | 20-28 | 4-19 |  | 1-3 |  |  | LSWISVVLSGFISISAY | 4-20 |
|  |  | KQAIFFRIFSLLLLTIIVWEQH | 26-47 | Secondary |  | 73-78 | 29-44 |  | 45-51 |  |  | IFFRIFSLLLLTIIVWE | 29-45 |
|  |  | ATPEVIFISLGLAVSMFAHGLRL | 50-72 | Secondary |  | 124-130 | 52-72 |  | 98-104 |  |  | VIFISLGLAVSMFAHGL | 54-70 |
|  |  | HKASFVLFLVAQLLFSKAFWVQL | 77-99 | Primary |  | 180-186 | 79-97 |  | 149-159 |  |  | ASFVLFLVAQLLFSKAF | 79-95 |
|  |  | VAASIVAFFLLLPQIDTLIFPVT | 112-134 | Primary |  |  | 105-123 |  | 208-209 |  |  | VWWLPALLVAASIVAFF L | 104-121 |
|  |  | ASGAGFLGCLVYILSATLLAIHD | 158-180 | Primary |  |  | 131-148 |  |  |  |  | IFPVTIMGLMLVQMTWA | 130-146 |
|  |  | GHTLISSSYLIAQALISASIVF | 188-209 | Primary |  |  | 160-179 |  |  |  |  | ATVASGAGFLGCLVYILSATLLAI | 155-178 |
|  |  |  |  |  |  |  | 187-207 |  |  |  |  | LISSSYLIAQALISASIV | 191-208 |
| **Q9KSV3** | Soluble |  | | | Globular | 1-202 |  |  |  |  |  |  | |
| **Q9KSV6** | Soluble |  | | | Globular |  |  |  | 52-201 |  |  | LSTVL LMSVVSASAYADNTCF | 36-56 |
| **Q9KND3** | Soluble |  | | | Globular |  |  |  | 18-186 |  |  |  | |
| **Q9KP29** | Soluble |  | | | Globular |  |  |  | 21-183 |  |  | LIALLLACSPTLAFAHNLTVG | 6-25 |
| **Q9KMX1** | Membrane | \| MVRFCG  WLMVFC  CFALPAY \| \| --- \| | 1-19 | Primary | Beta-barrel |  |  | 26-33 | 34-46 | 21-25 |  | GWLMVFCCFALPAYAGNSPY | 6-25 |
|  |  |  |  |  |  |  |  | 47-54 | 68-91 | 55-59 |  | FSPFIGLDLGYSGMIGNG | 56-73 |
|  |  |  |  |  |  |  |  | 60-67 | 114-137 | 99-105 |  | FYFGAFFEQPINNLTILYA | 90-109 |
|  |  |  |  |  |  |  |  | 92-98 |  | 147-153 |  |  | |
|  |  |  |  |  |  |  |  | 106-113 |  | 181-182 |  |  | |
|  |  |  |  |  |  |  |  | 138-146 |  |  |  |  | |
|  |  |  |  |  |  |  |  | 154-163 |  |  |  |  | |
|  |  |  |  |  |  |  |  | 172-180 |  |  |  |  | |
| **Q9KTE5** | Soluble |  | | | Globular | 1-156 |  |  |  |  |  |  | |
| **Q9KPD6** | Soluble |  | | | Globular | 1-148 |  |  |  |  |  |  | |
| **Q9KPA3** | Membrane | ITSAKSVKLLFIPSFYAQMIALL | 11-33 | Primary | Trans-membrane  helix | 1-26 | 27-44 |  | 45-46 |  |  | LLFIPSFYAQMIALLIMLLMLWALF | 19-43 |
|  |  | ALFVSSIPLVLVPYFAAWIWRGH | 41-63 | Primary |  | 61-148 | 47-60 |  |  |  |  |  | |
| **Q9KNF4** | Soluble |  | | | Globular | 1-145 |  |  |  |  |  |  | |
| **Q9KT53** | Membrane | GITSLCLGILGIFLLPLLPTTPFI | 14-36 | Primary | Trans-membrane  helix | 1-7 | 8-28 |  | 29-34 |  |  | LGILGIFLPLLPTTPFILLSS ACFL | 20-44 |
|  |  |  |  |  |  | 45-82 | 35-44 |  | 97-98 |  |  | LFIVLSFAFSIWMVPWLWLKVALFI | 83-107 |
|  |  | \| SFAFSIWM  VPWLWLK  VALFIWLV \| \| --- \| | 88-110 | Primary |  | 118-132 | 83=96 |  |  |  |  |  | |
|  |  |  |  |  |  |  | 99-117 |  |  |  |  |  | |
| **Q9KL56** | Membrane | FHRLMLKLSLLSWLLVSLMPVLN | 24-46 | Primary | Globular | 48-132 |  |  |  |  |  | LSLLSWLLVSLMPVNAHGNAAGVW | 31-55 |
| **Q9KRE6** | Soluble |  | | | Globular | 1-126 |  |  |  |  |  |  | |
| **Q9KLX2** | Soluble |  | | | Globular | 1-122 |  |  |  |  |  |  | |
| **Q9KLQ3** | Soluble |  | | | Globular | 1-115 |  |  |  |  |  |  | |
| **Q9KKS6** | Soluble |  | | | Globular | 1-113 |  |  |  |  |  |  | |
| **Q9KN87** | Soluble |  | | | Globular | 1-110 |  |  |  |  |  |  | |
| **Q9KU58** | Soluble |  | | | Globular | 1-104 |  |  |  |  |  |  | |
| **Q9KPP0** | Soluble |  | | | Globular | 1-102 |  |  |  |  |  |  | |
| **B1B1N2** | Membrane | KTALLLTVVGAVLLTGCARQE | 13-33 | Primary | Globular |  |  |  | 29-93 |  |  | TALLLTVVGAVLLTGCA | 14-30 |
| **Q9K2J6** | Soluble |  | | | Globular | 1-90 |  |  |  |  |  | AAALAGMSSINSFVLNAA I | 23-41 |
| **Q9KS64** | Soluble |  | | | Globular | 1-86 |  |  |  |  |  |  | |
| **Q9KN40** | Soluble |  | | | Globular | 1-86 |  |  |  |  |  |  | |
| **Q9KVW5** | Membrane | YLFSFGLVLTLLGMALTDLWMPM | 15-37 | Primary | Trans-membrane  helix | 1-14 | 15-28 |  | 29-37 |  |  | GLVLTLLGMALTDLWMMVVG AIVM | 20-44 |
|  |  | AIVMTALAVESWIRVAHIIPLHD | 41-63 | Primary |  | 50-85 | 38-49 |  |  |  |  |  | |
| **Q9KL81** | Soluble |  | | | Globular | 1-79 |  |  |  |  |  |  | |
| **Q9KPA0** | Soluble |  | | | Globular | 1-77 |  |  |  |  |  |  | |
| **Q9KL73** | Soluble |  | | | Globular | 1-67 |  |  |  |  |  |  | |
| **Q9KNG0** | Soluble |  | | | Globular | 1-65 |  |  |  |  |  |  | |
| **Q9KSJ4** | Soluble |  | | | Globular | 1-58 |  |  |  |  |  |  | |
| **Q9KPZ1** | Membrane | NAVGLSSMIVILGALGLMLFYGG | 11-33 | Primary | Trans-membrane  helix | 37-46 | 18-36 |  | 1-17 |  |  | SMIVIGALGLMLFYGGFFI | 17-36 |
| **Q9KNI6** | Soluble |  | | | Globular | 1-46 |  |  |  |  |  |  | |
| **Q9KVT0** | Soluble |  | | | Globular | 1-46 |  |  |  |  |  |  | |
| **Q9KST0** | Soluble |  | | | Globular |  |  |  | 31-45 |  |  |  | |
