# Supplementary material for: Comprehensive in silico analyses of fifty-one uncharacterized proteins from Vibrio cholerae
Source: PLoS One. 2024 Oct 4;19(10):e0311301. doi: 10.1371/journal.pone.0311301 (PMC11452002; doi:10.1371/journal.pone.0311301)
Supplement: S7 Table — (DOCX) [file pone.0311301.s007.docx]

**Table S7**

**Prediction of Protein Function using PFP (Protein Function Prediction) Server:** The molecular function, biological function and cellular location was predicted for each of the proteins using Protein Function Prediction (PFP) server as depicted by PFP score.

| **UniProt ID** | **Protein Function Prediction** | | | | | |
| --- | --- | --- | --- | --- | --- | --- |
|  | **Molecular Function Terms** | **PFP Score** | **Biological Process Terms** | **PFP Score** | **Cellular Component Terms** | **PFP Score** |
| Q9KRD2 | Phosphotransferase activity | 2673.47 | Regulation of nucleobase containing compound metabolic process | 4185.22 | Membrane | 3929.97 |
| Q9KVG3 | ATP Binding | 9.41 | Riboflavin biosynthetic process | 2.29 | Cytoplasm | 4.62 |
| Q9KT38 | Peptidoglycan muralytic | 5483.34 | Cell wall organization | 4843.32 | Membrane | 5486.11 |
| Q9KKL8 | ATP Binding | 48.25 | Cell morphogenesis | 67.72 | Cytoplasm | 91.42 |
| Q9KQX3 | ATP Binding | 36.03 | Nucleic acid templated transcription | 12.91 | Intracellular | 34.17 |
| Q9KLK5 | Methylthioadenosine nucleosidase activity | 93.10 | L-methionine salvage | 171.93 | Virus tail | 84.81 |
| Q9KT24 | DNA Binding | 9007.94 | Phosphorelay signal transduction | 12454.55 | Cytoplasm | 13802.65 |
| Q9KMS2 | Hydrolase | 14120.19 | L-Ascorbic Acid catabolism | 5585.98 | Cytoplasm | 6117.96 |
| Q9KMV6 | ATP Binding | 20.62 | One-carbon metabolism | 11.52 | Cytoplasm | 19.38 |
| Q9KRM9 | Transmembrane transporter | 6093.75 | Response to antibiotic | 1064.98 | Membrane | 7658.92 |
| Q9KU75 | Heat-shock protein binding | 915.76 | Photosynthesis | 3586.46 | Intracellular | 5503.45 |
| Q9KND1 | RNA Binding | 3319.03 | mRNA catabolism | 3317.88 | Membrane | 4081.64 |
| Q9KTC9 | ATP Binding | 23.06 | Entry into Host | 11.05 | Intrinsic component of Membrane | 24.99 |
| Q9KSQ9 | RNA Binding | 53.32 | RNA processing | 51.60 | Cytoplasm | 70.61 |
| Q9KS60 | ATP Binding | 16.46 | Regulation of Macromolecule metabolism | 10.21 | Cytoplasm | 35.01 |
| Q9KKX0 | C-C Chemokine receptor activity | 34.71 | Chemotaxis | 37.00 | Cytoplasm | 48.37 |
| Q9KND9 | Metal-ion Binding | 10.56 | Translation | 14.01 | Plasma Membrane | 19.71 |
| Q9KRJ5 | Magnesium-ion Binding | 9784.64 | Amino-acid salvage | 16927.73 | Cytoplasm | 2919.59 |
| Q9KVJ9 | Alkenylglycerophosphoethanolamine hydrolase activity | 222.38 | Ether lipid metabolism | 149.63 | Membrane | 483.31 |
| Q9KSV3 | ATP Binding | 22.34 | Establishment of competence for transformation | 309.01 | Intracellular | 18.92 |
| Q9KSV6 | ATP Binding | 9.77 | Positive regulation of receptor signalling pathway via STAT | 6.87 | Intracellular | 12.03 |
| Q9KND3 | Metal-ion Binding | 29.55 | Translation | 11.51 | Cytoplasm | 36.41 |
| Q9KP29 | DNA Binding | 19.16 | Transcription | 14.91 | Periplasmic Space | 183.90 |
| Q9KMX1 | Porin Activity | 23.87 | Ion-transport | 25.23 | Outer Membrane | 24.67 |
| Q9KTE5 | ATP Binding | 274.70 | AMP-salvage | 237.67 | Cytoplasm | 291.57 |
| Q9KPD6 | Phosphoric diester hydrolase activity | 8.19 | Lipid Metabolism | 9.50 | Plasma Membrane | 11.45 |
| Q9KPA3 | Glucose-6-Phosphate isomerase | 4.79 | Glycolytic process | 4.79 | Intrinsic component of Membrane | 7.26 |
| Q9KNF4 | ATP Binding | 50.17 | Translation | 27.77 | Cytosol | 60.83 |
| Q9KT53 | ATP Binding | 7.41 | Fatty acid biosynthesis | 5.87 | Membrane | 187.96 |
| Q9KL56 | Tachykinin Receptor | 4.49 | Positive regulation of flagellated sperm motility | 3.23 | Membrane | 8.53 |
| Q9KRE6 | Protein dimerization | 24.06 | Negative regulation of gene expression | 51.54 | Cytoplasm | 35.12 |
| Q9KLX2 | Translation Elongation Factor | 107.40 | Response to tumor necrosis factor | 9.43 | Cytoplasm | 128.09 |
| Q9KLQ3 | ATP Binding | 37.65 | Suppression by symbiont of host transmembrane receptor-mediated signal transduction | 25.03 | Cytoplasm | 44.94 |
| Q9KKS6 | ATP Binding | 9.08 | Translation | 8.67 | Cytoplasm | 16.84 |
| Q9KN87 | DNA Binding | 30.62 | tRNA modification | 7.76 | Cytoplasm | 18.11 |
| Q9KU58 | Kinase Binding | 14.39 | Protein stabilization | 10.15 | Cytoplasm | 21.27 |
| Q9KPP0 | ATP Binding | 25.01 | Cellular response to DNA damage stimulus | 41.68 | Cytosol | 40.23 |
| B1B1N2 | ATP Binding | 29.82 | Translation | 21.52 | Membrane | 18.18 |
| Q9K2J6 | Metal-ion Binding | 11.69 | Regulation of Transcription | 54.46 | Cytoplasm | 6.32 |
| Q9KS64 | Ion Binding | 9.23 | Protein Initiator Methionine Removal | 5.38 | Cytoplasm | 14.72 |
| Q9KN40 | Methylthioribulose 1-phosphate dehydratase | 6.33 | L-methionine salvage | 11.70 | Cytoplasm | 11.30 |
| Q9KVW5 | ATP Binding | 16.73 | Cellular amide metabolism | 11.13 | Intracellular | 40.08 |
| Q9KL81 | Cation Binding | 8.16 | Translation | 9.20 | Cytoplasm | 23.72 |
| Q9KPA0 | Carnitine O-octanoyltransferase | 4.78 | Fatty Acid Metabolism | 5.40 | Microbody | 4.89 |
| Q9KL73 | Unfolded Protein Binding | 19.14 | Protein Folding | 19.14 | Cytoplasm | 33.77 |
| Q9KNG0 | ATP Binding | 44.24 | rRNA base methylation | 26.52 | Cytoplasm | 83.22 |
| Q9KSJ4 | ATP Binding | 14.91 | Endoplasm Reticulum Organization | 8.08 | Cytoplasm | 17.35 |
| Q9KPZ1 | Motor Activity | 1.22 | Cell wall organization | 1.59 | Membrane | 8.34 |
| Q9KNI6 | ATP Binding | 8.25 | Cellular response to DNA damage | 5.49 | Nucleus | 5.11 |
| Q9KVT0 | DNA Binding | 4.21 | Cellular response to DNA damage | 1.67 | Cytoplasm | 2.09 |
| Q9KST0 | ATP Binding | 5.14 | Leucine Biosynthesis | 3.44 | Cytoplasm | 12.75 |
