# Supplementary material for: Comprehensive in silico analyses of fifty-one uncharacterized proteins from Vibrio cholerae
Source: PLoS One. 2024 Oct 4;19(10):e0311301. doi: 10.1371/journal.pone.0311301 (PMC11452002; doi:10.1371/journal.pone.0311301)
Supplement: S8 Table — (DOCX) [file pone.0311301.s008.docx]

**Table S8**

**Prediction of Protein Function using Argot2 Server:** The molecular function, biological function and cellular location was predicted for each of the proteins using Argot2 server as depicted by Argot2 score.

| **UniProt ID** | **Argot2** | | | | | |
| --- | --- | --- | --- | --- | --- | --- |
|  | **Molecular Function Terms** | **Argot2 Score** | **Biological Process Terms** | **Argot2 Score** | **Cellular Component Terms** | **Argot2 Score** |
|  |  |  |  |  |  |  |
| Q9KRD2 | Catalytic | 665.351 | Regulation of transcription | 11271.5 | Integral component of membrane | 825.271 |
| Q9KVG3 | DNA binding | 11459.4 | DNA integration | 7255.79 | Viral capsid | 20.325 |
| Q9KT38 | Nucleotidyl transferase | 3054.25 | Phosphorelay signal transduction | 101.884 | Integral component of membrane | 2609.8 |
| Q9KKL8 | Hydrolase | 96.132 | Cell morphogenesis | 26599.5 | Integral component of membrane | 76.2844 |
| Q9KQX3 | Acetyl-CoA Carboxylase | 2750.02 | Hydrogen-ion transmembrane transport | 2715.06 | Unknown | NA |
| Q9KLK5 | Catalytic | 83.6734 | Oxidation-reduction | 418.049 | Integral component of membrane | 2044.39 |
| Q9KT24 | Phosphorelay response regulator | 1827.9 | Phosphorelay signal transduction | 18235.3 | Intracellular | 4799.06 |
| Q9KMS2 | Carboxylic ester hydrolase | 3908.64 | L-ascorbic acid catabolism | 38638.5 | Cytoplasm | 9674.2 |
| Q9KMV6 | Phosphorelay sensor kinase | 131.765 | Carbohydrate transport | 18411.9 | Integral component of Membrane | 467.659 |
| Q9KRM9 | Transferase | 11.4804 | Protein secretion | 20211.6 | Membrane | 2492.88 |
| Q9KU75 | Unknown | NA | Unknown | NA | Plasma membrane | 6181.8 |
| Q9KND1 | Kinase | 10449.2 | Phosphorylation | 17347.1 | Intracellular | 118.475 |
| Q9KTC9 | Unknown | NA | Unknown | NA | Integral component of membrane | 1394.9 |
| Q9KSQ9 | Transferase | 5.15896 | Phosphorelay signal transduction | 40.9944 | Integral component of membrane | 1165.54 |
| Q9KS60 | Lyase | 483.616 | Unknown |  | Proteasome complex | 6.74105 |
| Q9KKX0 | Signal transducer | 6515.27 | Chemotaxis | 5786.09 | Integral component of membrane | 1342.61 |
| Q9KND9 | Hydrolase | 966.297 | Proteolysis | 541.669 | Integral component of membrane | 106.007 |
| Q9KRJ5 | Hydrolase | 2677.84 | Metabolism | 615.292 | Integral component of membrane | 67.2731 |
| Q9KVJ9 | Oxidoreductase | 18.5456 | rRNA methylation | 154.949 | Integral component of membrane | 2788.65 |
| Q9KSV3 | Zinc-ion binding | 64.2428 | Establishment of competence for transformation | 1553.85 | Integral component of membrane | 1229.01 |
| Q9KSV6 | Ferric ion binding | 140.453 | Iron ion transport | 113.469 | Cell | 59.5166 |
| Q9KND3 | Signal transducer | 93.4518 | Signal transduction | 161.955 | Integral component of membrane | 133.81 |
| Q9KP29 | DNA binding | 89.2807 | Cell redox homeostasis | 1229.15 | Periplasmic space | 1670.55 |
| Q9KMX1 | Unknown | NA | Transmembrane transport | 6.26808 | Integral component of membrane | 4079.66 |
| Q9KTE5 | DNA binding | 101.4 | Transcription | 119.72 | RNA Pol I core factor complex | 167.084 |
| Q9KPD6 | Hydrolase | 1397.04 | Phosphorylation | 1383.54 | Unknown | NA |
| Q9KPA3 | Unknown | NA | Unknown | NA | Integral component of membrane | 964.935 |
| Q9KNF4 | Catalytic | 87.0105 | Bacterial-type flagellum assembly | 1201.11 | Integral component of membrane | 40.2777 |
| Q9KT53 | ATPase | 68.8186 | Nucleoside metabolism | 393.802 | Integral component of membrane | 1256.27 |
| Q9KL56 | Unknown | NA | Unknown | NA | Integral component of membrane | 10.431 |
| Q9KRE6 | DNA binding | 3134.19 | Cellular response to phosphate starvation | 3835.81 | Integral component of membrane | 697.717 |
| Q9KLX2 | Lyase | 1933.27 | Oxidation-reduction | 2844.53 | Unknown | NA |
| Q9KLQ3 | Peptidase | 548.9 | Proteolysis | 730.604 | Cytoplasm | 9.06629 |
| Q9KKS6 | Unknown | NA | Unknown | NA | Unknown | NA |
| Q9KN87 | 1-4-alpha glucan branching enzyme | 753.186 | Phosphorylation | 3694.1 | Unknown | NA |
| Q9KU58 | Unknown | NA | Unknown | NA | Integral component of membrane | 211.993 |
| Q9KPP0 | DNA binding | 963.214 | SOS response | 540.768 | Cytosol | 1491.1 |
| B1B1N2 | Serine-type endopeptidase inhibitor | 855.549 | Hemolysis of symbiont by host erythrocyte | 873.151 | Integral component of membrane | 165.105 |
| Q9K2J6 | Oxidoreductase | 67.7891 | Regulation of transcription | 14167.9 | Integral component of membrane | 238.524 |
| Q9KS64 | Lamin binding | 666.243 | Viral process | 121.397 | Host cell nuclear lamina | 154.877 |
| Q9KN40 | GPCR binding | 96.19 | Sphingolipid metabolism | 83.606 | Integral component of membrane | 173.532 |
| Q9KVW5 | Amino-acyl tRNA ligase | 819.927 | Late endosome to vacuole transport via multivesicular body sorting pathway | 371.518 | Integral component of membrane | 396.436 |
| Q9KL81 | Unknown | NA | Unknown | NA | Unknown | NA |
| Q9KPA0 | Kinase | 135.732 | Phosphorylation | 1232.71 | Integral component of membrane | 171.24 |
| Q9KL73 | Unknown | NA | Unknown | NA | Unknown | NA |
| Q9KNG0 | DNA binding | 11.8076 | DNA biosynthetic process | 9.5782 | Unknown |  |
| Q9KSJ4 | Methyltransferase | 371.924 | Methylation | 2568.06 | Integral component of membrane | 183.591 |
| Q9KPZ1 | Phosphorelay sensor kinase | 40.4372 | Phosphorylation | 85.6623 | Integral component of membrane | 1341.77 |
| Q9KNI6 | Unknown | NA | Unknown | NA | Unknown | NA |
| Q9KVT0 | Unknown | NA | Unknown | NA | Unknown | NA |
| Q9KST0 | Unknown | NA | Tryptophan biosynthesis | 1591.81 | Unknown | NA |
