# Supplementary material for: Comprehensive in silico analyses of fifty-one uncharacterized proteins from Vibrio cholerae
Source: PLoS One. 2024 Oct 4;19(10):e0311301. doi: 10.1371/journal.pone.0311301 (PMC11452002; doi:10.1371/journal.pone.0311301)
Supplement: S9 Table — (DOCX) [file pone.0311301.s009.docx]

**Table S9**

**Protein-Protein Interaction:** Identification of interacting protein partners of candidate uncharacterized protein by STRING

| **UniProt ID** | **Gene name** | **Interacting Partners** | | **Scores** |
| --- | --- | --- | --- | --- |
| Q9KRD2 | VC_1710 | VC_2370 | Sensory box | 0.993 |
|  |  | VC_0303 | Sensory Histidine Kinase | 0.985 |
|  |  | VC_1349 | Sensor histidine kinase | 0.985 |
|  |  | VC_2369 | Sensor histidine kinase FexB | 0.985 |
|  |  | VC_1653 | Sensory box histididne kinase VieS | 0.978 |
|  |  | VC_2453 | Sensory box sensor histidine kinase | 0.978 |
|  |  | VC_A0709 | Sensor protein tors | 0.978 |
|  |  | VC_A0785 | GGDEF family protein | 0.975 |
|  |  | VC_A0080 | GGDEF family protein | 0.970 |
|  |  | VC_1376 | GGDEF family protein | 0.968 |
| Q9KVG3 | VC_0183 | VC_0182 | Uncharacterized protein | 0.866 |
|  |  | VC_0185 | Transposase | 0.837 |
|  |  | VC_0184 | Uncharacterized protein | 0.799 |
|  |  | VC_0186 | Glutathione reductase | 0.600 |
|  |  | VC_1531 | Hypothetical protein | 0.508 |
|  |  | RtxA | RTX toxin RtxA | 0.482 |
|  |  | VC_2032 | Hypothetical protein | 0.461 |
|  |  | VC_A0422 | Conserved hypothetical protein | 0.443 |
|  |  | VC_0176 | Trsnacriptional regulator (putative) | 0.428 |
|  |  | VC_0702 | Conserved hypothetical protein | 0.426 |
| Q9KT38 | VC_1067 | VC_A0785 | GGDEF Family protein | 0.961 |
|  |  | VC_1086 | Response regulator | 0.958 |
|  |  | VC_1710 | Conserved hypothetical protein | 0.956 |
|  |  | VC_0303 | Sensor Histidine kinase | 0.944 |
|  |  | VC_2453 | Sensor Histidine Kinase | 0.933 |
|  |  | VC_1349 | Sensoy box sensor histidine kinase | 0.931 |
|  |  | VC_2369 | Sensor histidine kinase FexB | 0.931 |
|  |  | VC_1653 | Sensoy box sensor histidine kinase | 0.914 |
|  |  | VC_0072 | Sensory box?GGDEF family protein | 0.909 |
|  |  | VC_0653 | c-di-GMP phosphodiesterase A-related protein | 0.899 |
| Q9KKL8 | VC_A0185 | VC_1885 | Hypothetical protein | 0.712 |
|  |  | VC_0377 | Conserved hypothetical protein | 0.704 |
|  |  | VC_1909 | Hypothetical protein | 0.699 |
|  |  | VC_2344 | Hypothetical protein | 0.693 |
|  |  | VC_1997 | Hypothetical protein | 0.693 |
|  |  | VC_2557 | Hypothetical protein | 0.665 |
|  |  | VC_2072 | peptidase | 0.634 |
|  |  | VC_1271 | Hypothetical protein | 0.632 |
|  |  | VC_2609 | Hypothetical protein | 0.632 |
|  |  | VC_1044 | Hypothetical protein | 0.627 |
| Q9KQX3 | VC_1874 | VC_1873 | Conserved hypothetical protein | 0.996 |
|  |  | VC_1872 | Uncharacterized protein | 0.994 |
|  |  | uspB | Universal stress protein B homolog | 0.582 |
|  |  | VC_2621 | Extracellular nuclease-related protein | 0.561 |
|  |  | VC_2507 | Conserved hypothetical protein | 0.477 |
|  |  | napD | napD protein, Chaperone for napA | 0.457 |
|  |  | VC_2166 | Uncharacterized protein | 0.450 |
|  |  | blc | Lipoprotein blc | 0.444 |
|  |  | VC_A0350 | Lipoprotein blc | 0.444 |
|  |  | VC_A0406 | Lipoprotein blc | 0.444 |
| Q9KLK5 | VC_A0738 | VC_A0583 | Uncharacterized protein | 0.887 |
|  |  | VC_2298 | Lipoprotein, putative | 0.869 |
|  |  | VC_A0739 | Conserved hypothetical protein | 0.794 |
|  |  | VC_A0148 | TagA-related protein | 0.791 |
|  |  | VC_A0740 | Conserved hypothetical protein | 0.763 |
|  |  | VC_A0059 | Major outer membrane lipoprotein | 0.740 |
|  |  | VC_1952 | Chitinase | 0.693 |
|  |  | nanH | Neuraminidase | 0.589 |
|  |  | luxP | Luxp protein | 0.587 |
|  |  | VC_A0027 | Chitinase | 0.559 |
| Q9KT24 | VC_1081 | VC_1085 | Sensor histidine kinase | 0.963 |
|  |  | VC_1086 | Response regulator | 0.937 |
|  |  | VC_1082 | Response regulator | 0.932 |
|  |  | VC_1084 | Sensory box sensor histidine kinase | 0.931 |
|  |  | VC_1080 | Uncharacterized protein | 0.911 |
|  |  | VC_0622 | Sensory box sensor histidine kinase | 0.900 |
|  |  | VC_1349 | Sensory box sensor histidine kinase | 0.879 |
|  |  | VC_A0709 | Sensory box sensor histidine kinase | 0.872 |
|  |  | VC_A1086 | Response regulator | 0.840 |
|  |  | VC_1087 | Response regulator | 0.835 |
| Q9KMS2 | VC_A0248 | VC_A0246 | SgaT protein | 0.994 |
|  |  | VC_A0245 | PTS system | 0.989 |
|  |  | VC_A0242 | Hexulose-6-phosphate synthase SgbH | 0.977 |
|  |  | VC_A0247 | Transcriptional regulator | 0.937 |
|  |  | VC_A0241 | Hexulose-6-phosphate isomerase SgbU | 0.893 |
|  |  | VC_A0244 | Sugar isomerase SgaE | 0.798 |
|  |  | rpsF | Ribosomal protein s6 | 0.783 |
|  |  | nfuA | Conserved hypothetical protein | 0.722 |
|  |  | VC_0486 | Transcriptional regulator | 0.538 |
|  |  | VC_A0243 | Uncharacterized protein | 0.497 |
| Q9KMV6 | VC_A0212 | VC_A0211 | Sensory box sensor histidine kinase | 0.869 |
|  |  | VC_A0583 | Uncharacterized proteins | 0.727 |
|  |  | VC_1603 | Uncharacterized proteins | 0.710 |
|  |  | VC_A0031 | Uncharacterized proteins | 0.679 |
|  |  | VC_A0981 | Uncharacterized proteins | 0.666 |
|  |  | VC_1601 | Uncharacterized proteins | 0.653 |
|  |  | VC_2622 | Uncharacterized proteins | 0.616 |
|  |  | VC_2640 | Uncharacterized proteins | 0.598 |
|  |  | VC_2038 | Uncharacterized proteins | 0.575 |
|  |  | VC_0930 | Haemolysin-related protein | 0.571 |
| Q9KRM9 | VC_1607 | VC_1609 | Uncharacterized protein | 0.918 |
|  |  | VC_1608 | Uncharacterized protein | 0.978 |
|  |  | VC_1606 | Uncharacterized protein | 0.973 |
|  |  | VC_1409 | Multidrug resistance protein | 0.426 |
| Q9KU75 | VC_0648 | pnp | Polyribonucleotide nucleotidyltransferase | 0.867 |
|  |  | proQ | Conserved hypothetical protein | 0.824 |
|  |  | VC_1496 | Tail-specific protease | 0.817 |
|  |  | metJ | Met repressor | 0.763 |
|  |  | VC_1099 | UPF0208 membrane protein VC_1099 | 0.762 |
|  |  | seqA | Seqa protein | 0.759 |
|  |  | rraB | Conserved hypothetical protein | 0.737 |
|  |  | VC_2466 | Sigma-e factor negative regulatory protein rsea | 0.730 |
|  |  | VC_1433 | Uncharacterized protein | 0.729 |
|  |  | yihI | Conserved hypothetical protein | 0.713 |
| Q9KND1 | VC_A0034 | rplT | Ribosomal protein l20 | 0.692 |
|  |  | VC_A0212 | Uncharacterized protein | 0.679 |
|  |  | VC_1349 | Sensory box sensor histidine kinase | 0.654 |
|  |  | VC_A0035 | Phosphatidylglycerophosphatase B | 0.589 |
|  |  | VC_1348 | Response regulator | 0.567 |
|  |  | VC_A0211 | Sensory box sensor histidine kinase | 0.562 |
|  |  | VC_2127 | Flagellar protein flil | 0.541 |
|  |  | VC_A0447 | Haemagglutinin associated protein | 0.534 |
|  |  | rpoB | Dna-directed rna polymerase | 0.530 |
|  |  | VC_A0210 | Response regulator, putative | 0.518 |
| Q9KTC9 | VC_0973 | VC_0972 | Porin | 0.668 |
|  |  | cueR | Transcriptional regulator | 0.654 |
|  |  | VC_a0530 | Pyruvate-flavoredoxin oxidoreductase | 0.652 |
|  |  | tatC | Conserved hypothetical protein | 0.452 |
|  |  | VC_A0033 | Uncharacterized protein | 0.452 |
|  |  | VC_0727 | Phosphate transport system regulatory protein phou | 0.402 |
| Q9KSQ9 | VC_1197 | VC_1538 | Uncharacterized protein | 0.711 |
|  |  | cheD | Chemotaxis protein ched | 0.631 |
|  |  | VC_0377 | Uncharacterized protein | 0.588 |
|  |  | VC_1198 | Uncharacterized protein | 0.553 |
|  |  | VC_A1030 | Uncharacterized protein | 0.544 |
|  |  | VC_1196 | Uncharacterized protein | 0.540 |
|  |  | VC_1199 | Uncharacterized protein | 0.536 |
|  |  | VC_1397 | hemotaxis protein CheA | 0.515 |
|  |  | VC_2063 | hemotaxis protein CheA | 0.515 |
|  |  | VC_A1095 | hemotaxis protein CheA | 0.515 |
| Q9KS60 | VC­_1400 | VC_A0530 | Pyruvate-flavoredoxin oxidoreductase | 0.974 |
|  |  | VC_1402 | Purine-binding chemotaxis protein | 0.955 |
|  |  | VC_1403 | Methyl-accepting chemotaxis protein | 0.952 |
|  |  | VC_1398 | Chemotaxis protein CheY | 0.919 |
|  |  | VC_1397 | Chemotaxis protein CheA | 0.905 |
|  |  | cheB3 | Protein-glutamate methylesterase cheb | 0.899 |
|  |  | cheR2 | Chemotaxis protein methyltransferase cher | 0.886 |
|  |  | VC_1396 | Uncharacterized protein | 0.854 |
|  |  | pyrG | Ctp synthase | 0.820 |
|  |  | VC_0809 | Uncharacterized protein | 0.793 |
| Q9KKX0 | VC_A0980 | VC_2063 | Chemotaxis protein CheA | 0.898 |
|  |  | VC_A0979 | Methyl-accepting chemotaxis protein | 0.830 |
|  |  | VC_1397 | Chemotaxis protein CheA | 0.816 |
|  |  | VC_A1095 | Chemotaxis protein CheA | 0.816 |
|  |  | VC_2059 | Purine-binding chemotaxis protein CheW | 0.790 |
|  |  | VC_2060 | Uncharacterized protein | 0.782 |
|  |  | VC_A0709 | Sensor protein tors; Histidine kinase | 0.782 |
|  |  | VC_A0978 | Amino acid ABC transporter | 0.778 |
|  |  | VC_A1093 | Purine-binding chemotaxis protein CheW | 0.757 |
|  |  | VC_A1094 | Purine-binding chemotaxis protein CheW | 0.757 |
| Q9KND9 | VC_A0026 | VC_0174 | Uncharacterized protein | 0.781 |
|  |  | VC_2168 | Uncharacterized protein | 0.701 |
|  |  | VC_1384 | Uncharacterized protein | 0.698 |
|  |  | VC_1154 | Uncharacterized protein | 0.696 |
|  |  | VC_2739 | Uncharacterized protein | 0.677 |
|  |  | VC_1887 | Uncharacterized protein | 0.670 |
|  |  | VC_A0035 | Phosphatidylglycerophosphatase B | 0.659 |
|  |  | VC_2298 | Lipoprotein | 0.658 |
|  |  | VC_A0530 | Pyruvate-flavoredoxin oxidoreductase | 0.652 |
|  |  | VC_1894 | Uncharacterized protein | 0.650 |
| Q9KRJ5 | VC_1645 | VC_2271 | Riboflavin-specific deaminase | 0.891 |
|  |  | VC_1594 | Aldose 1-epimerase | 0.725 |
|  |  | guaA | Gmp synthase | 0.709 |
|  |  | VC_A0013 | Maltodextrin phosphorylase | 0.671 |
|  |  | VC_2095 | Phosphoglucomutase | 0.670 |
|  |  | VC_0295 | UTP--glucose-1-phosphate uridylyltransferase | 0.667 |
|  |  | VC_A0014 | 4-alpha-glucanotransferase | 0.666 |
|  |  | GuaB | inosine-5`-monophosphate dehydrogenase | 0.660 |
| Q9KVJ9 | VC_0144 | VC_A0695 | Uncharacterized protein | 0.778 |
|  |  | VC_A0042 | Hypothetical protein | 0.769 |
|  |  | VC_1600 | Uncharacterized protein | 0.764 |
|  |  | VC_A0051 | Uncharacterized protein | 0.764 |
|  |  | VC_A0571 | Uncharacterized protein | 0.764 |
|  |  | VC_1380 | Uncharacterized protein | 0.761 |
|  |  | VC_2212 | Uncharacterized protein | 0.761 |
|  |  | VC_2717 | Uncharacterized protein | 0.761 |
|  |  | VC_2667 | Uncharacterized protein | 0.752 |
|  |  | VC_A0050 | Uncharacterized protein | 0.752 |
| Q9KSV3 | VC_1153 | VC_1495 | Uncharacterized protein | 0.774 |
|  |  | VC_A0052 | Uncharacterized protein | 0.772 |
|  |  | VC_1152 | Uncharacterized protein | 0.771 |
|  |  | VC_1832 | Hypothetical protein | 0.769 |
|  |  | VC_A0652 | Uncharacterized protein | 0.767 |
|  |  | VC_A0002 | Uncharacterized protein | 0.765 |
|  |  | VC_A0567 | Uncharacterized protein | 0.763 |
|  |  | VC_A1114 | Parb family proteins | 0.762 |
|  |  | VC_1899 | Uncharacterized protein | 0.761 |
|  |  | VC_1272 | Uncharacterized protein | 0.760 |
| Q9KSV6 | VC_1150 | VC_0882 | Uncharacterized protein | 0.783 |
|  |  | VC_0142 | Uncharacterized protein | 0.781 |
|  |  | VC_1431 | Uncharacterized protein | 0.781 |
|  |  | VC_A0039 | Uncharacterized protein | 0.781 |
|  |  | VC_0140 | Uncharacterized protein | 0.779 |
|  |  | toxS | Regulatory protein toxs | 0.773 |
|  |  | VC_A0261 | Uncharacterized protein | 0.766 |
|  |  | VC_1154 | Uncharacterized protein | 0.754 |
|  |  | VC_2609 | Uncharacterized protein | 0.752 |
|  |  | VC_2154 | Uncharacterized protein | 0.749 |
| Q9KND3 | VC_A0032 | VC_A0031 | Methyl-accepting chemotaxis protein | 0.723 |
|  |  | VC_1652 | Response regulator VieA | 0.558 |
|  |  | VC_2453 | Sensor histidine kinase/response regulator | 0.487 |
|  |  | VC_0008 | Amino acid ABC transporter | 0.475 |
|  |  | VC_1359 | Amino acid ABC transporter | 0.475 |
|  |  | VC_2369 | Sensor histidine kinase fexb | 0.475 |
|  |  | VC_A0709 | Sensor protein tors | 0.475 |
|  |  | VC_A0760 | Arginine ABC transporter | 0.475 |
|  |  | VC_A1037 | Amino acid ABC transporter | 0.475 |
|  |  | VC_1406 | Methyl-accepting chemotaxis protein | 0.454 |
| Q9KP29 | VC_2550 | msrA | Peptide methionine sulfoxide reductase | 0.604 |
|  |  | mukF | Mukf protein | 0.546 |
|  |  | mukB | Cell division protein mukb | 0.500 |
|  |  | mukE | Muke protein | 0.493 |
|  |  | uspB | Universal stress protein B homolog | 0.474 |
|  |  | VC_2551 | Uncharacterized protein | 0.456 |
|  |  | VC_2146 | Uncharacterized protein | 0.452 |
|  |  | matP | Conserved hypothetical protein | 0.449 |
|  |  | VC_1208 | Conserved hypothetical protein | 0.426 |
|  |  | VC_2547 | Uncharacterized protein | 0.423 |
| Q9KMX1 | VC_A0195 | VC_A0194 | Potassium channel protein | 0.643 |
|  |  | VC_A0196 | Uncharacterized protein | 0.552 |
|  |  | skp | Outer membrane protein omph | 0.453 |
| Q9KTE5 | VC_0957 | VC_2540 | Uncharacterized protein | 0.920 |
|  |  | VC_A1044 | Uncharacterized protein | 0.749 |
|  |  | leuS | Leucyl-trna synthetase | 0.653 |
|  |  | VC_1539 | Conserved hypothetical protein | 0.631 |
|  |  | rpsQ | Ribosomal protein s17 | 0.554 |
|  |  | rpsH | Ribosomal protein s8 | 0.552 |
|  |  | rpsE | Ribosomal protein s5 | 0.551 |
|  |  | rpsL | Ribosomal protein s12 | 0.548 |
|  |  | rpsO | Ribosomal protein s15 | 0.548 |
|  |  | rpsB | Belongs to the universal ribosomal protein uS2 family | 0.548 |
| Q9KPD6 | VC_2434 | VC_2435 | MutT/nudix family protein | 0.964 |
|  |  | cpdA | Cyclic amp phosphodiesterase | 0.873 |
|  |  | VC_2432 | Uncharacterized protein | 0.834 |
|  |  | VC_0330 | Regulator of sigma d | 0.676 |
|  |  | tolC | Outer membrane protein tolc | 0.572 |
|  |  | VC_0957 | Uncharacterized protein | 0.501 |
|  |  | VC_0351 | Uncharacterized protein | 0.455 |
|  |  | pare | Topoisomerase iv | 0.439 |
|  |  | VC_A0987 | Phosphoenolpyruvate synthase | 0.439 |
|  |  | zipA | Cell division protein zipa | 0.438 |
| Q9KPA3 | VC_2470 | sdhE | Conserved hypothetical protein | 0.845 |
|  |  | VC_2472 | Conserved hypothetical protein | 0.567 |
| Q9KNF4 | VC_A0010 | VC_A0009 | Uncharacterized protein | 0.845 |
|  |  | VC_2073 | Uncharacterized protein | 0.721 |
|  |  | VC_A0579 | Uncharacterized protein | 0.714 |
|  |  | VC_0370 | Uncharacterized protein | 0.664 |
|  |  | VC_A0008 | Methyl-accepting chemotaxis protein | 0.629 |
|  |  | trpR | Trp operon repressor, putative | 0.587 |
|  |  | VC_1224 | Uncharacterized protein | 0.580 |
|  |  | VC_0197 | Gene 3 protein-related protein | 0.572 |
|  |  | VC_A0059 | Major outer membrane lipoprotein | 0.563 |
|  |  | VC_A0582 | Uncharacterized protein | 0.563 |
| Q9KT53 | VC_1052 | VC_1051 | Uncharacterized protein | 0.854 |
|  |  | mobA | Molybdopterin-guanine dinucleotide biosynthesis protein a | 0.798 |
|  |  | trpR | Trp operon repressor, putative | 0.687 |
|  |  | apt | Adenine phosphoribosyltransferase | 0.605 |
|  |  | edd | Phosphogluconate dehydratase | 0.552 |
|  |  | VC_0285 | 4-hydroxy-2-oxoglutarate aldolase | 0.551 |
|  |  | viuB | Vibriobactin utilization protein viub | 0.530 |
|  |  | dnaX | Dna polymerase iii | 0.512 |
|  |  | VC_1050 | Response regulator | 0.491 |
|  |  | VC_1055 | Conserved hypothetical protein | 0.443 |
| Q9KL56 | VC_A0892 | VC_A0893 | Uncharacterized protein | 0.566 |
|  |  | VC_A0894 | Coserved hypothetical protein | 0.566 |
|  |  | VC_A0891 | Uncharacterized protein | 0.566 |
| Q9KRE6 | VC_1696 | VC_1697 | Uncharacterized protein | 0.575 |
|  |  | VC_1698 | Uncharacterized protein | 0.574 |
|  |  | VC_1695 | Formate transporter 1 | 0.522 |
|  |  | VC_1906 | Uncharacterized protein | 0.502 |
|  |  | VC_A0169 | Uncharacterized protein | 0.483 |
|  |  | VC_2555 | Uncharacterized protein | 0.458 |
|  |  | VC_A0314 | Uncharacterized protein | 0.454 |
|  |  | VC_0122 | Adenylate cyclase | 0.449 |
|  |  | VC_1499 | ABC transporter | 0.420 |
| Q9KLX2 | VC_A0619 | VC_A0620 | Thiosulfate sulfurtransferase SseA | 0.821 |
|  |  | VC_A0530 | Pyruvate-flavoredoxin oxidoreductase | 0.783 |
|  |  | VC_A0618 | Molybdopterin biosynthesis MoeB protein | 0.504 |
|  |  | VC_A0617 | Molybdopterin biosynthesis moea protein | 0.479 |
|  |  | VC_1644 | Uncharacterized protein | 0.475 |
|  |  | VolA | Lipase | 0.457 |
|  |  | FolE | GTP cyclohydrolase 1 | 0.438 |
|  |  | GuaA | Gmp synthase | 0.436 |
|  |  | vcc | Collagenase | 0.434 |
|  |  | VC_0026 | Zinc-binding alcohol dehydrogenase | 0.413 |
| Q9KLQ3 | VC_A0689 | VC_A0688 | Polyhydroxyalkanoic acid synthase | 0.913 |
|  |  | VC_A0691 | Acetoacetyl-CoA reductase | 0.856 |
|  |  | VC_A0690 | Acetyl-coa acetyltransferase | 0.761 |
|  |  | VC_0220 | Uncharacterized protein | 0.600 |
|  |  | VC_2207 | Uncharacterized protein | 0.592 |
|  |  | VC_2208 | Uncharacterized protein | 0.592 |
|  |  | VC_2221 | Uncharacterized protein | 0.551 |
|  |  | VC_1965 | Uncharacterized protein | 0.543 |
|  |  | VC_2232 | Uncharacterized protein | 0.536 |
|  |  | VC_2667 | Uncharacterized protein | 0.524 |
| Q9KKS6 | VC_A1024 | VC_1271 | Uncharacterized protein | 0.647 |
|  |  | VC_A0042 | Hypothetical protein | 0.640 |
|  |  | VC_A1087 | Anti-sigma F factor antagonist | 0.583 |
|  |  | VC_A0030 | Uncharacterized protein | 0.580 |
|  |  | VC_A1021 | Uncharacterized protein | 0.562 |
|  |  | VC_1997 | Uncharacterized protein | 0.550 |
|  |  | VC_A1086 | Response regulator | 0.540 |
|  |  | VC_1157 | Response regulator | 0.534 |
|  |  | VC_0144 | Uncharacterized protein | 0.499 |
|  |  | VC_1870 | Uncharacterized protein | 0.473 |
| Q9KN87 | VC_A0078 | VC_A0013 | Maltodextrin phosphorylase | 0.858 |
|  |  | VC_A1029 | Glycogen operon protein glgx | 0.839 |
|  |  | nnr | Conserved hypothetical protein | 0.775 |
|  |  | VC_2095 | Phosphoglucomutase | 0.770 |
|  |  | glgA | Glycogen synthase | 0.665 |
|  |  | VC_0245 | RfbG protein | 0.637 |
|  |  | VC_0259 | Lipopolysaccharide biosynthesis protein RfbV | 0.637 |
|  |  | VC_0920 | Exopolysaccharide biosynthesis protein EpsF | 0.637 |
|  |  | VC_0925 | Polysaccharide biosynthesis protein | 0.637 |
|  |  | VC_A0712 | Pyrazinamidase/nicotinamidase | 0.622 |
| Q9KU58 | VC_0666 | VC_0023 | NADH dehydrogenase subunit II-related protein | 0.756 |
|  |  | VC_1637 | Uncharacterized protein | 0.741 |
|  |  | VC_2344 | Uncharacterized protein | 0.739 |
|  |  | VC_A0597 | Uncharacterized protein | 0.738 |
|  |  | VC_2314 | Uncharacterized protein | 0.735 |
|  |  | VC_1342 | MutT/nudix family protein | 0.694 |
|  |  | VC_2139 | Flagellar rod protein FlaI | 0.693 |
|  |  | VC_0859 | Uncharacterized protein | 0.686 |
|  |  | VC_1193 | Uncharacterized protein | 0.681 |
|  |  | VC_A1065 | Uncharacterized protein | 0.681 |
| Q9KPP0 | VC_2326 | recN | Dna repair protein recn | 0.660 |
|  |  | VC_A0801 | Inosine-guanosine kinase | 0.624 |
|  |  | VC_1129 | Inosine-guanosine kinase | 0.622 |
|  |  | recA | Reca protein | 0.617 |
|  |  | VC_2324 | Transcriptional regulator | 0.549 |
|  |  | lexA | Lexa repressor | 0.483 |
|  |  | astE | Succinylglutamate desuccinylase | 0.434 |
|  |  | VC_A0608 | Uncharacterized protein | 0.409 |
|  |  | VC_2323 | Uncharacterized protein | 0.401 |
| B1B1N2 | VC_A0594 | VC_A0593 | Uncharacterized protein | 0.517 |
|  |  | VC_2365 | Uncharacterized protein | 0.481 |
|  |  | VC_A0592 | MutT/nudix family protein | 0.424 |
| Q9K2J6 | VC_A0319 | AAF96226.1 | Conserved hypothetical protein | 0.974 |
|  |  | VC_A0481 | Uncharacterized protein | 0.841 |
|  |  | VC_A0487 | Uncharacterized protein | 0.823 |
|  |  | VC_A0482 | Uncharacterized protein | 0.802 |
|  |  | AAF96228.1 | Hypothetical protein | 0.452 |
|  |  | VC_1949 | PvcA protein | 0.432 |
| Q9KS64 | VC_1396 | VC_1398 | Chemotaxis protein CheY | 0.878 |
|  |  | VC_1400 | Uncharacterized protein | 0.854 |
|  |  | VC_1397 | Chemotaxis protein CheA | 0.847 |
|  |  | cheR2 | Chemotaxis protein methyltransferase cher | 0.845 |
|  |  | cheB3 | Protein-glutamate methylesterase cheb | 0.842 |
|  |  | Vc_1402 | Purine-binding chemotaxis protein Chew | 0.793 |
|  |  | VC_1403 | Methyl-accepting chemotaxis protein | 0.778 |
|  |  | VC_1404 | Uncharacterized protein | 0.491 |
|  |  | VC_1394 | Methyl-accepting chemotaxis protein | 0.406 |
| Q9KN40 | VC_A0125 | rbsD | Ribose abc transporter protein | 0.777 |
|  |  | VC_A0175 | MoxR-related protein | 0.724 |
|  |  | VC_A0176 | Methyl-accepting chemotaxis protein | 0.724 |
|  |  | VC_2098 | Basal-body rod modification protein flgd; | 0.691 |
|  |  | VC_A0022 | Glutathione S-transfersae-related protein | 0.689 |
|  |  | VC_2090 | Succinate dehydrogenase | 0.683 |
|  |  | VC_A0126 | Uncharacterized protein | 0.629 |
|  |  | VC_1661 | Uncharacterized protein | 0.616 |
|  |  | vcc | Collagenase | 0.593 |
|  |  | VC_1035 | Uncharacterized protein | 0.581 |
| Q9KVW5 | VC_0023 | VC_2717 | Uncharacterized protein | 0.777 |
|  |  | VC_A0571 | Uncharacterized protein | 0.777 |
|  |  | VC_1988 | Uncharacterized protein | 0.774 |
|  |  | VC_A0052 | Uncharacterized protein | 0.773 |
|  |  | VC_A0920 | Uncharacterized protein | 0.773 |
|  |  | VC_0861 | Type IV pilin | 0.762 |
|  |  | LuxU | Phosphorelay protein | 0.761 |
|  |  | VC_2344 | Uncharacterized protein | 0.759 |
|  |  | VC_0425 | Uncharacterized protein | 0.758 |
|  |  | VC_2667 | Uncharacterized protein | 0.758 |
| Q9KL81 | VC_A0866 | hap | Hemagglutinin/protease | 0.629 |
| Q9KPA0 | VC_2473 | VC_2276 | Conserved hypothetical protein | 0.768 |
|  |  | zapC | Conserved hypothetical protein | 0.732 |
|  |  | VC_A0544 | Uncharacterized protein | 0.718 |
|  |  | VC_A0762 | Uncharacterized protein | 0.707 |
|  |  | bamC | ipoprotein-34 NlpB | 0.683 |
|  |  | VC_2264 | Conserved hypothetical protein | 0.683 |
|  |  | Crl | Transcriptional regulator crl | 0.667 |
|  |  | UspB | Universal stress protein B homolog | 0.660 |
|  |  | VC_0153 | Uncharacterized protein | 0.657 |
|  |  | VC_2112 | Uncharacterized protein | 0.653 |
| Q9KL73 | VC_A0874 |  | |  |
| Q9KNG0 | VC_A0004 | VC_A0005 | Uncharacterized protein | 0.887 |
|  |  | VC_0901 | Uncharacterized protein | 0.774 |
|  |  | VC_A0547 | Uncharacterized protein | 0.773 |
|  |  | VC_A0567 | Uncharacterized protein | 0.771 |
|  |  | VC_A0799 | Uncharacterized protein | 0.759 |
|  |  | VC_2286 | Uncharacterized protein | 0.754 |
|  |  | VC_A0015 | Hypothetical protein | 0.746 |
|  |  | VC_A0652 | Uncharacterized protein | 0.743 |
|  |  | VC_1158 | Uncharacterized protein | 0.742 |
|  |  | VC_2346 | Smp protein | 0.739 |
| Q9KSJ4 | VC_1262 | ribA | Gtp cyclohydrolase ii | 0.490 |
|  |  | VC_1261 | Uncharacterized protein | 0.480 |
| Q9KPZ1 | VC_2221 | VC_1480 | Uncharacterized protein | 0.701 |
|  |  | VC_A0023 | Uncharacterized protein | 0.692 |
|  |  | VC_0871 | Uncharacterized protein | 0.686 |
|  |  | VC_1076 | Transcriptional regulator | 0.619 |
|  |  | VC_2222 | Uncharacterized protein | 0.581 |
|  |  | VC_A0689 | Uncharacterized protein | 0.551 |
|  |  | VC_A0919 | Uncharacterized protein | 0.545 |
|  |  | VC_A0186 | Uncharacterized protein | 0.495 |
|  |  | VC_2154 | Uncharacterized protein | 0.488 |
|  |  | VC_1009 | Uncharacterized protein | 0.485 |
| Q9KNI6 | VC_2753 | VC_2752 | Uncharacterized protein | 0.831 |
|  |  | VC_2754 | Uncharacterized protein | 0.765 |
|  |  | VC_2755 | Protoporphyrinogen oxidase | 0.765 |
|  |  | VC_2756 | Potassium uptake protein trkh | 0.710 |
|  |  | VC_2757 | Uncharacterized protein | 0.670 |
|  |  | fadB | Fatty oxidation complex | 0.422 |
| Q9KVT0 | VC_0059 | VC_0940 | Uncharacterized protein | 0.683 |
|  |  | VC_A1063 | Ornithine decarboxylase | 0.683 |
|  |  | purC | Phosphoribosylaminoimidazole-succinocarboxamide synthase | 0.559 |
|  |  | VC_0058 | Carbonic anhydrase | 0.541 |
|  |  | VC_A0862 | Long-chain fatty acid transport protein | 0.535 |
| Q9KST0 | VC_1176 | VC_1175 | Uncharacterized protein | 0.675 |
|  |  | trpCF | indole-3-glycerol phosphate synthase | 0.674 |
|  |  | trpD | Anthranilate phosphoribosyltransferase | 0.674 |
|  |  | trpG | Anthranilate synthase component ii | 0.674 |
|  |  | trpE | Anthranilate synthase component i | 0.674 |
|  |  | trpA | Tryptophan synthase, alpha subunit | 0.566 |
|  |  | trpB | Tryptophan synthase, beta subunit | 0.566 |
|  |  | VC_1177 | Uncharacterized protein; subunit B | 0.566 |
|  |  | VC_1178 | Conserved hypothetical protein | 0.566 |
