# Supplementary material for: Comprehensive in silico analyses of fifty-one uncharacterized proteins from Vibrio cholerae
Source: PLoS One. 2024 Oct 4;19(10):e0311301. doi: 10.1371/journal.pone.0311301 (PMC11452002; doi:10.1371/journal.pone.0311301)
Supplement: S10 Table — (DOCX) [file pone.0311301.s010.docx]

**Table S10: -**

**Antigenicity, allergenicity and toxicity of candidate uncharacterized proteins**

| **UniProt ID** | **Gene name** | **^ψ^Overall prediction for the protective antigen** | **Conclusion** | **Allergenicity** | **Toxicity** | **^∆^Hybrid score** |
| --- | --- | --- | --- | --- | --- | --- |
| Q9KRD2 | VC_1710 | 0.4098 | Antigen | Non-allergen | Non-toxin | -0.28 |
| Q9KVG3 | VC_0183 | 0.4617 | Antigen | Non-allergen | Non-toxin | 0.26 |
| Q9KT38 | VC_1067 | 0.4389 | Antigen | Non-allergen | Non-Toxin | -0.32 |
| Q9KKL8 | VC_A0185 | 0.5392 | Antigen | Non-allergen | Non-Toxin | 0.29 |
| Q9KQX3 | VC_1874 | 0.3451 | Non-antigen | Non-allergen | Non-toxin | 0.2 |
| Q9KLK5 | VC_A0738 | 0.7235 | Antigen | Non-allergen | Non-toxin | 0.46 |
| Q9KT24 | VC_1081 | 0.3057 | Non-antigen | Non-allergen | Non-toxin | 0.18 |
| Q9KMS2 | VC_A0248 | 0.4801 | Antigen | Allergen | Non-toxin | 0.3 |
| Q9KMV6 | VC_A0212 | 0.3592 | Non-antigen | Non-allergen | Non-toxin | 0.27 |
| Q9KRM9 | VC_1607 | 0.6390 | Non-antigen | Non-allergen | Non-toxin | 0.31 |
| Q9KU75 | VC_0648 | 0.4544 | Antigen | Non-allergen | Non-toxin | 0.22 |
| Q9KND1 | VC_A0034 | 0.3168 | Non-antigen | Non-allergen | Non-toxin | 0.25 |
| Q9KTC9 | VC_0973 | 0.3874 | Non-antigen | Non-allergen | Non-toxin | 0.32 |
| Q9KSQ9 | VC_1197 | 0.3264 | Non-antigen | Non-allergen | Non-toxin | 0.23 |
| Q9KS60 | VC­_1400 | 0.3783 | Non-antigen | Non-allergen | Non-toxin | 0.18 |
| Q9KKX0 | VC_A0980 | 0.7476 | Antigen | Allergen | Non-toxin | 0.23 |
| Q9KND9 | VC_A0026 | 0.6600 | Antigen | Non-allergen | Non-toxin | 0.29 |
| Q9KRJ5 | VC_1645 | 0.2985 | Non-antigen | Non-allergen | Non-toxin | 0.2 |
| Q9KVJ9 | VC_0144 | 0.4486 | Antigen | Non-allergen | Non-toxin | 0.22 |
| Q9KSV3 | VC_1153 | 0.3211 | Non-antigen | Allergen | Toxin | 0.68 |
| Q9KSV6 | VC_1150 | 0.4595 | Antigen | Non-allergen | Non-toxin | 0.22 |
| Q9KND3 | VC_A0032 | 0.6382 | Antigen | Non-allergen | Non-toxin | 0.3 |
| Q9KP29 | VC_2550 | 0.3707 | Non-antigen | Non-allergen | Non-toxin | 0.26 |
| Q9KMX1 | VC_A0195 | 0.6380 | Antigen | Allergen | Non-toxin | 0.28 |
| Q9KTE5 | VC_0957 | 0.2614 | Non-antigen | Non-allergen | Non-toxin | 0.24 |
| Q9KPD6 | VC_2434 | 0.3921 | Non-antigen | Non-allergen | Non-toxin | 0.23 |
| Q9KPA3 | VC_2470 | 0.8699 | Antigen | Non-allergen | Non-toxin | 0.24 |
| Q9KNF4 | VC_A0010 | 0.3949 | Non-antigen | Non-allergen | Non-toxin | 0.21 |
| Q9KT53 | VC_1052 | 0.9049 | Antigen | Non-allergen | Non-toxin | 0.17 |
| Q9KL56 | VC_A0892 | 0.6174 | Antigen | Allergen | Non-toxin | 0.18 |
| Q9KRE6 | VC_1696 | 0.4427 | Antigen | Non-allergen | Non-toxin | 0.3 |
| Q9KLX2 | VC_A0619 | 0.3261 | Non-antigen | Non-allergen | Non-toxin | 0.25 |
| Q9KLQ3 | VC_A0689 | 0.2393 | Non-antigen | Non-allergen | Non-toxin | 0.37 |
| Q9KKS6 | VC_A1024 | 0.5352 | Antigen | Non-allergen | Non-toxin | 0.31 |
| Q9KN87 | VC_A0078 | 0.5352 | Antigen | Non-allergen | Non-toxin | 0.31 |
| Q9KU58 | VC_0666 | 0.5688 | Antigen | Non-allergen | Non-toxin | 0.32 |
| Q9KPP0 | VC_2326 | 0.4165 | Antigen | Non-allergen | Non-toxin | 0.49 |
| B1B1N2 | VC_A0594 | 0.5921 | Antigen | Non-allergen | Non-toxin | 0.41 |
| Q9K2J6 | VC_A0319 | 0.5203 | Antigen | Allergen | Non-toxin | 0.32 |
| Q9KS64 | VC_1396 | 0.5297 | Antigen | Allergen | Non-toxin | 0.33 |
| Q9KN40 | VC_A0125 | 0.4146 | Antigen | Allergen | Toxin | 0.79 |
| Q9KVW5 | VC_0023 | 0.3768 | Non-antigen | Non-allergen | Non-toxin | 0.38 |
| Q9KL81 | VC_A0866 | 0.6374 | Antigen | Non-allergen | Non-toxin | 0.12 |
| Q9KPA0 | VC_2473 | 0.3104 | Non-antigen | Non-allergen | Non-toxin | 0.3 |
| Q9KL73 | VC_A0874 | 0.4107 | Antigen | Non-allergen | Non-toxin | 0.46 |
| Q9KNG0 | VC_A0004 | 0.4339 | Antigen | Non-allergen | Non-toxin | 0.42 |
| Q9KSJ4 | VC_1262 | 0.3728 | Non-antigen | Non-allergen | Non-toxin | 0.24 |
| Q9KPZ1 | VC_2221 | 0.5986 | Antigen | Non-allergen | Non-toxin | 0.45 |
| Q9KNI6 | VC_2753 | 0.6258 | Antigen | Non-allergen | Non-toxin | 0.45 |
| Q9KVT0 | VC_0059 | 0.5438 | Antigen | Non-allergen | Non-toxin | 0.36 |
| Q9KST0 | VC_1176 | 0.3796 | Non-antigen | Non-allergen | Non-toxin | 0.3 |

**^ψ^** The potential antigenicity of all the uncharacterized candidate proteins was estimated through “VaxiJen” with a cutoff of value of 0.4. The proteins with a antigenicity score ≥ 0.4 are considered to have antigenic properties.

**^∆^** The toxicity of all the uncharacterized candidate proteins was estimated through “ToxinPred2.0” with a cutoff of value of 0.6. The proteins with values lower than 0.6 are considered to be non-toxic. Whereas, the proteins with values higher than the threshold value of 0.6 are considered to be toxic.
