# Supplementary material for: Comprehensive in silico analyses of fifty-one uncharacterized proteins from Vibrio cholerae
Source: PLoS One. 2024 Oct 4;19(10):e0311301. doi: 10.1371/journal.pone.0311301 (PMC11452002; doi:10.1371/journal.pone.0311301)
Supplement: S11 Table — (DOCX) [file pone.0311301.s011.docx]

**Table S11**

**Prediction of virulence of the candidate protein by VirulentPred.**

| **UniProt ID** | **Amino acid composition** | | **Dipeptide Composition** | | **PSI-BLAST created PSSM Profiles** | | **Cascade of SVMs and PSI- BLAST** | | **Higher order Dipeptide Composition Based** | | **^δ^ Average** |
| --- | --- | --- | --- | --- | --- | --- | --- | --- | --- | --- | --- |
|  | **Results** | **Score** | **Result** | **Score** | **Result** | **Score** | **Result** | **Score** | **Result** | **Score** |  |
| Q9KRD2 | Non-Virulent | -0.606 | Non-Virulent | -0.518 | Non-virulent | -0.817 | Virulent | 0.6402 | Non-Virulent | -0.742 | -0.40856 |
| Q9KVG3 | Virulent | 0.4798 | Virulent | 0.1159 | Virulent | 0.5855 | Virulent | 1.0577 | Virulent | 0.1392 | 0.47462 |
| Q9KT38 | Non-Virulent | -0.141 | Non-Virulent | -0.612 | Virulent | 0.3803 | Non-Virulent | -0.918 | Non-Virulent | -0.422 | -0.34254 |
| Q9KKL8 | Non-Virulent | -1.873 | Non-Virulent | -0.457 | Non-Virulent | -0.016 | Non-Virulent | -0.963 | Non-Virulent | -1.107 | -0.8952 |
| Q9KQX3 | Non-Virulent | -0.514 | Non-Virulent | -0.964 | Non-Virulent | -0.683 | Non-Virulent | -1.047 | Non-Virulent | -0.093 | -0.6602 |
| Q9KLK5 | Virulent | 0.1762 | Non-Virulent | -0.198 | Virulent | 0.3798 | Virulent | 0.7655 | Virulent | 0.3546 | 0.29562 |
| Q9KT24 | Non-virulent | -0.521 | Non-virulent | -0.412 | Non-Virulent | -0.302 | Non-virulent | -1.063 | Non-virulent | -0.412 | -0.5846 |
| Q9KMS2 | Non-Virulent | -0.614 | Non-Virulent | -0.929 | Non-Virulent | -0.711 | Non-Virulent | -0.936 | Virulent | 0.3401 | -0.56998 |
| Q9KMV6 | Non-Virulent | -1.133 | Non-Virulent | -1.390 | Non-Virulent | -0.808 | Non-Virulent | -1.014 | Non-Virulent | -0.523 | -0.9736 |
| Q9KRM9 | Non-Virulent | -0.053 | Non-Virulent | -0.253 | Non-Virulent | -0.521 | Non-Virulent | -0.963 | Non-Virulent | -0.253 | -0.4892 |
| Q9KU75 | Virulent | 0.4881 | Non-Virulent | -0.211 | Non-Virulent | -0.068 | Virulent | 0.1321 | Non-Virulent | -0.304 | 0.00744 |
| Q9KND1 | Virulent | 0.1086 | Virulent | 0.1425 | Virulent | 0.5906 | Virulent | 0.3055 | Non-Virulent | -0.451 | 0.13924 |
| Q9KTC9 | Virulent | 0.3569 | Non-Virulent | -0.791 | Virulent | 0.8628 | Virulent | 0.7431 | Non-Virulent | -0.791 | 0.28136 |
| Q9KSQ9 | Virulent | 0.9607 | Virulent | 0.4131 | Virulent | 0.1944 | Virulent | 0.5332 | Virulent | 1.0196 | 0.6242 |
| Q9KS60 | Non-Virulent | -0.337 | Non-Virulent | -0.501 | Non-Virulent | -0.122 | Virulent | 0.0182 | Non-Virulent | -0.917 | -0.37176 |
| Q9KKX0 | Virulent | 0.1647 | Virulent | 0.2116 | Non-Virulent | -0.146 | Virulent | 0.6094 | Virulent | 0.3411 | 0.23616 |
| Q9KND9 | Virulent | 0.0028 | Non-Virulent | -0.368 | Non-Virulent | -0.354 | Non-Virulent | -0.949 | Non-Virulent | -0.630 | -0.45964 |
| Q9KRJ5 | Non-Virulent | -1.160 | Non-Virulent | -0.584 | Non-Virulent | -1.390 | Non-Virulent | -1.020 | Non-Virulent | -0.393 | -0.9094 |
| Q9KVJ9 | Non-Virulent | -0.103 | Non-Virulent | -0.180 | Non-Virulent | -0.601 | Non-Virulent | -0.910 | Non-Virulent | -0.140 | -1.934 |
| Q9KSV3 | Non-Virulent | -0.860 | Non-Virulent | -0.233 | Non-Virulent | -0.424 | Non-Virulent | -0.989 | Non-Virulent | -0.420 | -0.5852 |
| Q9KSV6 | Virulent | 0.7094 | Virulent | 0.3282 | Virulent | 0.2852 | Virulent | 0.9778 | Virulent | 1.0399 | 0.6681 |
| Q9KND3 | Virulent | 0.5511 | Virulent | 0.3821 | Virulent | 1.1672 | Virulent | 1.0364 | Virulent | 0.1236 | 0.65208 |
| Q9KP29 | Virulent | 0.8772 | Virulent | 0.1104 | Virulent | 1.0597 | Virulent | 0.8765 | Non-Virulent | -0.158 | 0.55316 |
| Q9KMX1 | Virulent | 0.9746 | Virulent | 0.8327 | Virulent | 0.3068 | Virulent | 0.8998 | Virulent | 1.0292 | 0.80862 |
| Q9KTE5 | Non-Virulent | -0.691 | Non-Virulent | -0.157 | Non-Virulent | -0.245 | Non-Virulent | -0.983 | Virulent | 0.1614 | -0.38292 |
| Q9KPD6 | Virulent | 0.5041 | Virulent | 0.3427 | Virulent | 0.8440 | Virulent | 0.1397 | Virulent | 1.0795 | 0.582 |
| Q9KPA3 | Virulent | 0.5376 | Virulent | 0.1531 | Non-Virulent | -1.021 | Non-Virulent | -0.624 | Virulent | 0.4739 | -0.0908 |
| Q9KNF4 | Virulent | 0.7847 | Virulent | 0.1473 | Virulent | 0.5215 | Virulent | 1.0868 | Virulent | 0.2259 | 0.55324 |
| Q9KT53 | Virulent | 0.1233 | Non-Virulent | -0.348 | Non-Virulent | -0.773 | Non-Virulent | -0.802 | Virulent | 0.2428 | -0.31138 |
| Q9KL56 | Virulent | 0.6825 | Virulent | 0.4693 | Virulent | 0.3297 | Virulent | 1.0918 | Virulent | 0.3196 | 0.57858 |
| Q9KRE6 | Non-Virulent | -0.118 | Non-Virulent | -0.017 | Non-Virulent | -0.635 | Non-Virulent | -0.622 | Virulent | 0.4497 | -0.18846 |
| Q9KLX2 | Virulent | 0.4080 | Virulent | 0.5101 | Non-Virulent | -0.657 | Non-Virulent | -0.288 | Virulent | 0.0777 | 0.01016 |
| Q9KLQ3 | Virulent | 1.1013 | Virulent | 0.8439 | Virulent | 0.7527 | Virulent | 1.0035 | Virulent | 0.4628 | 0.83284 |
| Q9KKS6 | Virulent | 0.0713 | Virulent | 0.4446 | Virulent | 0.0297 | Virulent | 0.8055 | Virulent | 0.3543 | 0.34108 |
| Q9KN87 | Non-Virulent | -0.286 | Non-Virulent | -0.071 | Virulent | 0.0093 | Non-Virulent | -0.870 | Non-Virulent | -0.459 | -0.33534 |
| Q9KU58 | Virulent | 1.6919 | Virulent | 0.7159 | Virulent | 0.6321 | Virulent | 0.9618 | Virulent | 0.4652 | 0.89338 |
| Q9KPP0 | Virulent | 0.1875 | Non-Virulent | -0.096 | Virulent | 0.0962 | Virulent | 0.2721 | Non-Virulent | -0.066 | 0.07876 |
| B1B1N2 | Virulent | 0.9980 | Virulent | 0.7913 | Virulent | 0.8197 | Virulent | 0.9925 | Virulent | 0.2121 | 0.76272 |
| Q9K2J6 | Virulent | 0.7905 | Virulent | 0.4865 | Virulent | 0.5794 | Virulent | 1.0280 | Virulent | 0.4882 | 0.67452 |
| Q9KS64 | Virulent | 0.1339 | Virulent | 0.1748 | Virulent | 0.4445 | Virulent | 1.0316 | Virulent | 0.3656 | 0.43008 |
| Q9KN40 | Virulent | 0.5565 | Virulent | 0.3532 | Virulent | 0.4379 | Virulent | 1.0896 | Virulent | 0.7375 | 0.63494 |
| Q9KVW5 | Virulent | 0.9750 | Non-Virulent | -0.087 | Virulent | -0.445 | Non-Virulent | 0.3620 | Virulent | 0.2443 | 0.20986 |
| Q9KL81 | Virulent | 0.9275 | Virulent | 0.6050 | Virulent | 0.7450 | Virulent | 1.0051 | Virulent | 0.9363 | 0.84378 |
| Q9KPA0 | Virulent | 0.0051 | Virulent | 0.2595 | Non-Virulent | -0.100 | Virulent | 0.5674 | Virulent | 0.4859 | 0.24358 |
| Q9KL73 | Virulent | 0.2781 | Virulent | 0.3687 | Virulent | 0.0719 | Virulent | 0.8975 | Virulent | 0.1667 | 0.35658 |
| Q9KNG0 | Virulent | 0.4078 | Virulent | 0.4506 | Virulent | 0.5109 | Virulent | 1.1188 | Virulent | 0.6562 | 0.62886 |
| Q9KSJ4 | Virulent | 0.9028 | Virulent | 0.5351 | Virulent | 0.1068 | Virulent | 0.9083 | Virulent | 0.7468 | 0.63996 |
| Q9KPZ1 | Virulent | 0.2273 | Virulent | 0.3125 | Virulent | 0.0827 | Virulent | 1.0672 | Virulent | 0.6215 | 0.46224 |
| Q9KNI6 | Non-Virulent | -0.737 | Virulent | 0.4262 | Virulent | 0.6489 | Virulent | 0.1771 | Virulent | 0.0760 | 0.11836 |
| Q9KVT0 | Non-Virulent | -0.452 | Virulent | 0.4789 | Virulent | 0.0781 | Virulent | 0.2860 | Virulent | 0.5929 | 0.19678 |
| Q9KST0 | Virulent | 0.3545 | Virulent | 0.4899 | Virulent | 0.0790 | Virulent | 1.1075 | Virulent | 0.6262 | 0.53142 |

**^δ^** The virulence of all the uncharacterized proteins is determined by 5 algorithms based on amino acid composition, dipeptide composition, PSI-BLAST created PSSM profiles, cascade of SVMs and PSI-BLAST and higher order dipeptide composition. A positive average value indicates that the protein is a virulent protein, whereas, a negative value indicates that the protein is a non-virulent protein.
