# Supplementary material for: Comprehensive in silico analyses of fifty-one uncharacterized proteins from Vibrio cholerae
Source: PLoS One. 2024 Oct 4;19(10):e0311301. doi: 10.1371/journal.pone.0311301 (PMC11452002; doi:10.1371/journal.pone.0311301)
Supplement: S12 Table — (DOCX) [file pone.0311301.s012.docx]

**Table S12**

**Identification of linear B-cell epitopes present within the candidate proteins**

| **Position** | | **Amino acid residues** | **^ℷ^Score** |
| --- | --- | --- | --- |
| **UniProt ID- Q9KRD2** | | | |
| **5** | | **Ser** | **0.515** |
| **6** | | **Thr** | **0.534** |
| **7** | | **Val** | **0.54** |
| **8** | | **Gln** | **0.55** |
| **9** | | **Pro** | **0.543** |
| **10** | | **Gln** | **0.536** |
| **11** | | **Ser** | **0.535** |
| **12** | | **Ile** | **0.541** |
| **13** | | **Gly** | **0.544** |
| **14** | | **Leu** | **0.536** |
| **15** | | **Lys** | **0.529** |
| **16** | | **Thr** | **0.515** |
| **30** | | **Gln** | **0.506** |
| **31** | | **His** | **0.508** |
| **32** | | **Met** | **0.503** |
| **43** | | **Val** | **0.513** |
| **44** | | **Gly** | **0.547** |
| **45** | | **Asn** | **0.55** |
| **46** | | **Ser** | **0.559** |
| **47** | | **Arg** | **0.566** |
| **48** | | **Ser** | **0.571** |
| **49** | | **Ile** | **0.57** |
| **50** | | **Thr** | **0.573** |
| **51** | | **Lys** | **0.555** |
| **52** | | **Pro** | **0.541** |
| **53** | | **Asp** | **0.534** |
| **54** | | **Asp** | **0.535** |
| **55** | | **Fro** | **0.528** |
| **56** | | **Leu** | **0.543** |
| **57** | | **Glu** | **0.558** |
| **58** | | **Tyr** | **0.566** |
| **59** | | **Leu** | **0.562** |
| **60** | | **Pro** | **0.587** |
| **61** | | **Leu** | **0.595** |
| **62** | | **Thr** | **0.599** |
| **63** | | **Gln** | **0.589** |
| **64** | | **Arg** | **0.596** |
| **65** | | **Glu** | **0.582** |
| **66** | | **His** | **0.575** |
| **67** | | **Ala** | **0.547** |
| **68** | | **Ala** | **0.537** |
| **69** | | **Gly** | **0.523** |
| **70** | | **Phe** | **0.508** |
| **71** | | **Phe** | **0.513** |
| **72** | | **Val** | **0.528** |
| **73** | | **Gln** | **0.531** |
| **74** | | **Ala** | **0.535** |
| **75** | | **Glu** | **0.537** |
| **76** | | **Leu** | **0.549** |
| **77** | | **Ala** | **0.538** |
| **78** | | **Pro** | **0.521** |
| **79** | | **Gln** | **0.504** |
| **100** | | **Asp** | **0.506** |
| **101** | | **Glu** | **0.528** |
| **102** | | **Val** | **0.546** |
| **103** | | **Ser** | **0.537** |
| **104** | | **Ser** | **0.531** |
| **122** | | **GLU** | **0.501** |
| **124** | | **ALA** | **0.503** |
| **125** | | **PHE** | **0.503** |
| **155** | | **GLU** | **0.511** |
| **156** | | **GLN** | **0.524** |
| **157** | | **GLN** | **0.529** |
| **158** | | **THR** | **0.531** |
| **159** | | **GLY** | **0.561** |
| **160** | | **TYR** | **0.567** |
| **161** | | **GLN** | **0.561** |
| **162** | | **GLN** | **0.576** |
| **163** | | **ASN** | **0.58** |
| **164** | | **GLU** | **0.578** |
| **165** | | **LEU** | **0.58** |
| **166** | | **LEU** | **0.561** |
| **167** | | **GLY** | **0.556** |
| **168** | | **LEU** | **0.546** |
| **169** | | **LYS** | **0.521** |
| **170** | | **THR** | **0.534** |
| **171** | | **SER** | **0.528** |
| **172** | | **MET** | **0.532** |
| **173** | | **PHE** | **0.546** |
| **174** | | **ASN** | **0.543** |
| **175** | | **SER** | **0.554** |
| **176** | | **GLY** | **0.556** |
| **177** | | **LYS** | **0.563** |
| **178** | | **HIS** | **0.575** |
| **179** | | **SER** | **0.563** |
| **180** | | **GLN** | **0.561** |
| **181** | | **HIS** | **0.546** |
| **182** | | **PHE** | **0.51** |
| **183** | | **TYR** | **0.512** |
| **184** | | **VAL** | **0.515** |
| **191** | | **ARG** | **0.501** |
| **238** | | **HIS** | **0.508** |
| **239** | | **LEU** | **0.515** |
| **240** | | **ASP** | **0.528** |
| **241** | | **ARG** | **0.532** |
| **242** | | **VAL** | **0.538** |
| **243** | | **LEU** | **0.545** |
| **244** | | **ASP** | **0.539** |
| **245** | | **LYS** | **0.543** |
| **246** | | **GLN** | **0.534** |
| **247** | | **ALA** | **0.534** |
| **248** | | **GLY** | **0.541** |
| **249** | | **ASP** | **0.528** |
| **250** | | **VAL** | **0.522** |
| **251** | | **GLU** | **0.523** |
| **252** | | **LEU** | **0.516** |
| **253** | | **LEU** | **0.53** |
| **254** | | **THR** | **0.527** |
| **255** | | **GLN** | **0.529** |
| **256** | | **LEU** | **0.513** |
| **257** | | **PRO** | **0.51** |
| **271** | | **PRO** | **0.509** |
| **286** | | **LYS** | **0.52** |
| **287** | | **PHE** | **0.554** |
| **288** | | **SER** | **0.561** |
| **289** | | **SER** | **0.566** |
| **290** | | **ASP** | **0.572** |
| **291** | | **LYS** | **0.567** |
| **292** | | **TYR** | **0.556** |
| **293** | | **TYR** | **0.571** |
| **294** | | **ALA** | **0.578** |
| **295** | | **GLN** | **0.546** |
| **296** | | **ILE** | **0.525** |
| **297** | | **ARG** | **0.515** |
| **325** | | **ASP** | **0.511** |
| **326** | | **ALA** | **0.529** |
| **327** | | **GLU** | **0.553** |
| **328** | | **LYS** | **0.565** |
| **329** | | **ASN** | **0.58** |
| **330** | | **LEU** | **0.578** |
| **331** | | **GLU** | **0.584** |
| **332** | | **ALA** | **0.575** |
| **333** | | **GLN** | **0.57** |
| **334** | | **PRO** | **0.537** |
| **335** | | **LEU** | **0.535** |
| **336** | | **VAL** | **0.525** |
| **337** | | **GLY** | **0.507** |
| **354** | | **GLY** | **0.52** |
| **355** | | **GLN** | **0.511** |
| **375** | | **THR** | **0.502** |
| **376** | | **ASP** | **0.53** |
| **377** | | **LYS** | **0.518** |
| **378** | | **ILE** | **0.51** |
| **379** | | **GLU** | **0.501** |
| **401** | | **ASN** | **0.505** |
| **402** | | **PHE** | **0.508** |
| **404** | | **ASP** | **0.501** |
| **405** | | **ARG** | **0.512** |
| **406** | | **ALA** | **0.516** |
| **407** | | **ILE** | **0.513** |
| **408** | | **HIS** | **0.529** |
| **409** | | **GLN** | **0.528** |
| **410** | | **GLN** | **0.53** |
| **411** | | **ILE** | **0.531** |
| **412** | | **GLU** | **0.548** |
| **413** | | **ARG** | **0.536** |
| **414** | | **ARG** | **0.526** |
| **415** | | **LYS** | **0.523** |
| **416** | | **THR** | **0.545** |
| **417** | | **LEU** | **0.519** |
| **418** | | **GLU** | **0.537** |
| **419** | | **LYS** | **0.529** |
| **420** | | **TRP** | **0.516** |
| **459** | | **LEU** | **0.505** |
| **461** | | **HIS** | **0.518** |
| **462** | | **ALA** | **0.522** |
| **463** | | **SER** | **0.502** |
| **498** | | **LEU** | **0.503** |
| **500** | | **GLY** | **0.507** |
| **514** | | **ASP** | **0.525** |
| **515** | | **LEU** | **0.546** |
| **516** | | **SER** | **0.535** |
| **517** | | **ALA** | **0.527** |
| **518** | | **GLU** | **0.528** |
| **519** | | **GLN** | **0.517** |
| **520** | | **ILE** | **0.506** |
| **521** | | **LEU** | **0.502** |
| **522** | | **GLN** | **0.505** |
| **549** | | **ASP** | **0.51** |
| **550** | | **SER** | **0.513** |
| **551** | | **GLN** | **0.52** |
| **552** | | **SER** | **0.522** |
| **553** | | **GLN** | **0.53** |
| **588** | | **PHE** | **0.522** |
| **589** | | **ASP** | **0.513** |
| **590** | | **TYR** | **0.513** |
| **591** | | **LEU** | **0.512** |
| **592** | | **LYS** | **0.503** |
| **593** | | **ILE** | **0.505** |
| **603** | | **VAL** | **0.521** |
| **604** | | **GLY** | **0.521** |
| **605** | | **SER** | **0.517** |
| **606** | | **ARG** | **0.51** |
| **632** | | **THR** | **0.523** |
| **633** | | **GLU** | **0.537** |
| **634** | | **HIS** | **0.535** |
| **635** | | **GLU** | **0.516** |
| **636** | | **VAL** | **0.516** |
| **637** | | **TYR** | **0.513** |
| **638** | | **VAL** | **0.51** |
| **654** | | **LYS** | **0.502** |
| **655** | | **PRO** | **0.519** |
| **656** | | **LEU** | **0.535** |
| **657** | | **PRO** | **0.528** |
| **658** | | **LEU** | **0.536** |
| **659** | | **SER** | **0.526** |
| **660** | | **ASN** | **0.513** |
| **666** | | **ASP** | **0.528** |
| **667** | | **TYR** | **0.529** |
| **668** | | **ARG** | **0.541** |
| **669** | | **LYS** | **0.555** |
| **670** | | **HIS** | **0.572** |
| **671** | | **LEU** | **0.579** |
| **672** | | **LYS** | **0.583** |
| **673** | | **LEU** | **0.588** |
| **674** | | **THR** | **0.59** |
| **675** | | **ASN** | **0.588** |
| **676** | | **ARG** | **0.599** |
| **677** | | **GLU** | **0.606** |
| **678** | | **SER** | **0.607** |
| **679** | | **ASP** | **0.62** |
| **680** | | **LYS** | **0.619** |
| **681** | | **GLN** | **0.613** |
| **682** | | **SER** | **0.602** |
| **683** | | **ARG** | **0.584** |
| **684** | | **GLU** | **0.56** |
| **685** | | **ILE** | **0.565** |
| **686** | | **THR** | **0.555** |
| **687** | | **THR** | **0.547** |
| **688** | | **LEU** | **0.551** |
| **689** | | **GLN** | **0.549** |
| **690** | | **SER** | **0.556** |
| **691** | | **LEU** | **0.571** |
| **692** | | **ALA** | **0.594** |
| **693** | | **ILE** | **0.632** |
| **694** | | **PHE** | **0.639** |
| **695** | | **THR** | **0.65** |
| **696** | | **GLN** | **0.667** |
| **697** | | **TYR** | **0.655** |
| **698** | | **ARG** | **0.655** |
| **699** | | **LEU** | **0.65** |
| **700** | | **ASP** | **0.634** |
| **701** | | **PRO** | **0.619** |
| **702** | | **GLY** | **0.59** |
| **703** | | **ASP** | **0.567** |
| **704** | | **PRO** | **0.518** |
| **705** | | **LEU** | **0.504** |
| **713** | | **ASN** | **0.516** |
| **714** | | **ALA** | **0.516** |
| **715** | | **VAL** | **0.504** |
| **740** | | **HIS** | **0.503** |
| **743** | | **PRO** | **0.505** |
| **744** | | **THR** | **0.531** |
| **745** | | **MET** | **0.531** |
| **746** | | **GLY** | **0.559** |
| **747** | | **THR** | **0.568** |
| **748** | | **GLU** | **0.551** |
| **749** | | **LEU** | **0.542** |
| **750** | | **GLU** | **0.54** |
| **751** | | **THR** | **0.536** |
| **752** | | **ALA** | **0.528** |
| **753** | | **ARG** | **0.514** |
| **754** | | **GLU** | **0.504** |
| **756** | | **ALA** | **0.505** |
| **770** | | **GLN** | **0.508** |
| **771** | | **VAL** | **0.549** |
| **772** | | **LYS** | **0.565** |
| **773** | | **LEU** | **0.577** |
| **774** | | **ALA** | **0.586** |
| **775** | | **HIS** | **0.571** |
| **776** | | **ASP** | **0.563** |
| **777** | | **THR** | **0.541** |
| **778** | | **ASP** | **0.535** |
| **779** | | **LEU** | **0.516** |
| **788** | | **THR** | **0.504** |
| **789** | | **ASN** | **0.511** |
| **790** | | **PRO** | **0.525** |
| **791** | | **PRO** | **0.54** |
| **792** | | **PRO** | **0.526** |
| **793** | | **PRO** | **0.532** |
| **794** | | **TRP** | **0.526** |
| **795** | | **VAL** | **0.519** |
| **796** | | **LEU** | **0.518** |
| **797** | | **VAL** | **0.514** |
| **798** | | **GLU** | **0.512** |
| **799** | | **GLY** | **0.522** |
| **800** | | **ASN** | **0.527** |
| **801** | | **LYS** | **0.531** |
| **802** | | **TYR** | **0.508** |
| **803** | | **LYS** | **0.506** |
| **UniProt ID- Q9KVG3** | | | |
| **5** | | **GLU** | **0.51** |
| **6** | | **LEU** | **0.52** |
| **7** | | **PRO** | **0.538** |
| **8** | | **LYS** | **0.55** |
| **9** | | **ASN** | **0.561** |
| **10** | | **ILE** | **0.559** |
| **11** | | **LEU** | **0.585** |
| **12** | | **LYS** | **0.606** |
| **13** | | **GLU** | **0.602** |
| **14** | | **ASN** | **0.616** |
| **15** | | **LEU** | **0.632** |
| **16** | | **SER** | **0.637** |
| **17** | | **GLU** | **0.63** |
| **18** | | **TYR** | **0.619** |
| **19** | | **LYS** | **0.618** |
| **20** | | **ASN** | **0.602** |
| **21** | | **ILE** | **0.578** |
| **22** | | **VAL** | **0.575** |
| **23** | | **GLN** | **0.567** |
| **24** | | **LEU** | **0.555** |
| **25** | | **TYR** | **0.553** |
| **26** | | **SER** | **0.539** |
| **27** | | **ASP** | **0.558** |
| **28** | | **GLY** | **0.561** |
| **29** | | **LYS** | **0.551** |
| **30** | | **LEU** | **0.552** |
| **31** | | **LYS** | **0.544** |
| **32** | | **LYS** | **0.527** |
| **33** | | **LEU** | **0.508** |
| **43** | | **ASP** | **0.51** |
| **44** | | **GLY** | **0.516** |
| **45** | | **SER** | **0.515** |
| **54** | | **GLU** | **0.506** |
| **55** | | **TRP** | **0.528** |
| **56** | | **ASP** | **0.528** |
| **57** | | **PHE** | **0.531** |
| **58** | | **ARG** | **0.534** |
| **59** | | **ALA** | **0.528** |
| **60** | | **TYR** | **0.547** |
| **61** | | **LEU** | **0.549** |
| **62** | | **ASP** | **0.567** |
| **63** | | **ALA** | **0.571** |
| **64** | | **ARG** | **0.574** |
| **65** | | **ILE** | **0.577** |
| **66** | | **VAL** | **0.571** |
| **67** | | **TYR** | **0.537** |
| **68** | | **LYS** | **0.537** |
| **76** | | **ILE** | **0.515** |
| **77** | | **VAL** | **0.511** |
| **78** | | **SER** | **0.533** |
| **79** | | **ASP** | **0.543** |
| **80** | | **ASP** | **0.53** |
| **81** | | **LEU** | **0.501** |
| **97** | | **GLY** | **0.529** |
| **98** | | **HIS** | **0.577** |
| **99** | | **HIS** | **0.593** |
| **100** | | **ARG** | **0.619** |
| **101** | | **LYS** | **0.629** |
| **102** | | **GLY** | **0.636** |
| **103** | | **ALA** | **0.623** |
| **104** | | **VAL** | **0.598** |
| **105** | | **ILE** | **0.578** |
| **106** | | **LYS** | **0.53** |
| **107** | | **PRO** | **0.518** |
| **129** | | **PHE** | **0.524** |
| **130** | | **GLN** | **0.531** |
| **131** | | **SER** | **0.525** |
| **132** | | **ILE** | **0.527** |
| **133** | | **ASN** | **0.526** |
| **134** | | **SER** | **0.538** |
| **135** | | **LEU** | **0.537** |
| **136** | | **SER** | **0.531** |
| **137** | | **SER** | **0.528** |
| **138** | | **GLU** | **0.509** |
| **139** | | **ILE** | **0.503** |
| **149** | | **LYS** | **0.503** |
| **150** | | **GLY** | **0.504** |
| **151** | | **GLN** | **0.518** |
| **152** | | **ASN** | **0.535** |
| **153** | | **TYR** | **0.565** |
| **154** | | **SER** | **0.548** |
| **155** | | **SER** | **0.552** |
| **156** | | **GLY** | **0.545** |
| **157** | | **GLN** | **0.523** |
| **173** | | **ARG** | **0.503** |
| **174** | | **TYR** | **0.521** |
| **175** | | **LEU** | **0.525** |
| **176** | | **PRO** | **0.55** |
| **177** | | **ILE** | **0.551** |
| **178** | | **GLN** | **0.56** |
| **179** | | **PHE** | **0.568** |
| **180** | | **GLU** | **0.578** |
| **181** | | **ILE** | **0.589** |
| **182** | | **PRO** | **0.582** |
| **183** | | **GLN** | **0.583** |
| **184** | | **GLY** | **0.58** |
| **185** | | **ARG** | **0.572** |
| **186** | | **THR** | **0.583** |
| **187** | | **GLN** | **0.563** |
| **188** | | **CYS** | **0.548** |
| **189** | | **LYS** | **0.547** |
| **190** | | **PHE** | **0.535** |
| **191** | | **GLY** | **0.536** |
| **192** | | **ARG** | **0.541** |
| **193** | | **GLU** | **0.553** |
| **194** | | **ILE** | **0.556** |
| **195** | | **THR** | **0.567** |
| **196** | | **GLY** | **0.581** |
| **197** | | **ARG** | **0.596** |
| **198** | | **LEU** | **0.611** |
| **199** | | **TYR** | **0.614** |
| **200** | | **GLU** | **0.621** |
| **201** | | **GLY** | **0.596** |
| **202** | | **LYS** | **0.587** |
| **203** | | **ASN** | **0.58** |
| **204** | | **GLN** | **0.551** |
| **205** | | **PHE** | **0.539** |
| **206** | | **TYR** | **0.519** |
| **207** | | **ALA** | **0.501** |
| **229** | | **PRO** | **0.543** |
| **230** | | **TYR** | **0.541** |
| **231** | | **LYS** | **0.547** |
| **232** | | **GLU** | **0.555** |
| **233** | | **GLU** | **0.557** |
| **234** | | **ILE** | **0.554** |
| **235** | | **HIS** | **0.552** |
| **236** | | **GLU** | **0.556** |
| **237** | | **LEU** | **0.521** |
| **238** | | **LEU** | **0.51** |
| **239** | | **TYR** | **0.505** |
| **241** | **MET** | **0.504** | |
| **242** | **ARG** | **0.509** | |
| **243** | **GLN** | **0.512** | |
| **244** | **ASN** | **0.514** | |
| **245** | **TYR** | **0.529** | |
| **246** | **GLU** | **0.559** | |
| **247** | **GLU** | **0.554** | |
| **248** | **GLY** | **0.55** | |
| **249** | **LYS** | **0.56** | |
| **250** | **ARG** | **0.556** | |
| **251** | **ARG** | **0.558** | |
| **252** | **VAL** | **0.563** | |
| **253** | **ASP** | **0.565** | |
| **254** | **ASP** | **0.561** | |
| **255** | **LYS** | **0.56** | |
| **256** | **ILE** | **0.56** | |
| **257** | **GLN** | **0.57** | |
| **258** | **SER** | **0.553** | |
| **259** | **GLY** | **0.563** | |
| **260** | **ILE** | **0.551** | |
| **261** | **TRP** | **0.54** | |
| **262** | **GLN** | **0.528** | |
| **263** | **TRP** | **0.535** | |
| **264** | **ILE** | **0.529** | |
| **265** | **SER** | **0.535** | |
| **266** | **GLU** | **0.541** | |
| **267** | **GLU** | **0.549** | |
| **268** | **SER** | **0.544** | |
| **269** | **ASN** | **0.561** | |
| **270** | **GLU** | **0.577** | |
| **271** | **TYR** | **0.57** | |
| **272** | **ARG** | **0.58** | |
| **273** | **VAL** | **0.598** | |
| **274** | **GLU** | **0.602** | |
| **275** | **VAL** | **0.61** | |
| **276** | **ASN** | **0.618** | |
| **277** | **LYS** | **0.626** | |
| **278** | **HIS** | **0.626** | |
| **279** | **GLN** | **0.614** | |
| **280** | **PRO** | **0.627** | |
| **281** | **ALA** | **0.601** | |
| **282** | **SER** | **0.573** | |
| **283** | **TYR** | **0.556** | |
| **284** | **SER** | **0.548** | |
| **285** | **SER** | **0.534** | |
| **286** | **ILE** | **0.522** | |
| **287** | **ILE** | **0.505** | |
| **288** | **ASP** | **0.524** | |
| **289** | **ALA** | **0.535** | |
| **290** | **HIS** | **0.554** | |
| **291** | **ILE** | **0.577** | |
| **292** | **LYS** | **0.568** | |
| **293** | **ASP** | **0.574** | |
| **294** | **THR** | **0.585** | |
| **295** | **HIS** | **0.594** | |
| **296** | **LEU** | **0.617** | |
| **297** | **GLU** | **0.595** | |
| **298** | **SER** | **0.59** | |
| **299** | **LEU** | **0.588** | |
| **300** | **LEU** | **0.587** | |
| **301** | **PRO** | **0.612** | |
| **302** | **LYS** | **0.602** | |
| **303** | **SER** | **0.583** | |
| **304** | **ALA** | **0.574** | |
| **305** | **LEU** | **0.547** | |
| **306** | **ARG** | **0.55** | |
| **307** | **ILE** | **0.518** | |
| **338** | **SER** | **0.511** | |
| **339** | **ASN** | **0.511** | |
| **340** | **SER** | **0.525** | |
| **341** | **PHE** | **0.552** | |
| **342** | **LYS** | **0.563** | |
| **343** | **GLU** | **0.574** | |
| **344** | **LYS** | **0.575** | |
| **345** | **GLU** | **0.576** | |
| **346** | **MET** | **0.575** | |
| **347** | **TYR** | **0.555** | |
| **348** | **GLY** | **0.525** | |
| **359** | **HIS** | **0.502** | |
| **360** | **LYS** | **0.52** | |
| **361** | **PHE** | **0.554** | |
| **362** | **SER** | **0.568** | |
| **363** | **GLN** | **0.587** | |
| **364** | **GLY** | **0.583** | |
| **365** | **ARG** | **0.581** | |
| **366** | **GLY** | **0.584** | |
| **367** | **LYS** | **0.574** | |
| **368** | **MET** | **0.547** | |
| **369** | **ALA** | **0.523** | |
| **395** | **LEU** | **0.504** | |
| **396** | **LEU** | **0.546** | |
| **397** | **GLU** | **0.552** | |
| **398** | **ASP** | **0.571** | |
| **399** | **ASP** | **0.584** | |
| **400** | **ASP** | **0.597** | |
| **401** | **PRO** | **0.606** | |
| **402** | **MET** | **0.612** | |
| **403** | **SER** | **0.608** | |
| **404** | **VAL** | **0.608** | |
| **405** | **HIS** | **0.601** | |
| **406** | **ASN** | **0.606** | |
| **407** | **SER** | **0.59** | |
| **408** | **SER** | **0.586** | |
| **409** | **CYS** | **0.586** | |
| **410** | **LEU** | **0.584** | |
| **411** | **TRP** | **0.582** | |
| **412** | **LEU** | **0.605** | |
| **413** | **GLY** | **0.603** | |
| **414** | **GLN** | **0.605** | |
| **415** | **GLY** | **0.6** | |
| **416** | **ARG** | **0.606** | |
| **417** | **LYS** | **0.614** | |
| **418** | **SER** | **0.614** | |
| **419** | **GLN** | **0.603** | |
| **420** | **LYS** | **0.599** | |
| **421** | **PRO** | **0.578** | |
| **422** | **ILE** | **0.572** | |
| **423** | **ILE** | **0.571** | |
| **424** | **ARG** | **0.574** | |
| **425** | **GLN** | **0.565** | |
| **426** | **ASP** | **0.562** | |
| **427** | **ASN** | **0.561** | |
| **428** | **ASN** | **0.558** | |
| **429** | **MET** | **0.533** | |
| **430** | **ARG** | **0.542** | |
| **431** | **THR** | **0.54** | |
| **432** | **HIS** | **0.531** | |
| 438 | GLU | 0.506 | |
| **442** | **ALA** | **0.519** | |
| **443** | **LEU** | **0.526** | |
| **444** | **ILE** | **0.536** | |
| **445** | **THR** | **0.539** | |
| **446** | **GLU** | **0.537** | |
| **447** | **ASP** | **0.546** | |
| **448** | **ASP** | **0.543** | |
| **449** | **LEU** | **0.556** | |
| **450** | **ASP** | **0.571** | |
| **451** | **GLU** | **0.578** | |
| **452** | **PHE** | **0.58** | |
| **453** | **ARG** | **0.578** | |
| **454** | **VAL** | **0.592** | |
| **455** | **ILE** | **0.612** | |
| **456** | **ASN** | **0.628** | |
| **457** | **PRO** | **0.643** | |
| **458** | **ASN** | **0.641** | |
| **459** | **ARG** | **0.635** | |
| **460** | **GLU** | **0.628** | |
| **461** | **PRO** | **0.635** | |
| **462** | **ASN** | **0.642** | |
| **463** | **ASN** | **0.644** | |
| **464** | **ALA** | **0.635** | |
| **465** | **ASP** | **0.631** | |
| **466** | **GLU** | **0.624** | |
| **467** | **ARG** | **0.621** | |
| **468** | **LEU** | **0.631** | |
| **469** | **LYS** | **0.63** | |
| **470** | **VAL** | **0.625** | |
| **471** | **GLY** | **0.617** | |
| **472** | **ASN** | **0.599** | |
| **473** | **VAL** | **0.587** | |
| **474** | **TRP** | **0.558** | |
| **475** | **PRO** | **0.546** | |
| **476** | **LEU** | **0.529** | |
| **493** | **ASN** | **0.506** | |
| **494** | **LEU** | **0.521** | |
| **495** | **CYS** | **0.528** | |
| **505** | **LYS** | **0.511** | |
| **506** | **HIS** | **0.521** | |
| **507** | **LEU** | **0.526** | |
| **508** | **ASP** | **0.508** | |
| **509** | **LEU** | **0.521** | |
| **510** | **PRO** | **0.512** | |
| **511** | **THR** | **0.501** | |
| **513** | **GLU** | **0.5** | |
| **517** | **GLU** | **0.508** | |
| **518** | **GLY** | **0.506** | |
| **520** | **LEU** | **0.51** | |
| **521** | **ALA** | **0.534** | |
| **522** | **SER** | **0.522** | |
| **523** | **LYS** | **0.524** | |
| **524** | **ILE** | **0.523** | |
| **525** | **LYS** | **0.523** | |
| **526** | **ALA** | **0.53** | |
| **527** | **LEU** | **0.536** | |
| **528** | **GLN** | **0.545** | |
| **529** | **ILE** | **0.541** | |
| **530** | **ASP** | **0.544** | |
| **531** | **THR** | **0.537** | |
| **532** | **GLU** | **0.547** | |
| **533** | **LEU** | **0.524** | |
| **534** | **GLN** | **0.531** | |
| **535** | **SER** | **0.518** | |
| **536** | **PHE** | **0.523** | |
| **537** | **LEU** | **0.52** | |
| **538** | **ASN** | **0.529** | |
| **539** | **ASP** | **0.509** | |
| **540** | **VAL** | **0.518** | |
| **541** | **ILE** | **0.516** | |
| **542** | **GLN** | **0.546** | |
| **543** | **GLU** | **0.544** | |
| **544** | **SER** | **0.568** | |
| **545** | **THR** | **0.569** | |
| **546** | **THR** | **0.571** | |
| **547** | **GLN** | **0.566** | |
| **548** | **LYS** | **0.586** | |
| **549** | **ILE** | **0.589** | |
| **550** | **HIS** | **0.591** | |
| **551** | **GLU** | **0.588** | |
| **552** | **TRP** | **0.579** | |
| **553** | **TYR** | **0.576** | |
| **554** | **LYS** | **0.573** | |
| **555** | **GLY** | **0.572** | |
| **556** | **CYS** | **0.588** | |
| **557** | **ASP** | **0.57** | |
| **558** | **SER** | **0.563** | |
| **559** | **GLY** | **0.553** | |
| **560** | **GLN** | **0.552** | |
| **561** | **LEU** | **0.549** | |
| **562** | **MET** | **0.528** | |
| **563** | **GLY** | **0.518** | |
| **564** | **HIS** | **0.514** | |
| **569** | **ILE** | **0.505** | |
| **570** | **ASN** | **0.519** | |
| **571** | **LYS** | **0.535** | |
| **572** | **ASN** | **0.543** | |
| **573** | **ARG** | **0.555** | |
| **574** | **ILE** | **0.582** | |
| **575** | **SER** | **0.591** | |
| **576** | **LEU** | **0.598** | |
| **577** | **HIS** | **0.604** | |
| **578** | **LYS** | **0.6** | |
| **579** | **LYS** | **0.6** | |
| **580** | **TYR** | **0.595** | |
| **581** | **LYS** | **0.604** | |
| **582** | **SER** | **0.579** | |
| **583** | **TRP** | **0.567** | |
| **584** | **ASP** | **0.553** | |
| **585** | **ALA** | **0.528** | |
| **610** | **GLU** | **0.523** | |
| **611** | **CYS** | **0.549** | |
| **612** | **GLN** | **0.554** | |
| **613** | **MET** | **0.58** | |
| **614** | **HIS** | **0.576** | |
| **615** | **LYS** | **0.565** | |
| **616** | **VAL** | **0.593** | |
| **617** | **SER** | **0.585** | |
| **618** | **SER** | **0.568** | |
| **619** | **PRO** | **0.57** | |
| **620** | **ALA** | **0.566** | |
| **621** | **ASN** | **0.554** | |
| **622** | **CYS** | **0.543** | |
| **623** | **MET** | **0.545** | |
| **624** | **SER** | **0.554** | |
| **625** | **CYS** | **0.543** | |
| **626** | **GLU** | **0.539** | |
| **627** | **ASN** | **0.544** | |
| **628** | **GLN** | **0.54** | |
| **629** | **LEU** | **0.542** | |
| **630** | **ILE** | **0.567** | |
| **631** | **ASP** | **0.559** | |
| **632** | **LYS** | **0.561** | |
| **633** | **GLU** | **0.559** | |
| **634** | **LYS** | **0.561** | |
| **635** | **ALA** | **0.569** | |
| **636** | **GLU** | **0.582** | |
| **637** | **ASN** | **0.581** | |
| **638** | **TRP** | **0.578** | |
| **639** | **ASP** | **0.572** | |
| **640** | **LYS** | **0.585** | |
| **641** | **ARG** | **0.558** | |
| **642** | **TYR** | **0.557** | |
| **643** | **GLN** | **0.555** | |
| **644** | **TRP** | **0.536** | |
| **645** | **VAL** | **0.506** | |
| **646** | **CYS** | **0.501** | |
| **654** | **ALA** | **0.509** | |
| **655** | **ILE** | **0.519** | |
| **656** | **GLY** | **0.532** | |
| **657** | **SER** | **0.559** | |
| **658** | **LEU** | **0.561** | |
| **659** | **THR** | **0.567** | |
| **660** | **SER** | **0.564** | |
| **661** | **SER** | **0.55** | |
| **662** | **MET** | **0.525** | |
| **663** | **TYR** | **0.527** | |
| **664** | **SER** | **0.502** | |
| **682** | **ILE** | **0.502** | |
| **683** | **PRO** | **0.501** | |
| **685** | **THR** | **0.504** | |
| **686** | **ARG** | **0.502** | |
| **687** | **PHE** | **0.522** | |
| **688** | **GLU** | **0.53** | |
| **689** | **ILE** | **0.55** | |
| **690** | **GLY** | **0.562** | |
| **691** | **ASN** | **0.576** | |
| **692** | **ASP** | **0.592** | |
| **693** | **ARG** | **0.582** | |
| **694** | **TYR** | **0.594** | |
| **695** | **GLU** | **0.6** | |
| **696** | **GLN** | **0.586** | |
| **697** | **ILE** | **0.581** | |
| **698** | **PRO** | **0.563** | |
| **UniProt ID- Q9KT38** | | | |
| **54** | **THR** | **0.505** | |
| **55** | **GLU** | **0.502** | |
| **77** | **GLU** | **0.503** | |
| **78** | **TYR** | **0.515** | |
| **79** | **VAL** | **0.533** | |
| **80** | **ASN** | **0.53** | |
| **81** | **TYR** | **0.55** | |
| **82** | **PRO** | **0.558** | |
| **83** | **SER** | **0.54** | |
| **84** | **PHE** | **0.515** | |
| **85** | **ASN** | **0.506** | |
| **102** | **ILE** | **0.501** | |
| **103** | **THR** | **0.529** | |
| **104** | **TYR** | **0.541** | |
| **105** | **THR** | **0.558** | |
| **106** | **ASP** | **0.56** | |
| **107** | **LEU** | **0.57** | |
| **108** | **ARG** | **0.553** | |
| **109** | **ALA** | **0.557** | |
| **110** | **GLN** | **0.538** | |
| **111** | **ARG** | **0.535** | |
| **112** | **PHE** | **0.52** | |
| **113** | **ASP** | **0.517** | |
| **114** | **PHE** | **0.506** | |
| **125** | **LEU** | **0.51** | |
| **126** | **TYR** | **0.542** | |
| **127** | **SER** | **0.548** | |
| **128** | **TYR** | **0.579** | |
| **129** | **GLY** | **0.572** | |
| **130** | **GLY** | **0.577** | |
| **131** | **LEU** | **0.57** | |
| **132** | **ARG** | **0.561** | |
| **133** | **LEU** | **0.555** | |
| **134** | **PRO** | **0.529** | |
| **152** | **LYS** | **0.5** | |
| **153** | **GLU** | **0.522** | |
| **154** | **HIS** | **0.521** | |
| **155** | **TYR** | **0.516** | |
| **156** | **PRO** | **0.532** | |
| **157** | **TYR** | **0.524** | |
| **158** | **ILE** | **0.533** | |
| **159** | **GLN** | **0.522** | |
| **160** | **GLN** | **0.529** | |
| **161** | **VAL** | **0.516** | |
| **162** | **GLU** | **0.511** | |
| **163** | **TYR** | **0.52** | |
| **164** | **GLU** | **0.523** | |
| **165** | **GLY** | **0.508** | |
| **195** | **GLY** | **0.524** | |
| **196** | **LEU** | **0.561** | |
| **197** | **ASP** | **0.577** | |
| **198** | **VAL** | **0.588** | |
| **199** | **GLN** | **0.588** | |
| **200** | **LEU** | **0.6** | |
| **201** | **LEU** | **0.609** | |
| **202** | **ASN** | **0.611** | |
| **203** | **ASP** | **0.61** | |
| **204** | **GLN** | **0.603** | |
| **205** | **LEU** | **0.588** | |
| **206** | **PRO** | **0.574** | |
| **207** | **ILE** | **0.561** | |
| **208** | **GLN** | **0.543** | |
| **209** | **PRO** | **0.505** | |
| **215** | **PRO** | **0.515** | |
| **216** | **LYS** | **0.528** | |
| **217** | **GLY** | **0.532** | |
| **218** | **LYS** | **0.536** | |
| **219** | **HIS** | **0.55** | |
| **220** | **SER** | **0.535** | |
| **221** | **ALA** | **0.514** | |
| **227** | **GLU** | **0.511** | |
| **228** | **LYS** | **0.515** | |
| **229** | **TYR** | **0.526** | |
| **230** | **ALA** | **0.531** | |
| **231** | **HIS** | **0.548** | |
| **232** | **SER** | **0.55** | |
| **233** | **ALA** | **0.548** | |
| **234** | **HIS** | **0.54** | |
| **235** | **VAL** | **0.552** | |
| **236** | **GLN** | **0.559** | |
| **237** | **ARG** | **0.553** | |
| **238** | **LEU** | **0.562** | |
| **239** | **LEU** | **0.572** | |
| **240** | **ARG** | **0.584** | |
| **241** | **GLU** | **0.589** | |
| **242** | **SER** | **0.597** | |
| **243** | **ILE** | **0.605** | |
| **244** | **GLN** | **0.601** | |
| **245** | **LYS** | **0.606** | |
| **246** | **TYR** | **0.623** | |
| **247** | **GLN** | **0.638** | |
| **248** | **LEU** | **0.652** | |
| **249** | **ASP** | **0.662** | |
| **250** | **ILE** | **0.668** | |
| **251** | **ARG** | **0.668** | |
| **252** | **LYS** | **0.678** | |
| **253** | **GLN** | **0.671** | |
| **254** | **ALA** | **0.664** | |
| **255** | **LEU** | **0.659** | |
| **256** | **ARG** | **0.645** | |
| **257** | **GLN** | **0.632** | |
| **258** | **SER** | **0.62** | |
| **259** | **VAL** | **0.606** | |
| **260** | **VAL** | **0.58** | |
| **261** | **GLU** | **0.578** | |
| **262** | **SER** | **0.582** | |
| **263** | **GLY** | **0.578** | |
| **264** | **LEU** | **0.568** | |
| **265** | **ASN** | **0.557** | |
| **266** | **VAL** | **0.538** | |
| **267** | **GLN** | **0.524** | |
| **277** | **ASN** | **0.504** | |
| **278** | **PRO** | **0.511** | |
| **279** | **GLN** | **0.549** | |
| **280** | **TYR** | **0.561** | |
| **281** | **ALA** | **0.559** | |
| **282** | **LEU** | **0.56** | |
| **283** | **TYR** | **0.546** | |
| **284** | **GLN** | **0.527** | |
| **285** | **PRO** | **0.513** | |
| **286** | **ASP** | **0.51** | |
| **287** | **GLY** | **0.517** | |
| **311** | **VAL** | **0.518** | |
| **312** | **SER** | **0.526** | |
| **313** | **ASN** | **0.547** | |
| **314** | **GLY** | **0.556** | |
| **315** | **GLN** | **0.577** | |
| **316** | **GLU** | **0.577** | |
| **317** | **THR** | **0.558** | |
| **318** | **TRP** | **0.546** | |
| **319** | **GLU** | **0.55** | |
| **320** | **SER** | **0.53** | |
| **321** | **MET** | **0.501** | |
| **322** | **PHE** | **0.506** | |
| **323** | **ASP** | **0.513** | |
| **324** | **ASP** | **0.512** | |
| **325** | **LEU** | **0.521** | |
| **326** | **GLN** | **0.523** | |
| **327** | **ASP** | **0.52** | |
| **336** | **ILE** | **0.505** | |
| **337** | **THR** | **0.533** | |
| **338** | **VAL** | **0.554** | |
| **339** | **SER** | **0.575** | |
| **340** | **GLN** | **0.583** | |
| **341** | **GLN** | **0.584** | |
| **342** | **ARG** | **0.574** | |
| **343** | **LYS** | **0.574** | |
| **344** | **ASN** | **0.566** | |
| **345** | **LEU** | **0.558** | |
| **346** | **ALA** | **0.557** | |
| **347** | **TYR** | **0.552** | |
| **348** | **PHE** | **0.551** | |
| **349** | **SER** | **0.552** | |
| **350** | **GLU** | **0.548** | |
| **351** | **SER** | **0.537** | |
| **352** | **TYR** | **0.527** | |
| **353** | **TYR** | **0.513** | |
| **364** | **HIS** | **0.51** | |
| **365** | **TYR** | **0.54** | |
| **366** | **LYS** | **0.569** | |
| **367** | **ASP** | **0.591** | |
| **368** | **ASP** | **0.586** | |
| **369** | **VAL** | **0.585** | |
| **370** | **TYR** | **0.586** | |
| **371** | **SER** | **0.585** | |
| **372** | **ASN** | **0.564** | |
| **373** | **VAL** | **0.561** | |
| **374** | **SER** | **0.557** | |
| **375** | **GLU** | **0.55** | |
| **376** | **LEU** | **0.533** | |
| **377** | **VAL** | **0.52** | |
| **378** | **ALA** | **0.509** | |
| **387** | **ASP** | **0.513** | |
| **388** | **PHE** | **0.533** | |
| **389** | **PHE** | **0.527** | |
| **390** | **GLU** | **0.533** | |
| **391** | **GLU** | **0.534** | |
| **392** | **LEU** | **0.53** | |
| **393** | **LEU** | **0.525** | |
| **394** | **GLN** | **0.53** | |
| **395** | **GLN** | **0.532** | |
| **396** | **MET** | **0.534** | |
| **397** | **LEU** | **0.53** | |
| **398** | **PRO** | **0.546** | |
| **399** | **ASN** | **0.534** | |
| **400** | **LYS** | **0.536** | |
| **401** | **ILE** | **0.526** | |
| **402** | **LEU** | **0.524** | |
| **403** | **PHE** | **0.524** | |
| **404** | **SER** | **0.534** | |
| **405** | **TYR** | **0.54** | |
| **406** | **ALA** | **0.542** | |
| **407** | **SER** | **0.548** | |
| **408** | **GLN** | **0.54** | |
| **409** | **GLU** | **0.551** | |
| **410** | **GLU** | **0.55** | |
| **411** | **LYS** | **0.526** | |
| **412** | **VAL** | **0.517** | |
| **413** | **GLN** | **0.515** | |
| **414** | **ALA** | **0.51** | |
| **441** | **LEU** | **0.514** | |
| **442** | **PRO** | **0.544** | |
| **443** | **ILE** | **0.541** | |
| **444** | **VAL** | **0.542** | |
| **445** | **GLU** | **0.533** | |
| **446** | **ASP** | **0.549** | |
| **447** | **THR** | **0.55** | |
| **448** | **MET** | **0.556** | |
| **449** | **ILE** | **0.558** | |
| **450** | **GLY** | **0.555** | |
| **451** | **SER** | **0.546** | |
| **452** | **PHE** | **0.546** | |
| **453** | **TYR** | **0.544** | |
| **454** | **GLN** | **0.555** | |
| **455** | **TYR** | **0.555** | |
| **456** | **ASP** | **0.562** | |
| **457** | **ILE** | **0.564** | |
| **458** | **ALA** | **0.57** | |
| **459** | **ILE** | **0.575** | |
| **460** | **GLY** | **0.583** | |
| **461** | **PHE** | **0.587** | |
| **462** | **ALA** | **0.591** | |
| **463** | **LYS** | **0.581** | |
| **464** | **ASN** | **0.566** | |
| **465** | **PRO** | **0.552** | |
| **466** | **LEU** | **0.525** | |
| **467** | **GLY** | **0.511** | |
| **468** | **ALA** | **0.502** | |
| **474** | **PHE** | **0.505** | |
| **475** | **SER** | **0.529** | |
| **476** | **ARG** | **0.535** | |
| **477** | **ALA** | **0.54** | |
| **478** | **ILE** | **0.537** | |
| **479** | **LYS** | **0.55** | |
| **480** | **MET** | **0.544** | |
| **481** | **LEU** | **0.538** | |
| **482** | **ASN** | **0.54** | |
| **483** | **THR** | **0.528** | |
| **484** | **GLU** | **0.525** | |
| **485** | **GLN** | **0.523** | |
| **486** | **ILE** | **0.54** | |
| **487** | **ILE** | **0.545** | |
| **488** | **HIS** | **0.55** | |
| **489** | **THR** | **0.557** | |
| **490** | **TYR** | **0.554** | |
| **491** | **ASP** | **0.548** | |
| **492** | **TYR** | **0.551** | |
| **493** | **GLN** | **0.538** | |
| **494** | **PRO** | **0.524** | |
| **504** | **LYS** | **0.526** | |
| **505** | **LYS** | **0.531** | |
| **506** | **TYR** | **0.529** | |
| **507** | **GLN** | **0.519** | |
| **508** | **ARG** | **0.501** | |
| **537** | **LEU** | **0.5** | |
| **540** | **LEU** | **0.505** | |
| **551** | **ARG** | **0.506** | |
| **552** | **ARG** | **0.509** | |
| **553** | **GLY** | **0.5** | |
| **569** | **LYS** | **0.54** | |
| **570** | **SER** | **0.559** | |
| **571** | **ILE** | **0.557** | |
| **572** | **ASN** | **0.557** | |
| **573** | **ASP** | **0.567** | |
| **574** | **GLN** | **0.56** | |
| **575** | **TYR** | **0.56** | |
| **576** | **GLY** | **0.561** | |
| **577** | **HIS** | **0.543** | |
| **578** | **GLU** | **0.522** | |
| **594** | **VAL** | **0.503** | |
| **595** | **TRP** | **0.508** | |
| **596** | **ARG** | **0.515** | |
| **597** | **GLY** | **0.508** | |
| **598** | **ARG** | **0.504** | |
| **616** | **LYS** | **0.512** | |
| **626** | **GLU** | **0.501** | |
| **627** | **ARG** | **0.509** | |
| **628** | **PHE** | **0.515** | |
| **629** | **MET** | **0.523** | |
| **630** | **PHE** | **0.544** | |
| **631** | **VAL** | **0.54** | |
| **632** | **ASP** | **0.536** | |
| **633** | **ALA** | **0.532** | |
| **634** | **GLU** | **0.521** | |
| **654** | **THR** | **0.526** | |
| **655** | **GLU** | **0.552** | |
| **656** | **SER** | **0.533** | |
| **657** | **LEU** | **0.518** | |
| **658** | **ASN** | **0.506** | |
| **676** | **THR** | **0.501** | |
| **687** | **LYS** | **0.518** | |
| **688** | **GLY** | **0.506** | |
| **UniProt ID- Q9KKL8** | | | |
| **24** | **ALA** | **0.509** | |
| **25** | **LYS** | **0.505** | |
| **26** | **GLU** | **0.506** | |
| **27** | **LEU** | **0.516** | |
| **28** | **ASN** | **0.503** | |
| **29** | **ARG** | **0.513** | |
| **30** | **SER** | **0.503** | |
| **31** | **GLU** | **0.502** | |
| **41** | **ALA** | **0.514** | |
| **42** | **GLY** | **0.517** | |
| **43** | **LYS** | **0.513** | |
| **44** | **LEU** | **0.508** | |
| **45** | **PHE** | **0.508** | |
| **46** | **ILE** | **0.531** | |
| **47** | **LEU** | **0.533** | |
| **48** | **ASP** | **0.538** | |
| **49** | **GLU** | **0.55** | |
| **50** | **GLU** | **0.539** | |
| **51** | **VAL** | **0.517** | |
| **52** | **THR** | **0.525** | |
| **53** | **ARG** | **0.519** | |
| **54** | **PHE** | **0.502** | |
| **59** | **LYS** | **0.523** | |
| **60** | **GLU** | **0.567** | |
| **61** | **SER** | **0.57** | |
| **62** | **LYS** | **0.583** | |
| **63** | **ARG** | **0.601** | |
| **64** | **ALA** | **0.603** | |
| **65** | **ALA** | **0.59** | |
| **66** | **PHE** | **0.586** | |
| **67** | **GLU** | **0.568** | |
| **68** | **GLY** | **0.552** | |
| **69** | **ILE** | **0.535** | |
| **70** | **VAL** | **0.518** | |
| **71** | **ILE** | **0.519** | |
| **72** | **ALA** | **0.504** | |
| **98** | **TYR** | **0.505** | |
| **99** | **GLY** | **0.534** | |
| **100** | **GLY** | **0.553** | |
| **101** | **ARG** | **0.554** | |
| **102** | **GLY** | **0.565** | |
| **103** | **LEU** | **0.562** | |
| **104** | **ARG** | **0.536** | |
| **105** | **GLY** | **0.539** | |
| **106** | **ASN** | **0.529** | |
| **107** | **GLU** | **0.513** | |
| **120** | **GLY** | **0.511** | |
| **121** | **ILE** | **0.521** | |
| **122** | **ASN** | **0.518** | |
| **123** | **LYS** | **0.524** | |
| **124** | **LEU** | **0.519** | |
| **134** | **GLY** | **0.511** | |
| **135** | **GLN** | **0.545** | |
| **136** | **LEU** | **0.545** | |
| **137** | **LYS** | **0.557** | |
| **138** | **PRO** | **0.57** | |
| **139** | **GLY** | **0.569** | |
| **140** | **GLU** | **0.541** | |
| **141** | **VAL** | **0.531** | |
| **150** | **LYS** | **0.501** | |
| **151** | **GLU** | **0.531** | |
| **152** | **PRO** | **0.558** | |
| **153** | **ILE** | **0.567** | |
| **154** | **GLN** | **0.563** | |
| **155** | **GLY** | **0.548** | |
| **156** | **ARG** | **0.519** | |
| **157** | **ASP** | **0.501** | |
| **165** | **GLU** | **0.535** | |
| **166** | **ASP** | **0.567** | |
| **167** | **VAL** | **0.583** | |
| **168** | **SER** | **0.613** | |
| **169** | **LYS** | **0.645** | |
| **170** | **ARG** | **0.656** | |
| **171** | **VAL** | **0.675** | |
| **172** | **LEU** | **0.684** | |
| **173** | **ARG** | **0.676** | |
| **174** | **PRO** | **0.671** | |
| **175** | **GLN** | **0.653** | |
| **176** | **GLN** | **0.643** | |
| **177** | **LYS** | **0.623** | |
| **178** | **ALA** | **0.62** | |
| **179** | **GLY** | **0.59** | |
| **180** | **GLU** | **0.569** | |
| **181** | **ASN** | **0.541** | |
| **182** | **LYS** | **0.54** | |
| **183** | **LEU** | **0.531** | |
| **184** | **ASP** | **0.535** | |
| **185** | **MET** | **0.545** | |
| **186** | **ARG** | **0.554** | |
| **187** | **ASN** | **0.546** | |
| **188** | **LEU** | **0.555** | |
| **189** | **GLY** | **0.549** | |
| **190** | **GLU** | **0.535** | |
| **191** | **THR** | **0.515** | |
| **192** | **ILE** | **0.513** | |
| **193** | **THR** | **0.506** | |
| **203** | **ARG** | **0.501** | |
| **204** | **LEU** | **0.515** | |
| **205** | **PRO** | **0.534** | |
| **206** | **ALA** | **0.578** | |
| **207** | **THR** | **0.59** | |
| **208** | **LYS** | **0.6** | |
| **209** | **GLY** | **0.601** | |
| **210** | **GLU** | **0.582** | |
| **211** | **MET** | **0.569** | |
| **212** | **GLY** | **0.56** | |
| **213** | **TYR** | **0.548** | |
| **214** | **THR** | **0.54** | |
| **215** | **VAL** | **0.517** | |
| **219** | **PRO** | **0.503** | |
| **220** | **ILE** | **0.511** | |
| **221** | **PRO** | **0.513** | |
| **222** | **PRO** | **0.527** | |
| **223** | **LYS** | **0.531** | |
| **224** | **PRO** | **0.533** | |
| **225** | **GLY** | **0.544** | |
| **226** | **LYS** | **0.528** | |
| **227** | **GLU** | **0.526** | |
| **228** | **SER** | **0.514** | |
| **229** | **ALA** | **0.51** | |
| **230** | **LEU** | **0.508** | |
| **271** | **HIS** | **0.504** | |
| **272** | **VAL** | **0.512** | |
| **273** | **SER** | **0.513** | |
| **274** | **VAL** | **0.523** | |
| **275** | **ALA** | **0.506** | |
| **330** | **MET** | **0.506** | |
| **331** | **VAL** | **0.531** | |
| **332** | **THR** | **0.547** | |
| **333** | **ASP** | **0.565** | |
| **334** | **ASP** | **0.58** | |
| **335** | **GLU** | **0.571** | |
| **336** | **GLU** | **0.558** | |
| **337** | **ARG** | **0.523** | |
| **338** | **SER** | **0.505** | |
| **385** | **SER** | **0.504** | |
| **387** | **MET** | **0.52** | |
| **388** | **THR** | **0.507** | |
| **410** | **VAL** | **0.511** | |
| **411** | **GLU** | **0.522** | |
| **412** | **GLY** | **0.502** | |
| **425** | **GLN** | **0.517** | |
| **426** | **SER** | **0.538** | |
| **427** | **TYR** | **0.546** | |
| **428** | **LYS** | **0.562** | |
| **429** | **GLU** | **0.572** | |
| **430** | **ARG** | **0.573** | |
| **431** | **ILE** | **0.574** | |
| **432** | **ASN** | **0.572** | |
| **433** | **LYS** | **0.572** | |
| **434** | **HIS** | **0.582** | |
| **435** | **LYS** | **0.589** | |
| **436** | **GLU** | **0.593** | |
| **437** | **LEU** | **0.571** | |
| **438** | **TYR** | **0.57** | |
| **439** | **LYS** | **0.561** | |
| **440** | **GLN** | **0.554** | |
| **441** | **ALA** | **0.551** | |
| **442** | **GLN** | **0.545** | |
| **443** | **ASP** | **0.532** | |
| **444** | **THR** | **0.507** | |
| **445** | **THR** | **0.503** | |
| **446** | **MET** | **0.524** | |
| **447** | **ALA** | **0.517** | |
| **448** | **LEU** | **0.518** | |
| **449** | **ILE** | **0.515** | |
| **450** | **ARG** | **0.521** | |
| **451** | **ARG** | **0.5** | |
| **452** | **GLU** | **0.511** | |
| **453** | **ILE** | **0.526** | |
| **454** | **GLU** | **0.533** | |
| **455** | **PHE** | **0.522** | |
| **456** | **LYS** | **0.527** | |
| **457** | **LYS** | **0.535** | |
| **458** | **ARG** | **0.555** | |
| **459** | **PRO** | **0.561** | |
| **460** | **LYS** | **0.578** | |
| **461** | **ALA** | **0.577** | |
| **462** | **GLU** | **0.583** | |
| **463** | **ARG** | **0.588** | |
| **464** | **SER** | **0.589** | |
| **465** | **ASP** | **0.587** | |
| **466** | **GLU** | **0.585** | |
| **467** | **GLU** | **0.555** | |
| **468** | **ALA** | **0.555** | |
| **469** | **GLN** | **0.548** | |
| **470** | **GLU** | **0.539** | |
| **471** | **ILE** | **0.522** | |
| **472** | **GLU** | **0.509** | |
| **473** | **THR** | **0.508** | |
| **486** | **LYS** | **0.506** | |
| **487** | **LEU** | **0.534** | |
| **488** | **ALA** | **0.513** | |
| **489** | **LEU** | **0.501** | |
| **490** | **ASP** | **0.516** | |
| **491** | **MET** | **0.517** | |
| **492** | **LEU** | **0.504** | |
| **493** | **ASN** | **0.519** | |
| **494** | **GLU** | **0.53** | |
| **495** | **GLU** | **0.527** | |
| **496** | **PHE** | **0.524** | |
| **497** | **GLU** | **0.543** | |
| **498** | **GLN** | **0.545** | |
| **499** | **GLN** | **0.538** | |
| **500** | **LEU** | **0.52** | |
| **501** | **LEU** | **0.515** | |
| **525** | **LEU** | **0.515** | |
| **526** | **THR** | **0.527** | |
| **527** | **LYS** | **0.533** | |
| **528** | **ARG** | **0.537** | |
| **529** | **GLU** | **0.532** | |
| **530** | **HIS** | **0.532** | |
| **531** | **GLY** | **0.532** | |
| **532** | **ALA** | **0.52** | |
| **545** | **SER** | **0.5** | |
| **546** | **SER** | **0.5** | |
| **547** | **LEU** | **0.537** | |
| **548** | **LEU** | **0.554** | |
| **549** | **GLU** | **0.564** | |
| **550** | **ALA** | **0.58** | |
| **551** | **GLU** | **0.566** | |
| **552** | **ASP** | **0.503** | |
| **UniProt ID- Q9KLK5** | | | |
| **23** | **VAL** | **0.517** | |
| **24** | **GLY** | **0.544** | |
| **25** | **ASN** | **0.551** | |
| **26** | **GLY** | **0.534** | |
| **27** | **MET** | **0.508** | |
| **54** | **MET** | **0.527** | |
| **55** | **ASP** | **0.543** | |
| **56** | **ALA** | **0.538** | |
| **57** | **ARG** | **0.542** | |
| **58** | **GLY** | **0.549** | |
| **59** | **ALA** | **0.538** | |
| **60** | **GLY** | **0.519** | |
| **61** | **MET** | **0.524** | |
| **86** | **ARG** | **0.503** | |
| **87** | **LYS** | **0.505** | |
| **102** | **ALA** | **0.503** | |
| **103** | **GLU** | **0.514** | |
| **104** | **ASP** | **0.531** | |
| **105** | **LYS** | **0.544** | |
| **106** | **ASP** | **0.543** | |
| **107** | **GLU** | **0.551** | |
| **108** | **SER** | **0.539** | |
| **109** | **LEU** | **0.54** | |
| **110** | **LYS** | **0.53** | |
| **111** | **THR** | **0.513** | |
| **112** | **ILE** | **0.513** | |
| **113** | **ASP** | **0.513** | |
| **117** | **ASP** | **0.501** | |
| **120** | **GLU** | **0.533** | |
| **121** | **GLN** | **0.536** | |
| **122** | **PHE** | **0.551** | |
| **123** | **GLU** | **0.584** | |
| **124** | **ARG** | **0.607** | |
| **125** | **ALA** | **0.608** | |
| **126** | **GLY** | **0.61** | |
| **127** | **VAL** | **0.634** | |
| **128** | **GLY** | **0.621** | |
| **129** | **ALA** | **0.607** | |
| **130** | **ALA** | **0.613** | |
| **131** | **THR** | **0.605** | |
| **132** | **GLN** | **0.596** | |
| **133** | **GLU** | **0.596** | |
| **134** | **ASN** | **0.579** | |
| **135** | **VAL** | **0.58** | |
| **136** | **ASP** | **0.582** | |
| **137** | **GLN** | **0.579** | |
| **138** | **LEU** | **0.548** | |
| **139** | **ASN** | **0.549** | |
| **140** | **ARG** | **0.552** | |
| **141** | **TYR** | **0.536** | |
| **142** | **LEU** | **0.534** | |
| **143** | **ASP** | **0.547** | |
| **144** | **GLN** | **0.544** | |
| **145** | **LEU** | **0.532** | |
| **146** | **ALA** | **0.515** | |
| **147** | **ASP** | **0.516** | |
| **183** | **LYS** | **0.505** | |
| **184** | **ALA** | **0.526** | |
| **185** | **ASN** | **0.557** | |
| **186** | **VAL** | **0.578** | |
| **187** | **ALA** | **0.594** | |
| **188** | **ALA** | **0.606** | |
| **189** | **LYS** | **0.612** | |
| **190** | **SER** | **0.606** | |
| **191** | **GLY** | **0.625** | |
| **192** | **ASN** | **0.62** | |
| **193** | **SER** | **0.61** | |
| **194** | **ALA** | **0.611** | |
| **195** | **ASN** | **0.62** | |
| **196** | **GLU** | **0.636** | |
| **197** | **VAL** | **0.641** | |
| **198** | **LYS** | **0.653** | |
| **199** | **THR** | **0.662** | |
| **200** | **ARG** | **0.653** | |
| **201** | **TYR** | **0.649** | |
| **202** | **GLU** | **0.632** | |
| **203** | **SER** | **0.606** | |
| **204** | **SER** | **0.557** | |
| **205** | **ASP** | **0.523** | |
| **239** | **VAL** | **0.506** | |
| **240** | **GLN** | **0.515** | |
| **241** | **GLN** | **0.523** | |
| **242** | **LEU** | **0.531** | |
| **243** | **ARG** | **0.532** | |
| **244** | **THR** | **0.527** | |
| **245** | **TYR** | **0.522** | |
| **246** | **GLN** | **0.515** | |
| **247** | **ASP** | **0.508** | |
| **248** | **ASN** | **0.511** | |
| **249** | **ALA** | **0.524** | |
| **250** | **SER** | **0.532** | |
| **251** | **VAL** | **0.536** | |
| **252** | **LYS** | **0.542** | |
| **253** | **SER** | **0.553** | |
| **254** | **PHE** | **0.551** | |
| **255** | **ASP** | **0.56** | |
| **256** | **LEU** | **0.585** | |
| **257** | **ASP** | **0.584** | |
| **258** | **ASP** | **0.584** | |
| **259** | **TYR** | **0.596** | |
| **260** | **ASP** | **0.608** | |
| **261** | **LYS** | **0.615** | |
| **262** | **SER** | **0.608** | |
| **263** | **GLU** | **0.604** | |
| **264** | **VAL** | **0.591** | |
| **265** | **LYS** | **0.559** | |
| **266** | **ASP** | **0.534** | |
| **292** | **PHE** | **0.514** | |
| **293** | **ALA** | **0.54** | |
| **294** | **LYS** | **0.535** | |
| **295** | **ASP** | **0.549** | |
| **296** | **ILE** | **0.555** | |
| **297** | **GLN** | **0.553** | |
| **298** | **THR** | **0.551** | |
| **299** | **GLN** | **0.552** | |
| **300** | **ASN** | **0.535** | |
| **301** | **ARG** | **0.532** | |
| **302** | **ASN** | **0.525** | |
| **303** | **ASN** | **0.51** | |
| **331** | **THR** | **0.51** | |
| **332** | **LYS** | **0.525** | |
| **333** | **GLN** | **0.565** | |
| **334** | **ARG** | **0.581** | |
| **335** | **ARG** | **0.613** | |
| **336** | **PHE** | **0.621** | |
| **337** | **ASN** | **0.62** | |
| **338** | **GLY** | **0.612** | |
| **339** | **ASP** | **0.582** | |
| **340** | **ASN** | **0.566** | |
| **341** | **ASP** | **0.542** | |
| **355** | **TRP** | **0.504** | |
| **356** | **GLY** | **0.509** | |
| **357** | **TRP** | **0.51** | |
| **365** | **GLU** | **0.507** | |
| **366** | **VAL** | **0.524** | |
| **367** | **ASP** | **0.553** | |
| **368** | **LEU** | **0.59** | |
| **369** | **GLN** | **0.606** | |
| **370** | **ASN** | **0.617** | |
| **371** | **SER** | **0.605** | |
| **372** | **LEU** | **0.589** | |
| **373** | **ASP** | **0.552** | |
| **397** | **ALA** | **0.508** | |
| **398** | **GLY** | **0.529** | |
| **399** | **ASP** | **0.533** | |
| **400** | **ASN** | **0.541** | |
| **401** | **GLN** | **0.524** | |
| **402** | **PHE** | **0.51** | |
| **UniProt ID_ Q9KU75** | | | |
| **23** | **GLU** | **0.524** | |
| **24** | **SER** | **0.56** | |
| **25** | **ALA** | **0.559** | |
| **26** | **THR** | **0.54** | |
| **27** | **GLN** | **0.512** | |
| **42** | **GLN** | **0.521** | |
| **43** | **GLN** | **0.529** | |
| **44** | **GLU** | **0.536** | |
| **45** | **VAL** | **0.534** | |
| **46** | **GLN** | **0.506** | |
| **47** | **ILE** | **0.501** | |
| **48** | **ALA** | **0.509** | |
| **56** | **ARG** | **0.514** | |
| **57** | **THR** | **0.519** | |
| **58** | **ASP** | **0.544** | |
| **59** | **LEU** | **0.569** | |
| **60** | **GLU** | **0.573** | |
| **61** | **PRO** | **0.556** | |
| **62** | **GLU** | **0.556** | |
| **63** | **ILE** | **0.526** | |
| **75** | **TYR** | **0.5** | |
| **76** | **ASP** | **0.514** | |
| **77** | **SER** | **0.529** | |
| **78** | **VAL** | **0.53** | |
| **79** | **GLY** | **0.539** | |
| **80** | **LEU** | **0.531** | |
| **81** | **ARG** | **0.52** | |
| **82** | **ASP** | **0.507** | |
| **93** | **SER** | **0.517** | |
| **94** | **ILE** | **0.515** | |
| **95** | **ASN** | **0.51** | |
| **96** | **PRO** | **0.525** | |
| **97** | **ALA** | **0.518** | |
| **113** | **GLY** | **0.516** | |
| **114** | **GLU** | **0.517** | |
| **115** | **PHE** | **0.525** | |
| **116** | **ASP** | **0.517** | |
| **117** | **ALA** | **0.517** | |
| **120** | **GLU** | **0.505** | |
| **125** | **THR** | **0.505** | |
| **126** | **LEU** | **0.508** | |
| **127** | **GLU** | **0.527** | |
| **128** | **LEU** | **0.503** | |
| **129** | **ASP** | **0.503** | |
| **130** | **PRO** | **0.518** | |
| **131** | **SER** | **0.508** | |
| **147** | **GLU** | **0.525** | |
| **148** | **ARG** | **0.528** | |
| **149** | **PRO** | **0.541** | |
| **150** | **GLN** | **0.536** | |
| **151** | **LEU** | **0.539** | |
| **152** | **ALA** | **0.516** | |
| **153** | **LEU** | **0.512** | |
| **154** | **GLU** | **0.521** | |
| **157** | **THR** | **0.506** | |
| **158** | **LYS** | **0.503** | |
| **159** | **HIS** | **0.508** | |
| **160** | **TYR** | **0.509** | |
| **161** | **GLN** | **0.531** | |
| **162** | **GLN** | **0.52** | |
| **163** | **ASP** | **0.512** | |
| **164** | **PRO** | **0.528** | |
| **165** | **GLN** | **0.523** | |
| **181** | **ASP** | **0.517** | |
| **182** | **PRO** | **0.525** | |
| **183** | **THR** | **0.544** | |
| **184** | **LYS** | **0.549** | |
| **185** | **ALA** | **0.532** | |
| **186** | **ARG** | **0.522** | |
| **187** | **THR** | **0.53** | |
| **188** | **GLU** | **0.534** | |
| **189** | **LEU** | **0.52** | |
| **190** | **LEU** | **0.526** | |
| **191** | **GLU** | **0.538** | |
| **192** | **ARG** | **0.542** | |
| **193** | **TYR** | **0.542** | |
| **194** | **GLN** | **0.567** | |
| **195** | **GLN** | **0.579** | |
| **196** | **HIS** | **0.575** | |
| **197** | **ASP** | **0.572** | |
| **198** | **GLU** | **0.58** | |
| **199** | **GLN** | **0.542** | |
| **200** | **TRP** | **0.512** | |
| **215** | **GLU** | **0.506** | |
| **216** | **VAL** | **0.521** | |
| **217** | **ALA** | **0.511** | |
| **218** | **PHE** | **0.508** | |
| **219** | **LYS** | **0.503** | |
| **227** | **GLU** | **0.5** | |
| **229** | **GLU** | **0.519** | |
| **230** | **LEU** | **0.548** | |
| **231** | **LEU** | **0.54** | |
| **232** | **ALA** | **0.536** | |
| **233** | **GLN** | **0.542** | |
| **234** | **ARG** | **0.531** | |
| **235** | **LEU** | **0.512** | |
| **250** | **GLY** | **0.521** | |
| **251** | **GLU** | **0.511** | |
| **252** | **LEU** | **0.521** | |
| **253** | **ALA** | **0.525** | |
| **254** | **HIS** | **0.526** | |
| **255** | **ALA** | **0.505** | |
| **256** | **LEU** | **0.509** | |
| **257** | **SER** | **0.509** | |
| **265** | **PHE** | **0.502** | |
| **266** | **ASN** | **0.511** | |
| **267** | **VAL** | **0.541** | |
| **268** | **TYR** | **0.55** | |
| **269** | **GLU** | **0.541** | |
| **270** | **TYR** | **0.545** | |
| **271** | **VAL** | **0.543** | |
| **272** | **GLU** | **0.51** | |
| **287** | **ALA** | **0.51** | |
| **288** | **ARG** | **0.528** | |
| **289** | **GLU** | **0.524** | |
| **290** | **GLU** | **0.521** | |
| **291** | **GLN** | **0.518** | |
| **292** | **LEU** | **0.513** | |
| **UniProt ID- Q9KND9** | | | |
| **38** | **SER** | **0.53** | |
| **39** | **SER** | **0.556** | |
| **40** | **PRO** | **0.591** | |
| **41** | **LYS** | **0.604** | |
| **42** | **LEU** | **0.603** | |
| **43** | **SER** | **0.595** | |
| **44** | **GLY** | **0.602** | |
| **45** | **GLY** | **0.597** | |
| **46** | **PHE** | **0.583** | |
| **47** | **PHE** | **0.574** | |
| **48** | **ASP** | **0.566** | |
| **49** | **ALA** | **0.534** | |
| **50** | **THR** | **0.521** | |
| **51** | **LYS** | **0.509** | |
| **52** | **LYS** | **0.514** | |
| **53** | **LEU** | **0.507** | |
| **54** | **GLU** | **0.503** | |
| **55** | **LEU** | **0.501** | |
| **70** | **PHE** | **0.505** | |
| **71** | **SER** | **0.525** | |
| **72** | **GLN** | **0.542** | |
| **73** | **GLY** | **0.537** | |
| **74** | **ASN** | **0.531** | |
| **75** | **ASP** | **0.534** | |
| **76** | **ARG** | **0.526** | |
| **77** | **ILE** | **0.509** | |
| **78** | **ILE** | **0.501** | |
| **99** | **MET** | **0.504** | |
| **100** | **PRO** | **0.524** | |
| **101** | **LYS** | **0.527** | |
| **102** | **TYR** | **0.561** | |
| **103** | **ARG** | **0.572** | |
| **104** | **ASP** | **0.576** | |
| **105** | **ALA** | **0.564** | |
| **106** | **PRO** | **0.548** | |
| **107** | **GLN** | **0.523** | |
| **108** | **ALA** | **0.507** | |
| **109** | **THR** | **0.5** | |
| **122** | **GLN** | **0.501** | |
| **124** | **GLY** | **0.515** | |
| **125** | **LYS** | **0.523** | |
| **126** | **ALA** | **0.531** | |
| **127** | **VAL** | **0.525** | |
| **128** | **GLU** | **0.527** | |
| **129** | **LEU** | **0.53** | |
| **130** | **ARG** | **0.514** | |
| **131** | **GLN** | **0.523** | |
| **132** | **ASP** | **0.548** | |
| **133** | **ARG** | **0.559** | |
| **134** | **LEU** | **0.567** | |
| **135** | **ILE** | **0.564** | |
| **136** | **LYS** | **0.584** | |
| **137** | **GLU** | **0.578** | |
| **138** | **GLY** | **0.577** | |
| **139** | **MET** | **0.597** | |
| **140** | **GLN** | **0.591** | |
| **141** | **ILE** | **0.58** | |
| **142** | **GLY** | **0.58** | |
| **143** | **ARG** | **0.571** | |
| **144** | **ASN** | **0.58** | |
| **145** | **PHE** | **0.549** | |
| **146** | **GLU** | **0.553** | |
| **147** | **PHE** | **0.551** | |
| **148** | **GLU** | **0.534** | |
| **149** | **THR** | **0.526** | |
| **150** | **ALA** | **0.537** | |
| **151** | **GLU** | **0.543** | |
| **152** | **TYR** | **0.557** | |
| **153** | **ASN** | **0.552** | |
| **154** | **LYS** | **0.57** | |
| **155** | **LYS** | **0.573** | |
| **156** | **GLY** | **0.571** | |
| **157** | **GLY** | **0.571** | |
| **158** | **VAL** | **0.577** | |
| **159** | **ALA** | **0.57** | |
| **160** | **ALA** | **0.57** | |
| **161** | **LEU** | **0.576** | |
| **162** | **THR** | **0.591** | |
| **163** | **SER** | **0.594** | |
| **164** | **SER** | **0.605** | |
| **165** | **MET** | **0.619** | |
| **166** | **ALA** | **0.638** | |
| **167** | **VAL** | **0.645** | |
| **168** | **GLN** | **0.647** | |
| **169** | **PRO** | **0.657** | |
| **170** | **ILE** | **0.658** | |
| **171** | **ALA** | **0.654** | |
| **172** | **GLN** | **0.662** | |
| **173** | **GLN** | **0.66** | |
| **174** | **GLU** | **0.649** | |
| **175** | **ILE** | **0.651** | |
| **176** | **SER** | **0.646** | |
| **177** | **ASN** | **0.638** | |
| **178** | **ALA** | **0.619** | |
| **179** | **THR** | **0.602** | |
| **180** | **ALA** | **0.569** | |
| **181** | **MET** | **0.546** | |
| **182** | **ALA** | **0.529** | |
| **183** | **ALA** | **0.528** | |
| **184** | **ALA** | **0.502** | |
| **UniProt ID- Q9KVJ9** | | | |
| **20** | **GLU** | **0.521** | |
| **21** | **ASN** | **0.542** | |
| **22** | **GLN** | **0.546** | |
| **23** | **LYS** | **0.549** | |
| **24** | **LEU** | **0.547** | |
| **25** | **LYS** | **0.519** | |
| **44** | **GLU** | **0.534** | |
| **45** | **GLN** | **0.577** | |
| **46** | **HIS** | **0.609** | |
| **47** | **SER** | **0.625** | |
| **48** | **HIS** | **0.622** | |
| **49** | **ALA** | **0.613** | |
| **50** | **THR** | **0.6** | |
| **51** | **PRO** | **0.571** | |
| **52** | **GLU** | **0.533** | |
| **71** | **LEU** | **0.512** | |
| **72** | **ASN** | **0.519** | |
| **73** | **HIS** | **0.531** | |
| **74** | **ARG** | **0.534** | |
| **75** | **TYR** | **0.514** | |
| **95** | **TRP** | **0.516** | |
| **96** | **VAL** | **0.548** | |
| **97** | **GLN** | **0.577** | |
| **98** | **LEU** | **0.597** | |
| **99** | **SER** | **0.601** | |
| **100** | **GLY** | **0.603** | |
| **101** | **SER** | **0.594** | |
| **102** | **MET** | **0.56** | |
| **103** | **VAL** | **0.531** | |
| **125** | **ILE** | **0.507** | |
| **126** | **ASP** | **0.513** | |
| **127** | **THR** | **0.507** | |
| **148** | **GLU** | **0.526** | |
| **149** | **LEU** | **0.547** | |
| **150** | **TRP** | **0.571** | |
| **151** | **LEU** | **0.607** | |
| **152** | **GLN** | **0.621** | |
| **153** | **GLU** | **0.62** | |
| **154** | **ALA** | **0.605** | |
| **155** | **THR** | **0.59** | |
| **156** | **VAL** | **0.56** | |
| **157** | **ALA** | **0.522** | |
| **180** | **TYR** | **0.501** | |
| **181** | **ARG** | **0.539** | |
| **182** | **ARG** | **0.563** | |
| **183** | **PRO** | **0.568** | |
| **184** | **LEU** | **0.575** | |
| **185** | **PRO** | **0.573** | |
| **186** | **TRP** | **0.551** | |
| **187** | **GLY** | **0.505** | |
| **UniProt ID- Q9KSV6** | | | |
| **14** | **LEU** | **0.526** | |
| **15** | **ASN** | **0.551** | |
| **16** | **GLU** | **0.578** | |
| **17** | **VAL** | **0.594** | |
| **18** | **PRO** | **0.615** | |
| **19** | **SER** | **0.65** | |
| **20** | **ARG** | **0.662** | |
| **21** | **THR** | **0.679** | |
| **22** | **THR** | **0.689** | |
| **23** | **LYS** | **0.68** | |
| **24** | **ASN** | **0.677** | |
| **25** | **ASN** | **0.653** | |
| **26** | **ASN** | **0.647** | |
| **27** | **SER** | **0.62** | |
| **28** | **LYS** | **0.573** | |
| **29** | **GLY** | **0.544** | |
| **30** | **LYS** | **0.515** | |
| **54** | **CYS** | **0.501** | |
| **55** | **PHE** | **0.516** | |
| **56** | **SER** | **0.53** | |
| **57** | **GLN** | **0.525** | |
| **58** | **LYS** | **0.532** | |
| **59** | **TYR** | **0.522** | |
| **60** | **ASP** | **0.533** | |
| **61** | **ALA** | **0.534** | |
| **62** | **TYR** | **0.529** | |
| **63** | **ILE** | **0.516** | |
| **64** | **ASP** | **0.511** | |
| **65** | **ALA** | **0.509** | |
| **75** | **LYS** | **0.501** | |
| **76** | **LEU** | **0.514** | |
| **77** | **THR** | **0.515** | |
| **78** | **SER** | **0.547** | |
| **79** | **GLU** | **0.549** | |
| **80** | **ARG** | **0.533** | |
| **81** | **ASN** | **0.533** | |
| **82** | **PRO** | **0.546** | |
| **83** | **ASP** | **0.536** | |
| **84** | **LEU** | **0.521** | |
| **85** | **ALA** | **0.517** | |
| **86** | **GLU** | **0.537** | |
| **87** | **VAL** | **0.512** | |
| **89** | **GLN** | **0.506** | |
| **92** | **LEU** | **0.514** | |
| **93** | **GLU** | **0.523** | |
| **94** | **GLY** | **0.523** | |
| **95** | **ARG** | **0.515** | |
| **96** | **GLN** | **0.534** | |
| **97** | **HIS** | **0.536** | |
| **98** | **HIS** | **0.528** | |
| **99** | **PHE** | **0.533** | |
| **100** | **ALA** | **0.525** | |
| **101** | **LEU** | **0.505** | |
| **110** | **LEU** | **0.53** | |
| **111** | **GLN** | **0.566** | |
| **112** | **HIS** | **0.582** | |
| **113** | **ASP** | **0.577** | |
| **114** | **PRO** | **0.6** | |
| **115** | **SER** | **0.622** | |
| **116** | **LYS** | **0.616** | |
| **117** | **VAL** | **0.619** | |
| **118** | **ALA** | **0.613** | |
| **119** | **THR** | **0.594** | |
| **120** | **THR** | **0.591** | |
| **121** | **GLN** | **0.574** | |
| **122** | **HIS** | **0.574** | |
| **123** | **VAL** | **0.566** | |
| **124** | **GLU** | **0.569** | |
| **125** | **SER** | **0.556** | |
| **126** | **TRP** | **0.551** | |
| **127** | **LEU** | **0.54** | |
| **128** | **LYS** | **0.549** | |
| **129** | **LEU** | **0.549** | |
| **130** | **GLU** | **0.544** | |
| **131** | **GLN** | **0.565** | |
| **132** | **PRO** | **0.561** | |
| **133** | **GLU** | **0.54** | |
| **134** | **ILE** | **0.546** | |
| **135** | **LYS** | **0.538** | |
| **136** | **GLN** | **0.54** | |
| **137** | **LEU** | **0.53** | |
| **138** | **ALA** | **0.52** | |
| **139** | **THR** | **0.53** | |
| **140** | **ARG** | **0.511** | |
| **141** | **SER** | **0.512** | |
| **142** | **ASP** | **0.524** | |
| **143** | **GLU** | **0.529** | |
| **144** | **LEU** | **0.512** | |
| **145** | **GLY** | **0.515** | |
| **146** | **LYS** | **0.508** | |
| **147** | **LEU** | **0.502** | |
| **151** | **THR** | **0.505** | |
| **152** | **PHE** | **0.521** | |
| **153** | **ALA** | **0.553** | |
| **154** | **ASP** | **0.572** | |
| **155** | **ARG** | **0.599** | |
| **156** | **GLN** | **0.608** | |
| **157** | **ALA** | **0.635** | |
| **158** | **THR** | **0.644** | |
| **159** | **PRO** | **0.635** | |
| **160** | **HIS** | **0.62** | |
| **161** | **PRO** | **0.604** | |
| **162** | **LYS** | **0.575** | |
| **163** | **ASN** | **0.566** | |
| **164** | **TYR** | **0.543** | |
| **165** | **GLU** | **0.523** | |
| **178** | **LYS** | **0.512** | |
| **179** | **GLN** | **0.526** | |
| **180** | **ILE** | **0.501** | |
| **181** | **GLU** | **0.505** | |
| **182** | **SER** | **0.512** | |
| **183** | **ALA** | **0.513** | |
| **184** | **LEU** | **0.506** | |
| **185** | **ASN** | **0.521** | |
| **186** | **ARG** | **0.514** | |
| **187** | **TYR** | **0.52** | |
| **188** | **ASN** | **0.519** | |
| **189** | **GLN** | **0.542** | |
| **190** | **ALA** | **0.537** | |
| **191** | **ILE** | **0.538** | |
| **192** | **GLU** | **0.526** | |
| **193** | **LYS** | **0.518** | |
| **194** | **VAL** | **0.517** | |
| **UniProt ID - Q9KND3** | | | |
| **16** | **ALA** | **0.521** | |
| **17** | **ASN** | **0.53** | |
| **18** | **ASP** | **0.533** | |
| **19** | **TYR** | **0.521** | |
| **20** | **GLN** | **0.526** | |
| **21** | **ALA** | **0.526** | |
| **22** | **GLU** | **0.514** | |
| **32** | **LYS** | **0.509** | |
| **33** | **GLN** | **0.512** | |
| **34** | **ILE** | **0.516** | |
| **35** | **SER** | **0.526** | |
| **36** | **ALA** | **0.529** | |
| **37** | **ASP** | **0.503** | |
| **46** | **LEU** | **0.509** | |
| **47** | **GLN** | **0.532** | |
| **48** | **ASN** | **0.559** | |
| **49** | **ALA** | **0.596** | |
| **50** | **LYS** | **0.584** | |
| **51** | **ASN** | **0.588** | |
| **52** | **ALA** | **0.582** | |
| **53** | **ASN** | **0.566** | |
| **54** | **LEU** | **0.566** | |
| **55** | **THR** | **0.548** | |
| **56** | **GLN** | **0.55** | |
| **57** | **SER** | **0.536** | |
| **58** | **GLU** | **0.519** | |
| **59** | **ILE** | **0.516** | |
| **60** | **ASP** | **0.518** | |
| **61** | **GLN** | **0.521** | |
| **62** | **LEU** | **0.518** | |
| **63** | **ASP** | **0.529** | |
| **64** | **LYS** | **0.529** | |
| **65** | **GLN** | **0.512** | |
| **67** | **ARG** | **0.52** | |
| **68** | **ALA** | **0.526** | |
| **69** | **GLU** | **0.53** | |
| **70** | **VAL** | **0.534** | |
| **71** | **GLY** | **0.543** | |
| **72** | **GLN** | **0.521** | |
| **73** | **SER** | **0.532** | |
| **74** | **ASP** | **0.521** | |
| **75** | **THR** | **0.547** | |
| **76** | **PRO** | **0.519** | |
| **77** | **MET** | **0.514** | |
| **78** | **ILE** | **0.503** | |
| **79** | **GLN** | **0.506** | |
| **81** | **VAL** | **0.504** | |
| **83** | **ASN** | **0.501** | |
| **84** | **THR** | **0.501** | |
| **85** | **PRO** | **0.519** | |
| **86** | **ALA** | **0.504** | |
| **87** | **SER** | **0.516** | |
| **88** | **GLN** | **0.512** | |
| **89** | **LYS** | **0.519** | |
| **90** | **LEU** | **0.513** | |
| **91** | **MET** | **0.523** | |
| **92** | **GLU** | **0.54** | |
| **93** | **ILE** | **0.534** | |
| **94** | **GLN** | **0.529** | |
| **95** | **ASN** | **0.541** | |
| **96** | **ALA** | **0.535** | |
| **97** | **SER** | **0.517** | |
| **98** | **ASN** | **0.501** | |
| **115** | **ALA** | **0.503** | |
| **116** | **GLN** | **0.509** | |
| **117** | **SER** | **0.521** | |
| **118** | **GLU** | **0.533** | |
| **119** | **VAL** | **0.556** | |
| **120** | **THR** | **0.586** | |
| **121** | **SER** | **0.596** | |
| **122** | **ASP** | **0.61** | |
| **123** | **TYR** | **0.608** | |
| **124** | **TRP** | **0.606** | |
| **125** | **GLN** | **0.612** | |
| **126** | **GLY** | **0.616** | |
| **127** | **ASP** | **0.604** | |
| **128** | **GLU** | **0.599** | |
| **129** | **ALA** | **0.591** | |
| **130** | **LYS** | **0.582** | |
| **131** | **TRP** | **0.573** | |
| **132** | **GLN** | **0.577** | |
| **133** | **ASN** | **0.588** | |
| **134** | **THR** | **0.592** | |
| **135** | **TYR** | **0.6** | |
| **136** | **SER** | **0.606** | |
| **137** | **LYS** | **0.606** | |
| **138** | **GLY** | **0.603** | |
| **139** | **VAL** | **0.607** | |
| **140** | **GLY** | **0.596** | |
| **141** | **ALA** | **0.581** | |
| **142** | **PHE** | **0.562** | |
| **143** | **ASP** | **0.547** | |
| **144** | **ILE** | **0.542** | |
| **145** | **SER** | **0.537** | |
| **146** | **ASP** | **0.535** | |
| **147** | **ILE** | **0.544** | |
| **148** | **GLU** | **0.542** | |
| **149** | **GLU** | **0.549** | |
| **150** | **ASP** | **0.557** | |
| **151** | **GLU** | **0.551** | |
| **152** | **SER** | **0.544** | |
| **153** | **THR** | **0.533** | |
| **154** | **GLN** | **0.522** | |
| **155** | **VAL** | **0.508** | |
| **168** | **SER** | **0.505** | |
| **169** | **SER** | **0.513** | |
| **UniProt ID - Q9KPA3** | | | |
| **10** | **ILE** | **0.508** | |
| **11** | **THR** | **0.509** | |
| **12** | **SER** | **0.51** | |
| **13** | **ALA** | **0.507** | |
| **18** | **LEU** | **0.504** | |
| **19** | **LEU** | **0.505** | |
| **20** | **PHE** | **0.508** | |
| **21** | **ILE** | **0.518** | |
| **22** | **PRO** | **0.511** | |
| **23** | **SER** | **0.503** | |
| **62** | **HIS** | **0.512** | |
| **63** | **LEU** | **0.541** | |
| **64** | **HIS** | **0.551** | |
| **65** | **SER** | **0.546** | |
| **66** | **GLN** | **0.551** | |
| **67** | **TRP** | **0.564** | |
| **68** | **MET** | **0.567** | |
| **69** | **PRO** | **0.561** | |
| **70** | **ALA** | **0.553** | |
| **71** | **SER** | **0.538** | |
| **72** | **LEU** | **0.518** | |
| **73** | **VAL** | **0.521** | |
| **74** | **GLY** | **0.51** | |
| **75** | **ASN** | **0.5** | |
| **85** | **GLU** | **0.515** | |
| **86** | **GLN** | **0.533** | |
| **87** | **GLU** | **0.543** | |
| **88** | **GLY** | **0.55** | |
| **89** | **GLN** | **0.546** | |
| **90** | **ARG** | **0.543** | |
| **91** | **GLU** | **0.529** | |
| **92** | **ILE** | **0.509** | |
| **93** | **PHE** | **0.514** | |
| **94** | **HIS** | **0.504** | |
| **120** | **SER** | **0.504** | |
| **121** | **CYS** | **0.53** | |
| **122** | **ASP** | **0.54** | |
| **123** | **GLU** | **0.554** | |
| **124** | **ALA** | **0.557** | |
| **125** | **THR** | **0.529** | |
| **126** | **TYR** | **0.509** | |
| **127** | **ARG** | **0.505** | |
| **136** | **GLU** | **0.505** | |
| **137** | **LEU** | **0.551** | |
| **138** | **ASN** | **0.608** | |
| **139** | **GLY** | **0.624** | |
| **140** | **SER** | **0.627** | |
| **141** | **ARG** | **0.651** | |
| **142** | **ASP** | **0.654** | |
| **143** | **HIS** | **0.632** | |
| **144** | **ALA** | **0.562** | |
| **UniProt ID- Q9KT53** | | | |
| **10** | **SER** | **0.508** | |
| **11** | **VAL** | **0.509** | |
| **12** | **ILE** | **0.51** | |
| **13** | **GLY** | **0.507** | |
| **18** | **CYS** | **0.504** | |
| **19** | **LEU** | **0.505** | |
| **20** | **GLY** | **0.508** | |
| **21** | **ILE** | **0.518** | |
| **22** | **LEU** | **0.511** | |
| **23** | **GLY** | **0.503** | |
| **62** | **MET** | **0.512** | |
| **63** | **ILE** | **0.541** | |
| **64** | **GLN** | **0.551** | |
| **65** | **ASN** | **0.546** | |
| **66** | **TRP** | **0.551** | |
| **67** | **GLN** | **0.564** | |
| **68** | **GLN** | **0.567** | |
| **69** | **HIS** | **0.561** | |
| **70** | **GLY** | **0.553** | |
| **71** | **ALA** | **0.538** | |
| **72** | **VAL** | **0.518** | |
| **73** | **SER** | **0.521** | |
| **74** | **LYS** | **0.51** | |
| **75** | **ALA** | **0.5** | |
| **85** | VAL | **0.515** | |
| **86** | LEU | **0.533** | |
| **87** | SER | **0.543** | |
| **88** | PHE | **0.55** | |
| **89** | ALA | **0.546** | |
| **90** | PHE | **0.543** | |
| **91** | SER | **0.529** | |
| **92** | ILE | **0.509** | |
| **93** | TRP | **0.514** | |
| **94** | MET | **0.504** | |
| **120** | **THR** | **0.504** | |
| **121** | **HIS** | **0.53** | |
| **122** | **GLU** | **0.54** | |
| **123** | **LEU** | **0.554** | |
| **124** | **VAL** | **0.557** | |
| **125** | **ALA** | **0.529** | |
| **126** | **ASN** | **0.509** | |
| **127** | **GLN** | **0.505** | |
| **136** | **LEU** | **0.505** | |
| **137** | **PRO** | **0.551** | |
| **138** | **ARG** | **0.608** | |
| **139** | **PHE** | **0.624** | |
| **140** | **LEU** | **0.627** | |
| **141** | **PHE** | **0.651** | |
| **142** | **SER** | **0.654** | |
| **143** | **VAL** | **0.632** | |
| **144** | **ILE** | **0.562** | |
| **UniProt ID- Q9KRE6** | | | |
| **4** | **SER** | **0.513** | |
| **5** | **LYS** | **0.533** | |
| **6** | **HIS** | **0.548** | |
| **7** | **PHE** | **0.548** | |
| **8** | **PRO** | **0.556** | |
| **9** | **HIS** | **0.577** | |
| **10** | **LEU** | **0.576** | |
| **11** | **ALA** | **0.571** | |
| **12** | **LEU** | **0.577** | |
| **13** | **LYS** | **0.57** | |
| **14** | **LYS** | **0.573** | |
| **15** | **THR** | **0.558** | |
| **16** | **PRO** | **0.564** | |
| **17** | **ASP** | **0.567** | |
| **18** | **ARG** | **0.542** | |
| **19** | **VAL** | **0.521** | |
| **20** | **LYS** | **0.516** | |
| **21** | **LEU** | **0.517** | |
| **22** | **THR** | **0.505** | |
| **24** | **LEU** | **0.5** | |
| **37** | **LEU** | **0.518** | |
| **38** | **LEU** | **0.558** | |
| **39** | **PRO** | **0.539** | |
| **40** | **GLN** | **0.557** | |
| **41** | **GLN** | **0.57** | |
| **42** | **GLN** | **0.571** | |
| **43** | **LEU** | **0.563** | |
| **44** | **LYS** | **0.56** | |
| **45** | **LYS** | **0.557** | |
| **46** | **VAL** | **0.559** | |
| **47** | **ILE** | **0.552** | |
| **48** | **ALA** | **0.565** | |
| **49** | **MET** | **0.547** | |
| **50** | **LYS** | **0.538** | |
| **51** | **PHE** | **0.542** | |
| **52** | **THR** | **0.549** | |
| **53** | **GLN** | **0.541** | |
| **54** | **GLN** | **0.547** | |
| **55** | **HIS** | **0.531** | |
| **56** | **ILE** | **0.546** | |
| **57** | **ASP** | **0.538** | |
| **58** | **GLU** | **0.515** | |
| **59** | **LEU** | **0.514** | |
| **60** | **ASN** | **0.517** | |
| **61** | **LEU** | **0.505** | |
| **62** | **LEU** | **0.52** | |
| **63** | **LEU** | **0.524** | |
| **64** | **GLN** | **0.541** | |
| **65** | **PHE** | **0.54** | |
| **66** | **ASP** | **0.555** | |
| **67** | **LEU** | **0.586** | |
| **68** | **SER** | **0.588** | |
| **69** | **SER** | **0.582** | |
| **70** | **ALA** | **0.588** | |
| **71** | **ALA** | **0.582** | |
| **72** | **THR** | **0.57** | |
| **73** | **GLY** | **0.572** | |
| **74** | **ILE** | **0.575** | |
| **75** | **LYS** | **0.583** | |
| **76** | **VAL** | **0.571** | |
| **77** | **HIS** | **0.577** | |
| **78** | **HIS** | **0.585** | |
| **79** | **ASP** | **0.587** | |
| **80** | **ALA** | **0.57** | |
| **81** | **SER** | **0.567** | |
| **82** | **GLU** | **0.551** | |
| **83** | **ALA** | **0.52** | |
| **93** | **ASN** | **0.515** | |
| **94** | **LYS** | **0.522** | |
| **95** | **GLY** | **0.534** | |
| **96** | **LEU** | **0.548** | |
| **97** | **CYS** | **0.555** | |
| **98** | **THR** | **0.553** | |
| **99** | **GLN** | **0.539** | |
| **100** | **PRO** | **0.541** | |
| **101** | **ASP** | **0.546** | |
| **102** | **GLY** | **0.552** | |
| **103** | **GLY** | **0.549** | |
| **104** | **TYR** | **0.533** | |
| **105** | **LEU** | **0.531** | |
| **106** | **THR** | **0.531** | |
| **107** | **ASP** | **0.531** | |
| **108** | **GLU** | **0.52** | |
| **UniProt ID- Q9KKS6** | | | |
| **4** | **ALA** | **0.515** | |
| **5** | **ASP** | **0.545** | |
| **6** | **ALA** | **0.544** | |
| **7** | **ASP** | **0.542** | |
| **8** | **HIS** | **0.551** | |
| **9** | **LYS** | **0.553** | |
| **10** | **LEU** | **0.543** | |
| **11** | **ALA** | **0.524** | |
| **17** | **ALA** | **0.511** | |
| **18** | **ASN** | **0.525** | |
| **19** | **THR** | **0.538** | |
| **20** | **ASP** | **0.558** | |
| **21** | **TYR** | **0.568** | |
| **22** | **GLY** | **0.58** | |
| **23** | **HIS** | **0.594** | |
| **24** | **MET** | **0.593** | |
| **25** | **SER** | **0.576** | |
| **26** | **ARG** | **0.558** | |
| **27** | **LYS** | **0.561** | |
| **28** | **LYS** | **0.563** | |
| **29** | **ILE** | **0.564** | |
| **30** | **MET** | **0.555** | |
| **31** | **LYS** | **0.558** | |
| **32** | **ASN** | **0.534** | |
| **33** | **GLU** | **0.525** | |
| **34** | **LEU** | **0.529** | |
| **35** | **GLU** | **0.516** | |
| **47** | **MET** | **0.501** | |
| **48** | **GLU** | **0.539** | |
| **49** | **ASN** | **0.544** | |
| **50** | **GLY** | **0.562** | |
| **51** | **SER** | **0.584** | |
| **52** | **PRO** | **0.582** | |
| **53** | **THR** | **0.577** | |
| **54** | **GLU** | **0.56** | |
| **55** | **PHE** | **0.555** | |
| **56** | **GLY** | **0.547** | |
| **57** | **LYS** | **0.532** | |
| **58** | **ILE** | **0.513** | |
| **59** | **TYR** | **0.513** | |
| **64** | **ALA** | **0.505** | |
| **65** | **TYR** | **0.512** | |
| **66** | **ALA** | **0.508** | |
| **67** | **ASP** | **0.505** | |
| **91** | **HIS** | **0.513** | |
| **92** | **LEU** | **0.521** | |
| **93** | **ALA** | **0.531** | |
| **94** | **TYR** | **0.55** | |
| **95** | **GLU** | **0.547** | |
| **96** | **GLN** | **0.539** | |
| **97** | **GLU** | **0.547** | |
| **98** | **HIS** | **0.544** | |
| **99** | **LEU** | **0.524** | |
| **100** | **ARG** | **0.521** | |
| **101** | **ASP** | **0.515** | |
| **UniProt ID- Q9KN87** | | | |
| **5** | **SER** | **0.513** | |
| **6** | **LYS** | **0.531** | |
| **7** | **MET** | **0.54** | |
| **8** | **GLU** | **0.546** | |
| **9** | **PHE** | **0.568** | |
| **10** | **ASP** | **0.586** | |
| **11** | **MET** | **0.595** | |
| **12** | **ILE** | **0.599** | |
| **13** | **ASN** | **0.598** | |
| **14** | **LYS** | **0.594** | |
| **15** | **LYS** | **0.592** | |
| **16** | **PHE** | **0.594** | |
| **17** | **LEU** | **0.591** | |
| **18** | **LYS** | **0.58** | |
| **19** | **THR** | **0.545** | |
| **20** | **LYS** | **0.528** | |
| **45** | **LEU** | **0.53** | |
| **46** | **GLY** | **0.547** | |
| **47** | **TRP** | **0.576** | |
| **48** | **GLN** | **0.589** | |
| **49** | **PRO** | **0.604** | |
| **50** | **GLU** | **0.602** | |
| **51** | **PRO** | **0.606** | |
| **52** | **MET** | **0.612** | |
| **53** | **LYS** | **0.596** | |
| **54** | **LYS** | **0.589** | |
| **55** | **VAL** | **0.581** | |
| **56** | **ALA** | **0.569** | |
| **57** | **LYS** | **0.547** | |
| **58** | **SER** | **0.536** | |
| **59** | **SER** | **0.51** | |
| **83** | **GLU** | **0.511** | |
| **84** | **TRP** | **0.545** | |
| **85** | **VAL** | **0.561** | |
| **86** | **ASN** | **0.549** | |
| **87** | **ASP** | **0.556** | |
| **88** | **PRO** | **0.559** | |
| **89** | **HIS** | **0.561** | |
| **90** | **ALA** | **0.566** | |
| **91** | **ASP** | **0.568** | |
| **92** | **GLN** | **0.579** | |
| **93** | **TYR** | **0.568** | |
| **94** | **ILE** | **0.568** | |
| **95** | **ALA** | **0.579** | |
| **96** | **ASN** | **0.578** | |
| **97** | **GLY** | **0.575** | |
| **98** | **PHE** | **0.562** | |
| **99** | **GLY** | **0.538** | |
| **100** | **GLU** | **0.518** | |
| **UniProt ID- Q9KU58** | | | |
| **13** | **SER** | **0.501** | |
| **14** | **LYS** | **0.547** | |
| **15** | **THR** | **0.585** | |
| **16** | **THR** | **0.609** | |
| **17** | **GLY** | **0.614** | |
| **18** | **GLU** | **0.612** | |
| **19** | **ASN** | **0.6** | |
| **20** | **THR** | **0.574** | |
| **21** | **MET** | **0.523** | |
| **40** | **ARG** | **0.516** | |
| **41** | **GLU** | **0.52** | |
| **42** | **GLU** | **0.541** | |
| **43** | **ARG** | **0.574** | |
| **44** | **VAL** | **0.569** | |
| **45** | **TRP** | **0.571** | |
| **46** | **LYS** | **0.579** | |
| **47** | **ARG** | **0.588** | |
| **48** | **LYS** | **0.577** | |
| **49** | **VAL** | **0.572** | |
| **50** | **ARG** | **0.585** | |
| **51** | **ARG** | **0.587** | |
| **52** | **SER** | **0.571** | |
| **53** | **SER** | **0.571** | |
| **54** | **TYR** | **0.572** | |
| **55** | **HIS** | **0.571** | |
| **56** | **LEU** | **0.561** | |
| **57** | **PRO** | **0.559** | |
| **58** | **TRP** | **0.563** | |
| **59** | **ASN** | **0.547** | |
| **60** | **ASN** | **0.524** | |
| **61** | **PRO** | **0.547** | |
| **62** | **HIS** | **0.556** | |
| **63** | **LEU** | **0.548** | |
| **64** | **LEU** | **0.546** | |
| **65** | **ARG** | **0.543** | |
| **66** | **ASP** | **0.553** | |
| **67** | **ILE** | **0.549** | |
| **68** | **GLY** | **0.57** | |
| **69** | **LEU** | **0.591** | |
| **70** | **GLU** | **0.585** | |
| **71** | **THR** | **0.585** | |
| **72** | **ASP** | **0.599** | |
| **73** | **GLY** | **0.604** | |
| **74** | **ARG** | **0.616** | |
| **75** | **PRO** | **0.625** | |
| **76** | **ILE** | **0.634** | |
| **77** | **GLY** | **0.627** | |
| **78** | **LEU** | **0.623** | |
| **79** | **SER** | **0.61** | |
| **80** | **LEU** | **0.593** | |
| **81** | **PRO** | **0.591** | |
| **82** | **ASP** | **0.576** | |
| **83** | **ALA** | **0.565** | |
| **84** | **VAL** | **0.555** | |
| **85** | **VAL** | **0.552** | |
| **86** | **ALA** | **0.545** | |
| **87** | **GLU** | **0.553** | |
| **88** | **ARG** | **0.556** | |
| **89** | **ARG** | **0.536** | |
| **90** | **VAL** | **0.543** | |
| **91** | **ARG** | **0.55** | |
| **92** | **HIS** | **0.545** | |
| **93** | **ILE** | **0.515** | |
| **94** | **ARG** | **0.514** | |
| **95** | **ARG** | **0.51** | |
| **UniProt ID- Q9KPP0** | | | |
| **11** | **ASN** | **0.52** | |
| **12** | **GLY** | **0.529** | |
| **13** | **GLU** | **0.562** | |
| **14** | **GLU** | **0.565** | |
| **15** | **LYS** | **0.564** | |
| **16** | **MET** | **0.56** | |
| **17** | **THR** | **0.574** | |
| **18** | **PHE** | **0.567** | |
| **19** | **THR** | **0.559** | |
| **20** | **SER** | **0.539** | |
| **21** | **LYS** | **0.542** | |
| **22** | **ALA** | **0.536** | |
| **23** | **GLU** | **0.543** | |
| **24** | **ALA** | **0.544** | |
| **25** | **ASP** | **0.551** | |
| **26** | **ALA** | **0.534** | |
| **27** | **TYR** | **0.523** | |
| **28** | **ASP** | **0.517** | |
| **39** | **GLU** | **0.51** | |
| **40** | **LEU** | **0.52** | |
| **41** | **LEU** | **0.534** | |
| **42** | **GLY** | **0.556** | |
| **43** | **LYS** | **0.553** | |
| **44** | **SER** | **0.554** | |
| **45** | **ALA** | **0.57** | |
| **46** | **LEU** | **0.605** | |
| **47** | **ILE** | **0.6** | |
| **48** | **GLU** | **0.59** | |
| **49** | **ASP** | **0.577** | |
| **50** | **GLU** | **0.571** | |
| **51** | **ALA** | **0.56** | |
| **52** | **LYS** | **0.53** | |
| **53** | **GLN** | **0.515** | |
| **72** | **GLY** | **0.526** | |
| **73** | **ALA** | **0.546** | |
| **74** | **LYS** | **0.559** | |
| **75** | **ARG** | **0.592** | |
| **76** | **LYS** | **0.627** | |
| **77** | **PRO** | **0.631** | |
| **78** | **ALA** | **0.644** | |
| **79** | **PRO** | **0.655** | |
| **80** | **ALA** | **0.651** | |
| **81** | **THR** | **0.645** | |
| **82** | **GLY** | **0.652** | |
| **83** | **LYS** | **0.651** | |
| **84** | **LYS** | **0.649** | |
| **85** | **PRO** | **0.645** | |
| **86** | **LYS** | **0.649** | |
| **87** | **ALA** | **0.644** | |
| **88** | **VAL** | **0.628** | |
| **89** | **ALA** | **0.618** | |
| **90** | **ASP** | **0.623** | |
| **91** | **SER** | **0.609** | |
| **92** | **ASP** | **0.6** | |
| **93** | **ASP** | **0.594** | |
| **94** | **ASP** | **0.597** | |
| **95** | **GLU** | **0.598** | |
| **96** | **SER** | **0.588** | |
| **97** | **VAL** | **0.568** | |
| **98** | **ASP** | **0.506** | |
| **UniProt ID- B1B1N2** | | | |
| **4** | **THR** | **0.528** | |
| **5** | **LYS** | **0.558** | |
| **6** | **ASN** | **0.552** | |
| **7** | **GLY** | **0.556** | |
| **8** | **GLU** | **0.567** | |
| **9** | **SER** | **0.548** | |
| **10** | **MET** | **0.522** | |
| **30** | **ARG** | **0.534** | |
| **31** | **GLN** | **0.564** | |
| **32** | **GLU** | **0.593** | |
| **33** | **ASN** | **0.616** | |
| **34** | **GLU** | **0.638** | |
| **35** | **TYR** | **0.644** | |
| **36** | **THR** | **0.642** | |
| **37** | **VAL** | **0.637** | |
| **38** | **LYS** | **0.622** | |
| **39** | **GLU** | **0.61** | |
| **40** | **TYR** | **0.599** | |
| **41** | **THR** | **0.588** | |
| **42** | **SER** | **0.583** | |
| **43** | **MET** | **0.554** | |
| **44** | ALA | **0.532** | |
| **45** | ASN | **0.514** | |
| **53** | GLU | **0.5** | |
| **54** | GLN | **0.51** | |
| **55** | **GLY** | **0.522** | |
| **56** | GLY | **0.539** | |
| **57** | GLN | **0.546** | |
| **58** | LEU | **0.546** | |
| **59** | GLU | **0.542** | |
| **60** | MET | **0.54** | |
| **61** | VAL | **0.544** | |
| **62** | **THR** | **0.542** | |
| **63** | **GLU** | **0.54** | |
| **64** | **ASN** | **0.531** | |
| **65** | **GLU** | **0.521** | |
| **66** | GLN | **0.515** | |
| **77** | **GLU** | **0.51** | |
| **78** | **LYS** | **0.538** | |
| **79** | **ILE** | **0.551** | |
| **80** | **GLU** | **0.561** | |
| **81** | **GLN** | **0.552** | |
| **82** | **TRP** | **0.552** | |
| **83** | **GLU** | **0.555** | |
| **84** | **TYR** | **0.559** | |
| **85** | **PHE** | **0.555** | |
| **86** | **ARG** | **0.564** | |
| **87** | **GLN** | **0.56** | |
| **88** | **ASN** | **0.553** | |
| **UniProt ID - Q9KL81** | | | |
| **6** | **SER** | **0.506** | |
| **7** | **ILE** | **0.508** | |
| **8** | **LEU** | **0.512** | |
| **9** | **GLU** | **0.514** | |
| **10** | **MET** | **0.517** | |
| **11** | **LEU** | **0.529** | |
| **12** | **ASN** | **0.537** | |
| **13** | **VAL** | **0.553** | |
| **14** | **LEU** | **0.559** | |
| **15** | **SER** | **0.56** | |
| **16** | **VAL** | **0.577** | |
| **17** | **GLU** | **0.569** | |
| **18** | **PRO** | **0.565** | |
| **19** | **LEU** | **0.568** | |
| **20** | **PRO** | **0.559** | |
| **21** | **SER** | **0.552** | |
| **22** | **THR** | **0.553** | |
| **23** | **PRO** | **0.549** | |
| **24** | **ILE** | **0.545** | |
| **25** | **ALA** | **0.533** | |
| **26** | **ARG** | **0.543** | |
| **27** | **GLU** | **0.545** | |
| **28** | **ILE** | **0.537** | |
| **29** | **PRO** | **0.541** | |
| **30** | **ARG** | **0.546** | |
| **31** | **SER** | **0.53** | |
| **32** | **ILE** | **0.525** | |
| **33** | **LYS** | **0.524** | |
| **34** | **VAL** | **0.529** | |
| **35** | **VAL** | **0.529** | |
| **36** | **GLN** | **0.543** | |
| **37** | **VAL** | **0.547** | |
| **38** | **ASN** | **0.549** | |
| **39** | **GLU** | **0.556** | |
| **40** | **LYS** | **0.559** | |
| **41** | **HIS** | **0.551** | |
| **42** | **GLN** | **0.576** | |
| **43** | **ILE** | **0.577** | |
| **44** | **ARG** | **0.565** | |
| **45** | **VAL** | **0.544** | |
| **46** | **LYS** | **0.551** | |
| **47** | **LYS** | **0.545** | |
| **48** | **MET** | **0.528** | |
| **49** | **LEU** | **0.51** | |
| **50** | **THR** | **0.518** | |
| **51** | **SER** | **0.503** | |
| **59** | **LYS** | **0.514** | |
| **60** | **LEU** | **0.531** | |
| **61** | **LEU** | **0.552** | |
| **62** | **SER** | **0.593** | |
| **63** | **LYS** | **0.59** | |
| **64** | **GLN** | **0.592** | |
| **65** | **GLU** | **0.608** | |
| **66** | **TYR** | **0.624** | |
| **67** | **SER** | **0.63** | |
| **68** | **ILE** | **0.624** | |
| **69** | **ARG** | **0.601** | |
| **70** | **LYS** | **0.597** | |
| **71** | **PRO** | **0.586** | |
| **72** | **GLU** | **0.566** | |
| **73** | **ILE** | **0.553** | |
| **74** | **LYS** | **0.533** | |
| **UniProt ID -Q9KL73** | | | |
| **3** | **LYS** | **0.511** | |
| **4** | **ARG** | **0.582** | |
| **5** | **VAL** | **0.605** | |
| **6** | **LYS** | **0.625** | |
| **7** | **LEU** | **0.64** | |
| **8** | **PRO** | **0.647** | |
| **9** | **LYS** | **0.657** | |
| **10** | **LYS** | **0.657** | |
| **11** | **ASN** | **0.64** | |
| **12** | **LYS** | **0.623** | |
| **13** | **LYS** | **0.613** | |
| **14** | **GLY** | **0.584** | |
| **15** | **ALA** | **0.576** | |
| **16** | **TYR** | **0.551** | |
| **17** | **GLU** | **0.543** | |
| **18** | **ALA** | **0.523** | |
| **19** | **LYS** | **0.5** | |
| 20 | PHE | 0.487 | |
| **21** | **GLU** | **0.507** | |
| **22** | **GLU** | **0.505** | |
| **23** | **MET** | **0.519** | |
| **24** | **VAL** | **0.525** | |
| **25** | **LYS** | **0.538** | |
| **26** | **GLU** | **0.517** | |
| **27** | **TYR** | **0.528** | |
| **28** | **HIS** | **0.537** | |
| **29** | **SER** | **0.536** | |
| **30** | **ALA** | **0.508** | |
| **31** | **GLN** | **0.506** | |
| **39** | **VAL** | **0.519** | |
| **40** | **GLY** | **0.523** | |
| **41** | **SER** | **0.541** | |
| **42** | **GLU** | **0.554** | |
| **43** | **GLU** | **0.554** | |
| **44** | **TYR** | **0.554** | |
| **45** | **THR** | **0.563** | |
| **46** | **GLU** | **0.567** | |
| **47** | **GLN** | **0.535** | |
| **48** | **LYS** | **0.522** | |
| **49** | **ILE** | **0.519** | |
| **60** | **ARG** | **0.505** | |
| **UniProt ID -Q9KNG0** | | | |
| **4** | **SER** | **0.502** | |
| **5** | **ILE** | **0.536** | |
| **6** | **ASP** | **0.539** | |
| **7** | **HIS** | **0.567** | |
| **8** | **ASP** | **0.577** | |
| **9** | **ASP** | **0.579** | |
| **10** | **MET** | **0.583** | |
| **11** | **THR** | **0.587** | |
| **12** | **ASN** | **0.597** | |
| **13** | **ILE** | **0.585** | |
| **14** | **ALA** | **0.586** | |
| **15** | **ASN** | **0.594** | |
| **16** | **LYS** | **0.577** | |
| **17** | **TRP** | **0.576** | |
| **18** | **ASP** | **0.58** | |
| **19** | **SER** | **0.576** | |
| **20** | **ILE** | **0.577** | |
| **21** | **GLU** | **0.57** | |
| **22** | **GLU** | **0.576** | |
| **23** | **ILE** | **0.569** | |
| **24** | **GLU** | **0.579** | |
| **25** | **SER** | **0.581** | |
| **26** | **GLN** | **0.585** | |
| **27** | **ARG** | **0.576** | |
| **28** | **PRO** | **0.575** | |
| **29** | **THR** | **0.573** | |
| **30** | **LYS** | **0.573** | |
| **31** | **ASN** | **0.567** | |
| **32** | **LEU** | **0.568** | |
| **33** | **LYS** | **0.558** | |
| **34** | **SER** | **0.563** | |
| **35** | **ALA** | **0.556** | |
| **36** | **GLU** | **0.56** | |
| **37** | **ALA** | **0.549** | |
| **38** | **ARG** | **0.549** | |
| **39** | **ARG** | **0.543** | |
| **40** | **ARG** | **0.529** | |
| **41** | **ILE** | **0.533** | |
| **42** | **GLU** | **0.526** | |
| **45** | **ARG** | **0.506** | |
| **46** | **GLU** | **0.522** | |
| **47** | **ILE** | **0.52** | |
| **48** | **ARG** | **0.53** | |
| **49** | **GLU** | **0.548** | |
| **50** | **SER** | **0.536** | |
| **51** | **GLY** | **0.546** | |
| **52** | **LEU** | **0.566** | |
| **53** | **THR** | **0.555** | |
| **54** | **ILE** | **0.556** | |
| **55** | **GLU** | **0.557** | |
| **56** | **GLU** | **0.549** | |
| **57** | **ALA** | **0.547** | |
| **58** | **LYS** | **0.544** | |
| **59** | **GLU** | **0.541** | |
| **60** | **LEU** | **0.527** | |
| **UniProt ID -Q9KPZ1** | | | |
| **40** | **THR** | **0.509** | |
| **41** | **ASP** | **0.518** | |
| **UniProt ID -Q9KNI6** | | | |
| **5** | **VAL** | **0.527** | |
| **6** | **LEU** | **0.542** | |
| **7** | **GLY** | **0.544** | |
| **8** | **GLY** | **0.557** | |
| **9** | **GLU** | **0.561** | |
| **10** | **ILE** | **0.564** | |
| **11** | **ALA** | **0.568** | |
| **12** | **ASN** | **0.557** | |
| **13** | **GLU** | **0.557** | |
| **14** | **ASN** | **0.544** | |
| **15** | **LYS** | **0.532** | |
| **16** | **LEU** | **0.522** | |
| **17** | **LYS** | **0.519** | |
| **18** | **LYS** | **0.51** | |
| **31** | **ALA** | **0.516** | |
| **32** | **SER** | **0.548** | |
| **33** | **SER** | **0.557** | |
| **34** | **GLU** | **0.576** | |
| **35** | **ALA** | **0.595** | |
| **36** | **ASP** | **0.6** | |
| **37** | **ARG** | **0.61** | |
| **38** | **GLN** | **0.612** | |
| **39** | **GLU** | **0.599** | |
| **40** | **GLU** | **0.593** | |
| **41** | **ARG** | **0.582** | |
| **42** | **SER** | **0.517** | |
| **5** | **VAL** | **0.514** | |
| **6** | **LEU** | **0.521** | |
| **7** | **GLY** | **0.531** | |
| **8** | **ARG** | **0.551** | |
| **9** | **GLU** | **0.555** | |
| **10** | **ILE** | **0.553** | |
| **11** | **ALA** | **0.554** | |
| **12** | **ASN** | **0.552** | |
| **13** | **GLU** | **0.545** | |
| **14** | **ASN** | **0.542** | |
| **15** | **LYS** | **0.547** | |
| **16** | **LEU** | **0.538** | |
| **17** | **LYS** | **0.534** | |
| **18** | **LYS** | **0.525** | |
| **19** | **VAL** | **0.514** | |
| **20** | **PHE** | **0.507** | |
| **21** | **ASP** | **0.505** | |
| **32** | **SER** | **0.519** | |
| **33** | **SER** | **0.514** | |
| **34** | **GLU** | **0.53** | |
| **35** | **ALA** | **0.541** | |
| **36** | **ASP** | **0.553** | |
| **37** | **CYS** | **0.563** | |
| **38** | **LYS** | **0.565** | |
| **39** | **GLU** | **0.561** | |
| **40** | **GLU** | **0.56** | |
| **41** | **ARG** | **0.557** | |
| **42** | **SER** | **0.507** | |

**^ℷ^** the threshold score for the determination of linear B-cell epitope within uncharacterized candidate

protein is 0.5
