# Supplementary material for: Comprehensive in silico analyses of fifty-one uncharacterized proteins from Vibrio cholerae
Source: PLoS One. 2024 Oct 4;19(10):e0311301. doi: 10.1371/journal.pone.0311301 (PMC11452002; doi:10.1371/journal.pone.0311301)
Supplement: S13 Table — (DOCX) [file pone.0311301.s013.docx]

**Table S13**

**Identification of discontinuous B-cell epitopes present within the candidate uncharacterized proteins**

| **UniProt ID -Q9KRD2** | | | | |
| --- | --- | --- | --- | --- |
| **Residue ID** | **Residue Name** | **Contact Number** | **Propensity score** | **Discotope score** |
| **1** | **MET** | **0** | **-1.523** | **-1.348** |
| **2** | **SER** | **1** | **-1.649** | **-1.574** |
| **3** | **VAL** | **2** | **-1.718** | **-1.751** |
| **4** | **ASN** | **2** | **-1.125** | **-1.226** |
| **5** | **VAL** | **2** | **-1.723** | **-1.755** |
| **6** | **SER** | **2** | **-1.104** | **-1.207** |
| **7** | **THR** | **1** | **-1.746** | **-1.661** |
| **8** | **VAL** | **3** | **-2.55** | **-2.602** |
| **9** | **GLN** | **4** | **-3.163** | **-3.259** |
| **10** | **PRO** | **2** | **-3.787** | **-3.582** |
| **11** | **GLN** | **1** | **-2.164** | **-2.03** |
| **13** | **ILE** | **0** | **-2.64** | **-2.337** |
| **181** | **GLN** | **0** | **0.855** | **0.756** |
| **182** | **HIS** | **5** | **1.817** | **1.033** |
| **185** | **VAL** | **4** | **1.258** | **0.654** |
| **186** | **ASP** | **12** | **0.833** | **-0.643** |
| 187 | MET | 25 | -2.302 | -4.912 |
| **188** | **TRP** | **14** | **-0.876** | **-2.386** |
| **189** | **GLN** | **6** | **2.31** | **1.354** |
| **190** | **GLN** | **14** | **2.445** | **0.554** |
| **191** | **LEU** | **26** | **-0.208** | **-3.174** |
| **192** | **ARG** | **9** | **1.479** | **0.274** |
| **193** | **GLU** | **6** | **2.797** | **1.785** |
| **194** | **GLN** | **11** | **0.472** | **-0.847** |
| **224** | **LYS** | **3** | **-0.911** | **-1.151** |
| **225** | **GLY** | **0** | **0.546** | **0.483** |
| **290** | **SER** | **0** | **-3.547** | **-3.14** |
| **291** | **ASP** | **1** | **-1.688** | **-1.609** |
| **331** | **LEU** | **2** | **-3.328** | **-3.175** |
| **394** | **HIS** | **8** | **-0.788** | **-1.617** |
| **395** | **ALA** | **0** | **0.722** | **0.639** |
| **396** | **GLY** | **1** | **2.335** | **1.952** |
| **397** | **GLU** | **16** | **-1.449** | **-3.123** |
| **398** | **THR** | **1** | **-1.446** | **-1.395** |
| **673** | **LYS** | **7** | **-1.87** | **-2.46** |
| **675** | **THR** | **10** | **-1.319** | **-2.317** |
| **676** | **ASN** | **1** | **0.78** | **0.575** |
| **677** | **ARG** | **9** | **-0.139** | **-1.158** |
| **678** | **GLU** | **8** | **0.343** | **-0.617** |
| **679** | **SER** | **3** | **3.755** | **2.978** |
| **680** | **ASP** | **6** | **1.543** | **0.676** |
| **681** | **LYS** | **9** | **-0.148** | **-1.166** |
| **682** | **GLN** | **3** | **1.442** | **0.931** |
| **683** | **SER** | **0** | **-1.634** | **-1.446** |
| **749** | **GLU** | **0** | **-4.005** | **-3.545** |
| **UniProt ID -Q9KVG3** | | | | |
| **1** | **MET** | **8** | **1.519** | **0.424** |
| **2** | **THR** | **23** | **-0.89** | **-3.433** |
| **43** | **PHE** | **15** | **-1.813** | **-3.33** |
| **44** | **ASP** | **6** | **2.255** | **1.305** |
| **45** | **GLY** | **9** | **-0.957** | **-1.882** |
| **46** | **SER** | **3** | **-0.334** | **-0.641** |
| **153** | **ASN** | **0** | **-2.034** | **-1.8** |
| **155** | **SER** | **10** | **-0.967** | **-2.006** |
| **156** | **SER** | **18** | **-1.308** | **-3.228** |
| **157** | **GLY** | **7** | **0.566** | **-0.304** |
| **189** | **CYS** | **4** | **-2.692** | **-2.842** |
| **190** | **LYS** | **7** | **-3.252** | **-3.683** |
| **193** | **ARG** | **8** | **-1.543** | **-2.285** |
| **197** | **GLY** | **5** | **-0.277** | **-0.82** |
| **198** | **ARG** | **19** | **2.339** | **-0.115** |
| **199** | **LEU** | **5** | **2.405** | **1.553** |
| **200** | **TYR** | **23** | **2.866** | **-0.109** |
| **201** | **GLU** | **15** | **5.28** | **2.948** |
| **202** | **GLY** | **9** | **4.032** | **2.533** |
| **203** | **LYS** | **11** | **4.754** | **2.942** |
| **204** | **ASN** | **20** | **3.16** | **0.497** |
| **205** | **GLN** | **9** | **-2.146** | **-2.934** |
| **240** | **TYR** | **14** | **-0.928** | **-2.431** |
| **241** | **ASP** | **20** | **2.406** | **-0.17** |
| **242** | **MET** | **30** | **3.13** | **-0.68** |
| **243** | **ARG** | **25** | **4.939** | **1.496** |
| **244** | **GLN** | **12** | **5.777** | **3.733** |
| **245** | **ASN** | **20** | **7.738** | **4.548** |
| **246** | **TYR** | **21** | **9.349** | **5.859** |
| **247** | **GLU** | **13** | **9.491** | **6.905** |
| **248** | **GLU** | **12** | **9.635** | **7.147** |
| **249** | **GLY** | **22** | **10.717** | **6.954** |
| **250** | **LYS** | **14** | **10.66** | **7.824** |
| **251** | **ARG** | **7** | **11.054** | **8.978** |
| **252** | **ARG** | **16** | **10.466** | **7.422** |
| **253** | **VAL** | **25** | **10.914** | **6.784** |
| **254** | **ASP** | **7** | **11.678** | **9.53** |
| **255** | **ASP** | **8** | **10.182** | **8.091** |
| **256** | **LYS** | **19** | **8.102** | **4.985** |
| **257** | **ILE** | **18** | **8.131** | **5.126** |
| **258** | **GLN** | **6** | **7.67** | **6.098** |
| **259** | **SER** | **7** | **5.563** | **4.118** |
| **260** | **GLY** | **0** | **4.811** | **4.258** |
| **261** | **ILE** | **8** | **5.29** | **3.762** |
| **262** | **TRP** | **20** | **5.797** | **2.83** |
| **263** | **GLN** | **1** | **4.492** | **3.86** |
| **264** | **TRP** | **3** | **4.33** | **3.487** |
| **265** | **ILE** | **18** | **6.997** | **4.123** |
| **266** | **SER** | **2** | **8.045** | **6.89** |
| **267** | **GLU** | **12** | **10.389** | **7.814** |
| **268** | **GLU** | **0** | **8.841** | **7.824** |
| **269** | **SER** | **16** | **8.215** | **5.43** |
| **270** | **ASN** | **0** | **7.952** | **7.038** |
| **271** | **GLU** | **8** | **7.765** | **5.952** |
| **272** | **TYR** | **19** | **9.988** | **6.654** |
| **273** | **ARG** | **7** | **9.407** | **7.52** |
| **274** | **VAL** | **5** | **8.407** | **6.866** |
| **275** | **GLU** | **20** | **8.753** | **5.447** |
| **276** | **VAL** | **22** | **9.854** | **6.191** |
| **277** | **ASN** | **12** | **9.861** | **7.347** |
| **278** | **LYS** | **9** | **8.065** | **6.102** |
| **279** | **HIS** | **18** | **8.935** | **5.838** |
| **280** | **GLN** | **21** | **10.024** | **6.457** |
| **281** | **PRO** | **14** | **8.043** | **5.508** |
| **282** | **ALA** | **17** | **5.75** | **3.133** |
| **283** | **SER** | **5** | **4.56** | **3.461** |
| **284** | **TYR** | **19** | **3.984** | **1.341** |
| **285** | **SER** | **1** | **0.894** | **0.676** |
| **286** | **SER** | **7** | **0.997** | **0.078** |
| **287** | **ILE** | **24** | **2.566** | **-0.489** |
| **288** | **ILE** | **17** | **0.032** | **-1.927** |
| **289** | **ASP** | **5** | **-0.605** | **-1.111** |
| **290** | **ALA** | **15** | **-0.588** | **-2.246** |
| **293** | **LYS** | **7** | **-2.887** | **-3.36** |
| **294** | **ASP** | **2** | **-3.108** | **-2.981** |
| **303** | **LYS** | **9** | **-1.795** | **-2.623** |
| **304** | **SER** | **4** | **-0.562** | **-0.957** |
| **305** | **ALA** | **21** | **-0.093** | **-2.497** |
| **306** | **LEU** | **0** | **-1.254** | **-1.11** |
| **346** | **GLU** | **1** | **-0.705** | **-0.739** |
| **347** | **MET** | **7** | **-0.425** | **-1.181** |
| **348** | **TYR** | **3** | **-0.131** | **-0.461** |
| **349** | **GLY** | **0** | **2.393** | **2.118** |
| **350** | **LYS** | **7** | **1.048** | **0.122** |
| **362** | **PHE** | **7** | **-1.732** | **-2.337** |
| **397** | **LEU** | **12** | **-2.537** | **-3.625** |
| **398** | **GLU** | **8** | **0.929** | **-0.098** |
| **399** | **ASP** | **20** | **3.735** | **1.005** |
| **400** | **ASP** | **1** | **4.345** | **3.73** |
| **401** | **ASP** | **16** | **2.538** | **0.406** |
| **402** | **PRO** | **16** | **1.574** | **-0.447** |
| **403** | **MET** | **23** | **0.274** | **-2.403** |
| **404** | **SER** | **15** | **1.489** | **-0.407** |
| **405** | **VAL** | **10** | **-1.881** | **-2.814** |
| **416** | **GLY** | **9** | **1.436** | **0.236** |
| **417** | **ARG** | **6** | **7.177** | **5.661** |
| **418** | **LYS** | **24** | **8.141** | **4.445** |
| **419** | **SER** | **15** | **9.306** | **6.51** |
| **420** | **GLN** | **9** | **8.673** | **6.641** |
| **447** | **GLU** | **0** | **-3.934** | **-3.481** |
| **451** | **ASP** | **7** | **-2.245** | **-2.792** |
| **454** | **ARG** | **12** | **-1.207** | **-2.448** |
| **458** | **PRO** | **18** | **1.246** | **-0.967** |
| **459** | **ASN** | **23** | **2.733** | **-0.227** |
| **460** | **ARG** | **27** | **1.951** | **-1.378** |
| **461** | **GLU** | **13** | **4.645** | **2.616** |
| **462** | **PRO** | **2** | **3.616** | **2.97** |
| **463** | **ASN** | **5** | **4.622** | **3.515** |
| **464** | **ASN** | **18** | **3.52** | **1.045** |
| **465** | **ALA** | **26** | **0.717** | **-2.355** |
| **466** | **ASP** | **8** | **2.46** | **1.258** |
| **467** | **GLU** | **8** | **2.9** | **1.647** |
| **468** | **ARG** | **19** | **1.802** | **-0.59** |
| **470** | **LYS** | **4** | **-1.928** | **-2.166** |
| **471** | **VAL** | **6** | **-3.101** | **-3.434** |
| **508** | **LEU** | **4** | **-0.278** | **-0.706** |
| **509** | **ASP** | **4** | **0.655** | **0.12** |
| **510** | **LEU** | **11** | **-2.057** | **-3.085** |
| **511** | **PRO** | **2** | **0.23** | **-0.026** |
| **512** | **THR** | **12** | **-0.509** | **-1.83** |
| 513 | THR | 18 | -3.597 | -5.253 |
| **514** | **GLU** | **5** | **-1.878** | **-2.237** |
| **515** | **TRP** | **15** | **-0.325** | **-2.013** |
| **518** | **GLU** | **14** | **-1.326** | **-2.784** |
| **531** | **ASP** | **10** | **-2.643** | **-3.489** |
| **532** | **THR** | **0** | **-2.981** | **-2.638** |
| **533** | **GLU** | **0** | **-3.006** | **-2.66** |
| **548** | **GLN** | **7** | **-1.592** | **-2.214** |
| **551** | **HIS** | **18** | **-0.305** | **-2.34** |
| **552** | **GLU** | **15** | **-0.742** | **-2.381** |
| **554** | **TYR** | **23** | **-0.234** | **-2.852** |
| **555** | **LYS** | **12** | **1.552** | **-0.006** |
| **556** | **GLY** | **20** | **0.68** | **-1.698** |
| **557** | **CYS** | **0** | **0.798** | **0.707** |
| **558** | **ASP** | **8** | **1.468** | **0.379** |
| **559** | **SER** | **9** | **-2.288** | **-3.06** |
| **560** | **GLY** | **6** | **-1.397** | **-1.926** |
| **561** | **GLN** | **14** | **-1.346** | **-2.802** |
| **571** | **ASN** | **14** | **-1.164** | **-2.64** |
| **572** | **LYS** | **6** | **-0.486** | **-1.121** |
| **573** | **ASN** | **19** | **0.93** | **-1.362** |
| **574** | **ARG** | **20** | **1.199** | **-1.239** |
| **575** | **ILE** | **7** | **2.937** | **1.794** |
| **576** | **SER** | **6** | **3.185** | **2.129** |
| **577** | **LEU** | **18** | **2.048** | **-0.258** |
| **578** | **HIS** | **3** | **2.956** | **2.271** |
| **579** | **LYS** | **0** | **3.278** | **2.901** |
| **580** | **LYS** | **11** | **2.132** | **0.622** |
| **581** | **TYR** | **25** | **1.917** | **-1.179** |
| **582** | **LYS** | **8** | **2.236** | **1.059** |
| **583** | **SER** | **6** | **1.833** | **0.933** |
| **584** | **TRP** | **9** | **0.608** | **-0.497** |
| **585** | **ASP** | **1** | **0.463** | **0.294** |
| **586** | **ALA** | **11** | **-0.094** | **-1.348** |
| **587** | **ILE** | **27** | **-0.455** | **-3.508** |
| **588** | **ASN** | **11** | **-2.078** | **-3.104** |
| **589** | **GLU** | **8** | **-0.331** | **-1.213** |
| **644** | **GLN** | **8** | **-2.886** | **-3.474** |
| **648** | **LYS** | **8** | **-1.686** | **-2.412** |
| **651** | **THR** | **11** | **-2.52** | **-3.495** |
| **652** | **ASP** | **7** | **-2.138** | **-2.697** |
| **655** | **ALA** | **6** | **-2.3** | **-2.726** |
| **656** | **ILE** | **7** | **-2.267** | **-2.811** |
| **657** | **GLY** | **0** | **-2.901** | **-2.567** |
| **658** | **SER** | **7** | **-2.341** | **-2.877** |
| **660** | **THR** | **3** | **-2.26** | **-2.345** |
| **661** | **SER** | **6** | **-1.925** | **-2.394** |
| **662** | **SER** | **0** | **-2.69** | **-2.38** |
| **682** | **GLN** | **4** | **-0.834** | **-1.198** |
| **684** | **PRO** | **0** | **-0.676** | **-0.598** |
| **686** | **THR** | **9** | **0.372** | **-0.706** |
| **687** | **ARG** | **8** | **-0.053** | **-0.967** |
| **689** | **GLU** | **6** | **-0.474** | **-1.109** |
| **691** | **GLY** | **2** | **-0.195** | **-0.402** |
| **692** | **ASN** | **14** | **-0.529** | **-2.078** |
| **693** | **ASP** | **0** | **0.755** | **0.668** |
| **694** | **ARG** | **14** | **0.104** | **-1.518** |
| **695** | **TYR** | **25** | **-0.25** | **-3.096** |
| **696** | **GLU** | **9** | **0.808** | **-0.32** |
| **697** | **GLN** | **10** | **0.018** | **-1.134** |
| **698** | **ILE** | **18** | **-1.7** | **-3.575** |
| **699** | **PRO** | **3** | **-0.179** | **-0.504** |
| **700** | **ASN** | **8** | **-0.21** | **-1.106** |
| **701** | **TYR** | **14** | **-1.957** | **-3.342** |
| **702** | **GLU** | **0** | **-1.46** | **-1.292** |
| **703** | **GLY** | **14** | **-0.709** | **-2.238** |
| **UniProt ID -Q9KT38** | | | | |
| **1** | **MET** | **0** | **-2.329** | **-2.061** |
| **2** | **GLY** | **0** | **-3.343** | **-2.959** |
| **11** | **LYS** | **2** | **-2.479** | **-2.424** |
| **12** | **ASN** | **0** | **-1.527** | **-1.351** |
| **13** | **THR** | **2** | **-1.527** | **-1.582** |
| **14** | **THR** | **1** | **-1.799** | **-1.707** |
| **15** | **TYR** | **3** | **-2.124** | **-2.224** |
| **16** | **HIS** | **0** | **-1.29** | **-1.142** |
| **17** | **TYR** | **5** | **-1.482** | **-1.886** |
| **18** | **SER** | **3** | **-1.669** | **-1.822** |
| **19** | **THR** | **0** | **-3.357** | **-2.971** |
| **20** | **SER** | **0** | **-3.914** | **-3.464** |
| **234** | **ALA** | **0** | **-4.154** | **-3.677** |
| **286** | **PRO** | **0** | **-0.653** | **-0.578** |
| **287** | **ASP** | **3** | **-2.669** | **-2.707** |
| **439** | **THR** | **0** | **-3.206** | **-2.837** |
| **441** | **MET** | **3** | **-2.953** | **-2.959** |
| **485** | **GLU** | **1** | **-3.24** | **-2.982** |
| **486** | **GLN** | **7** | **-2.485** | **-3.004** |
| **489** | **HIS** | **6** | **-2.403** | **-2.817** |
| **494** | **GLN** | **10** | **-1.674** | **-2.632** |
| **495** | **PRO** | **16** | **-0.317** | **-2.121** |
| **496** | **ASN** | **10** | **0.45** | **-0.752** |
| **497** | **TRP** | **0** | **-0.242** | **-0.215** |
| **498** | **ARG** | **0** | **-0.079** | **-0.07** |
| **499** | **ALA** | **4** | **-0.365** | **-0.783** |
| **500** | **THR** | **6** | **-0.993** | **-1.569** |
| **501** | **LEU** | **4** | **-1.557** | **-1.838** |
| **502** | **LEU** | **5** | **-0.613** | **-1.117** |
| **503** | **ALA** | **6** | **-1.603** | **-2.109** |
| **504** | **GLU** | **6** | **-2.114** | **-2.561** |
| **505** | **LYS** | **6** | **-1.255** | **-1.801** |
| **506** | **LYS** | **6** | **-1.357** | **-1.891** |
| **507** | **TYR** | **6** | **-2.35** | **-2.77** |
| **508** | **GLN** | **6** | **-2.734** | **-3.109** |
| **533** | **SER** | **10** | **-2.352** | **-3.231** |
| **537** | **ASN** | **3** | **-2.825** | **-2.845** |
| **545** | **ARG** | **9** | **-2.444** | **-3.198** |
| **548** | **TYR** | **8** | **-2.858** | **-3.45** |
| **549** | **ASN** | **13** | **-1.548** | **-2.865** |
| **552** | **ARG** | **4** | **0.615** | **0.084** |
| **553** | **ARG** | **4** | **1.134** | **0.544** |
| **554** | **GLY** | **10** | **-2.73** | **-3.566** |
| **574** | **ASP** | **5** | **-1.9** | **-2.256** |
| **575** | **GLN** | **13** | **-1.871** | **-3.151** |
| **577** | **GLY** | **6** | **-1.251** | **-1.797** |
| **578** | **HIS** | **2** | **-2.037** | **-2.032** |
| **579** | **GLU** | **6** | **-2.295** | **-2.721** |
| **635** | **GLU** | **6** | **-2.295** | **-2.721** |
| **637** | **ASP** | **0** | **-2.335** | **-2.066** |
| **653** | **GLU** | **1** | **-3.027** | **-2.794** |
| **654** | **ARG** | **3** | **-2.364** | **-2.437** |
| **655** | **THR** | **6** | **-3.332** | **-3.639** |
| **657** | **SER** | **3** | **-2.165** | **-2.261** |
| **681** | **PHE** | **0** | **-2.688** | **-2.379** |
| **682** | **GLN** | **7** | **-3.058** | **-3.511** |
| **684** | **ALA** | **1** | **-3.844** | **-3.517** |
| **UniProt ID -Q9KKL8** | | | | |
| **151** | **LYS** | **18** | **0.105** | **-1.977** |
| **152** | **GLU** | **5** | **3.265** | **2.314** |
| **153** | **PRO** | **20** | **7.739** | **4.549** |
| **154** | **ILE** | **6** | **8.487** | **6.821** |
| **155** | **GLN** | **7** | **9.488** | **7.592** |
| **156** | **GLY** | **18** | **9.353** | **6.208** |
| **157** | **ARG** | **6** | **6.569** | **5.123** |
| **158** | **ASP** | **8** | **3.952** | **2.577** |
| **159** | **THR** | **17** | **1.948** | **-0.231** |
| **160** | **GLN** | **8** | **0.158** | **-0.78** |
| **174** | **ARG** | **6** | **-2.645** | **-3.031** |
| **175** | **PRO** | **10** | **-0.548** | **-1.635** |
| **176** | **GLN** | **15** | **0.585** | **-1.207** |
| **177** | **GLN** | **4** | **2.175** | **1.465** |
| **178** | **LYS** | **11** | **1.519** | **0.079** |
| **179** | **ALA** | **1** | **2.176** | **1.811** |
| **180** | **GLY** | **0** | **4** | **3.54** |
| **181** | **GLU** | **9** | **3.847** | **2.369** |
| **182** | **ASN** | **0** | **3.518** | **3.114** |
| **183** | **LYS** | **4** | **3.232** | **2.4** |
| **184** | **LEU** | **9** | **2.183** | **0.897** |
| **185** | **ASP** | **19** | **-0.194** | **-2.357** |
| **186** | **MET** | **6** | **-2.307** | **-2.731** |
| **205** | **LEU** | **13** | **-0.023** | **-1.515** |
| **206** | **PRO** | **7** | **2.074** | **1.031** |
| **207** | **ALA** | **15** | **4.068** | **1.876** |
| **208** | **THR** | **10** | **6.586** | **4.679** |
| **209** | **LYS** | **5** | **8.175** | **6.66** |
| **210** | **GLY** | **20** | **9.344** | **5.97** |
| **211** | **GLU** | **6** | **8.53** | **6.859** |
| **212** | **MET** | **9** | **7.491** | **5.594** |
| **213** | **GLY** | **20** | **4.674** | **1.836** |
| **214** | **TYR** | **12** | **0.128** | **-1.267** |
| **219** | **LYS** | **12** | **-0.867** | **-2.148** |
| **220** | **PRO** | **6** | **3.242** | **2.179** |
| **221** | **ILE** | **21** | **4.76** | **1.798** |
| **222** | **PRO** | **3** | **8.061** | **6.789** |
| **223** | **PRO** | **18** | **8.976** | **5.874** |
| **224** | **LYS** | **6** | **9.552** | **7.764** |
| **225** | **PRO** | **6** | **9.288** | **7.53** |
| **226** | **GLY** | **19** | **7.41** | **4.373** |
| **227** | **LYS** | **3** | **5.092** | **4.161** |
| **228** | **GLU** | **8** | **3.106** | **1.829** |
| **229** | **SER** | **16** | **0.082** | **-1.768** |
| **230** | **ALA** | **6** | **-1.186** | **-1.739** |
| **241** | **PRO** | **2** | **-2.623** | **-2.551** |
| **242** | **ASN** | **4** | **-2.259** | **-2.459** |
| **244** | **PRO** | **5** | **-2.244** | **-2.561** |
| **245** | **ASN** | **10** | **-2.328** | **-3.21** |
| **333** | **THR** | **2** | **-3.584** | **-3.401** |
| **335** | **ASP** | **0** | **-2.366** | **-2.093** |
| **426** | **GLN** | **10** | **-1.83** | **-2.769** |
| **427** | **SER** | **10** | **-0.074** | **-1.215** |
| **428** | **TYR** | **24** | **0.623** | **-2.208** |
| **429** | **LYS** | **13** | **2.484** | **0.703** |
| **430** | **GLU** | **7** | **4.35** | **3.045** |
| **431** | **ARG** | **16** | **4.06** | **1.753** |
| **432** | **ILE** | **17** | **4.237** | **1.795** |
| **433** | **ASN** | **6** | **4.673** | **3.445** |
| **434** | **LYS** | **8** | **4.392** | **2.967** |
| **435** | **HIS** | **20** | **2.808** | **0.185** |
| **436** | **LYS** | **7** | **3.342** | **2.153** |
| **437** | **GLU** | **6** | **3.064** | **2.022** |
| **438** | **LEU** | **18** | **1.575** | **-0.676** |
| **439** | **TYR** | **17** | **1.467** | **-0.657** |
| **440** | **LYS** | **6** | **1.528** | **0.662** |
| **441** | **GLN** | **10** | **0.883** | **-0.368** |
| **442** | **ALA** | **20** | **-0.426** | **-2.677** |
| **443** | **GLN** | **9** | **0.103** | **-0.944** |
| **444** | **ASP** | **6** | **0.003** | **-0.688** |
| **445** | **THR** | **19** | **-0.577** | **-2.695** |
| **446** | **THR** | **17** | **-0.631** | **-2.513** |
| **447** | **MET** | **6** | **0.053** | **-0.644** |
| **448** | **ALA** | **11** | **0.831** | **-0.53** |
| **449** | **LEU** | **22** | **0.202** | **-2.352** |
| **450** | **ILE** | **9** | **0.571** | **-0.53** |
| **451** | **ARG** | **6** | **1.055** | **0.244** |
| **452** | **ARG** | **18** | **1.066** | **-1.126** |
| **453** | **GLU** | **15** | **1.596** | **-0.312** |
| **454** | **ILE** | **6** | **1.915** | **1.004** |
| **455** | **GLU** | **9** | **1.956** | **0.696** |
| **456** | **PHE** | **18** | **1.769** | **-0.505** |
| **457** | **LYS** | **8** | **2.729** | **1.495** |
| **458** | **LYS** | **6** | **3.196** | **2.138** |
| **459** | **ARG** | **14** | **3.432** | **1.427** |
| **460** | **PRO** | **2** | **3.91** | **3.23** |
| **461** | **LYS** | **0** | **3.757** | **3.325** |
| **462** | **ALA** | **0** | **3.555** | **3.147** |
| **463** | **GLU** | **7** | **3.43** | **2.231** |
| **464** | **ARG** | **16** | **3.161** | **0.957** |
| **465** | **SER** | **5** | **2.187** | **1.36** |
| **466** | **ASP** | **0** | **1.726** | **1.527** |
| **467** | **GLU** | **1** | **0.987** | **0.759** |
| **468** | **GLU** | **16** | **1.135** | **-0.836** |
| **469** | **ALA** | **10** | **1.027** | **-0.241** |
| **470** | **GLN** | **3** | **0.634** | **0.216** |
| **471** | **GLU** | **11** | **0.615** | **-0.721** |
| **472** | **ILE** | **17** | **0.287** | **-1.701** |
| **473** | **GLU** | **8** | **0.463** | **-0.51** |
| **474** | **THR** | **6** | **-0.608** | **-1.228** |
| **475** | **ALA** | **19** | **-0.569** | **-2.688** |
| **476** | **LYS** | **15** | **-0.779** | **-2.414** |
| **477** | **ALA** | **6** | **-1.757** | **-2.245** |
| **478** | **ASP** | **9** | **-1.935** | **-2.747** |
| **480** | **SER** | **9** | **-1.79** | **-2.619** |
| **481** | **THR** | **6** | **-2.049** | **-2.503** |
| **482** | **GLN** | **18** | **-1.155** | **-3.092** |
| **483** | **MET** | **17** | **-0.823** | **-2.684** |
| **484** | **GLU** | **6** | **-0.42** | **-1.062** |
| **485** | **LYS** | **10** | **-0.87** | **-1.92** |
| **486** | **ILE** | **22** | **0.647** | **-1.958** |
| **487** | **LYS** | **11** | **0.75** | **-0.601** |
| **488** | **LEU** | **6** | **-0.347** | **-0.997** |
| **489** | **ALA** | **18** | **1.359** | **-0.868** |
| **490** | **LEU** | **18** | **2.455** | **0.103** |
| **491** | **ASP** | **6** | **1.973** | **1.056** |
| **492** | **MET** | **6** | **2.41** | **1.443** |
| **493** | **LEU** | **22** | **2.461** | **-0.352** |
| **494** | **ASN** | **11** | **3.388** | **1.733** |
| **495** | **GLU** | **6** | **1.879** | **0.973** |
| **496** | **GLU** | **17** | **-0.097** | **-2.041** |
| **498** | **GLU** | **6** | **-1.235** | **-1.783** |
| **541** | **TYR** | **0** | **-1.491** | **-1.32** |
| **555** | **GLY** | **3** | **-3.512** | **-3.453** |
| **556** | **ILE** | **0** | **-3.245** | **-2.872** |
| **UniProt ID -Q9KLK5** | | | | |
| **1** | **MET** | **0** | **-2.402** | **-2.126** |
| **2** | **SER** | **1** | **-2.481** | **-2.311** |
| **3** | **LEU** | **1** | **-2.512** | **-2.338** |
| **4** | **TYR** | **1** | **-2.197** | **-2.06** |
| **5** | **PHE** | **0** | **-2.928** | **-2.591** |
| **6** | **LEU** | **2** | **-2.994** | **-2.88** |
| **7** | **GLY** | **0** | **-2.228** | **-1.972** |
| **8** | **LYS** | **2** | **-2.314** | **-2.278** |
| **9** | **ASN** | **1** | **-2.613** | **-2.427** |
| **10** | **HIS** | **1** | **-2.95** | **-2.726** |
| **11** | **SER** | **2** | **-3.052** | **-2.931** |
| **13** | **ILE** | **2** | **-3.88** | **-3.664** |
| **14** | **ASN** | **2** | **-3.306** | **-3.156** |
| **15** | **LEU** | **1** | **-3.388** | **-3.113** |
| **16** | **VAL** | **3** | **-3.271** | **-3.24** |
| **17** | **TYR** | **2** | **-3.353** | **-3.197** |
| **18** | **GLY** | **1** | **-2.858** | **-2.645** |
| **19** | **ILE** | **3** | **-1.874** | **-2.004** |
| **20** | **THR** | **3** | **-0.243** | **-0.56** |
| **21** | **GLN** | **3** | **0.875** | **0.43** |
| **22** | **ASN** | **3** | **0.945** | **0.491** |
| **23** | **GLU** | **3** | **2.097** | **1.51** |
| **24** | **VAL** | **2** | **2.527** | **2.007** |
| **25** | **GLY** | **3** | **2.139** | **1.548** |
| **26** | **ASN** | **3** | **2.027** | **1.449** |
| **27** | **GLY** | **1** | **1.542** | **1.25** |
| **28** | **MET** | **5** | **0.799** | **0.133** |
| **29** | **ASN** | **3** | **0.58** | **0.168** |
| **30** | **ILE** | **0** | **-1.529** | **-1.353** |
| **31** | **GLN** | **2** | **-3.908** | **-3.689** |
| **125** | **ARG** | **6** | **-1.138** | **-1.697** |
| **127** | **GLY** | **2** | **-2.326** | **-2.288** |
| **129** | **GLY** | **0** | **-1.904** | **-1.685** |
| **130** | **ALA** | **9** | **-1.843** | **-2.666** |
| **132** | **THR** | **5** | **-2.018** | **-2.361** |
| **133** | **GLN** | **2** | **-1.864** | **-1.879** |
| **134** | **GLU** | **2** | **-1.223** | **-1.313** |
| **137** | **ASP** | **3** | **-1.916** | **-2.04** |
| **138** | **GLN** | **17** | **-1.912** | **-3.647** |
| **141** | **ARG** | **10** | **-1.386** | **-2.377** |
| **144** | **ASP** | **12** | **-2.029** | **-3.176** |
| **147** | **ALA** | **16** | **-1.915** | **-3.534** |
| **148** | **ASP** | **7** | **-0.611** | **-1.346** |
| **189** | **ALA** | **1** | **-3.039** | **-2.804** |
| **192** | **GLY** | **6** | **-2.344** | **-2.764** |
| **193** | **ASN** | **8** | **-1.826** | **-2.536** |
| **194** | **SER** | **6** | **-2.229** | **-2.663** |
| **196** | **ASN** | **1** | **-3.847** | **-3.519** |
| **262** | **LYS** | **5** | **-3.282** | **-3.479** |
| **265** | **VAL** | **20** | **-1.045** | **-3.225** |
| **266** | **LYS** | **13** | **-1.045** | **-2.42** |
| **267** | **ASP** | **22** | **-0.776** | **-3.217** |
| **268** | **ASN** | **3** | **-2.748** | **-2.777** |
| **296** | **ASP** | **14** | **1.084** | **-0.65** |
| **297** | **ILE** | **27** | **1.423** | **-1.846** |
| **298** | **GLN** | **4** | **3.288** | **2.45** |
| **299** | **THR** | **24** | **1.597** | **-1.347** |
| **300** | **GLN** | **15** | **2.612** | **0.586** |
| **301** | **ASN** | **16** | **3.057** | **0.866** |
| **302** | **ARG** | **0** | **5.008** | **4.432** |
| **303** | **ASN** | **12** | **2.681** | **0.992** |
| **304** | **ASN** | **30** | **2.302** | **-1.413** |
| **305** | **THR** | **9** | **3.212** | **1.808** |
| **306** | **TYR** | **30** | **-0.105** | **-3.543** |
| **307** | **LYS** | **16** | **-1.196** | **-2.899** |
| **335** | **ARG** | **17** | **-1.144** | **-2.967** |
| **338** | **ASN** | **12** | **3.401** | **1.63** |
| **339** | **GLY** | **14** | **1.754** | **-0.058** |
| **341** | **ASN** | **4** | **1.228** | **0.626** |
| **371** | **ASN** | **0** | **-2.472** | **-2.188** |
| **UniProt ID -Q9KU75** | | | | |
| **1** | **MET** | **0** | **0.791** | **0.7** |
| **2** | **LYS** | **1** | **0.5** | **0.327** |
| **3** | **TRP** | **2** | **-0.259** | **-0.459** |
| **4** | **PHE** | **1** | **-1.015** | **-1.013** |
| **5** | **ARG** | **1** | **-1.227** | **-1.201** |
| **6** | **PHE** | **3** | **-2.448** | **-2.512** |
| **7** | **PRO** | **2** | **-3.214** | **-3.074** |
| **58** | **THR** | **0** | **-2.942** | **-2.604** |
| **59** | **ASP** | **0** | **-2.003** | **-1.773** |
| **61** | **GLU** | **3** | **-2.478** | **-2.538** |
| **132** | **SER** | **0** | **-3.564** | **-3.154** |
| **162** | **GLN** | **9** | **0.656** | **-0.454** |
| **163** | **GLN** | **18** | **0.798** | **-1.363** |
| **164** | **ASP** | **16** | **1.066** | **-0.896** |
| **165** | **PRO** | **9** | **0.314** | **-0.757** |
| **166** | **GLN** | **3** | **-0.385** | **-0.686** |
| **182** | **ASP** | **9** | **-2.729** | **-3.451** |
| **184** | **THR** | **0** | **-3.39** | **-3.001** |
| **185** | **LYS** | **9** | **-1.607** | **-2.457** |
| **188** | **THR** | **5** | **-2.396** | **-2.695** |
| **192** | **GLU** | **9** | **-1.217** | **-2.112** |
| **195** | **GLN** | **6** | **-1.631** | **-2.133** |
| **196** | **GLN** | **10** | **-0.675** | **-1.747** |
| **198** | **ASP** | **9** | **-2.744** | **-3.464** |
| **290** | **GLU** | **6** | **-2.425** | **-2.836** |
| **291** | **GLU** | **8** | **-2.478** | **-3.113** |
| **292** | **GLN** | **6** | **-2.985** | **-3.332** |
| **293** | **LEU** | **6** | **-3.21** | **-3.531** |
| **294** | **ALA** | **6** | **-2.089** | **-2.539** |
| **295** | **ARG** | **6** | **-2.203** | **-2.64** |
| **296** | **ALA** | **6** | **-2.121** | **-2.567** |
| **297** | **LYS** | **6** | **-1.341** | **-1.877** |
| **298** | **ALA** | **6** | **-1.771** | **-2.258** |
| **299** | **GLU** | **6** | **-1.465** | **-1.987** |
| **300** | **ALA** | **6** | **-1.652** | **-2.152** |
| **301** | **MET** | **6** | **-1.642** | **-2.143** |
| **302** | **THR** | **6** | **-1.63** | **-2.132** |
| **303** | **GLN** | **6** | **-1.068** | **-1.635** |
| **UniProt ID -Q9KND9** | | | | |
| **1** | **MET** | **0** | **2.782** | **2.462** |
| **2** | **LYS** | **3** | **2.177** | **1.581** |
| **3** | **PRO** | **2** | **1.378** | **0.99** |
| **4** | **MET** | **2** | **0.087** | **-0.153** |
| **5** | **GLN** | **2** | **-1.31** | **-1.389** |
| **6** | **ARG** | **2** | **-3.18** | **-3.044** |
| **47** | **PHE** | **0** | **-3.983** | **-3.525** |
| **48** | **PHE** | **0** | **-2.147** | **-1.9** |
| **70** | **TYR** | **13** | **0.031** | **-1.467** |
| **71** | **PHE** | **15** | **2.166** | **0.192** |
| **72** | **SER** | **2** | **3.355** | **2.739** |
| **73** | **GLN** | **14** | **3.646** | **1.617** |
| **74** | **GLY** | **1** | **3.338** | **2.84** |
| **75** | **ASN** | **0** | **2.456** | **2.174** |
| **76** | **ASP** | **6** | **2.424** | **1.455** |
| **77** | **ARG** | **7** | **1.882** | **0.861** |
| **78** | **ILE** | **13** | **1.339** | **-0.31** |
| **79** | **ILE** | **10** | **0.04** | **-1.114** |
| **80** | **ILE** | **22** | **0.38** | **-2.194** |
| **81** | **ASP** | **13** | **-2.196** | **-3.438** |
| **102** | **LYS** | **3** | **1.34** | **0.841** |
| **103** | **TYR** | **27** | **2.117** | **-1.232** |
| **104** | **ARG** | **2** | **3.582** | **2.94** |
| **105** | **ASP** | **4** | **3.492** | **2.63** |
| **106** | **ALA** | **11** | **2.942** | **1.339** |
| **107** | **PRO** | **1** | **3.553** | **3.03** |
| **108** | **GLN** | **10** | **3.66** | **2.089** |
| **109** | **ALA** | **23** | **1.251** | **-1.538** |
| **110** | **THR** | **6** | **1.903** | **0.994** |
| **111** | **LYS** | **6** | **2.848** | **1.83** |
| **112** | **ALA** | **14** | **0.066** | **-1.552** |
| **114** | **LYS** | **4** | **-3.62** | **-3.664** |
| **124** | **GLN** | **8** | **-1.443** | **-2.197** |
| **125** | **GLY** | **0** | **-1.796** | **-1.59** |
| **138** | **GLU** | **0** | **-2.446** | **-2.165** |
| **139** | **GLY** | **3** | **-0.4** | **-0.699** |
| **140** | **MET** | **2** | **-1.49** | **-1.548** |
| **142** | **ILE** | **1** | **-2.367** | **-2.21** |
| **143** | **GLY** | **2** | **-2.72** | **-2.637** |
| **169** | **GLN** | **4** | **-2.909** | **-3.034** |
| **170** | **PRO** | **4** | **-3.321** | **-3.399** |
| **172** | **ALA** | **2** | **-2.491** | **-2.435** |
| **173** | **GLN** | **1** | **-1.86** | **-1.761** |
| **174** | **GLN** | **9** | **-2.427** | **-3.183** |
| **175** | **GLU** | **3** | **-2.613** | **-2.657** |
| **194** | **ASN** | **6** | **-2.746** | **-3.121** |
| **197** | **ASP** | **1** | **-1.595** | **-1.527** |
| **198** | **ALA** | **0** | **-1.09** | **-0.965** |
| **199** | **GLU** | **0** | **-0.873** | **-0.773** |
| **200** | **THR** | **9** | **-1.955** | **-2.765** |
| **201** | **LYS** | **9** | **-2.019** | **-2.822** |
| **202** | **ALA** | **3** | **-1.771** | **-1.912** |
| **203** | **ARG** | **7** | **-1.658** | **-2.273** |
| **205** | **LYS** | **8** | **-2.366** | **-3.014** |
| **206** | **ALA** | **6** | **-1.586** | **-2.093** |
| **207** | **PHE** | **8** | **-2.41** | **-3.053** |
| **209** | **ASN** | **6** | **-1.507** | **-2.024** |
| **210** | **GLN** | **6** | **-0.802** | **-1.4** |
| **211** | **GLN** | **5** | **-1.9** | **-2.256** |
| **UniProt ID -Q9KSV6** | | | | |
| **17** | **GLU** | **2** | **-0.852** | **-0.984** |
| **18** | **VAL** | **5** | **0.729** | **0.07** |
| **19** | **PRO** | **0** | **2.548** | **2.255** |
| **20** | **SER** | **1** | **3.465** | **2.951** |
| **21** | **ARG** | **3** | **4.018** | **3.211** |
| **22** | **THR** | **0** | **3.342** | **2.958** |
| **23** | **THR** | **1** | **4.247** | **3.643** |
| **24** | **LYS** | **0** | **4.492** | **3.976** |
| **25** | **ASN** | **1** | **4.889** | **4.212** |
| **26** | **ASN** | **1** | **5.342** | **4.612** |
| **27** | **ASN** | **0** | **5.516** | **4.881** |
| **28** | **SER** | **1** | **4.991** | **4.302** |
| **29** | **LYS** | **0** | **4.911** | **4.346** |
| **30** | **GLY** | **2** | **4.454** | **3.711** |
| **31** | **LYS** | **0** | **3.313** | **2.932** |
| **32** | **ILE** | **4** | **2.059** | **1.362** |
| **33** | **MET** | **1** | **0.652** | **0.462** |
| **34** | **LYS** | **0** | **-0.255** | **-0.226** |
| **35** | **ARG** | **3** | **-1.61** | **-1.77** |
| **83** | **PRO** | **0** | **-3.031** | **-2.683** |
| **84** | **ASP** | **7** | **-2.986** | **-3.447** |
| **132** | **GLN** | **5** | **-6.202** | **-6.064** |
| **142** | **SER** | **0** | **-3.992** | **-3.533** |
| **159** | **THR** | **3** | **-2.41** | **-2.478** |
| **162** | **PRO** | **3** | **-1.361** | **-1.55** |
| **UniProt ID -Q9KND3** |  |  |  |  |
| **1** | **MET** | **0** | **-0.466** | **-0.412** |
| **2** | **HIS** | **2** | **-1.137** | **-1.236** |
| **3** | **LYS** | **2** | **-2.337** | **-2.298** |
| **53** | **ALA** | **4** | **-2.637** | **-2.793** |
| **54** | **ASN** | **0** | **-0.676** | **-0.598** |
| **55** | **LEU** | **23** | **-0.702** | **-3.266** |
| **56** | **THR** | **3** | **1.612** | **1.082** |
| **57** | **GLN** | **2** | **1.681** | **1.258** |
| **58** | **SER** | **0** | **2.352** | **2.082** |
| **59** | **GLU** | **7** | **1.021** | **0.098** |
| **60** | **ILE** | **21** | **-0.498** | **-2.856** |
| **61** | **ASP** | **3** | **1.798** | **1.246** |
| **62** | **GLN** | **6** | **2.055** | **1.129** |
| **63** | **LEU** | **27** | **-0.131** | **-3.221** |
| **64** | **ASP** | **19** | **-0.427** | **-2.563** |
| **65** | **LYS** | **6** | **1.917** | **1.007** |
| **66** | **GLN** | **15** | **0.574** | **-1.217** |
| **67** | **TRP** | **24** | **-0.61** | **-3.3** |
| **68** | **ARG** | **10** | **0.316** | **-0.87** |
| **69** | **ALA** | **8** | **0.737** | **-0.268** |
| **70** | **GLU** | **21** | **0.321** | **-2.131** |
| **71** | **VAL** | **14** | **0.452** | **-1.21** |
| **72** | **GLY** | **5** | **-0.694** | **-1.189** |
| **73** | **GLN** | **14** | **0.264** | **-1.376** |
| **74** | **SER** | **0** | **0.646** | **0.571** |
| **75** | **ASP** | **1** | **0.428** | **0.264** |
| **76** | **THR** | **20** | **-0.588** | **-2.82** |
| **77** | **PRO** | **3** | **-0.928** | **-1.166** |
| **80** | **GLN** | **7** | **-2.977** | **-3.44** |
| **122** | **SER** | **6** | **-2.714** | **-3.092** |
| **152** | **GLU** | **0** | **-1.859** | **-1.645** |
| **153** | **SER** | **1** | **-1.668** | **-1.591** |
| **155** | **GLN** | **0** | **-3.098** | **-2.742** |
| **UniProt ID -Q9KPA3** | | | | |
| **144** | **HIS** | **8** | **-3.036** | **-3.607** |
| **146** | **GLN** | **6** | **-0.219** | **-0.884** |
| **147** | **ILE** | **6** | **0.563** | **-0.192** |
| **148** | **SER** | **6** | **0.255** | **-0.464** |
| **UniProt ID -Q9KT53** | | | | |
| **1** | **MET** | **3** | **-2.087** | **-2.192** |
| **69** | **GLN** | **12** | **-1.849** | **-3.016** |
| **123** | **GLU** | **7** | **-2.154** | **-2.711** |
| **124** | **LEU** | **19** | **-0.341** | **-2.487** |
| **125** | **VAL** | **4** | **0.823** | **0.268** |
| **126** | **ALA** | **4** | **2.668** | **1.901** |
| **127** | **ASN** | **2** | **3.029** | **2.45** |
| **128** | **GLN** | **2** | **3.5** | **2.867** |
| **129** | **GLN** | **2** | **3.89** | **3.212** |
| **130** | **GLU** | **1** | **3.952** | **3.383** |
| **131** | **ASN** | **2** | **4.093** | **3.392** |
| **132** | **HIS** | **0** | **2.845** | **2.518** |
| **UniProt ID -Q9KRE6** | | | | |
| **9** | **PRO** | **1** | **-3.855** | **-3.527** |
| **10** | **HIS** | **1** | **-2.357** | **-2.201** |
| **11** | **LEU** | **1** | **-1.445** | **-1.394** |
| **12** | **ALA** | **2** | **-0.215** | **-0.421** |
| **13** | **LEU** | **1** | **-0.34** | **-0.416** |
| **14** | **LYS** | **2** | **0.349** | **0.079** |
| **15** | **LYS** | **3** | **0.424** | **0.03** |
| **16** | **THR** | **1** | **0.596** | **0.413** |
| **17** | **PRO** | **2** | **0.2** | **-0.053** |
| **18** | **ASP** | **2** | **-0.419** | **-0.6** |
| **19** | **ARG** | **2** | **-0.237** | **-0.44** |
| **20** | **VAL** | **2** | **-1.043** | **-1.153** |
| **21** | **LYS** | **3** | **-1.743** | **-1.887** |
| **42** | **GLN** | **1** | **-2.487** | **-2.316** |
| **43** | **GLN** | **4** | **-1.978** | **-2.211** |
| **44** | **LEU** | **5** | **-2.991** | **-3.222** |
| **45** | **LYS** | **4** | **-0.992** | **-1.338** |
| **46** | **LYS** | **5** | **-0.299** | **-0.84** |
| **47** | **VAL** | **6** | **-2.504** | **-2.906** |
| **48** | **ILE** | **6** | **-3.236** | **-3.554** |
| **49** | **ALA** | **6** | **-2.035** | **-2.491** |
| **UniProt ID -Q9KKS6** | | | | |
| **9** | **HIS** | **0** | **-4.12** | **-3.646** |
| **11** | **LEU** | **0** | **-3.643** | **-3.224** |
| **12** | **ALA** | **3** | **-2.829** | **-2.848** |
| **13** | **SER** | **1** | **-2.162** | **-2.028** |
| **14** | **TRP** | **2** | **-2.38** | **-2.336** |
| **15** | **ARG** | **0** | **-1.729** | **-1.531** |
| **16** | **LEU** | **1** | **-2.002** | **-1.886** |
| **17** | **THR** | **4** | **-1.665** | **-1.934** |
| **18** | **ALA** | **2** | **-1.442** | **-1.506** |
| **19** | **ASN** | **4** | **-0.348** | **-0.768** |
| **20** | **THR** | **1** | **0.694** | **0.499** |
| **21** | **ASP** | **3** | **1.987** | **1.414** |
| **22** | **TYR** | **1** | **2.253** | **1.879** |
| **23** | **GLY** | **3** | **2.823** | **2.153** |
| **24** | **HIS** | **1** | **3.516** | **2.997** |
| **25** | **MET** | **6** | **4.551** | **3.338** |
| **26** | **SER** | **3** | **4.631** | **3.753** |
| **27** | **ARG** | **3** | **5.609** | **4.619** |
| **28** | **LYS** | **5** | **5.283** | **4.101** |
| **29** | **LYS** | **4** | **2.923** | **2.127** |
| **30** | **ILE** | **3** | **1.664** | **1.128** |
| **31** | **MET** | **6** | **1.021** | **0.214** |
| **32** | **LYS** | **5** | **0.304** | **-0.306** |
| **33** | **ASN** | **17** | **-1.899** | **-3.636** |
| **34** | **GLU** | **8** | **-2.553** | **-3.18** |
| **53** | **PRO** | **0** | **-3.415** | **-3.022** |
| **55** | **GLU** | **0** | **-2.722** | **-2.409** |
| **69** | **TYR** | **0** | **-1.926** | **-1.704** |
| **70** | **ASP** | **5** | **-1.48** | **-1.884** |
| **88** | **HIS** | **0** | **-2.955** | **-2.615** |
| **89** | **ASN** | **3** | **-3.534** | **-3.472** |
| **96** | **GLU** | **5** | **-3.011** | **-3.239** |
| **97** | **GLN** | **6** | **-2.143** | **-2.587** |
| **98** | **GLU** | **2** | **-2.2** | **-2.177** |
| **99** | **HIS** | **0** | **-1.937** | **-1.714** |
| **102** | **ASP** | **6** | **-3.221** | **-3.54** |
| **UniProt ID -Q9KN87** | | | | |
| **1** | **MET** | **0** | **-3.804** | **-3.366** |
| **16** | **LYS** | **10** | **-1.649** | **-2.609** |
| **17** | **PHE** | **9** | **-1.31** | **-2.195** |
| **18** | **LEU** | **13** | **-0.103** | **-1.586** |
| **19** | **LYS** | **0** | **1.592** | **1.409** |
| **20** | **THR** | **0** | **1.812** | **1.603** |
| **21** | **LYS** | **13** | **1.179** | **-0.452** |
| **22** | **ASP** | **4** | **-0.157** | **-0.599** |
| **23** | **GLU** | **10** | **-1.242** | **-2.249** |
| **69** | **PRO** | **8** | **-1.889** | **-2.591** |
| **70** | **LYS** | **10** | **-0.706** | **-1.775** |
| **71** | **ASP** | **3** | **-1.119** | **-1.335** |
| **72** | **ARG** | **15** | **-1.144** | **-2.738** |
| **98** | **GLY** | **1** | **-3.913** | **-3.578** |
| **100** | **GLY** | **0** | **-2.999** | **-2.654** |
| **109** | **TYR** | **7** | **-1.476** | **-2.111** |
| **110** | **GLN** | **13** | **0.236** | **-1.286** |
| **UniProt ID -Q9KU58** | | | | |
| **1** | **MET** | **0** | **-1.006** | **-0.891** |
| **2** | **SER** | **2** | **-1.553** | **-1.604** |
| **3** | **SER** | **3** | **-2.534** | **-2.587** |
| **4** | **ASP** | **2** | **-3.202** | **-3.064** |
| **11** | **ILE** | **1** | **-3.344** | **-3.075** |
| **12** | **PHE** | **3** | **-2.357** | **-2.431** |
| **13** | **PRO** | **4** | **-0.626** | **-1.014** |
| **14** | **SER** | **5** | **-0.112** | **-0.674** |
| **15** | **LYS** | **0** | **0.454** | **0.402** |
| **16** | **THR** | **3** | **0.907** | **0.458** |
| **17** | **THR** | **4** | **0.672** | **0.135** |
| **18** | **GLY** | **3** | **0.702** | **0.276** |
| **19** | **GLU** | **4** | **0.239** | **-0.249** |
| **20** | **ASN** | **5** | **-0.228** | **-0.777** |
| **21** | **THR** | **5** | **-1.181** | **-1.621** |
| **22** | **MET** | **5** | **-1.178** | **-1.617** |
| **23** | **ARG** | **6** | **-2.041** | **-2.496** |
| **24** | **GLN** | **6** | **-2.559** | **-2.955** |
| **40** | **ARG** | **6** | **-3.001** | **-3.346** |
| **41** | **ARG** | **6** | **-2.079** | **-2.53** |
| **42** | **GLU** | **6** | **-1.289** | **-1.83** |
| **43** | **GLU** | **6** | **-0.453** | **-1.091** |
| **44** | **ARG** | **6** | **0.258** | **-0.462** |
| **45** | **VAL** | **6** | **0.806** | **0.023** |
| **46** | **TRP** | **6** | **1.446** | **0.59** |
| **47** | **LYS** | **6** | **2.348** | **1.388** |
| **48** | **ARG** | **6** | **3.028** | **1.989** |
| **49** | **LYS** | **6** | **3.544** | **2.446** |
| **50** | **VAL** | **6** | **3.722** | **2.604** |
| **51** | **ARG** | **6** | **4.141** | **2.975** |
| **52** | **ARG** | **6** | **4.745** | **3.51** |
| **53** | **SER** | **8** | **3.863** | **2.499** |
| **54** | **SER** | **10** | **4.13** | **2.505** |
| **55** | **TYR** | **5** | **4.038** | **2.998** |
| **56** | **HIS** | **7** | **3.636** | **2.413** |
| **57** | **LEU** | **19** | **3.007** | **0.476** |
| **58** | **PRO** | **10** | **3.331** | **1.798** |
| **59** | **TRP** | **7** | **3.313** | **2.127** |
| **60** | **ASN** | **2** | **3.047** | **2.466** |
| **61** | **ASN** | **11** | **2.792** | **1.206** |
| **62** | **PRO** | **2** | **1.83** | **1.389** |
| **63** | **HIS** | **0** | **1.843** | **1.631** |
| **64** | **LEU** | **9** | **1.61** | **0.39** |
| **65** | **LEU** | **17** | **1.662** | **-0.484** |
| **66** | **ARG** | **3** | **1.144** | **0.668** |
| **67** | **ASP** | **8** | **0.786** | **-0.224** |
| **68** | **ILE** | **19** | **1.119** | **-1.194** |
| **69** | **GLY** | **4** | **-0.467** | **-0.873** |
| **70** | **LEU** | **16** | **0.081** | **-1.768** |
| **71** | **GLU** | **7** | **-0.375** | **-1.137** |
| **72** | **THR** | **4** | **1.195** | **0.597** |
| **73** | **ASP** | **5** | **0.807** | **0.139** |
| **74** | **GLY** | **6** | **1.442** | **0.586** |
| **75** | **ARG** | **6** | **0.353** | **-0.377** |
| **76** | **PRO** | **5** | **-0.534** | **-1.048** |
| **77** | **ILE** | **11** | **-1.623** | **-2.702** |
| **78** | **GLY** | **6** | **-2.492** | **-2.896** |
| **82** | **PRO** | **3** | **-3.19** | **-3.168** |
| **83** | **ASP** | **0** | **-2.967** | **-2.626** |
| **84** | **ALA** | **1** | **-3.885** | **-3.553** |
| **89** | **ARG** | **7** | **-3.193** | **-3.631** |
| **90** | **ARG** | **7** | **-3.093** | **-3.542** |
| **92** | **ARG** | **6** | **-3.246** | **-3.563** |
| **93** | **HIS** | **6** | **-2.943** | **-3.294** |
| **94** | **ILE** | **6** | **-3.13** | **-3.46** |
| **95** | **ARG** | **6** | **-2.816** | **-3.182** |
| **96** | **ARG** | **6** | **-2.671** | **-3.054** |
| **97** | **VAL** | **6** | **-2.875** | **-3.234** |
| **98** | **LEU** | **6** | **-3.141** | **-3.47** |
| **99** | **ALA** | **6** | **-2.588** | **-2.98** |
| **100** | **ALA** | **6** | **-2.123** | **-2.569** |
| **101** | **ARG** | **6** | **-1.594** | **-2.101** |
| **102** | **ILE** | **6** | **-1.425** | **-1.951** |
| **103** | **PRO** | **0** | **-1.401** | **-1.24** |
| **104** | **THR** | **2** | **-1.586** | **-1.633** |
| **UniProt ID -Q9KPP0** | | | | |
| **1** | **MET** | **1** | **-3.493** | **-3.207** |
| **10** | **GLU** | **6** | **-2.717** | **-3.095** |
| **12** | **ASN** | **3** | **-1.453** | **-1.631** |
| **13** | **GLY** | **0** | **-0.221** | **-0.195** |
| **14** | **GLU** | **3** | **-1.058** | **-1.281** |
| **15** | **GLU** | **6** | **-2.643** | **-3.029** |
| **22** | **LYS** | **1** | **-3.317** | **-3.051** |
| **23** | **ALA** | **0** | **-3.838** | **-3.397** |
| **78** | **PRO** | **9** | **-2.88** | **-3.584** |
| **79** | **ALA** | **4** | **-1.441** | **-1.735** |
| **80** | **PRO** | **4** | **-0.285** | **-0.712** |
| **81** | **ALA** | **3** | **0.436** | **0.041** |
| **82** | **THR** | **1** | **0.973** | **0.746** |
| **83** | **GLY** | **2** | **1.482** | **1.081** |
| **84** | **LYS** | **3** | **1.787** | **1.237** |
| **85** | **LYS** | **3** | **1.279** | **0.787** |
| **86** | **PRO** | **3** | **0.916** | **0.466** |
| **87** | **LYS** | **3** | **0.488** | **0.087** |
| **88** | **ALA** | **3** | **0** | **-0.345** |
| **89** | **VAL** | **2** | **0.177** | **-0.073** |
| **90** | **ALA** | **2** | **0.155** | **-0.093** |
| **91** | **ASP** | **2** | **0.04** | **-0.195** |
| **92** | **SER** | **2** | **0.01** | **-0.221** |
| **93** | **ASP** | **2** | **0.269** | **0.008** |
| **94** | **ASP** | **1** | **-0.073** | **-0.18** |
| **95** | **ASP** | **2** | **-0.495** | **-0.668** |
| **96** | **GLU** | **1** | **-0.413** | **-0.48** |
| **97** | **SER** | **2** | **-0.512** | **-0.683** |
| **98** | **VAL** | **1** | **-0.779** | **-0.804** |
| **99** | **ASP** | **1** | **-1.144** | **-1.128** |
| **100** | **ASP** | **0** | **-1.508** | **-1.334** |
| **101** | **ALA** | **0** | **-2.167** | **-1.918** |
| **102** | **ALA** | **0** | **-2.431** | **-2.152** |
| **UniProt ID -B1B1N2** | | | | |
| **1** | **MET** | **0** | **1.982** | **1.754** |
| **2** | **GLN** | **0** | **2.353** | **2.082** |
| **3** | **PRO** | **1** | **2.488** | **2.087** |
| **4** | **VAL** | **1** | **2.323** | **1.941** |
| **5** | **THR** | **2** | **2.879** | **2.318** |
| **6** | **LYS** | **0** | **3.469** | **3.07** |
| **7** | **ASN** | **2** | **3.683** | **3.029** |
| **8** | **GLY** | **1** | **3.235** | **2.748** |
| **9** | **GLU** | **2** | **2.491** | **1.974** |
| **10** | **SER** | **2** | **1.372** | **0.984** |
| **11** | **MET** | **3** | **0.056** | **-0.296** |
| **12** | **ASN** | **0** | **-0.27** | **-0.239** |
| **13** | **LYS** | **3** | **-1.752** | **-1.896** |
| **14** | **THR** | **1** | **-3.475** | **-3.19** |
| **31** | **ARG** | **2** | **-3.339** | **-3.185** |
| **32** | **GLN** | **2** | **-1.911** | **-1.921** |
| **33** | **GLU** | **3** | **0.634** | **0.216** |
| **34** | **ASN** | **2** | **1.249** | **0.876** |
| **35** | **GLU** | **4** | **0.489** | **-0.027** |
| **36** | **TYR** | **3** | **0.612** | **0.197** |
| **37** | **THR** | **1** | **0.262** | **0.116** |
| **38** | **VAL** | **9** | **0.157** | **-0.896** |
| **39** | **LYS** | **2** | **0.23** | **-0.026** |
| **40** | **GLU** | **2** | **-0.411** | **-0.594** |
| **41** | **TYR** | **10** | **-2.389** | **-3.265** |
| **42** | **THR** | **0** | **-2.144** | **-1.897** |
| **43** | **SER** | **4** | **-3.213** | **-3.303** |
| **44** | **MET** | **0** | **-3.69** | **-3.266** |
| **47** | **PRO** | **12** | **-2.027** | **-3.174** |
| **56** | **GLY** | **3** | **-3.634** | **-3.561** |
| **63** | **THR** | **1** | **-2.644** | **-2.455** |
| **64** | **GLU** | **9** | **-1.852** | **-2.674** |
| **65** | **ASN** | **1** | **0.452** | **0.285** |
| **66** | **GLU** | **0** | **0.084** | **0.074** |
| **67** | **GLN** | **7** | **-1.476** | **-2.111** |
| **68** | **ARG** | **5** | **-2.725** | **-2.986** |
| **75** | **LYS** | **6** | **-3.105** | **-3.438** |
| **76** | **ASP** | **5** | **-2.152** | **-2.48** |
| **77** | **GLY** | **0** | **-2.579** | **-2.283** |
| **78** | **GLU** | **10** | **-1.908** | **-2.839** |
| **79** | **LYS** | **6** | **-2.387** | **-2.803** |
| **80** | **ILE** | **21** | **-1.83** | **-4.035** |
| **81** | **GLU** | **9** | **-1.673** | **-2.516** |
| **82** | **GLN** | **25** | **-2.389** | **-4.989** |
| **83** | **TRP** | **6** | **-1.014** | **-1.588** |
| **84** | **GLU** | **12** | **-0.295** | **-1.641** |
| **85** | **TYR** | **29** | **-1.098** | **-4.307** |
| **86** | **PHE** | **19** | **-0.766** | **-2.863** |
| **87** | **ARG** | **7** | **0.944** | **0.03** |
| **88** | **GLN** | **13** | **1.078** | **-0.541** |
| **89** | **ASN** | **17** | **1.381** | **-0.733** |
| **90** | **HIS** | **15** | **1.723** | **-0.2** |
| **91** | **ASP** | **6** | **1.804** | **0.906** |
| **92** | **GLN** | **1** | **3.289** | **2.795** |
| **93** | **GLN** | **3** | **3.28** | **2.558** |
| **UniProt ID -Q9KL81** | | | | |
| **1** | **MET** | **0** | **-3.015** | **-2.668** |
| **2** | **TYR** | **0** | **-3.489** | **-3.088** |
| **3** | **HIS** | **0** | **-4.045** | **-3.58** |
| **19** | **PRO** | **4** | **-2.387** | **-2.573** |
| **20** | **LEU** | **13** | **-2.092** | **-3.346** |
| **21** | **PRO** | **1** | **-0.868** | **-0.883** |
| **22** | **SER** | **9** | **-0.725** | **-1.676** |
| **23** | **THR** | **3** | **-0.285** | **-0.598** |
| **24** | **PRO** | **0** | **0.099** | **0.088** |
| **25** | **ILE** | **0** | **0.049** | **0.043** |
| **26** | **ALA** | **10** | **0.137** | **-1.029** |
| **27** | **ARG** | **2** | **0.733** | **0.419** |
| **28** | **GLU** | **1** | **0.31** | **0.16** |
| **29** | **ILE** | **11** | **0.854** | **-0.509** |
| **30** | **PRO** | **2** | **1.433** | **1.038** |
| **31** | **ARG** | **9** | **-0.008** | **-1.042** |
| **32** | **SER** | **1** | **-0.541** | **-0.593** |
| **33** | **ILE** | **3** | **-0.21** | **-0.531** |
| **34** | **LYS** | **8** | **-0.026** | **-0.943** |
| **35** | **VAL** | **13** | **-1.219** | **-2.574** |
| **36** | **VAL** | **5** | **-1.153** | **-1.595** |
| **37** | **GLN** | **5** | **-0.43** | **-0.955** |
| **38** | **VAL** | **11** | **-1.091** | **-2.231** |
| **39** | **ASN** | **16** | **-1.953** | **-3.568** |
| **40** | **GLU** | **5** | **-1.534** | **-1.932** |
| **41** | **LYS** | **5** | **-0.888** | **-1.361** |
| **42** | **HIS** | **14** | **-2.247** | **-3.598** |
| **44** | **ILE** | **5** | **-2.628** | **-2.901** |
| **45** | **ARG** | **8** | **-2.661** | **-3.275** |
| **59** | **HIS** | **7** | **-2.588** | **-3.095** |
| **60** | **LYS** | **7** | **-2.713** | **-3.206** |
| **61** | **LEU** | **7** | **-2.638** | **-3.14** |
| **62** | **LEU** | **7** | **-2.079** | **-2.645** |
| **63** | **SER** | **7** | **-0.766** | **-1.483** |
| **64** | **LYS** | **7** | **-0.128** | **-0.918** |
| **65** | **GLN** | **6** | **0.557** | **-0.197** |
| **66** | **GLU** | **6** | **1.131** | **0.311** |
| **67** | **TYR** | **7** | **1.877** | **0.856** |
| **68** | **SER** | **7** | **2.172** | **1.117** |
| **69** | **ILE** | **5** | **2.683** | **1.799** |
| **70** | **ARG** | **6** | **3.351** | **2.275** |
| **71** | **LYS** | **6** | **3.595** | **2.492** |
| **72** | **PRO** | **5** | **4.671** | **3.559** |
| **73** | **GLU** | **5** | **4.479** | **3.389** |
| **74** | **ILE** | **6** | **4.19** | **3.018** |
| **75** | **LYS** | **6** | **4.24** | **3.062** |
| **76** | **LYS** | **5** | **4.675** | **3.562** |
| **77** | **ILE** | **4** | **4.14** | **3.204** |
| **78** | **LYS** | **5** | **4.075** | **3.031** |
| **79** | **SER** | **5** | **3.275** | **2.323** |
| **UniProt ID -Q9KL73** | | | | |
| **1** | **MET** | **0** | **3.077** | **2.723** |
| **2** | **LYS** | **4** | **3.762** | **2.869** |
| **3** | **ASN** | **2** | **4.188** | **3.476** |
| **4** | **LYS** | **3** | **4.62** | **3.744** |
| **5** | **ARG** | **2** | **5.019** | **4.211** |
| **6** | **VAL** | **2** | **4.65** | **3.886** |
| **7** | **LYS** | **4** | **5.071** | **4.028** |
| **8** | **LEU** | **4** | **4.967** | **3.936** |
| **9** | **PRO** | **5** | **5.336** | **4.148** |
| **10** | **LYS** | **2** | **5.74** | **4.85** |
| **11** | **LYS** | **8** | **4.874** | **3.393** |
| **12** | **ASN** | **4** | **5.259** | **4.194** |
| **13** | **LYS** | **7** | **4.158** | **2.874** |
| **14** | **LYS** | **2** | **3.684** | **3.031** |
| **15** | **GLY** | **4** | **3.198** | **2.37** |
| **16** | **ALA** | **5** | **2.857** | **1.954** |
| **17** | **TYR** | **12** | **1.75** | **0.169** |
| **18** | **GLU** | **4** | **1.084** | **0.499** |
| **19** | **ALA** | **6** | **-0.041** | **-0.726** |
| **20** | **LYS** | **17** | **-0.563** | **-2.453** |
| **21** | **PHE** | **15** | **-1.48** | **-3.035** |
| **22** | **GLU** | **7** | **-2.276** | **-2.819** |
| **23** | **GLU** | **6** | **-2.749** | **-3.123** |
| **60** | **GLU** | **8** | **-2.955** | **-3.535** |
| **61** | **ARG** | **7** | **-0.954** | **-1.649** |
| **62** | **PHE** | **20** | **-0.074** | **-2.365** |
| **63** | **PHE** | **11** | **0.218** | **-1.072** |
| **64** | **LYS** | **6** | **1.057** | **0.246** |
| **65** | **GLN** | **10** | **1.793** | **0.437** |
| **66** | **ASN** | **17** | **2.816** | **0.537** |
| **67** | **GLN** | **7** | **3.775** | **2.536** |
| **UniProt ID -Q9KNG0** |  |  |  |  |
| **1** | **MET** | **0** | **0.707** | **0.626** |
| **2** | **SER** | **3** | **1.191** | **0.709** |
| **3** | **ILE** | **3** | **1.607** | **1.077** |
| **4** | **ASN** | **3** | **1.713** | **1.171** |
| **5** | **SER** | **2** | **2.33** | **1.832** |
| **6** | **ILE** | **4** | **2.71** | **1.938** |
| **7** | **ASP** | **5** | **3.205** | **2.261** |
| **8** | **HIS** | **2** | **3.389** | **2.77** |
| **9** | **ASP** | **3** | **3.63** | **2.867** |
| **10** | **ASP** | **4** | **3.419** | **2.566** |
| **11** | **MET** | **4** | **3.29** | **2.452** |
| **12** | **THR** | **4** | **3.297** | **2.457** |
| **13** | **ASN** | **5** | **3.712** | **2.71** |
| **14** | **ILE** | **6** | **3.733** | **2.614** |
| **15** | **ALA** | **6** | **3.539** | **2.442** |
| **16** | **ASN** | **6** | **3.949** | **2.805** |
| **17** | **LYS** | **6** | **4.532** | **3.32** |
| **18** | **TRP** | **6** | **4.453** | **3.251** |
| **19** | **ASP** | **6** | **4.48** | **3.275** |
| **20** | **SER** | **6** | **4.585** | **3.368** |
| **21** | **ILE** | **6** | **4.134** | **2.968** |
| **22** | **GLU** | **6** | **3.959** | **2.814** |
| **23** | **GLU** | **6** | **3.724** | **2.606** |
| **24** | **ILE** | **6** | **3.564** | **2.464** |
| **25** | **GLU** | **6** | **3.778** | **2.653** |
| **26** | **SER** | **7** | **3.995** | **2.731** |
| **27** | **GLN** | **7** | **4.231** | **2.94** |
| **28** | **ARG** | **7** | **3.917** | **2.662** |
| **29** | **PRO** | **0** | **3.882** | **3.435** |
| **30** | **THR** | **2** | **3.125** | **2.536** |
| **31** | **LYS** | **7** | **3.098** | **1.936** |
| **32** | **ASN** | **8** | **2.867** | **1.618** |
| **33** | **LEU** | **3** | **1.778** | **1.229** |
| **34** | **LYS** | **6** | **1.004** | **0.199** |
| **35** | **SER** | **8** | **1.109** | **0.062** |
| **36** | **ALA** | **7** | **0.696** | **-0.189** |
| **37** | **GLU** | **8** | **-0.33** | **-1.212** |
| **38** | **ALA** | **6** | **-0.531** | **-1.16** |
| **39** | **ARG** | **6** | **-0.201** | **-0.868** |
| **40** | **ARG** | **11** | **-1.049** | **-2.194** |
| **41** | **ARG** | **12** | **-1.893** | **-3.055** |
| **42** | **ILE** | **6** | **-1.762** | **-2.25** |
| **43** | **GLU** | **7** | **-1.973** | **-2.551** |
| **46** | **ARG** | **6** | **-2.903** | **-3.259** |
| **49** | **ARG** | **6** | **-3.131** | **-3.461** |
| **50** | **GLU** | **6** | **-2.661** | **-3.045** |
| **52** | **GLY** | **0** | **-2.998** | **-2.653** |
| **54** | **THR** | **3** | **-3.68** | **-3.602** |
| **56** | **GLU** | **0** | **-3.924** | **-3.473** |
| **UniProt ID -Q9KPZ1** | | | | |
| **38** | **LYS** | **7** | **-2.88** | **-3.353** |
| **39** | **VAL** | **6** | **-2.488** | **-2.891** |
| **40** | **MET** | **7** | **-1.487** | **-2.121** |
| **41** | **THR** | **7** | **-0.609** | **-1.344** |
| **42** | **ASP** | **7** | **-0.063** | **-0.861** |
| **43** | **LYS** | **6** | **0.77** | **-0.008** |
| **44** | **SER** | **3** | **1.471** | **0.957** |
| **45** | **PRO** | **1** | **2.12** | **1.761** |
| **46** | **HIS** | **0** | **2.17** | **1.921** |
| **UniProt ID -Q9KNI6** |  |  |  |  |
| **1** | **MET** | **0** | **-2.269** | **-2.008** |
| **2** | **ILE** | **0** | **-2.057** | **-1.821** |
| **3** | **GLN** | **0** | **-2.432** | **-2.153** |
| **4** | **PHE** | **1** | **-2.464** | **-2.295** |
| **5** | **GLY** | **2** | **-2.053** | **-2.047** |
| **6** | **VAL** | **3** | **-2.524** | **-2.579** |
| **7** | **LEU** | **4** | **-2.806** | **-2.943** |
| **8** | **GLY** | **5** | **-2.325** | **-2.632** |
| **9** | **GLY** | **5** | **-2.217** | **-2.537** |
| **10** | **GLU** | **6** | **-2.448** | **-2.856** |
| **11** | **ILE** | **6** | **-2.4** | **-2.814** |
| **12** | **ALA** | **6** | **-2.277** | **-2.705** |
| **13** | **ASN** | **7** | **-2.754** | **-3.242** |
| **14** | **GLU** | **7** | **-2.869** | **-3.344** |
| **15** | **ASN** | **6** | **-2.457** | **-2.864** |
| **16** | **LYS** | **7** | **-2.595** | **-3.102** |
| **35** | **GLU** | **6** | **-3.315** | **-3.624** |
| **36** | **ALA** | **6** | **-2.91** | **-3.266** |
| **37** | **ASP** | **6** | **-2.359** | **-2.777** |
| **38** | **ARG** | **6** | **-1.791** | **-2.275** |
| **39** | **GLN** | **6** | **-1.582** | **-2.09** |
| **40** | **GLU** | **6** | **-1.193** | **-1.746** |
| **41** | **GLU** | **6** | **-0.936** | **-1.518** |
| **42** | **ARG** | **6** | **-0.412** | **-1.054** |
| **43** | **SER** | **6** | **-0.461** | **-1.098** |
| **44** | **LEU** | **6** | **-0.935** | **-1.518** |
| **45** | **THR** | **5** | **-0.705** | **-1.199** |
| **46** | **ILE** | **5** | **-0.208** | **-0.759** |
| **UniProt ID -Q9KVT0** |  |  |  |  |
| **1** | **MET** | **0** | **-2.338** | **-2.069** |
| **2** | **ILE** | **0** | **-2.319** | **-2.052** |
| **3** | **GLN** | **0** | **-2.421** | **-2.142** |
| **4** | **LEU** | **1** | **-2.498** | **-2.326** |
| **5** | **GLY** | **2** | **-1.954** | **-1.959** |
| **6** | **VAL** | **3** | **-2.282** | **-2.364** |
| **7** | **LEU** | **4** | **-2.559** | **-2.724** |
| **8** | **GLY** | **5** | **-2.007** | **-2.352** |
| **9** | **ARG** | **4** | **-1.689** | **-1.955** |
| **10** | **GLU** | **6** | **-1.724** | **-2.215** |
| **11** | **ILE** | **6** | **-1.973** | **-2.436** |
| **12** | **ALA** | **6** | **-1.793** | **-2.277** |
| **13** | **ASN** | **6** | **-1.929** | **-2.397** |
| **14** | **GLU** | **6** | **-2.389** | **-2.804** |
| **15** | **ASN** | **6** | **-1.997** | **-2.457** |
| **16** | **LYS** | **6** | **-1.98** | **-2.442** |
| **17** | **LEU** | **6** | **-2.951** | **-3.302** |
| **18** | **LYS** | **6** | **-3.2** | **-3.522** |
| **19** | **LYS** | **6** | **-2.981** | **-3.328** |
| **45** | **THR** | **5** | **-3.462** | **-3.639** |
| **46** | **ILE** | **3** | **-2.574** | **-2.623** |
