# Supplementary material for: Comprehensive in silico analyses of fifty-one uncharacterized proteins from Vibrio cholerae
Source: PLoS One. 2024 Oct 4;19(10):e0311301. doi: 10.1371/journal.pone.0311301 (PMC11452002; doi:10.1371/journal.pone.0311301)
Supplement: S14 Table — (DOCX) [file pone.0311301.s014.docx]

**Table S14**

**Prediction of MHC-I binding of candidate proteins by NetCTL**

| **S.no** | **Start** | **End** | **Sequence** | **Value** |
| --- | --- | --- | --- | --- |
| **UniProt ID- Q9KRD2** | | | | |
| 1. | 11 | 19 | QSIGLKTRY | 1.1051 |
| 2. | 18 | 26 | RYAPLDWCY | 0.8912 |
| 3. | 27 | 35 | QLASQHMDY | 2.1422 |
| 4. | 141 | 149 | ITDDQTRIL | 1.0496 |
| 5. | 184 | 192 | YVDMWQQLR | 0.9156 |
| 6. | 233 | 241 | TTDLAPHLD | 0.8032 |
| 7. | 243 | 251 | VLDKQAGDV | 0.7949 |
| 8. | 263 | 271 | FSDQLKQRL | 1.1642 |
| 9. | 289 | 297 | SSDKYYAQI | 1.3613 |
| 10. | 375 | 383 | DTDKIERLV | 0.8197 |
| 11. | 438 | 446 | IVDSQSWSV | 1.0826 |
| 12. | 513 | 521 | NSDLSAEQI | 0.8143 |
| 13. | 540 | 548 | TVELTETAY | 1.6594 |
| 14. | 570 | 578 | AIDDFGTGF | 0.9114 |
| 15. | 575 | 583 | GTGFSSFSY | 1.8332 |
| 16. | 583 | 591 | YLTECQFDY | 1.5714 |
| 17. | 584 | 592 | LTECQFDYL | 1.0580 |
| 18. | 601 | 609 | NIEVGSRRY | 1.1169 |
| 19. | 615 | 623 | VTDLAHTLG | 0.7963 |
| 20. | 630 | 638 | GVETEHEVY | 2.0103 |
| 21. | 644 | 652 | GVDLLQGFF | 1.0298 |
| 22. | 665 | 673 | ASDYRKHLK | 1.0407 |
| 23. | 690 | 698 | QSLAIFTQY | 1.5519 |
| 24. | 732 | 740 | LVDRAALNL | 0.7884 |
| 25. | 795 | 803 | WVLVEGNKY | 1.1011 |
| 26. | 807 | 815 | VTAPRLLAY | 3.1245 |
| **UniProt ID- Q9KVG3** | | | | |
| 1. | 78 | 86 | VSDDLAREM | 0.8424 |
| 2. | 87 | 95 | KLICFCWLY | 1.2031 |
| 3. | 114 | 122 | RFSKLSILY | 0.8482 |
| 4. | 137 | 145 | SSEIVFSEF | 1.6083 |
| 5. | 140 | 148 | IVFSEFCEY | 0.8748 |
| 6. | 155 | 163 | SSGQVEGIY | 1.9789 |
| 7. | 199 | 207 | LYEGKNQFY | 1.0829 |
| 8. | 212 | 220 | RLMEKIYRY | 0.9768 |
| 9. | 220 | 228 | YCFDVVETY | 0.9893 |
| 10. | 232 | 240 | KEEIHELLY | 0.7952 |
| 11. | 238 | 246 | LLYDMRQNY | 0.8064 |
| 12. | 264 | 272 | WISEESNEY | 2.4630 |
| 13. | 284 | 292 | YSSIIDAHI | 0.8377 |
| 14. | 340 | 348 | NSFKEKEMY | 1.4117 |
| 15. | 369 | 377 | MAEWVTTKF | 0.9114 |
| 16. | 383 | 391 | ELAEAISRY | 1.2873 |
| 17. | 445 | 453 | ITEDDLDEF | 0.8007 |
| 18. | 529 | 537 | QIDTELQSF | 0.8346 |
| 19. | 546 | 554 | TTQKIHEWY | 2.8491 |
| 20. | 635 | 643 | KAENWDKRY | 1.2576 |
| 21. | 659 | 667 | LTSSMYSHF | 1.6775 |
| 22. | 550 | 668 | TSSMYSHFI | 1.0189 |
| **UniProt ID- Q9KT38** | | | | |
| 1. | 7 | 17 | KCAAKNTTY | 0.9839 |
| 2. | 9 | 17 | AAKNTTYHY | 0.9141 |
| 3. | 18 | 26 | STSMLLNAF | 1.0804 |
| 4. | 43 | 51 | SLAATSTTY | 2.0729 |
| 5. | 74 | 82 | NLEIEYVNY | 1.7972 |
| 6. | 105 | 113 | YTDLRAQRF | 2.7280 |
| 7. | 115 | 123 | FSRPTNIEY | 2.0845 |
| 8. | 119 | 127 | TNIEYTYLY | 0.7573 |
| 9. | 150 | 158 | TLLKEHYPY | 0.8666 |
| 10. | 182 | 190 | VVDAINQLK | 0.7519 |
| 11. | 340 | 348 | SQQRKNLAY | 1.2343 |
| 12. | 345 | 353 | NLAYFSESY | 1.7553 |
| 13. | 349 | 357 | FSESYYHPQ | 0.7629 |
| 14. | 448 | 456 | TMIGSFYQY | 0.8745 |
| 15. | 483 | 491 | NTEQIIHTY | 3.5971 |
| 16. | 520 | 528 | IVLFMVAFY | 0.7756 |
| 17. | 534 | 542 | HTDNLTKLR | 0.9005 |
| 18. | 555 | 563 | LSPRLSLVY | 2.0416 |
| **UniProt ID- Q9KKL8** | | | | |
| 1. | 47 | 55 | ILDEEVTRF | 0.9107 |
| 2. | 91 | 99 | ASMVVTGAY | 2.3493 |
| 3. | 273 | 282 | SVATGHIRF | 0.7600 |
| 4. | 360 | 368 | AADSIHLGV | 0.9121 |
| 5. | 414 | 422 | DTATHVEAF | 0.9611 |
| 6. | 431 | 439 | RINKHKELY | 1.0883 |
| 7. | 464 | 472 | RSDEEAQEI | 1.0481 |
| 8. | 489 | 497 | ALDMLNEEF | 1.2570 |
| 9. | 533 | 541 | ASIFKFNQY | 1.1833 |
| **UniProt ID- Q9KLK5** | | | | |
| 1. | 9 | 17 | NHSCINLVY | 0.8433 |
| 2. | 45 | 53 | SSSAFASNL | 0.7939 |
| 3. | 46 | 54 | SSAFASNLL | 0.8591 |
| 4. | 64 | 72 | NTGVSTADY | 2.4258 |
| 5. | 69 | 77 | TADYLLAPY | 2.2724 |
| 6. | 146 | 154 | LADDKPLAV | 1.0887 |
| 7. | 165 | 173 | PLDAVSLNF | 1.0109 |
| 8. | 170 | 178 | SLNFFTRGY | 0.8807 |
| 9. | 194 | 202 | SANEVKTRY | 1.2546 |
| 10. | 204 | 212 | SSDVDLTAF | 1.6201 |
| 11. | 206 | 214 | DVDLTAFGY | 2.1531 |
| 12. | 209 | 217 | LTAFGYTEV | 0.8575 |
| 13. | 262 | 270 | KSEVKDNSF | 0.8255 |
| 14. | 298 | 306 | QTQNRNNTY | 3.2134 |
| 15. | 319 | 327 | VSDFFIAAV | 1.4108 |
| 16. | 389 | 397 | VSLDLAGSY | 1.6646 |
| 17. | 405 | 413 | LSANLAFTF | 1.2437 |
| **UniProt ID- Q9KU75** | | | | |
| 1. | 23 | 31 | NESATQWLY | 1.1692 |
| 2. | 68 | 76 | MLFERGNYY | 1.2538 |
| 3. | 112 | 120 | EAGEFDAAY | 0.8823 |
| 4. | 153 | 161 | ALEEITKHY | 1.3437 |
| 5. | 209 | 217 | MLDQLSEEV | 1.1568 |
| 6. | 232 | 240 | LAQRLTEAY | 1.0808 |
| 7. | 252 | 260 | ELAHALSLY | 1.4757 |
| 8. | 261 | 269 | KLAISFNVY | 1.3386 |
| 9. | 263 | 271 | AISFNVYEY | 1.4343 |
| 10. | 268 | 276 | VYEYVEHRY | 0.7944 |
| **UniProt ID- Q9KND9** | | | | |
| 1. | 7 | 15 | LTCLLALCF | 1.0518 |
| 2. | 81 | 89 | DSEVVIATF | 0.8895 |
| 3. | 145 | 153 | NFEFETAEY | 0.7704 |
| 4. | 183 | 191 | AAAEEMLHF | 0.7660 |
| **UniProt ID- Q9KVJ9** | | | | |
| 1. | 173 | 181 | ATLLAIHDY | 1.7722 |
| **UniProt ID- Q9KSV6** | | | | |
| 1. | 42 | 50 | MSVVSASAY | 2.2662 |
| 2. | 63 | 71 | YIDASLNWY | 3.1550 |
| 3. | 85 | 93 | LAEVSQWFL | 0.8542 |
| 4. | 92 | 100 | FLEGRQHHF | 0.7555 |
| 5. | 101 | 109 | ALNRAAVHY | 0.9731 |
| 6. | 180 | 188 | QIESALNRY | 1.7619 |
| **UniProt ID- Q9KND3** | | | | |
| 1. | 12 | 20 | SLPVMANDY | 1.2021 |
| 2. | 107 | 115 | VMDNKGLNV | 1.1445 |
| 3. | 116 | 124 | AQSEVTSDY | 1.3166 |
| 4. | 121 | 129 | TSDYWQGDE | 0.8145 |
| 5. | 155 | 163 | QVFQAQVSY | 0.7941 |
| **UniProt ID- Q9KRE6** | | | | |
| 1. | 56 | 64 | HIDELNLLL | 1.5776 |
| 2. | 85 | 93 | VQAAVVRLY | 1.0165 |
| 3. | 106 | 114 | LTDEGIEIA | 0.8029 |
| **UniProt ID- Q9KKS6** | | | | |
| 1. | 87 | 95 | FHNTYHLAY | 1.1545 |
| **UniProt ID- Q9KN87** | | | | |
| 1. | 86 | 94 | VNDPHADQY | 1.0745 |
| **UniProt ID- Q9KU58** | | | | |
| 1. | 1 | 9 | MSSDFSLSI | 1.2022 |
| 2. | 2 | 10 | SSDFSLSIV | 1.4041 |
| 3. | 27 | 35 | YLQLAVLLV | 0.7881 |
| **UniProt ID- Q9KPP0** | | | | |
| 1. | 20 | 28 | TSKAEADAY | 1.6939 |
| 2. | 31 | 39 | MLDMADELF | 1.6747 |
| 3. | 34 | 42 | MADELFELL | 1.0191 |
| **UniProt ID- B1B1N2** | | | | |
| 1. | 43 | 51 | SMANPASVY | 1.6493 |
| **UniProt ID- Q9KL73** | | | | |
| 1. | 37 | 45 | EMSVGSEEY | 1.6409 |
| **UniProt ID- Q9KPZ1** | | | | |
| 1. | 23 | 31 | GALGLMLFY | 1.1281 |

NetCTL prediction depends on the binding affinity of MHCI, C-terminal cleavage function, and transporter function associated with antigen processing. The combined threshold value for MHC-I prediction is 0.75.
