# Supplementary material for: Comprehensive in silico analyses of fifty-one uncharacterized proteins from Vibrio cholerae
Source: PLoS One. 2024 Oct 4;19(10):e0311301. doi: 10.1371/journal.pone.0311301 (PMC11452002; doi:10.1371/journal.pone.0311301)
Supplement: S15 Table — (DOCX) [file pone.0311301.s015.docx]

**Table S15**

**Prediction of binding affinity between MHC-II and candidate uncharacterized protein depicted by scores generated from IEDB MHC II**

| **S.no** | **Start** | **End** | **Length** | **Sequence** | **Score** |
| --- | --- | --- | --- | --- | --- |
| **UniProt ID- Q9KRD2** | | | | | |
| 1. | 88 | 102 | 15 | AEKLVALVAFSVDEV | 0.38 |
| 2. | 89 | 103 | 15 | EKLVALVAFSVDEVS | 0.38 |
| 3. | 85 | 99 | 15 | LVSAEKLVALVAFSV | 0.38 |
| 4. | 87 | 101 | 15 | SAEKLVALVAFSVDE | 0.38 |
| 5. | 86 | 100 | 15 | VSAEKLVALVAFSVD | 0.38 |
| 6. | 23 | 37 | 15 | SREITTLQSLAIFTQ | 1.7 |
| 7. | 15 | 29 | 15 | KIHALFGHHVGVSIN | 1.9 |
| 8. | 35 | 49 | 15 | HEVYVLKSLGVDLLQ | 2 |
| 9. | 34 | 48 | 15 | EHEVYVLKSLGVDLL | 2.1 |
| 10. | 14 | 28 | 15 | PKIHALFGHHVGVSI | 2.1 |
| 11. | 22 | 36 | 15 | QSREITTLQSLAIFT | 2.1 |
| **UniProt ID- Q9KVG3** | | | | | |
| 1. | 23 | 37 | 15 | KNQFYAIPTRLMEKI | 0.47 |
| 2. | 22 | 36 | 15 | GKNQFYAIPTRLMEK | 0.52 |
| 3. | 8 | 22 | 15 | KGFQSINSLSSEIVF | 0.91 |
| 4. | 21 | 35 | 15 | EGKNQFYAIPTRLME | 0.99 |
| 5. | 24 | 38 | 15 | NQFYAIPTRLMEKIY | 1.1 |
| 6. | 20 | 34 | 15 | YEGKNQFYAIPTRLM | 1.6 |
| 7. | 7 | 21 | 15 | SKGFQSINSLSSEIV | 1.8 |
| **UniProt ID- Q9KT38** | | | | | |
| 1. | 23 | 37 | 15 | KNQFYAIPTRLMEKI | 0.47 |
| 2. | 22 | 36 | 15 | GKNQFYAIPTRLMEK | 0.52 |
| 3. | 8 | 22 | 15 | KGFQSINSLSSEIVF | 0.91 |
| 4. | 21 | 35 | 15 | EGKNQFYAIPTRLME | 0.99 |
| 5. | 24 | 38 | 15 | NQFYAIPTRLMEKIY | 1.1 |
| 6. | 20 | 34 | 15 | YEGKNQFYAIPTRLM | 1.6 |
| 7. | 7 | 21 | 15 | SKGFQSINSLSSEIV | 1.8 |
| **UniProt ID- Q9KKL8** | | | | | |
| 1. | 23 | 37 | 15 | KNQFYAIPTRLMEKI | 0.47 |
| 2. | 22 | 36 | 15 | GKNQFYAIPTRLMEK | 0.52 |
| 3. | 8 | 22 | 15 | KGFQSINSLSSEIVF | 0.91 |
| 4. | 21 | 35 | 15 | EGKNQFYAIPTRLME | 0.99 |
| 5. | 24 | 38 | 15 | NQFYAIPTRLMEKIY | 1.1 |
| 6. | 20 | 34 | 15 | YEGKNQFYAIPTRLM | 1.6 |
| 7. | 7 | 21 | 15 | SKGFQSINSLSSEIV | 1.8 |
| **UniProt ID- Q9KU75** | | | | | |
| 1. | 9 | 23 | 15 | AVIGLVLLSGCATQN | 0.41 |
| 2. | 12 | 26 | 15 | GLVLLSGCATQNESA | 0.41 |
| 3. | 8 | 22 | 15 | IAVIGLVLLSGCATQ | 0.41 |
| 4. | 11 | 25 | 15 | IGLVLLSGCATQNES | 0.41 |
| 5. | 13 | 27 | 15 | LVLLSGCATQNESAT | 0.41 |
| 6. | 7 | 21 | 15 | PIAVIGLVLLSGCAT | 0.41 |
| 7. | 10 | 24 | 15 | VIGLVLLSGCATQNE | 0.41 |
| **UniProt ID- Q9KND9** | | | | | |
| 1. | 2 | 16 | 15 | KPMQRLTCLLALCFA | 0.91 |
| 2. | 1 | 15 | 15 | MKPMQRLTCLLALCF | 0.91 |
| 3. | 4 | 18 | 15 | MQRLTCLLALCFAAS | 0.91 |
| 4. | 3 | 17 | 15 | PMQRLTCLLALCFAA | 0.91 |
| **UniProt ID- Q9KVJ9** | | | | | |
| 1. | 106 | 120 | 15 | CLVYILSATLLAIHD | 0.23 |
| 2. | 103 | 117 | 15 | FLGCLVYILSATLLA | 0.23 |
| 3. | 105 | 119 | 15 | GCLVYILSATLLAIH | 0.23 |
| 4. | 102 | 116 | 15 | GFLGCLVYILSATLL | 0.23 |
| 5. | 104 | 118 | 15 | LGCLVYILSATLLAI | 0.23 |
| 6. | 107 | 121 | 15 | LVYILSATLLAIHDY | 0.23 |
| 7. | 108 | 122 | 15 | VYILSATLLAIHDYR | 0.23 |
| 8. | 19 | 33 | 15 | ASFVLFLVAQLLFSK | 0.46 |
| 9. | 21 | 35 | 15 | FVLFLVAQLLFSKAF | 0.46 |
| 10. | 17 | 31 | 15 | HKASFVLFLVAQLLF | 0.46 |
| 11. | 18 | 32 | 15 | KASFVLFLVAQLLFS | 0.46 |
| 12. | 20 | 34 | 15 | SFVLFLVAQLLFSKA | 0.46 |
| 13. | 22 | 36 | 15 | VLFLVAQLLFSKAFW | 0.46 |
| 14. | 16 | 30 | 15 | YHKASFVLFLVAQLL | 0.46 |
| 15. | 101 | 115 | 15 | AGFLGCLVYILSATL | 0.53 |
| **UniProt ID- Q9KSV6** | | | | | |
| 1. | 97 | 111 | 15 | QHHFALNRAAVHYYL | 0.86 |
| 2. | 98 | 112 | 15 | HHFALNRAAVHYYLQ | 1.3 |
| 3. | 96 | 110 | 15 | RQHHFALNRAAVHYY | 1.4 |
| **UniProt ID- Q9KND3** | | | | | |
| 1. | 2 | 16 | 15 | HKSLALFILASLPVM | 0.67 |
| 2. | 3 | 17 | 15 | KSLALFILASLPVMA | 0.67 |
| 3. | 5 | 19 | 15 | LALFILASLPVMAND | 0.67 |
| 4. | 4 | 18 | 15 | SLALFILASLPVMAN | 0.67 |
| 5. | 1 | 15 | 15 | MHKSLALFILASLPV | 0.91 |
| 6. | 6 | 20 | 15 | ALFILASLPVMANDY | 1.6 |
| **UniProt ID- Q9KPA3** | | | | | |
| 1. | 21 | 35 | 15 | FIPSFYAQMIALLIM | 0.01 |
| 2. | 22 | 36 | 15 | IPSFYAQMIALLIML | 0.01 |
| 3. | 20 | 34 | 15 | LFIPSFYAQMIALLI | 0.01 |
| 4. | 23 | 37 | 15 | PSFYAQMIALLIMLL | 0.01 |
| 5. | 24 | 38 | 15 | SFYAQMIALLIMLLM | 0.01 |
| 6. | 6 | 20 | 15 | IRLSLITSAKSVKLL | 0.14 |
| 7. | 7 | 21 | 15 | RLSLITSAKSVKLLF | 0.14 |
| 8. | 25 | 39 | 15 | FYAQMIALLIMLLML | 0.16 |
| 9. | 5 | 19 | 15 | WIRLSLITSAKSVKL | 0.44 |
| 10. | 33 | 47 | 15 | LIMLLMLWALFVSSI | 0.56 |
| 11. | 31 | 45 | 15 | ALLIMLLMLWALFVS | 0.67 |
| 12. | 30 | 44 | 15 | IALLIMLLMLWALFV | 0.67 |
| 13. | 34 | 48 | 15 | IMLLMLWALFVSSIP | 0.67 |
| 14. | 32 | 46 | 15 | LLIMLLMLWALFVSS | 0.67 |
| 15. | 4 | 18 | 15 | WWIRLSLITSAKSVK | 0.96 |
| 16. | 38 | 52 | 15 | MLWALFVSSIPLVLV | 1.8 |
| 17. | 41 | 55 | 15 | ALFVSSIPLVLVPYF | 2 |
| 18. | 19 | 33 | 15 | LLFIPSFYAQMIALL | 2 |
| 19. | 39 | 53 | 15 | LWALFVSSIPLVLVP | 2 |
| 20. | 3 | 17 | 15 | RWWIRLSLITSAKSV | 2.1 |
| 21. | 37 | 51 | 15 | LMLWALFVSSIPLVL | 2.2 |
| 22. | 40 | 54 | 15 | WALFVSSIPLVLVPY | 2.2 |
| **UniProt ID- Q9KT53** | | | | | |
| 1. | 21 | 35 | 15 | FIPSFYAQMIALLIM | 0.01 |
| 2. | 22 | 36 | 15 | IPSFYAQMIALLIML | 0.01 |
| 3. | 20 | 34 | 15 | LFIPSFYAQMIALLI | 0.01 |
| 4. | 23 | 37 | 15 | PSFYAQMIALLIMLL | 0.01 |
| 5. | 24 | 38 | 15 | SFYAQMIALLIMLLM | 0.01 |
| 6. | 24 | 34 | 11 | SFYAQMIALLI | 0.06 |
| 7. | 6 | 20 | 15 | IRLSLITSAKSVKLL | 0.14 |
| 8. | 7 | 21 | 15 | RLSLITSAKSVKLLF | 0.14 |
| 9. | 25 | 39 | 15 | FYAQMIALLIMLLML | 0.16 |
| 10. | 5 | 19 | 15 | WIRLSLITSAKSVKL | 0.44 |
| 11. | 25 | 35 | 11 | FYAQMIALLIM | 0.46 |
| 12. | 33 | 47 | 15 | LIMLLMLWALFVSSI | 0.56 |
| 13. | 31 | 45 | 15 | ALLIMLLMLWALFVS | 0.67 |
| 14. | 30 | 44 | 15 | IALLIMLLMLWALFV | 0.67 |
| 15. | 34 | 48 | 15 | IMLLMLWALFVSSIP | 0.67 |
| 16. | 32 | 46 | 15 | LLIMLLMLWALFVSS | 0.67 |
| 17. | 4 | 18 | 15 | WWIRLSLITSAKSVK | 0.96 |
| 18. | 7 | 17 | 11 | RLSLITSAKSV | 1.03 |
| 19. | 34 | 44 | 11 | IMLLMLWALFV | 1.26 |
| 20. | 38 | 52 | 15 | MLWALFVSSIPLVLV | 1.8 |
| 21. | 41 | 55 | 15 | ALFVSSIPLVLVPYF | 2 |
| 22. | 19 | 33 | 15 | LLFIPSFYAQMIALL | 2 |
| 23. | 39 | 53 | 15 | LWALFVSSIPLVLVP | 2 |
| 24. | 3 | 17 | 15 | RWWIRLSLITSAKSV | 2.1 |
| 25. | 37 | 51 | 15 | LMLWALFVSSIPLVL | 2.2 |
| 26. | 40 | 54 | 15 | WALFVSSIPLVLVPY | 2.2 |
| **UniProt ID- Q9KU58** | | | | | |
| 1. | 22 | 36 | 15 | MRQSIYLQLAVLLVR | 0.01 |
| 2. | 24 | 38 | 15 | QSIYLQLAVLLVRAE | 0.01 |
| 3. | 23 | 37 | 15 | RQSIYLQLAVLLVRA | 0.01 |
| 4. | 25 | 39 | 15 | SIYLQLAVLLVRAEL | 0.01 |
| 5. | 21 | 35 | 15 | TMRQSIYLQLAVLLV | 0.01 |
| 6. | 26 | 40 | 15 | IYLQLAVLLVRAELR | 0.38 |
| 7. | 88 | 102 | 15 | ERRVRHIRRVLAARI | 0.67 |
| 8. | 89 | 103 | 15 | RRVRHIRRVLAARIP | 0.91 |
| 9. | 90 | 104 | 15 | RVRHIRRVLAARIPT | 0.91 |
| 10. | 20 | 34 | 15 | NTMRQSIYLQLAVLL | 1.3 |
| **UniProt ID- B1B1N2** | | | | | |
| 1. | 39 | 53 | 15 | KEYTSMANPASVYCV | 0.91 |
| 2. | 37 | 51 | 15 | TVKEYTSMANPASVY | 0.91 |
| 3. | 38 | 52 | 15 | VKEYTSMANPASVYC | 0.91 |
| 4. | 15 | 29 | 15 | ALLLTVVGAVLLTGC | 1.1 |
| 5. | 13 | 27 | 15 | KTALLLTVVGAVLLT | 1.1 |
| 6. | 14 | 28 | 15 | TALLLTVVGAVLLTG | 1.1 |
| 7. | 12 | 26 | 15 | NKTALLLTVVGAVLL | 1.3 |
| 8. | 36 | 50 | 15 | YTVKEYTSMANPASV | 1.6 |
| 9. | 16 | 30 | 15 | LLLTVVGAVLLTGCA | 2.2 |
| **UniProt ID- Q9KPZ1** | | | | | |
| 1. | 15 | 29 | 15 | LSSMIVILGALGLML | 0.38 |
| 2. | 17 | 31 | 15 | SMIVILGALGLMLFY | 0.38 |
| 3. | 16 | 30 | 15 | SSMIVILGALGLMLF | 0.38 |
| 4. | 14 | 28 | 15 | GLSSMIVILGALGLM | 0.56 |
| 5. | 13 | 27 | 15 | VGLSSMIVILGALGL | 0.67 |
| 6. | 17 | 27 | 11 | SMIVILGALGL | 1.26 |
| 7. | 19 | 33 | 15 | IVILGALGLMLFYGG | 1.6 |
| 8. | 18 | 32 | 15 | MIVILGALGLMLFYG | 1.6 |
| 9. | 19 | 29 | 11 | IVILGALGLML | 2.17 |
