# Supplementary material for: Comprehensive in silico analyses of fifty-one uncharacterized proteins from Vibrio cholerae
Source: PLoS One. 2024 Oct 4;19(10):e0311301. doi: 10.1371/journal.pone.0311301 (PMC11452002; doi:10.1371/journal.pone.0311301)
Supplement: S1 Fig — (DOCX) [file pone.0311301.s016.docx]

**Figure S1**

**Hydropathy plot (Kyte/Doolittle plot):** Hydropathy plots illustrating the hydrophilic and hydrophobic region within each of the uncharacterized proteins.


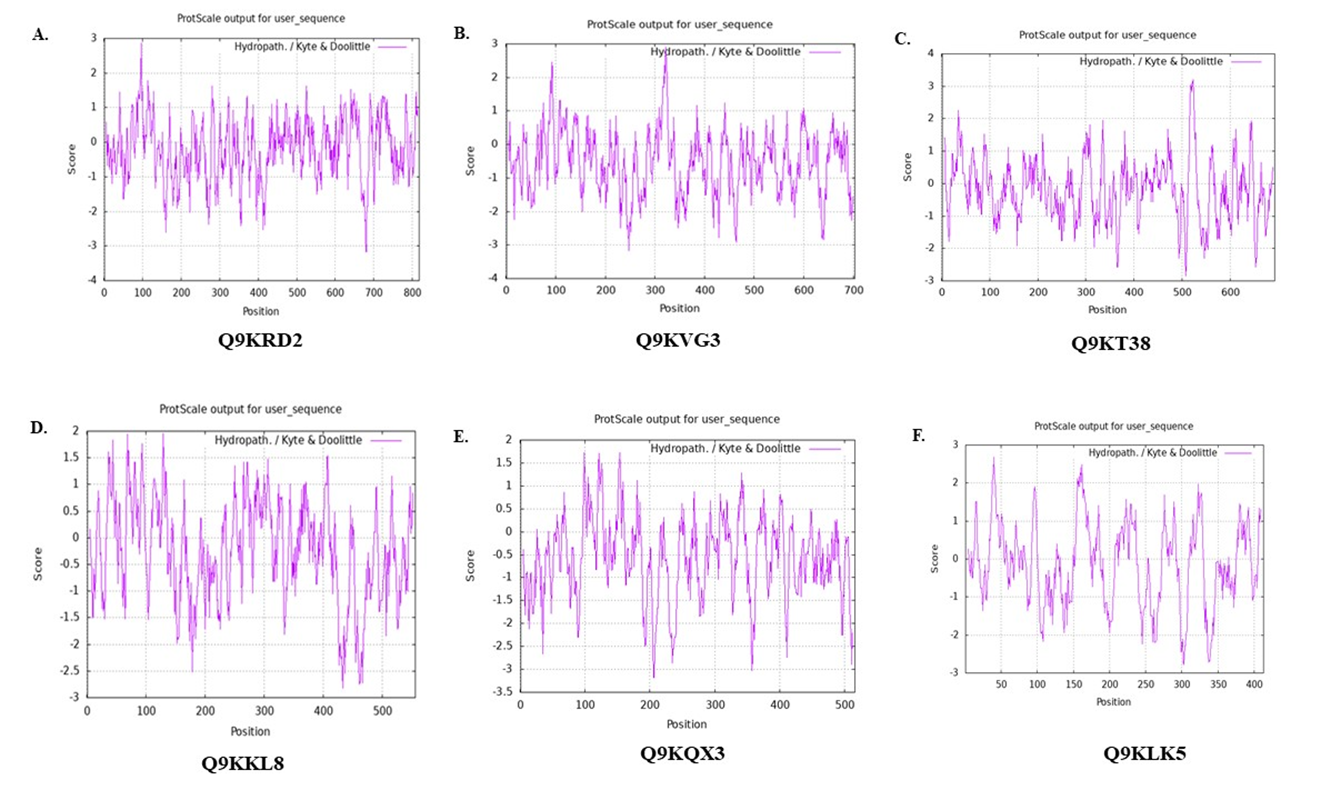


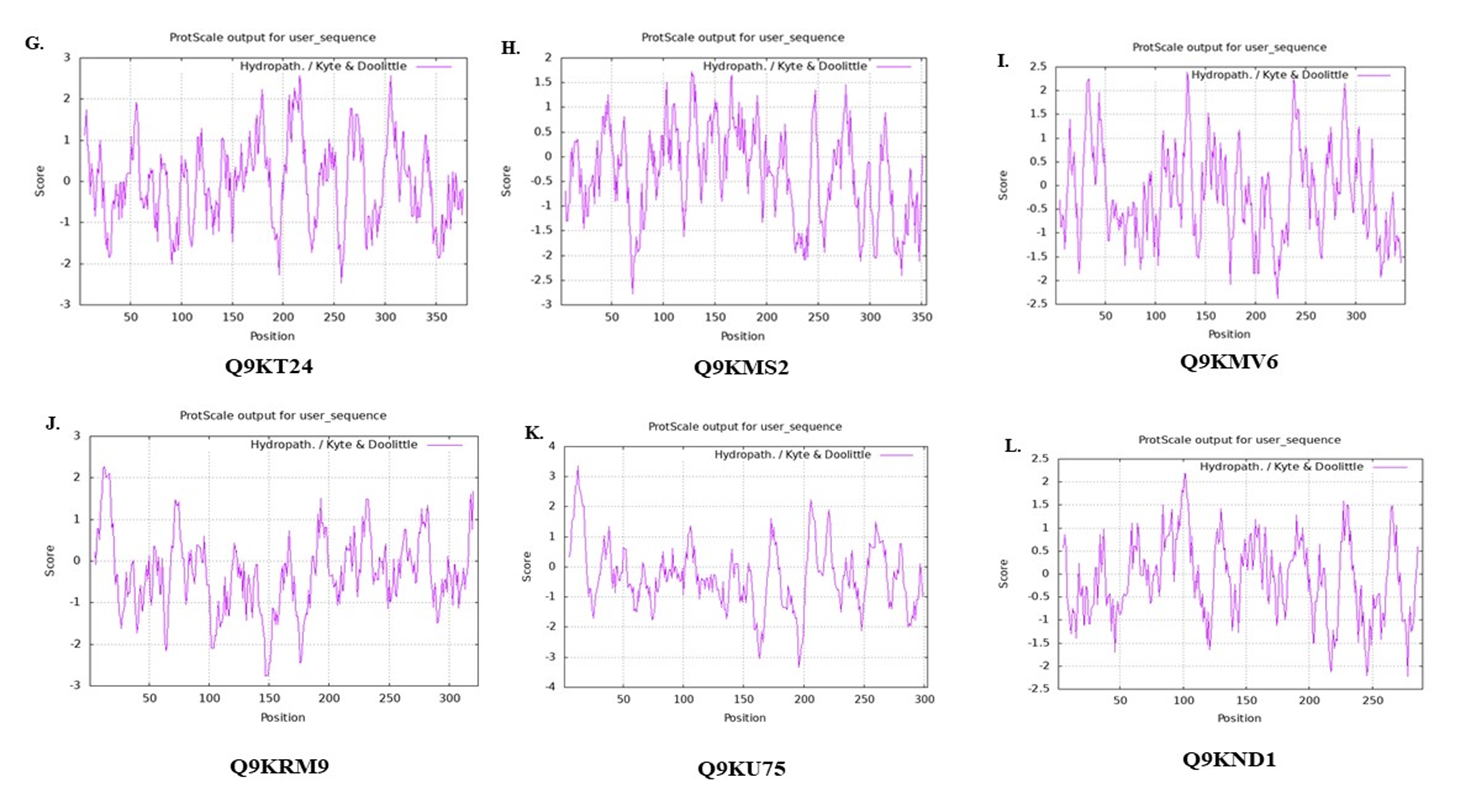


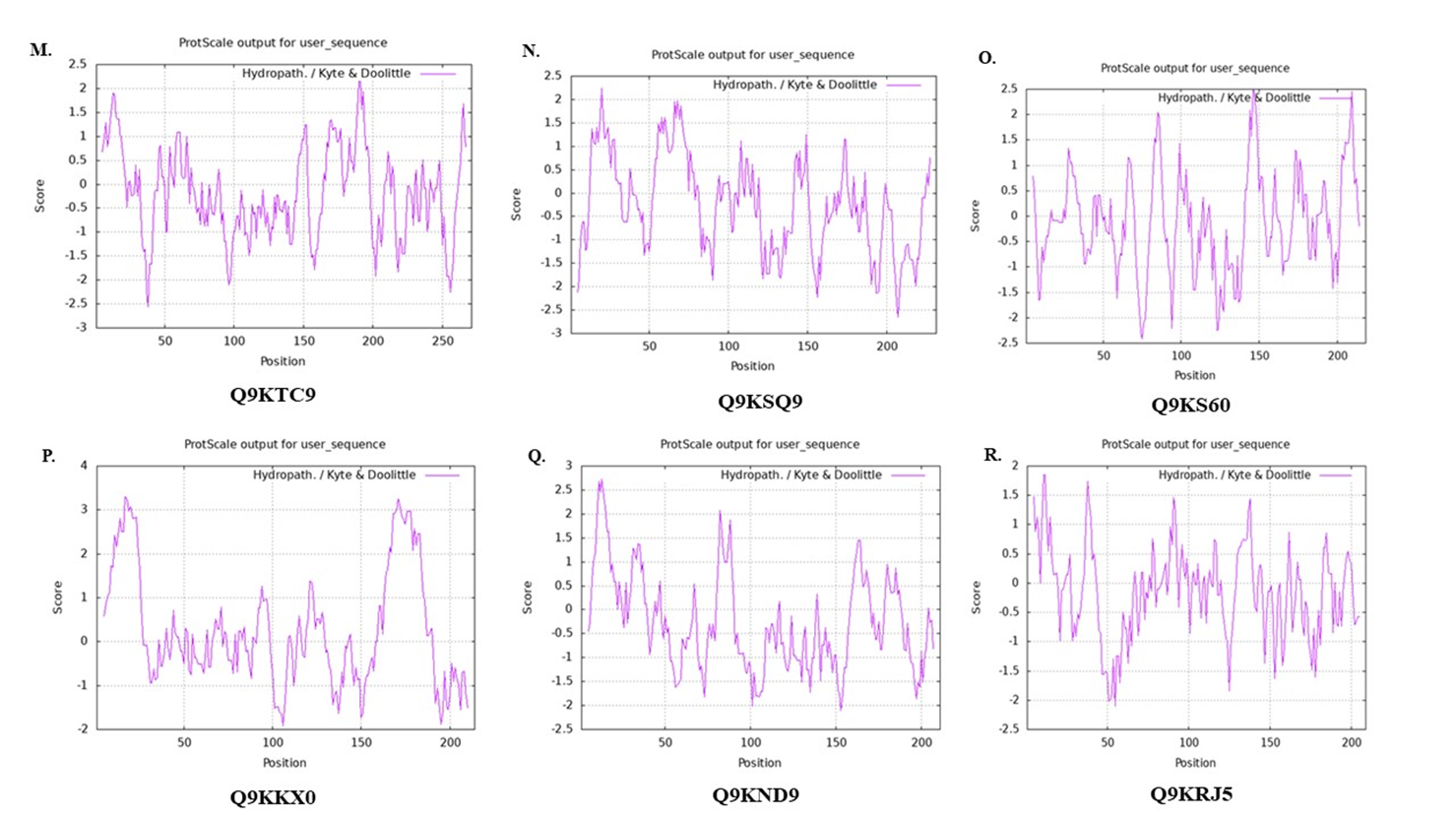


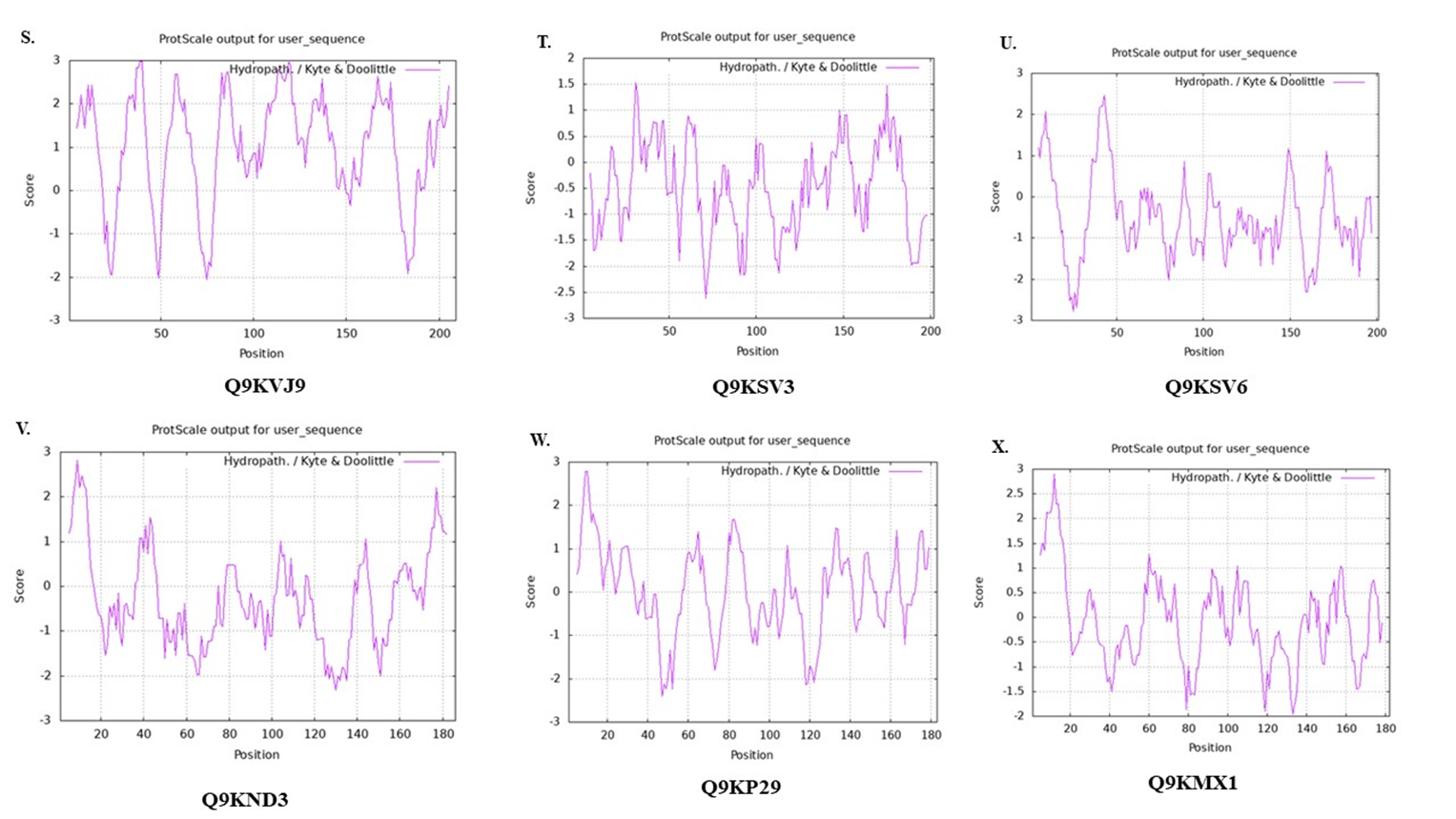


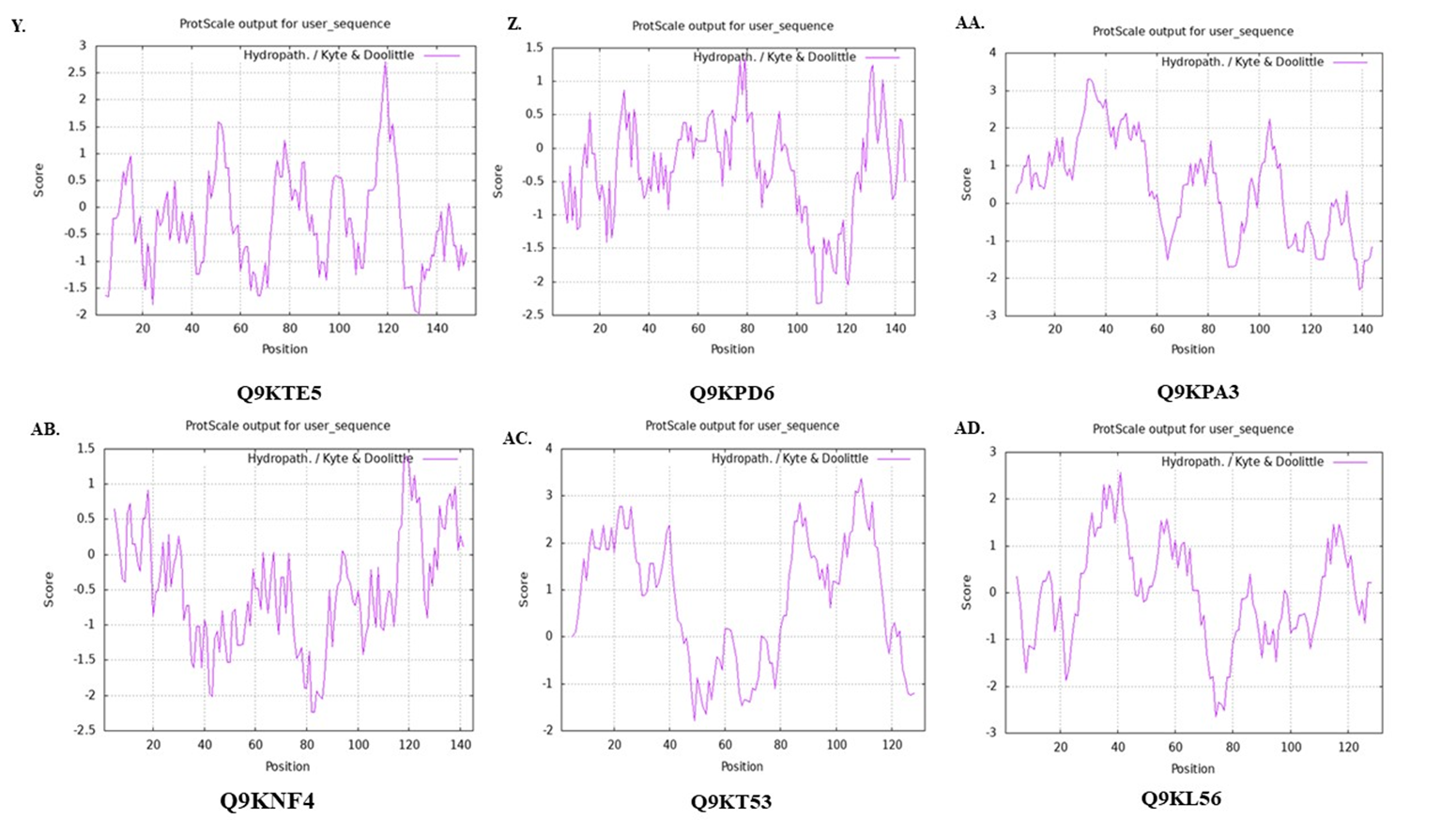


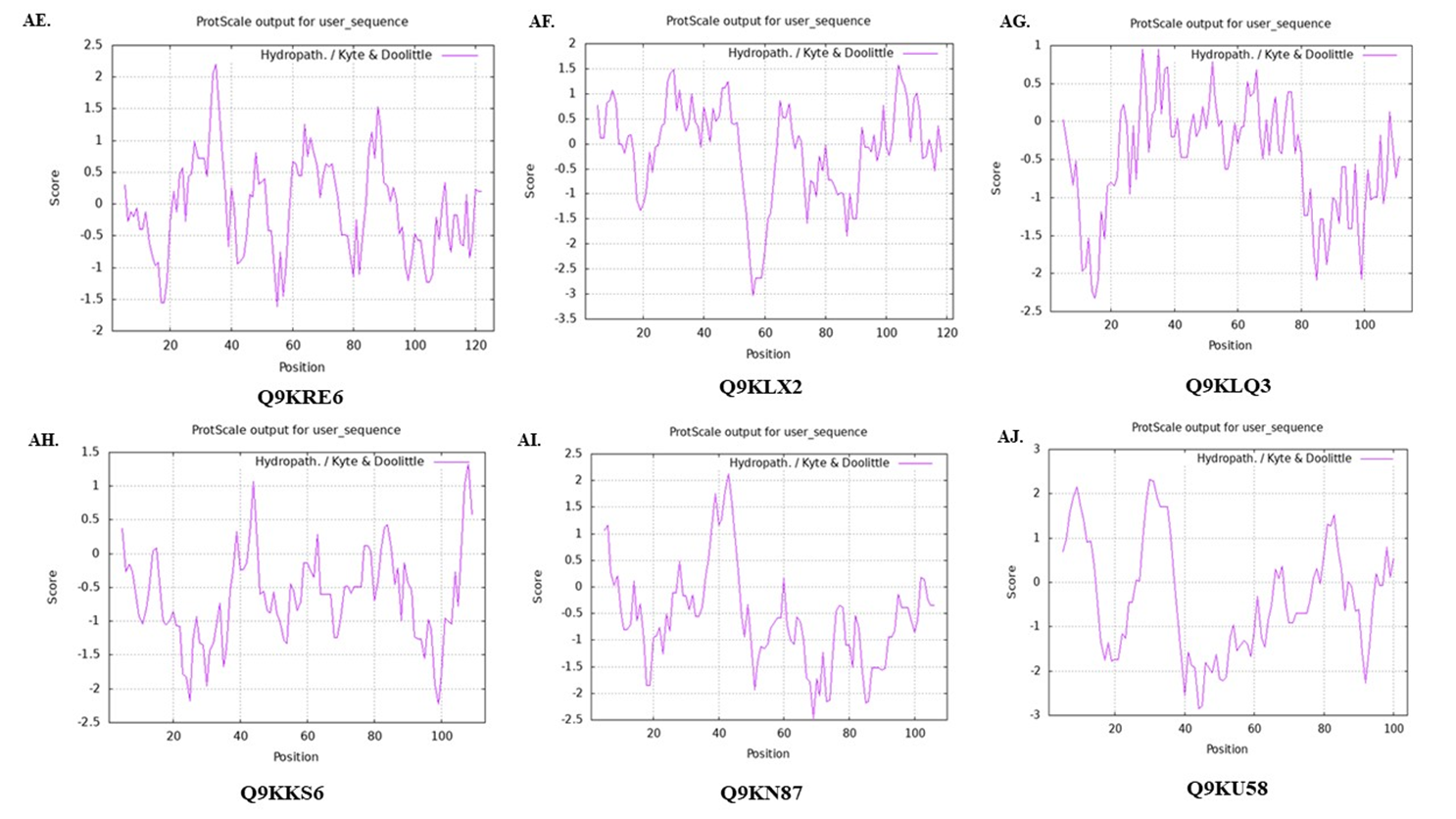


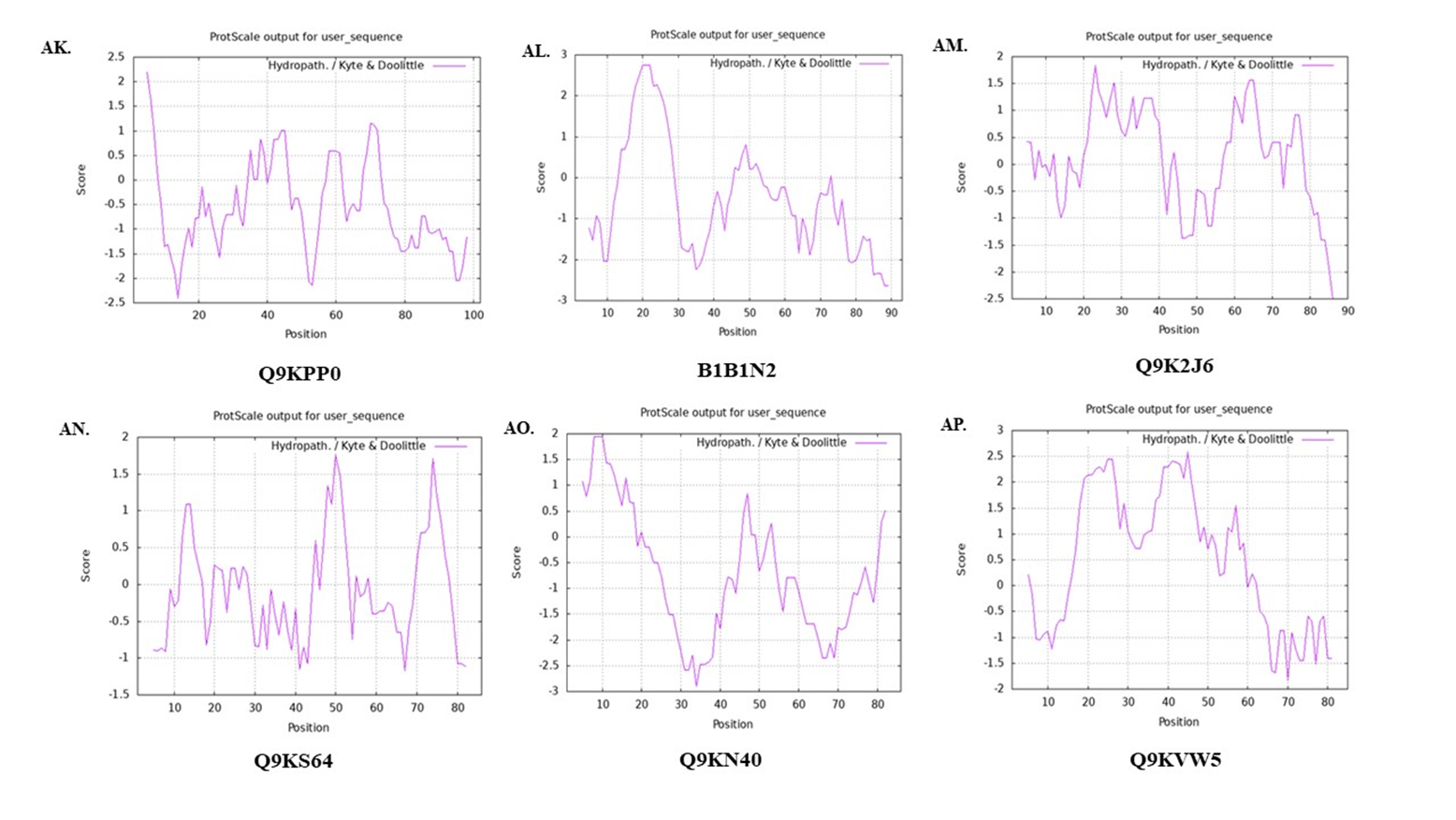


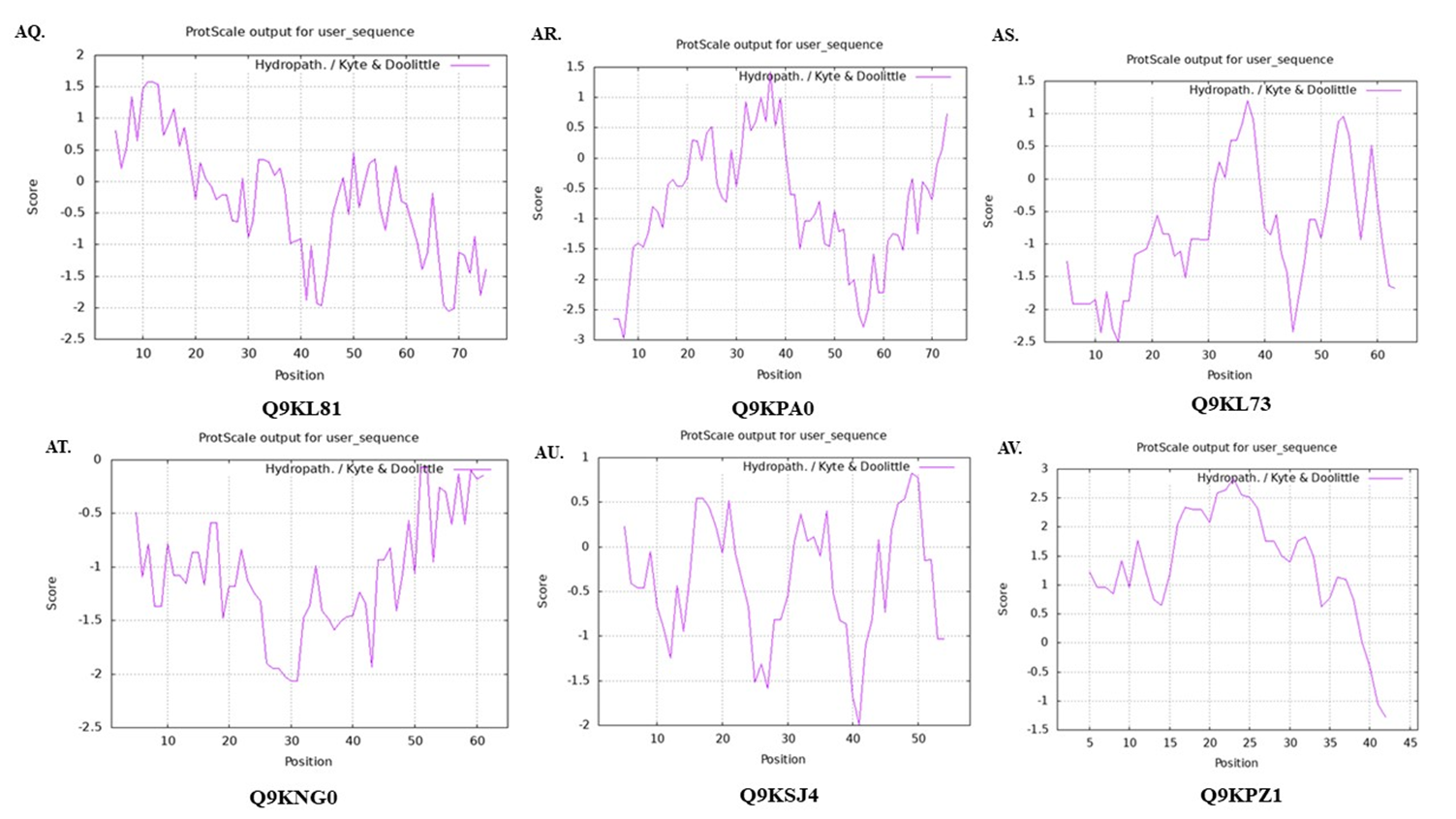


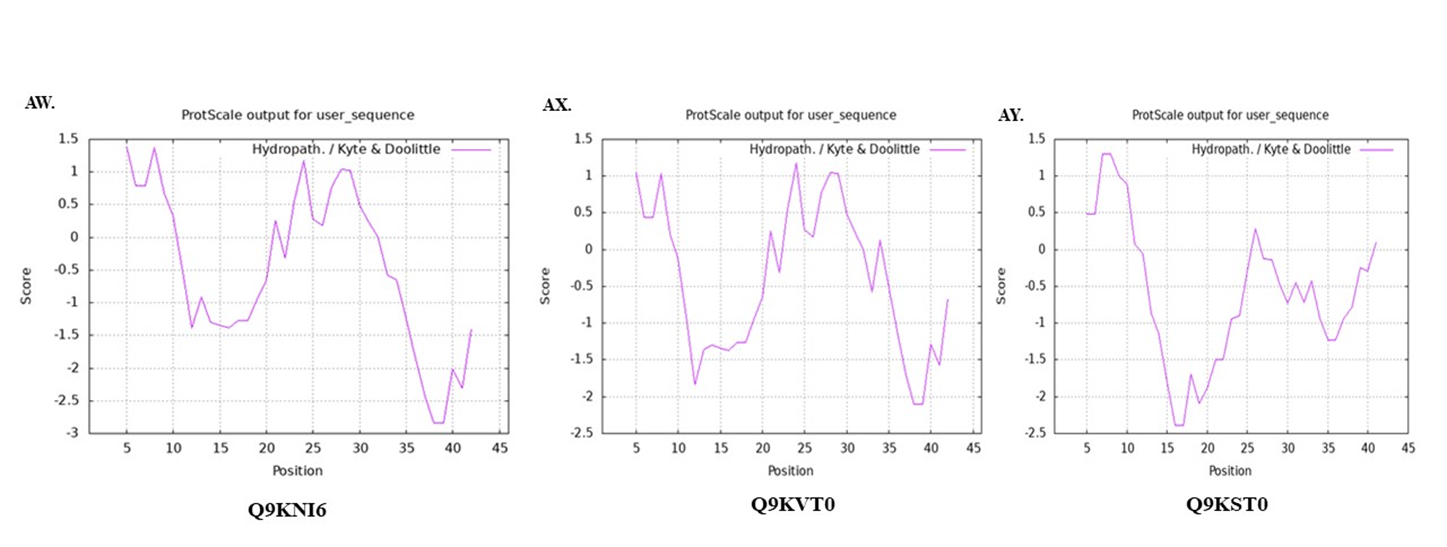


The Kyte-Doolittle hydropathy plot displays the hydrophobic and hydrophilic tendencies of an amino acid sequence forming 3-dimensional structure of a protein. A hydropathy scale is used, which assigns a hydropathy index to each amino acid, based on its relative hydrophobicity (positive value) or hydrophilicity (negative value).
