# Supplementary figures and images for: Comprehensive in silico analyses of fifty-one uncharacterized proteins from Vibrio cholerae
Source: PLoS One. 2024 Oct 4;19(10):e0311301. doi: 10.1371/journal.pone.0311301 (PMC11452002; doi:10.1371/journal.pone.0311301)

**Figure S2**

**Computational prediction of protein-protein interaction (PPI) network by STRING**

**
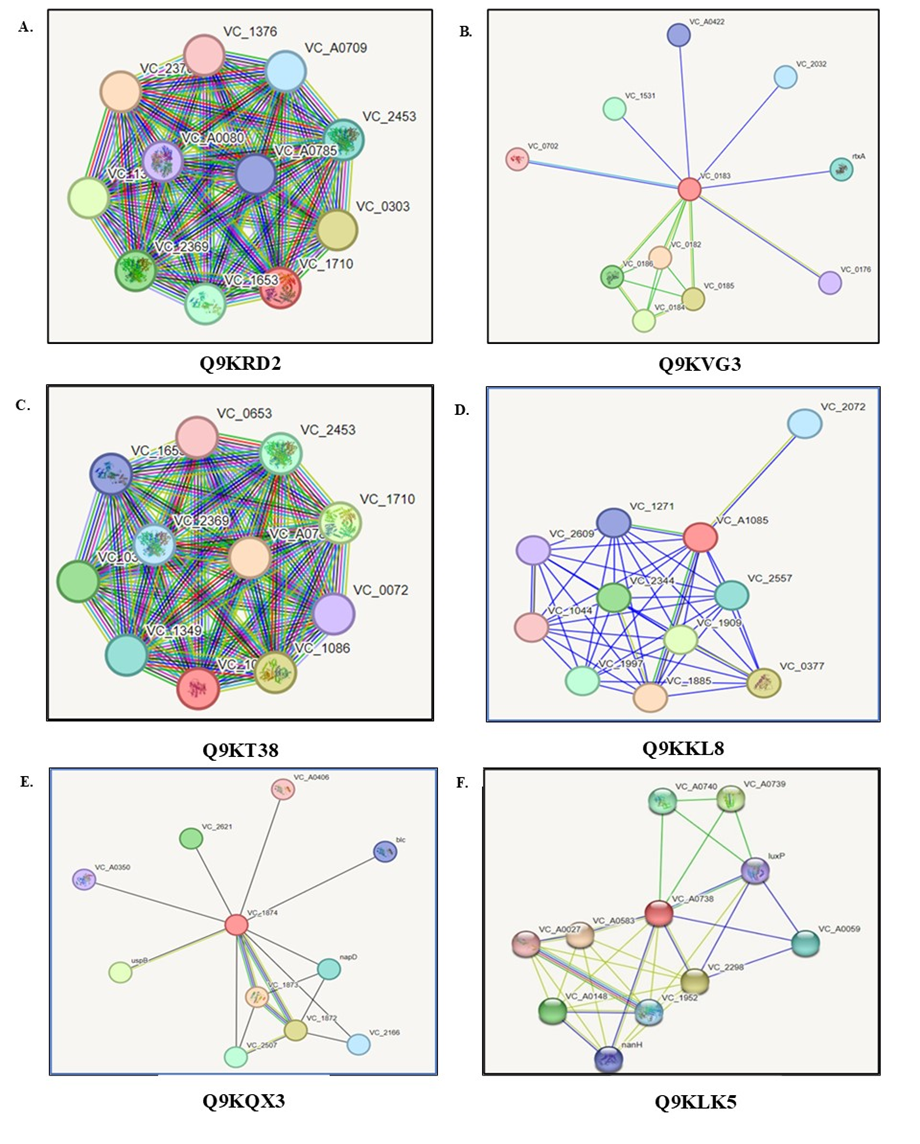
**


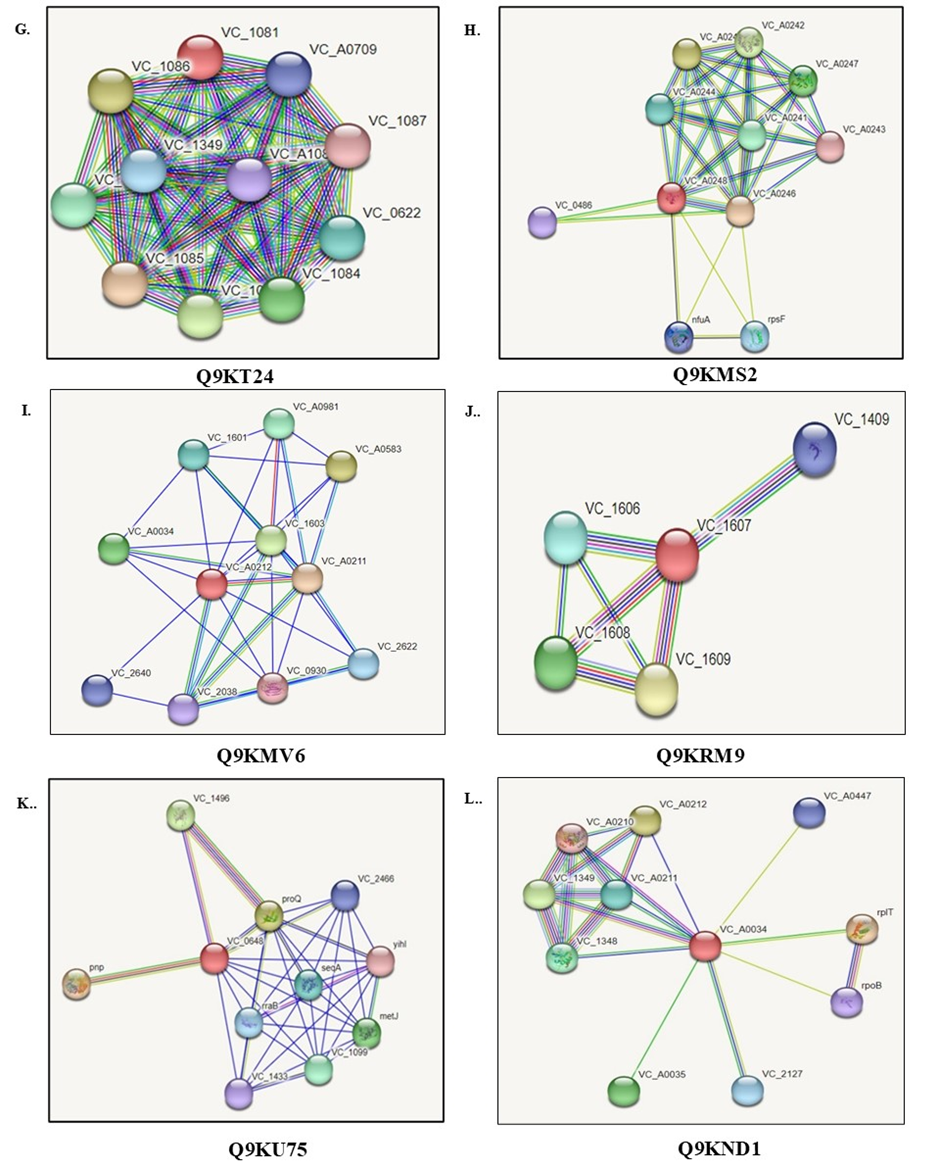


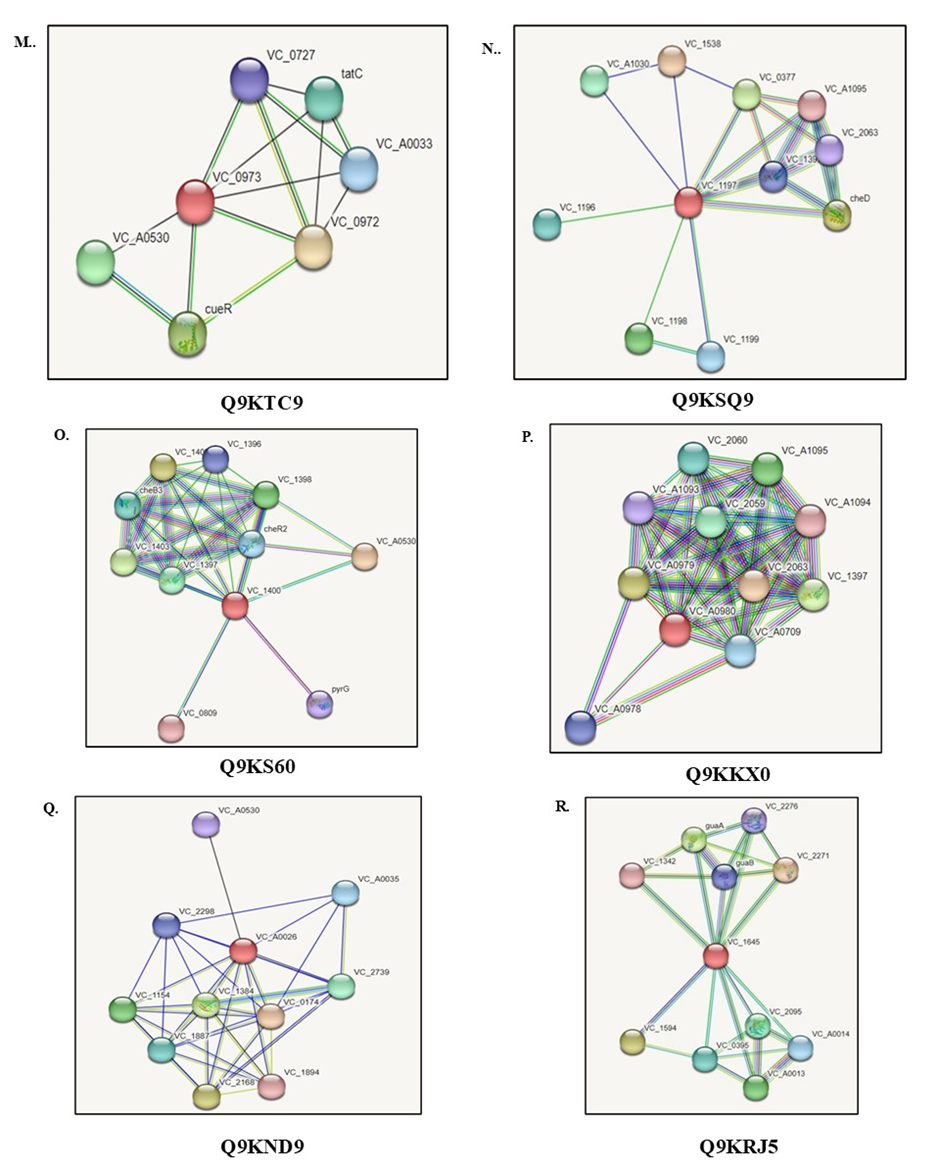


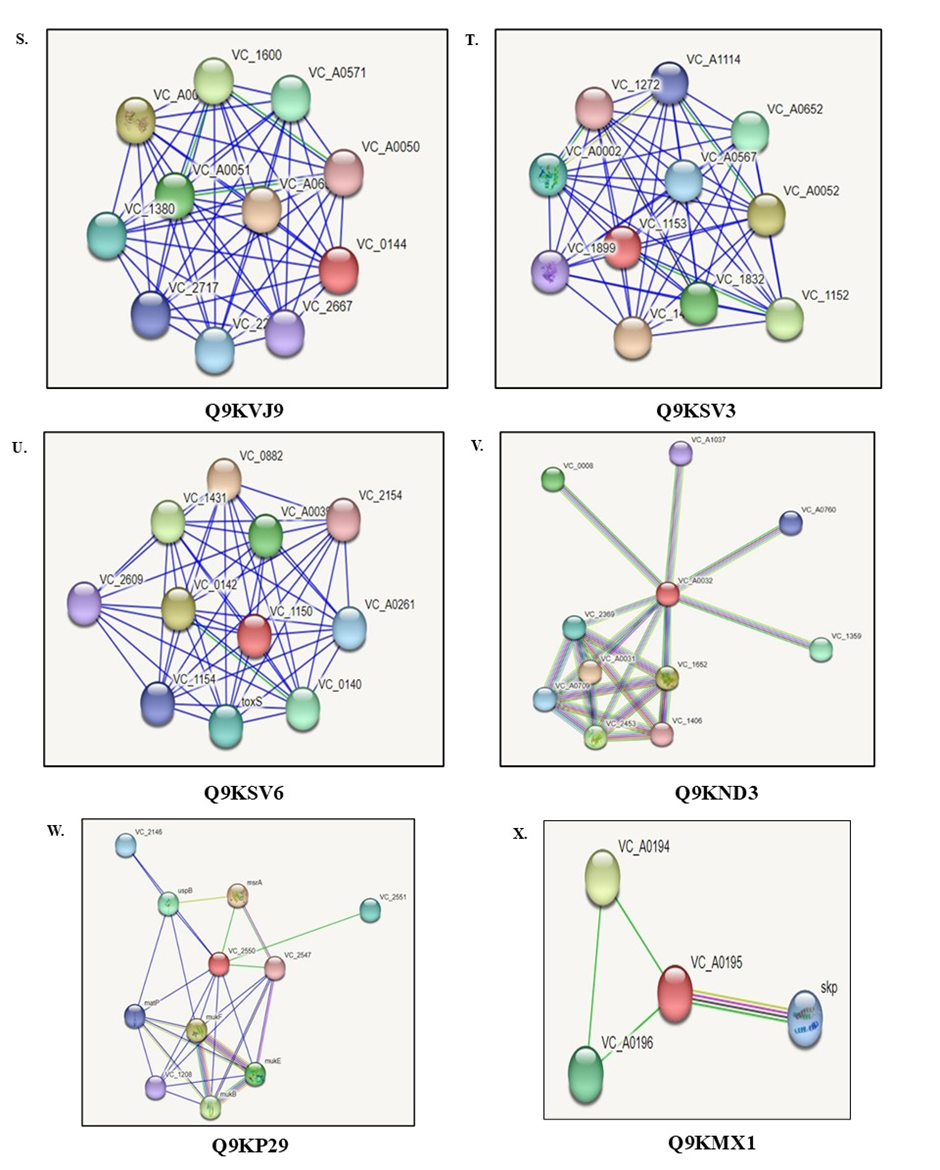


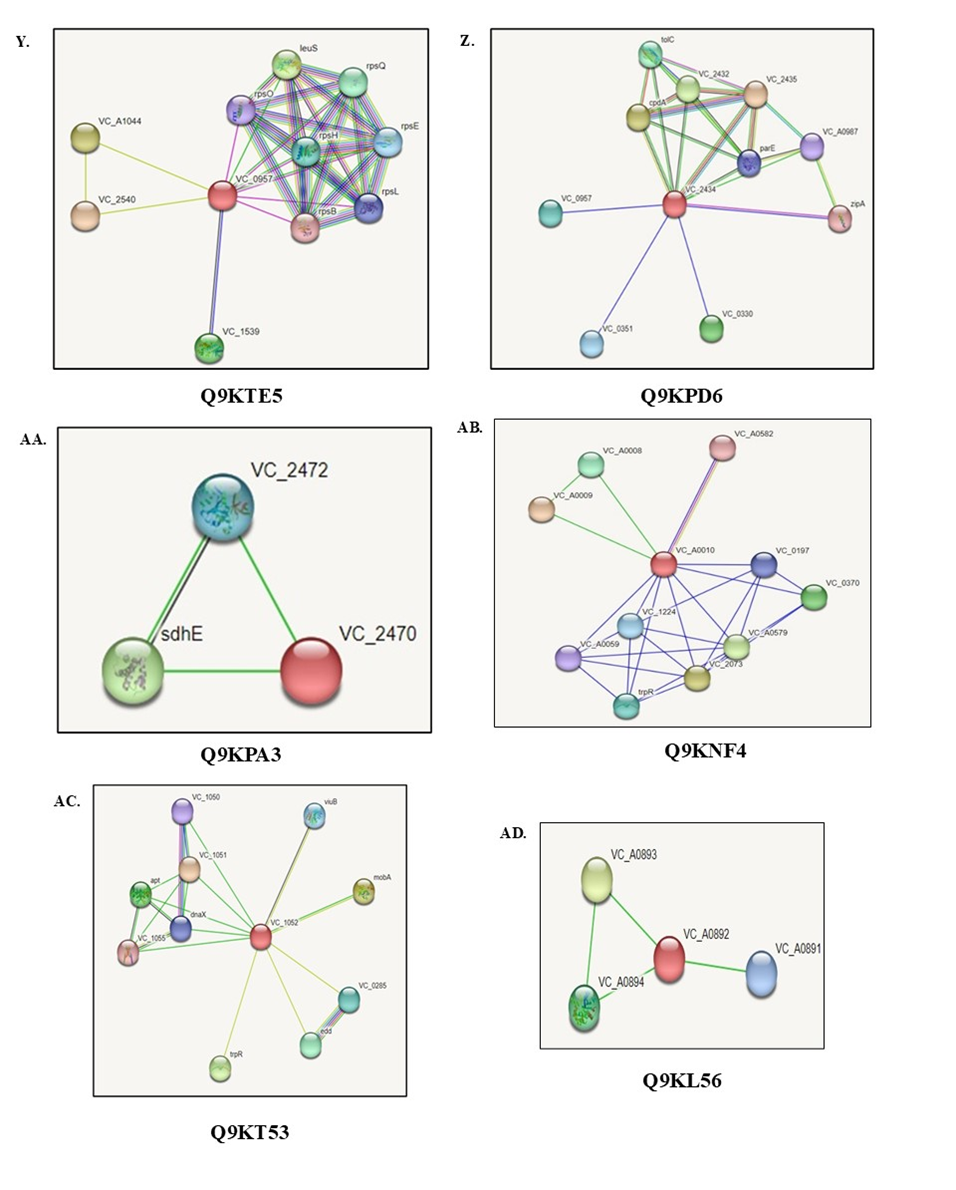


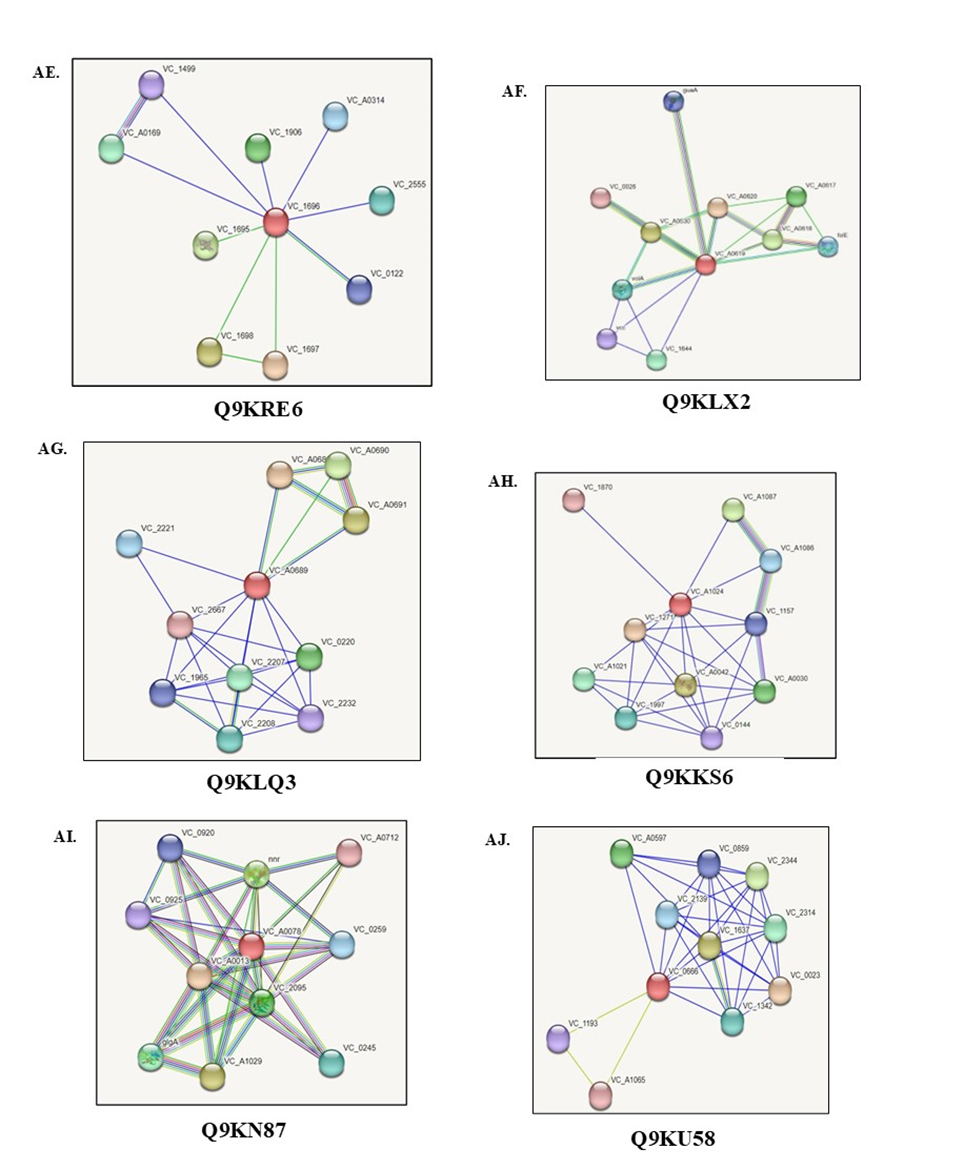


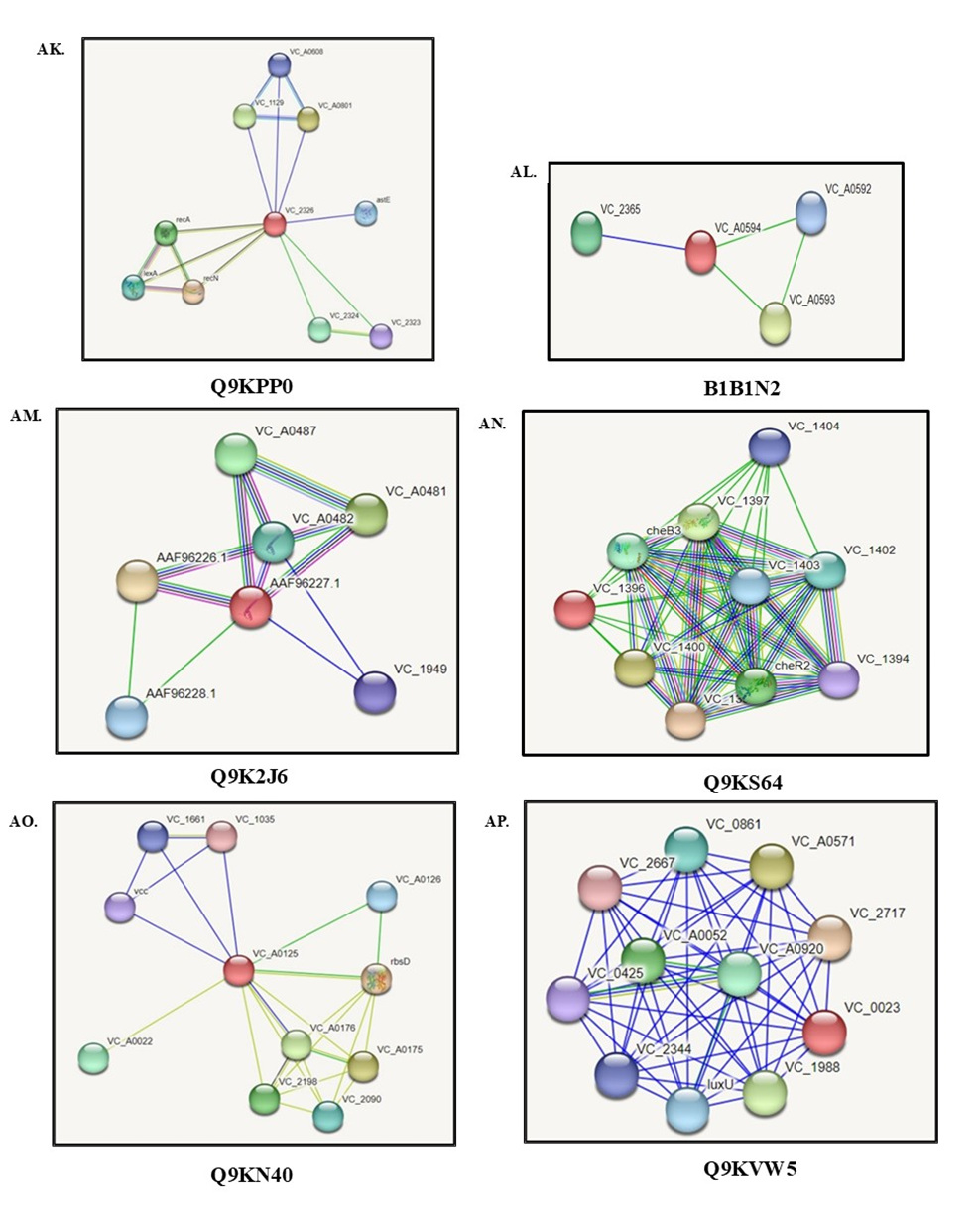

Supplement: S2 Fig — (DOCX) [file pone.0311301.s017.docx]
