# Supplementary material for: Comprehensive in silico analyses of fifty-one uncharacterized proteins from Vibrio cholerae
Source: PLoS One. 2024 Oct 4;19(10):e0311301. doi: 10.1371/journal.pone.0311301 (PMC11452002; doi:10.1371/journal.pone.0311301)

**Figure S3**

**Identification of secondary structures present within each of the uncharacterized protein by PSIPRED.**


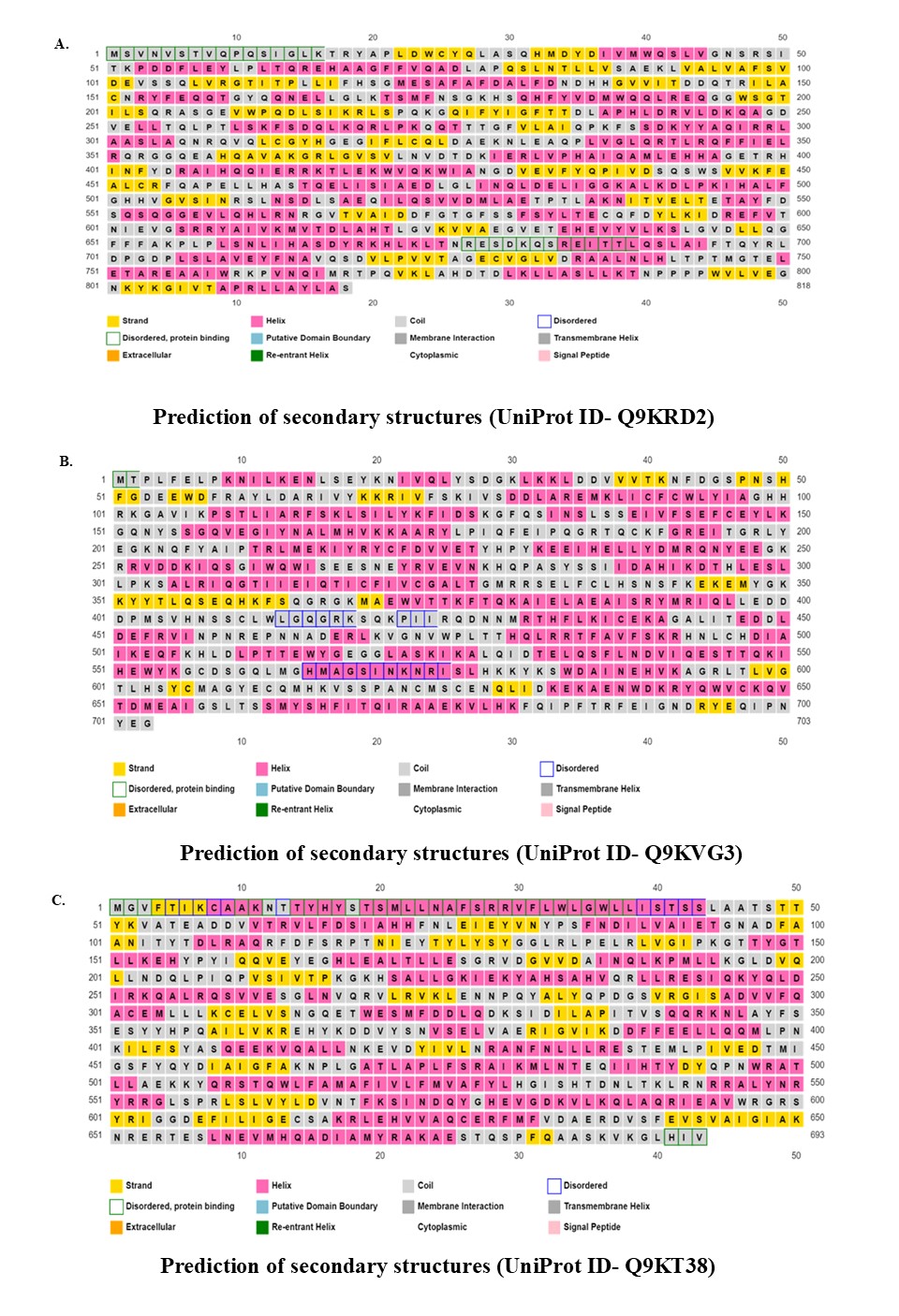


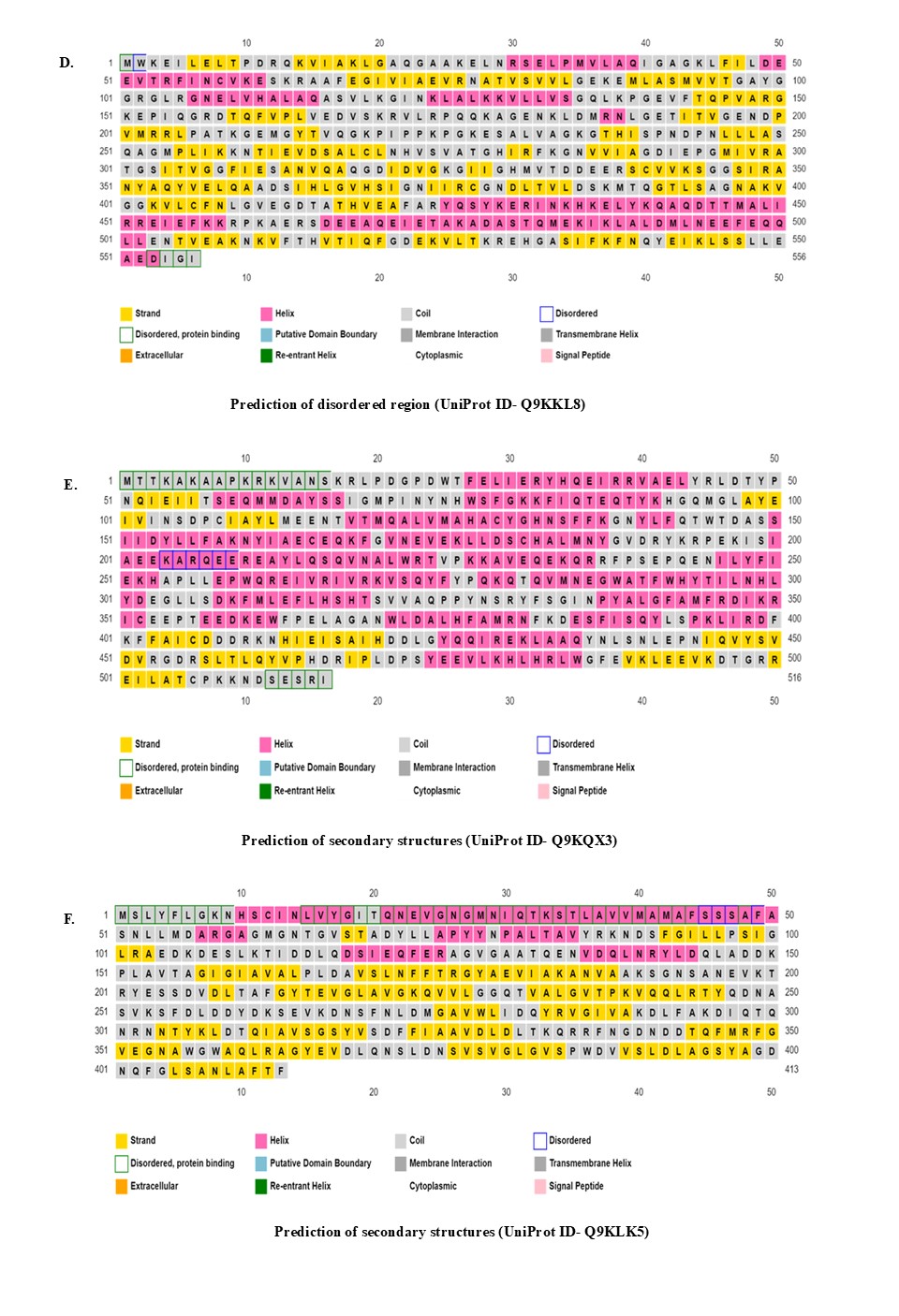


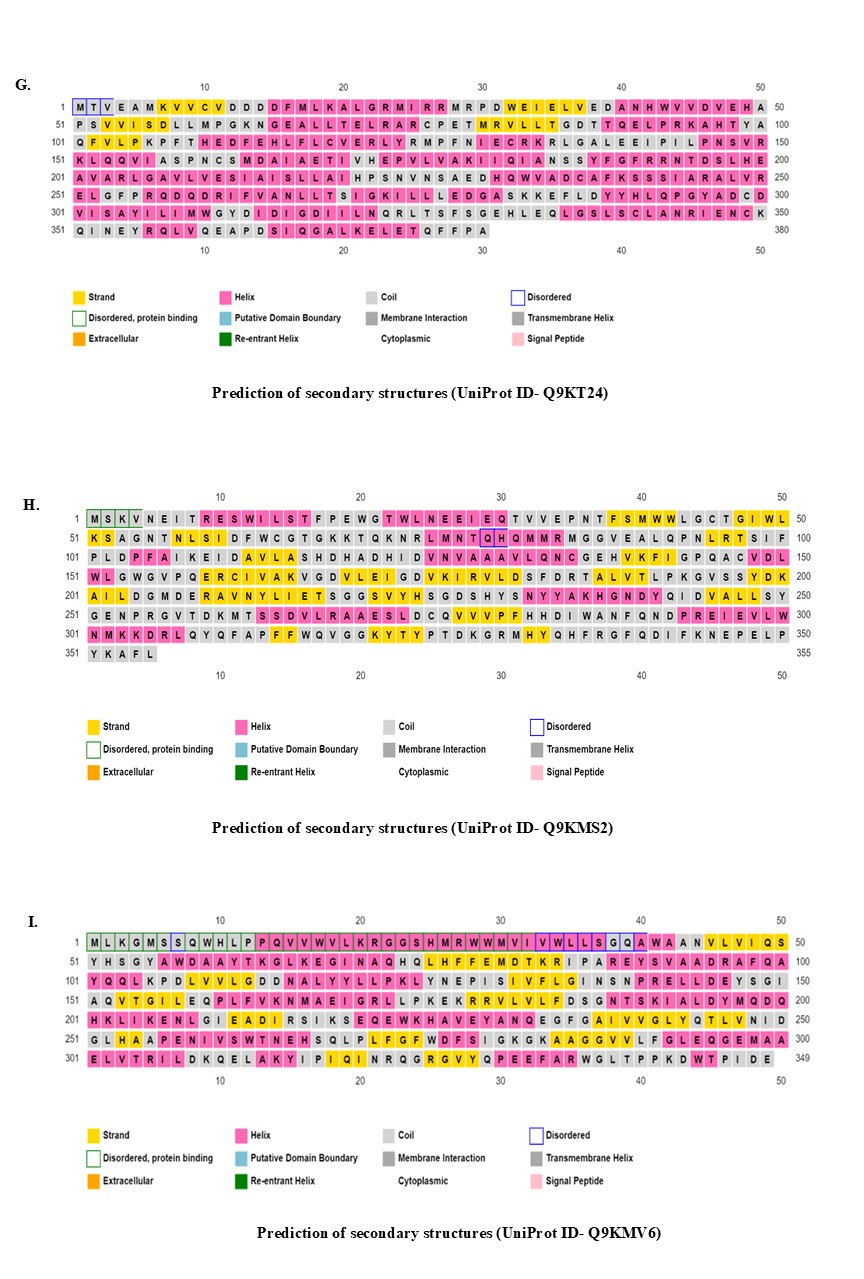


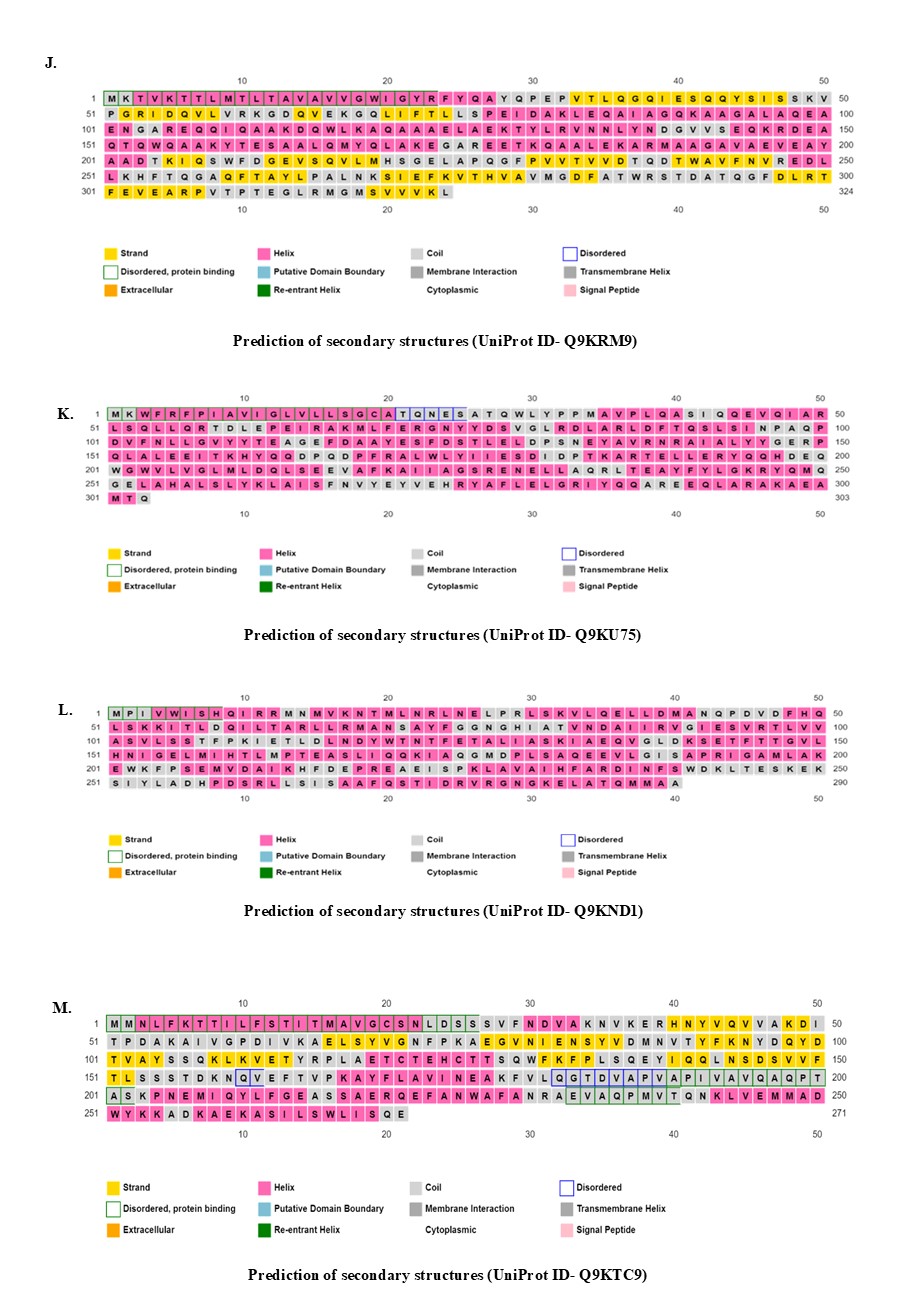


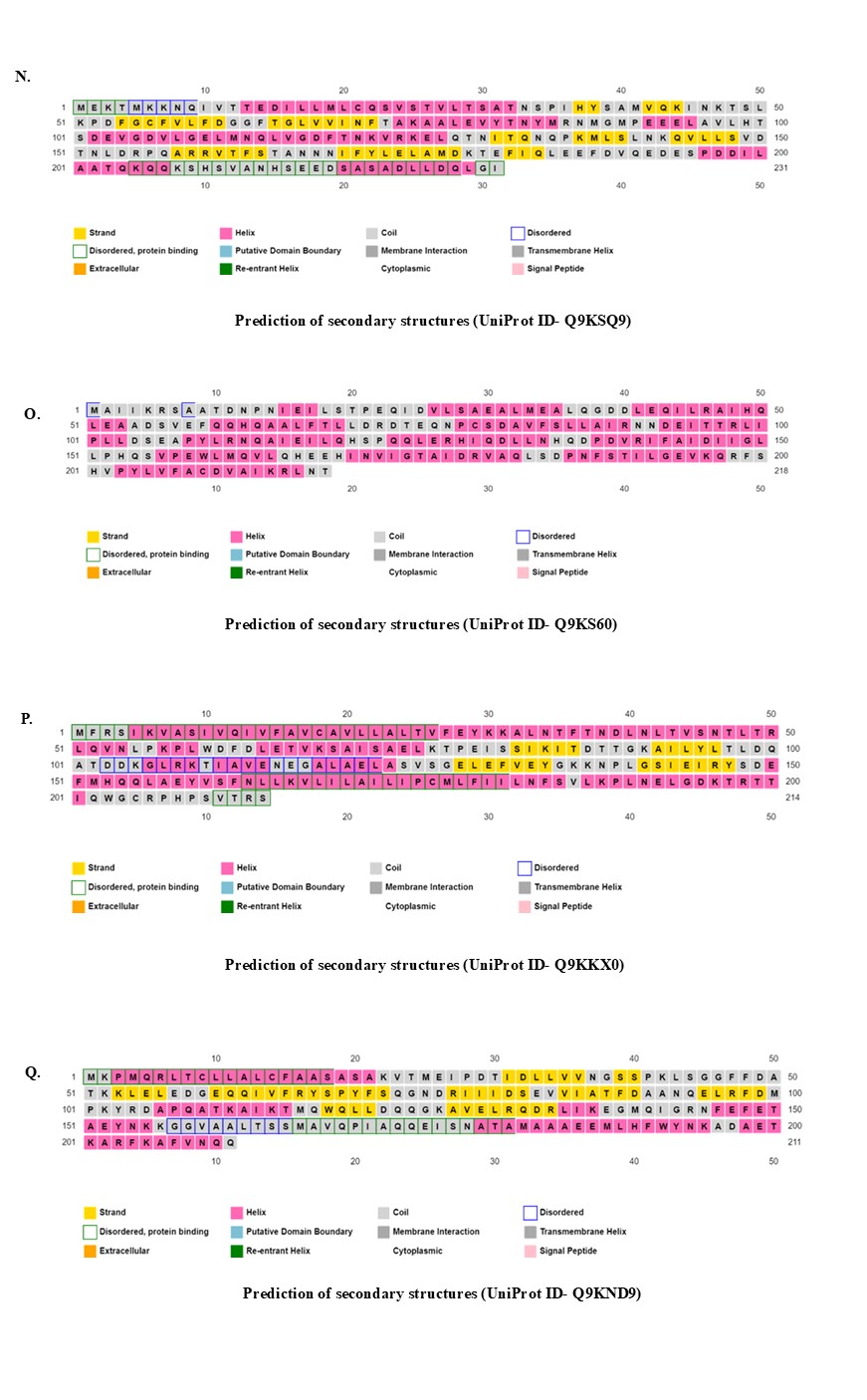


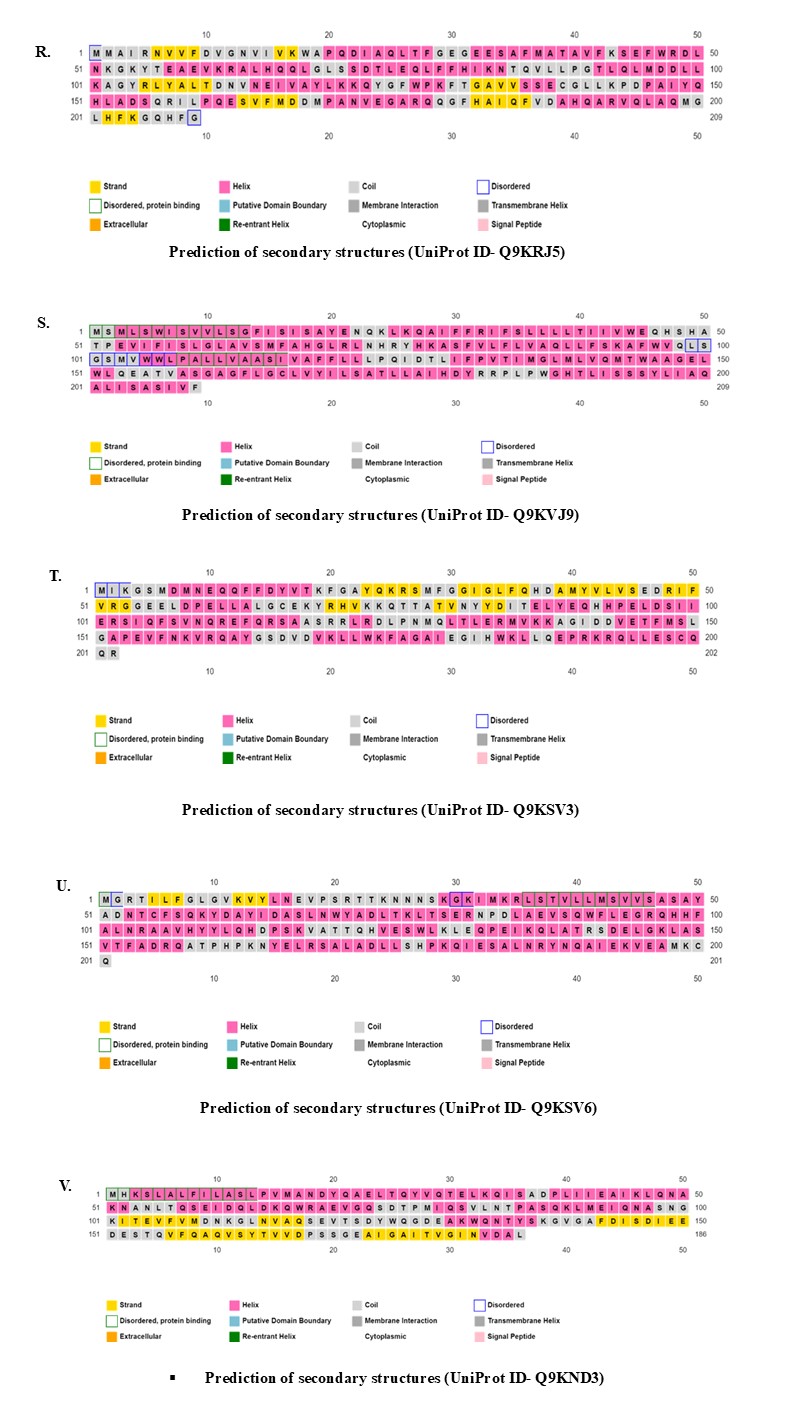


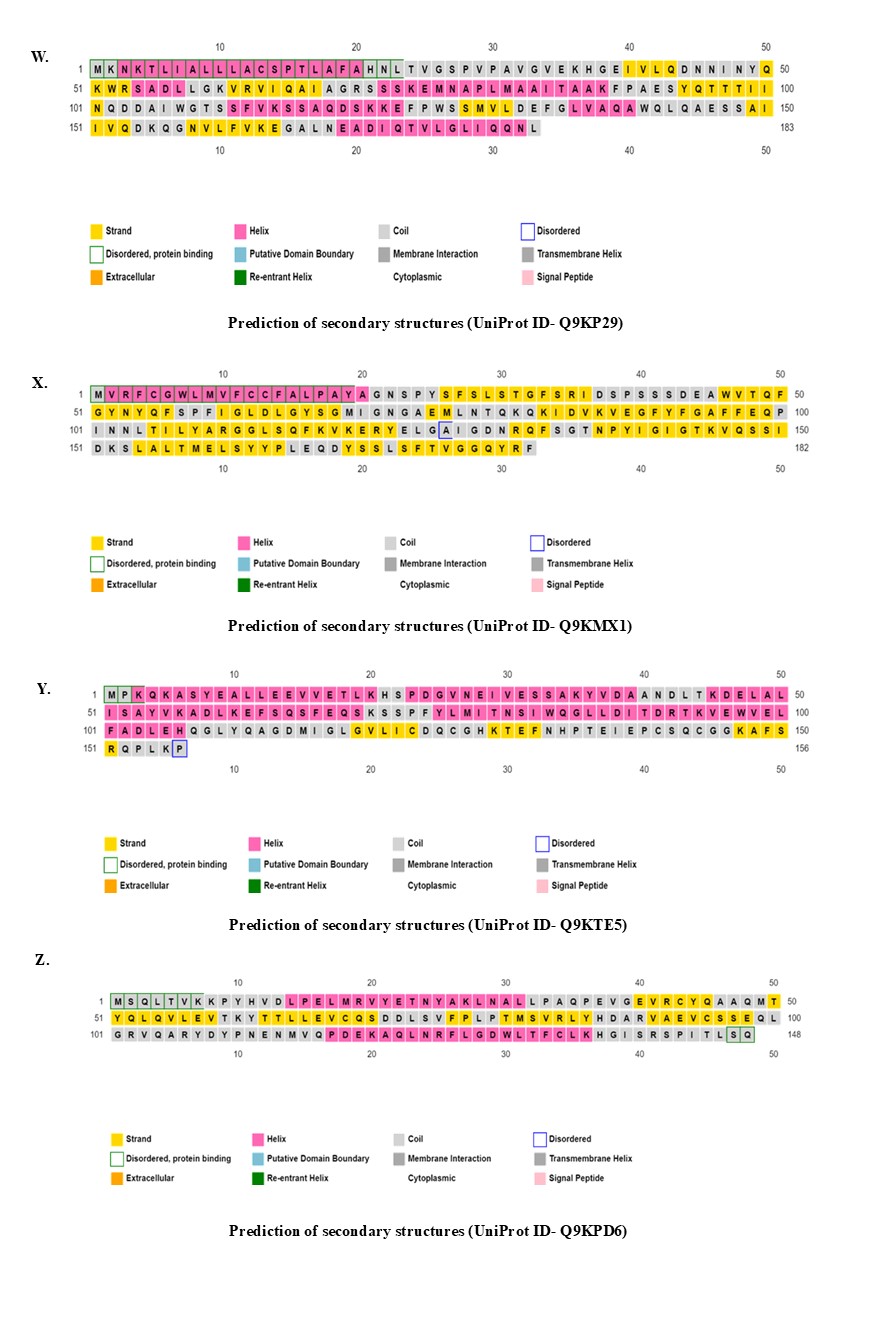


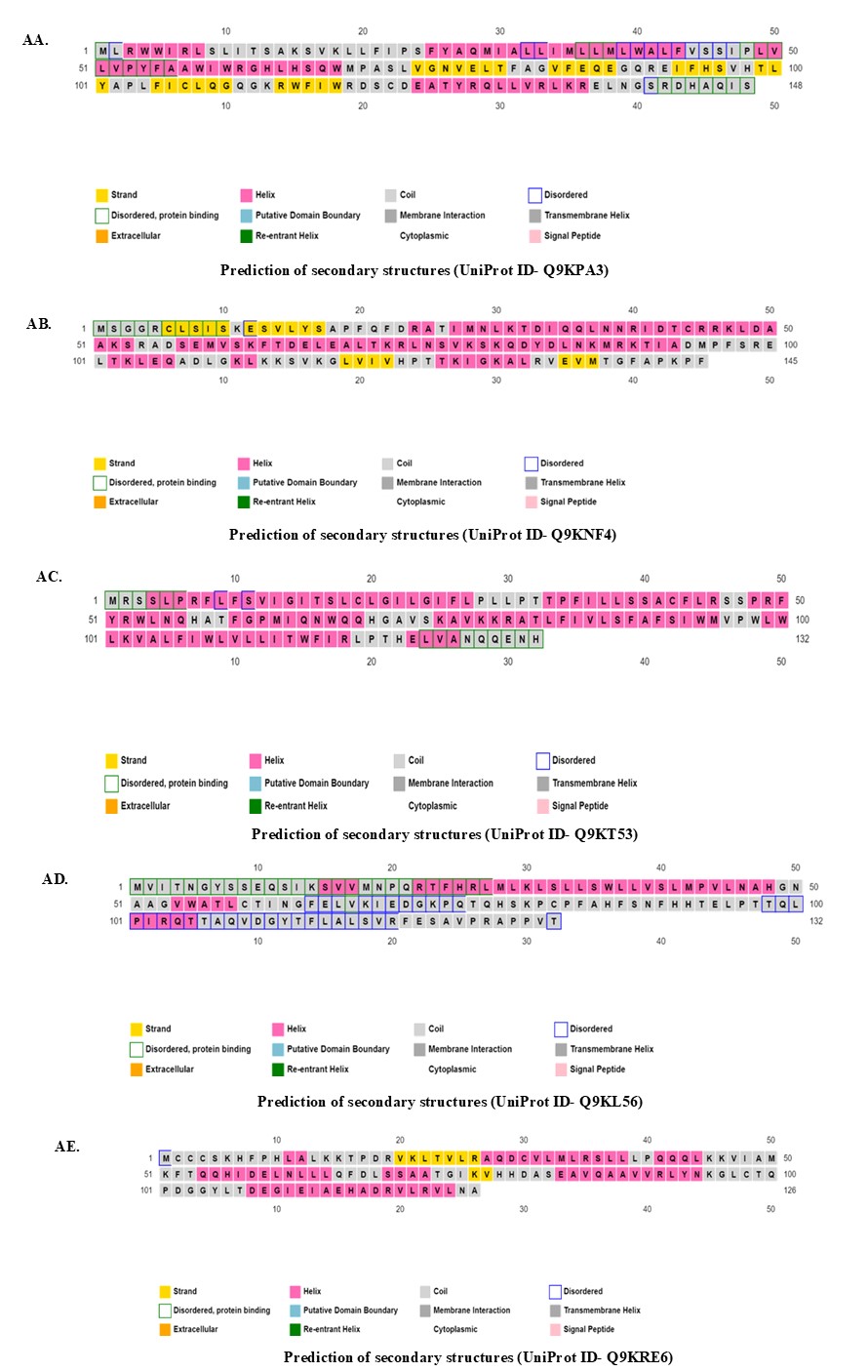


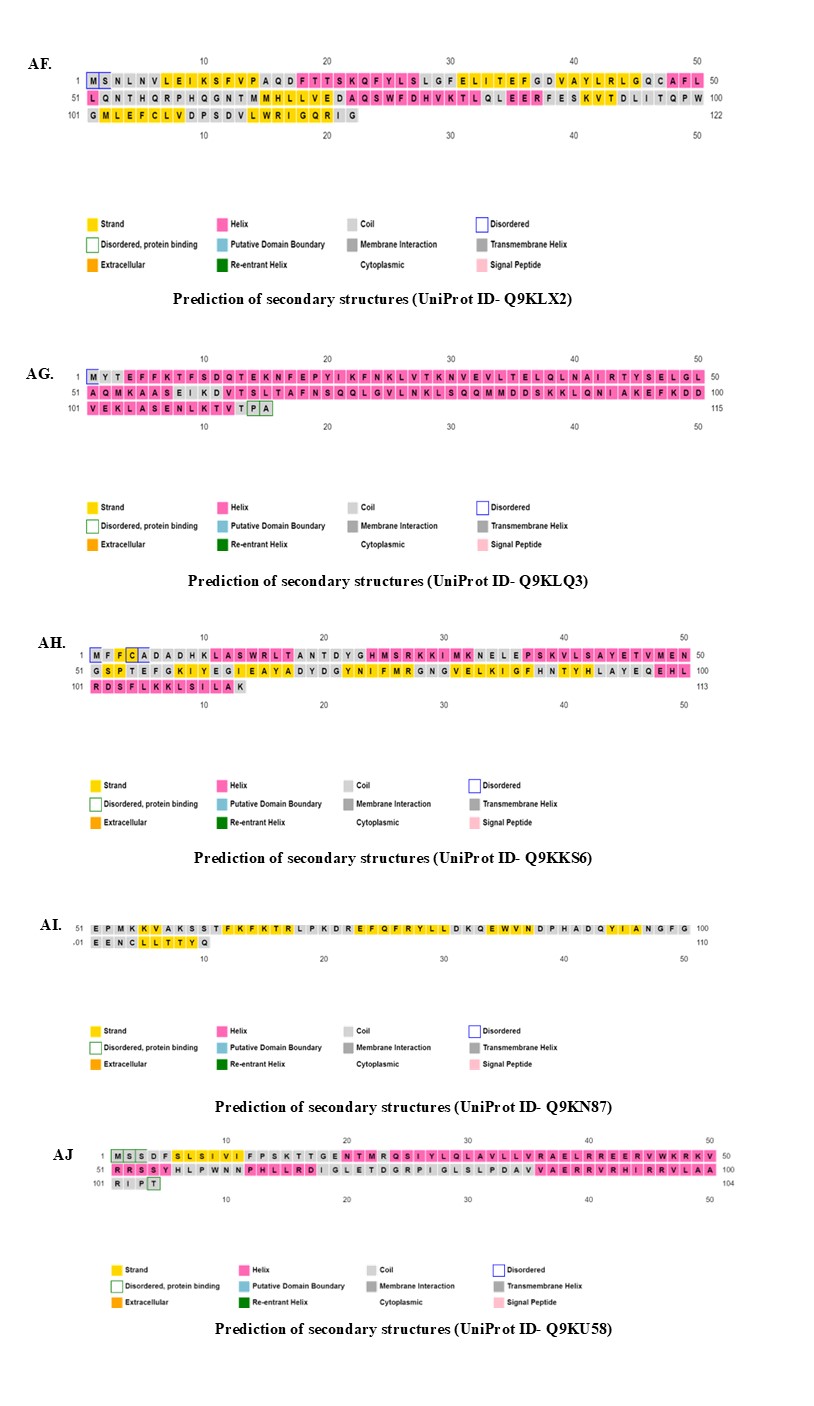


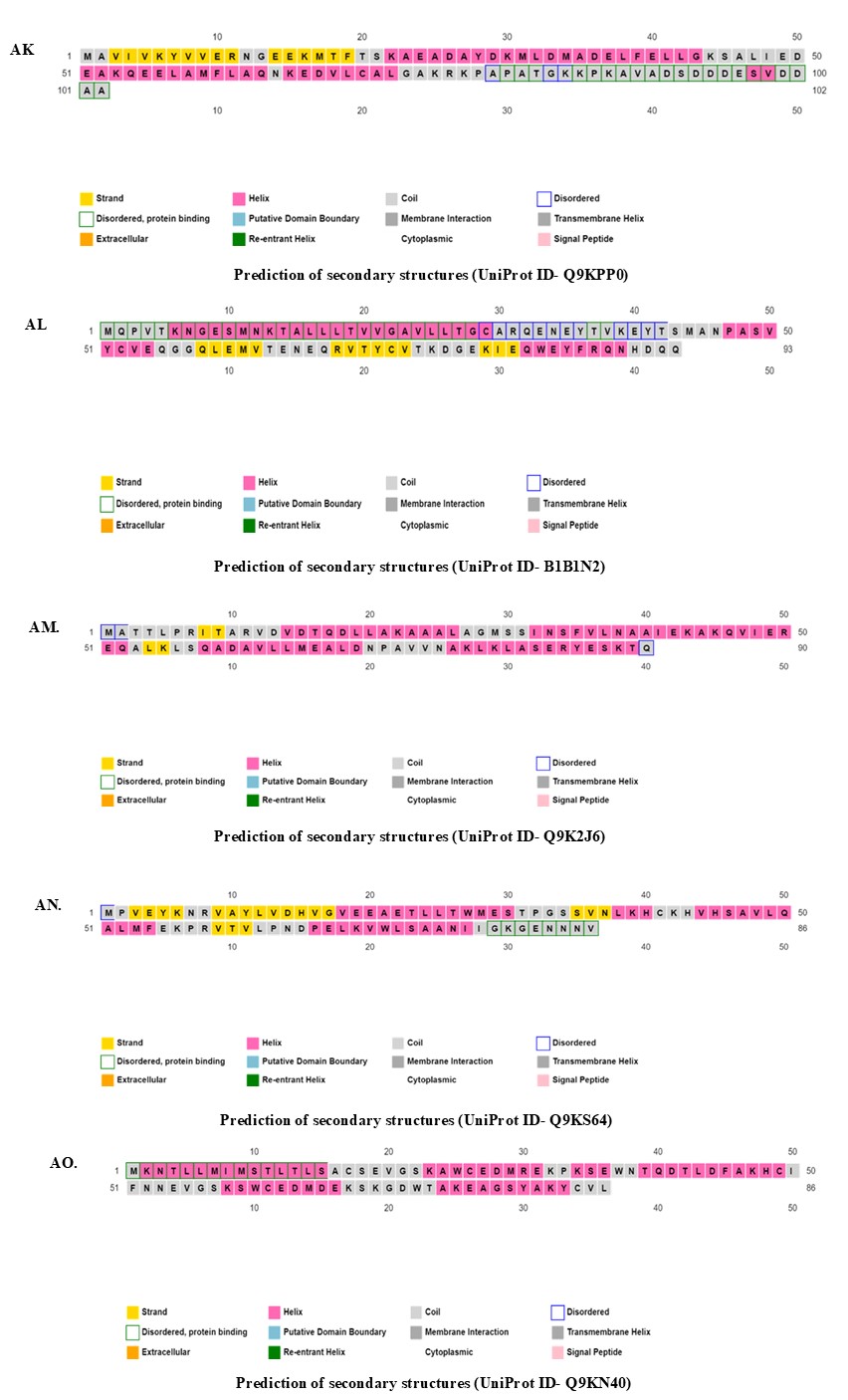


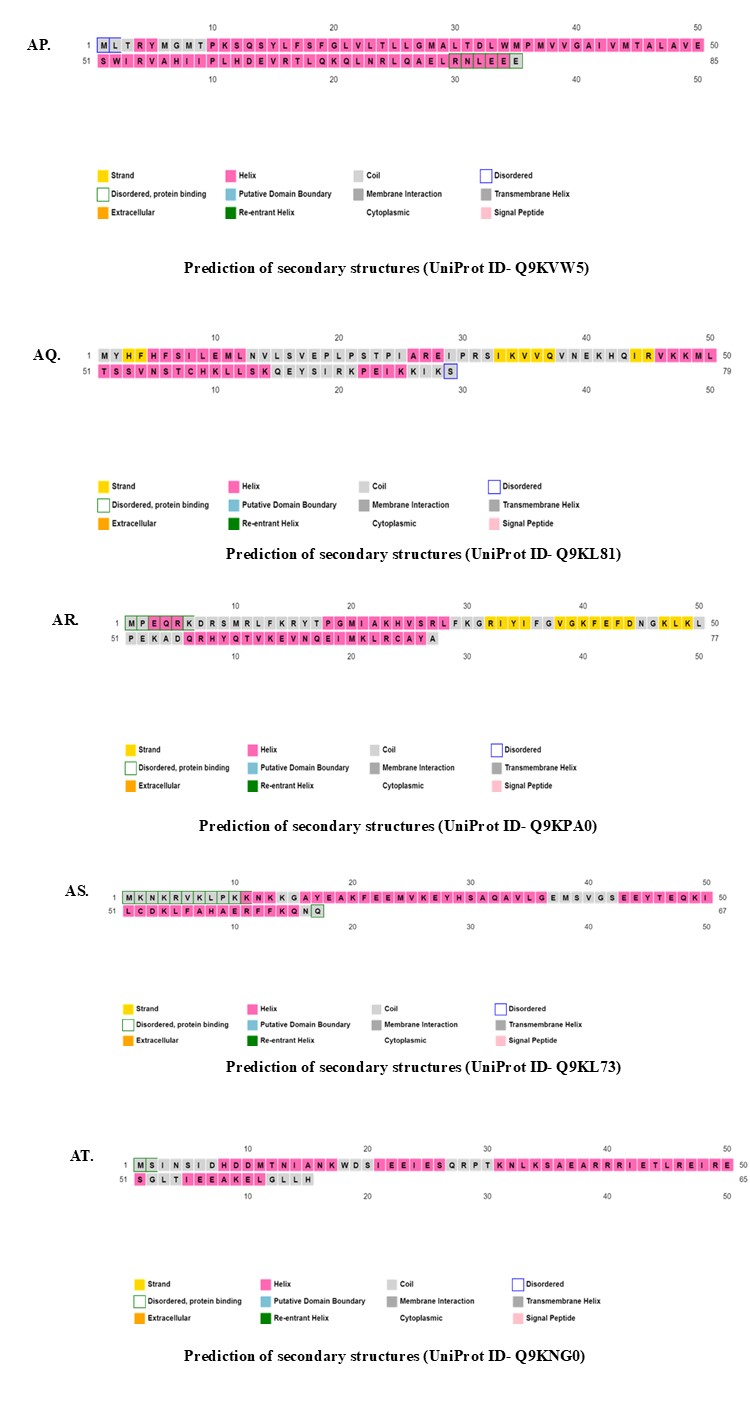


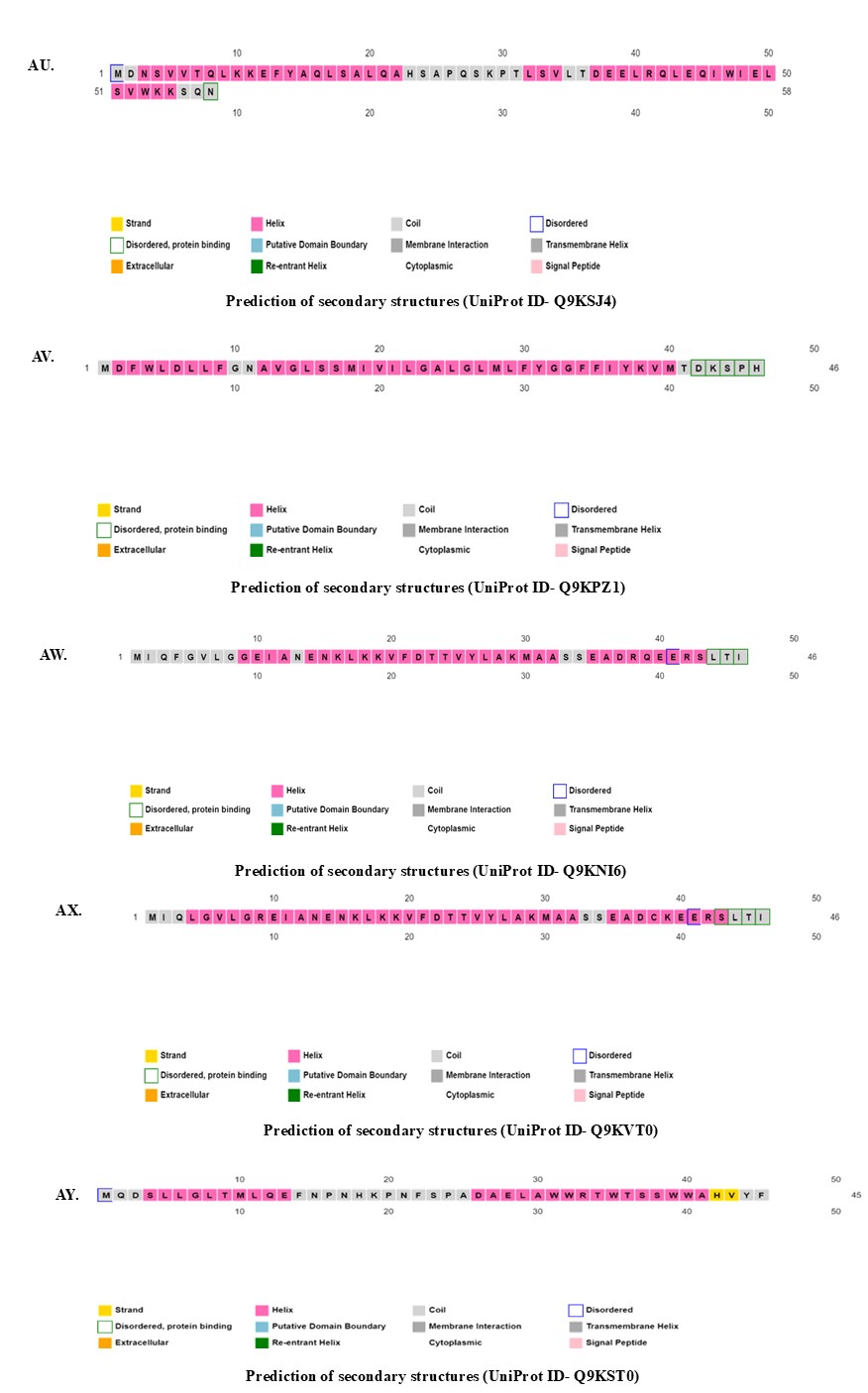

Supplement: S3 Fig — (DOCX) [file pone.0311301.s018.docx]
