# Supplementary material for: Comprehensive in silico analyses of fifty-one uncharacterized proteins from Vibrio cholerae
Source: PLoS One. 2024 Oct 4;19(10):e0311301. doi: 10.1371/journal.pone.0311301 (PMC11452002; doi:10.1371/journal.pone.0311301)

**Figure S4**

**Prediction of disordered regions present within candidate uncharacterized proteins by DISOPRED.**


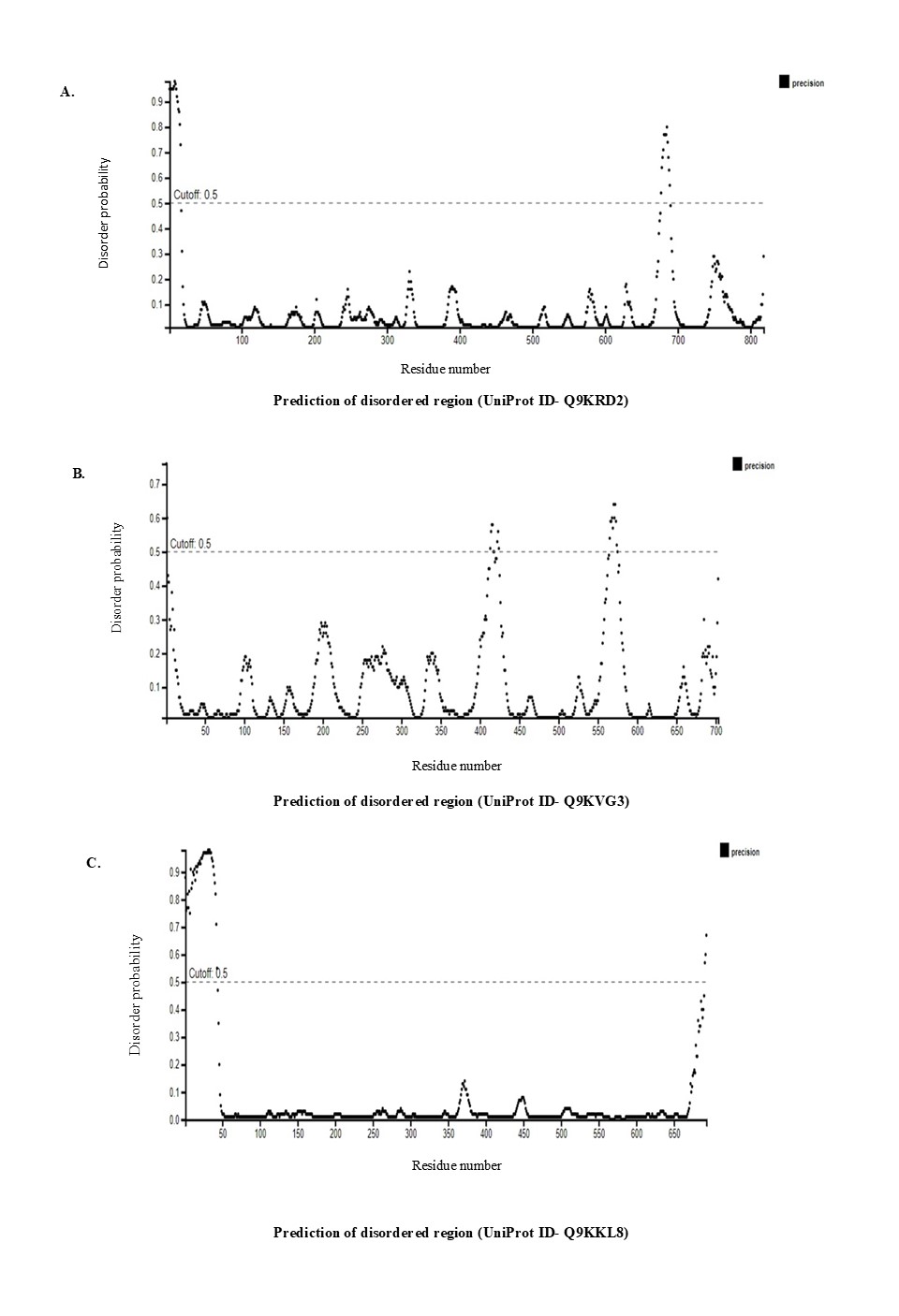


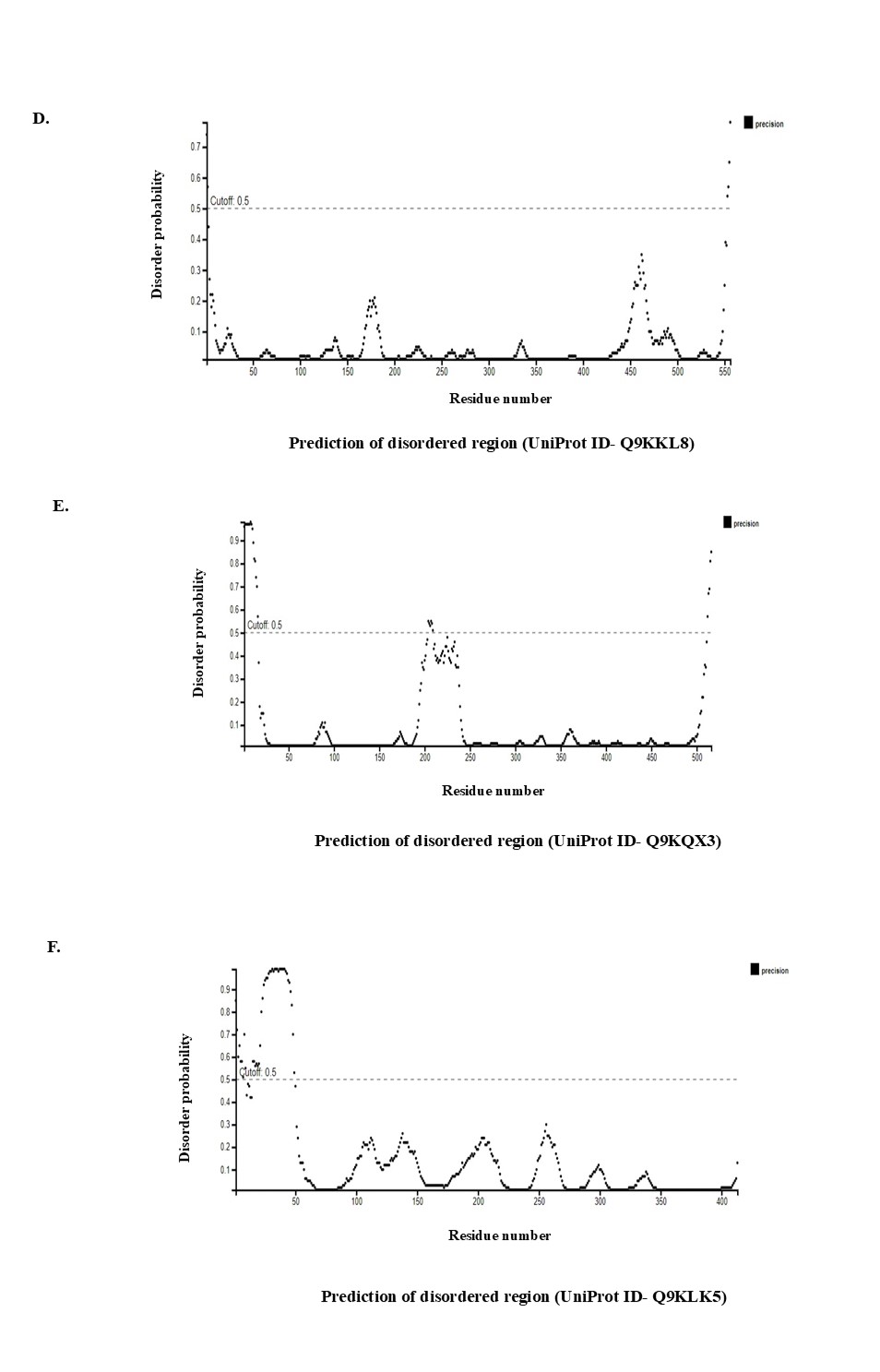


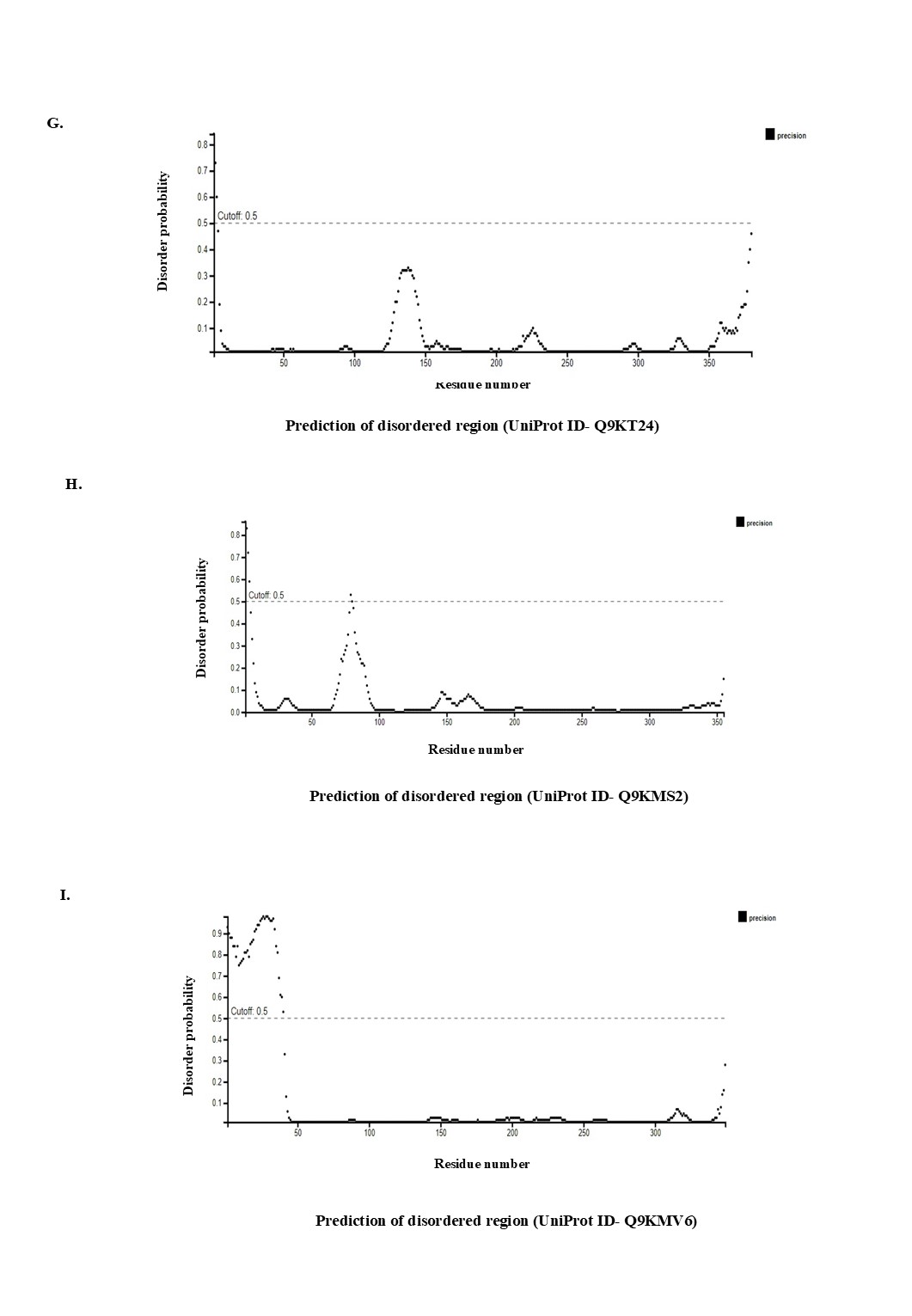


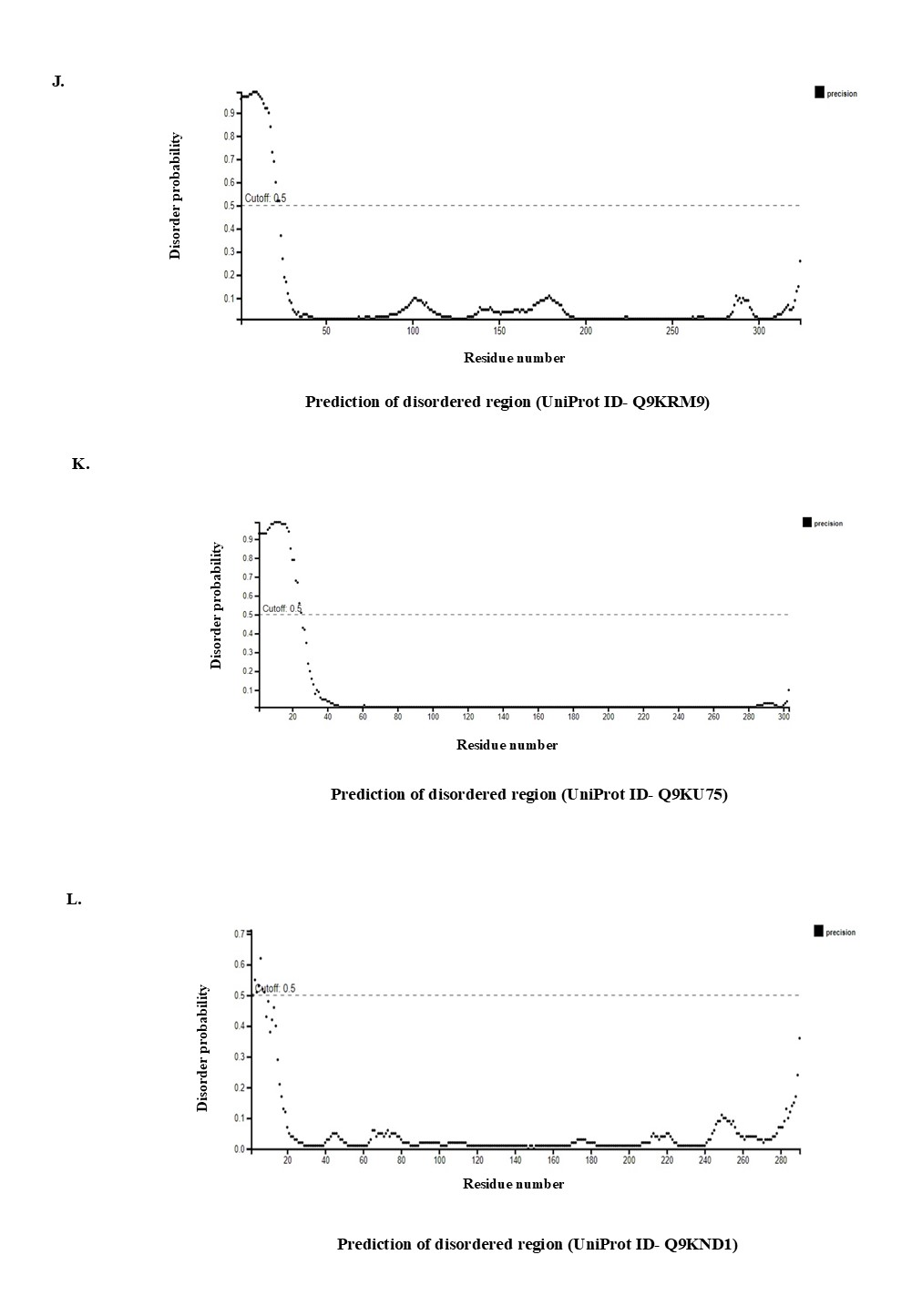


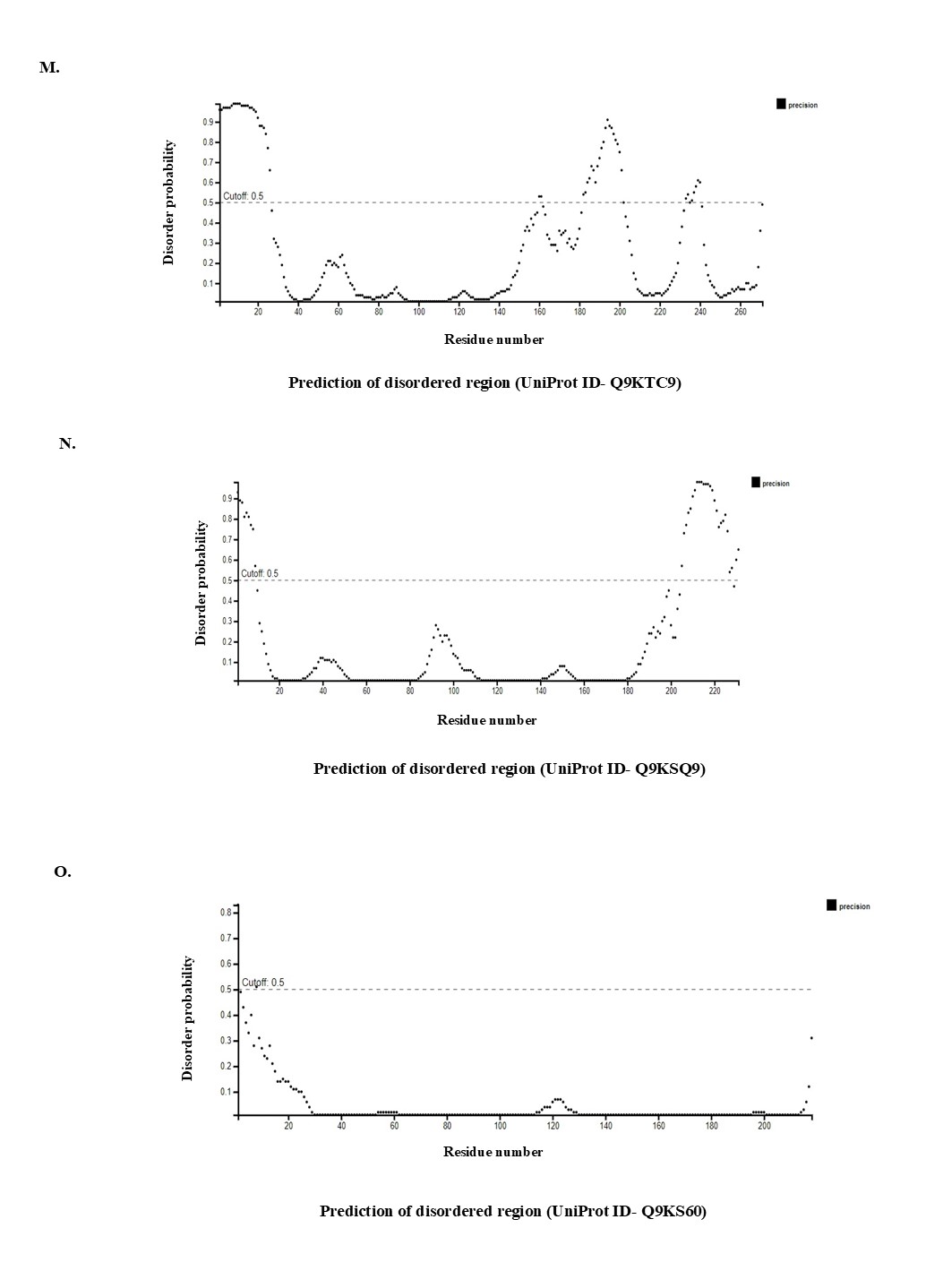


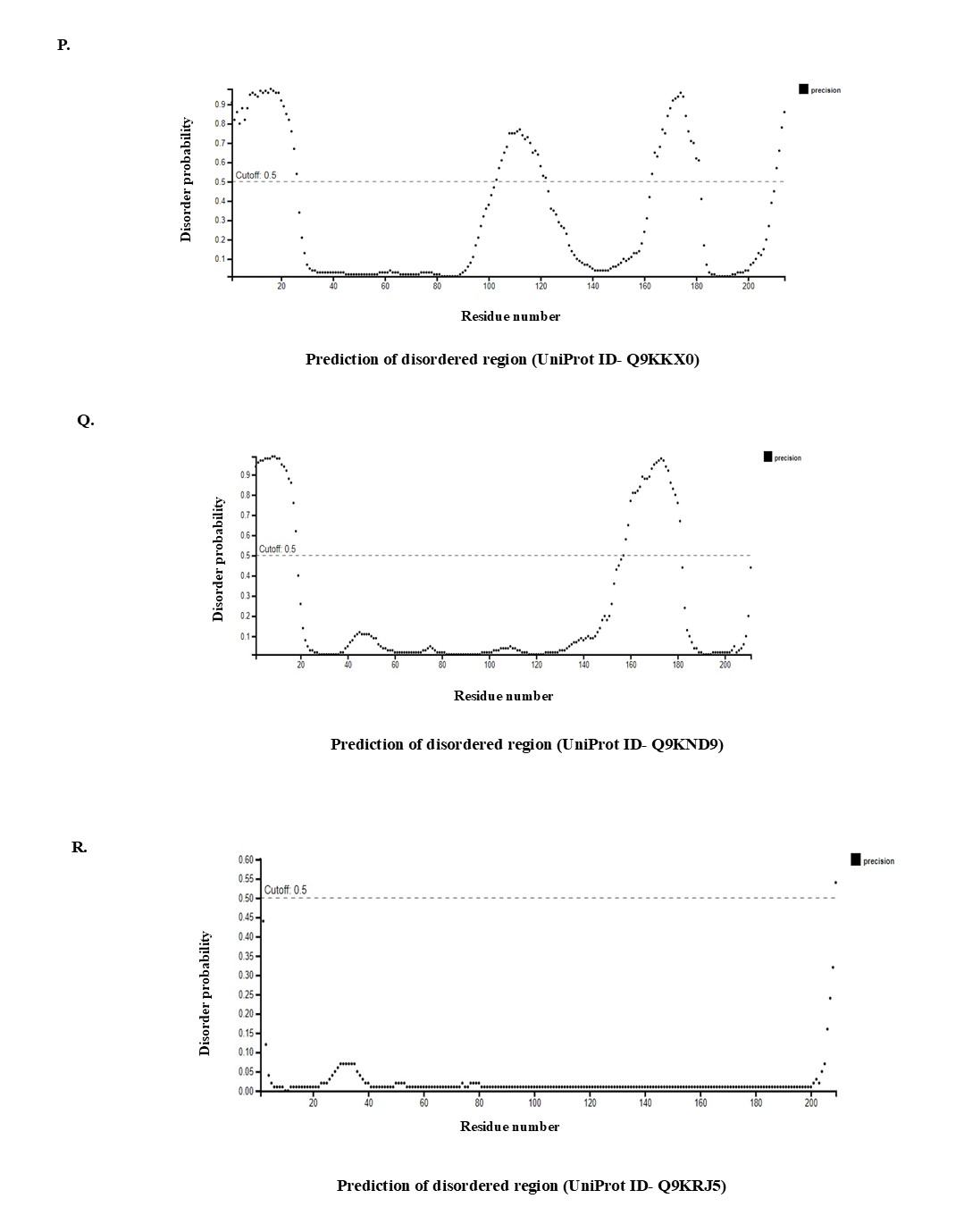


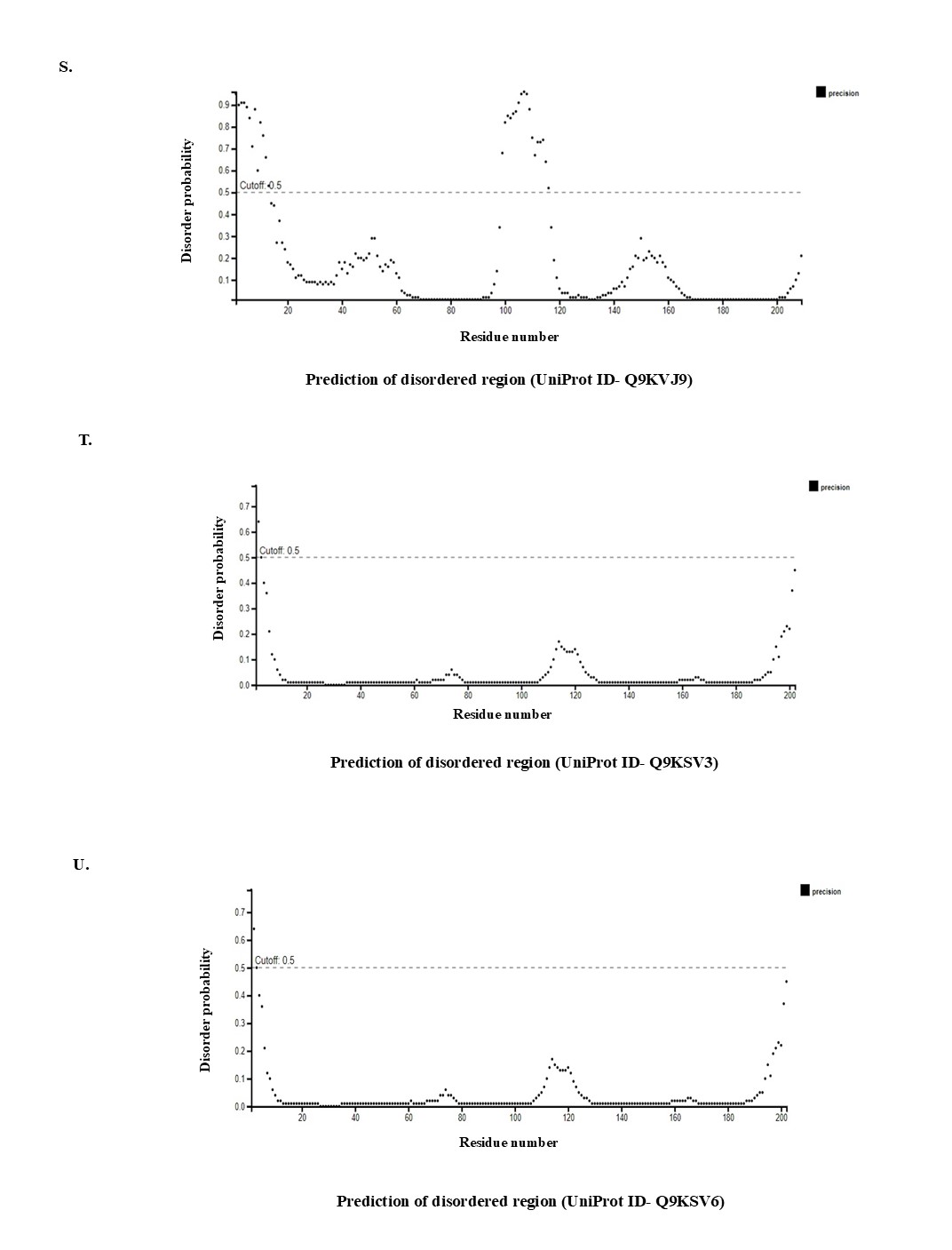


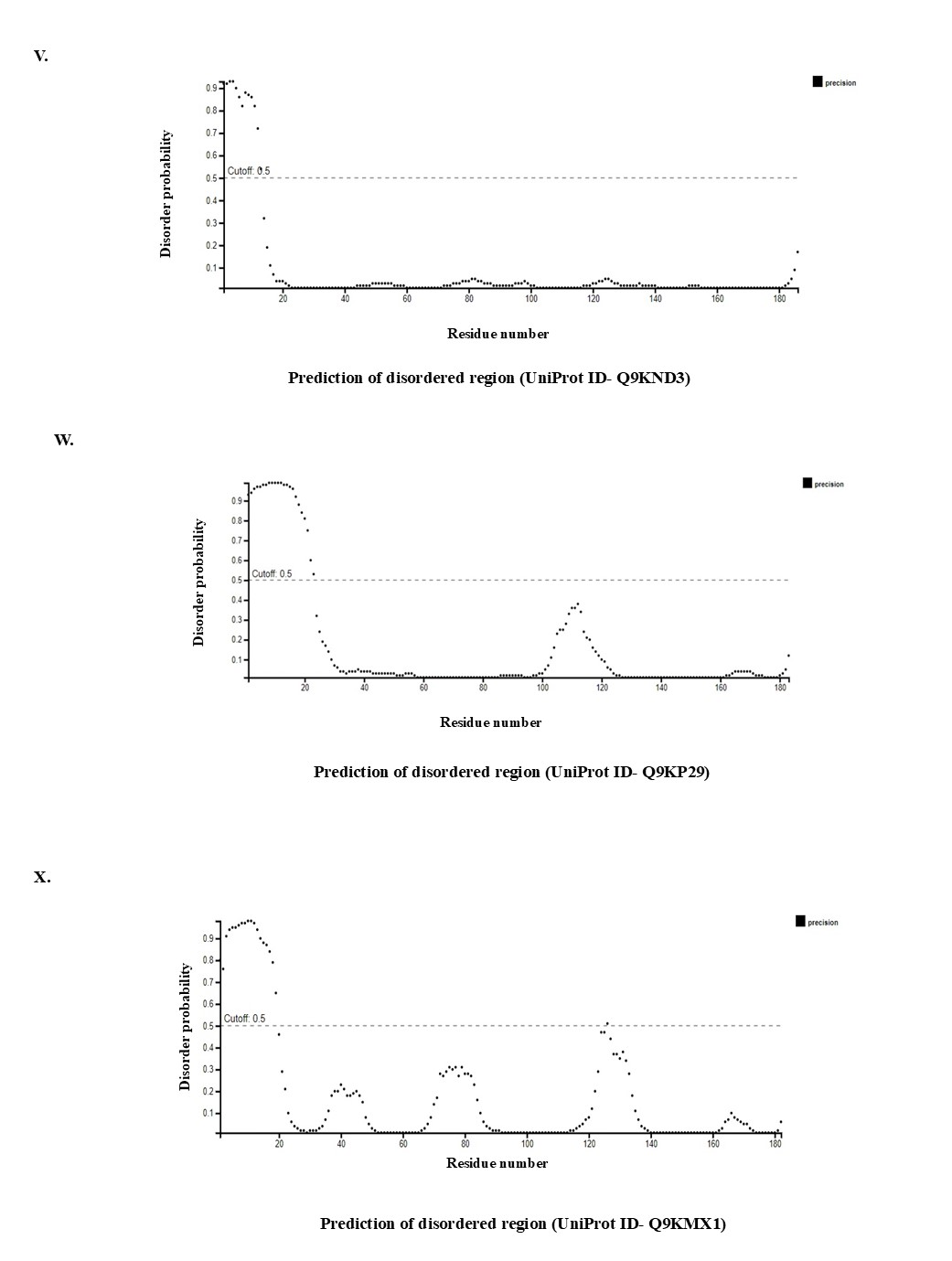


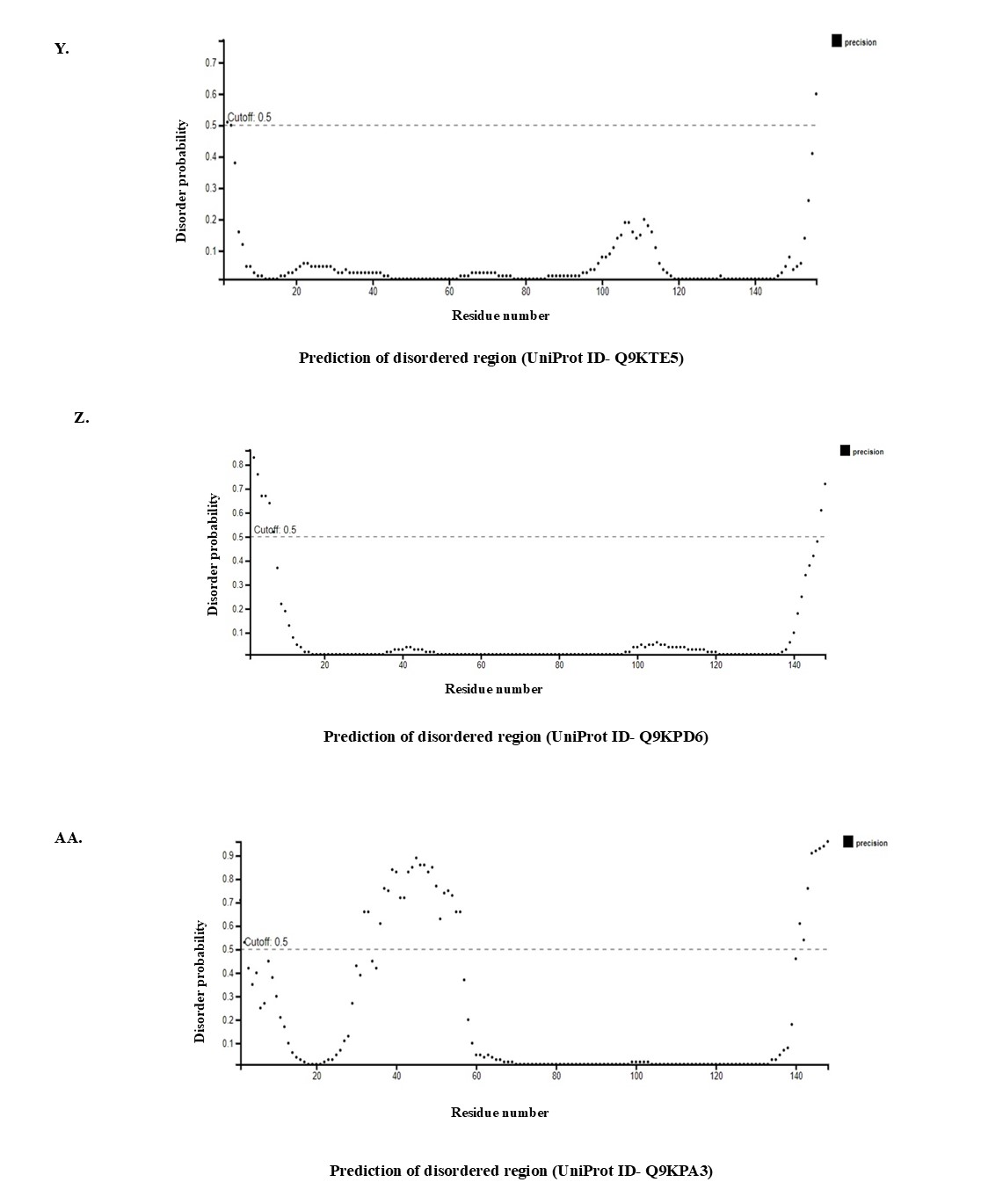


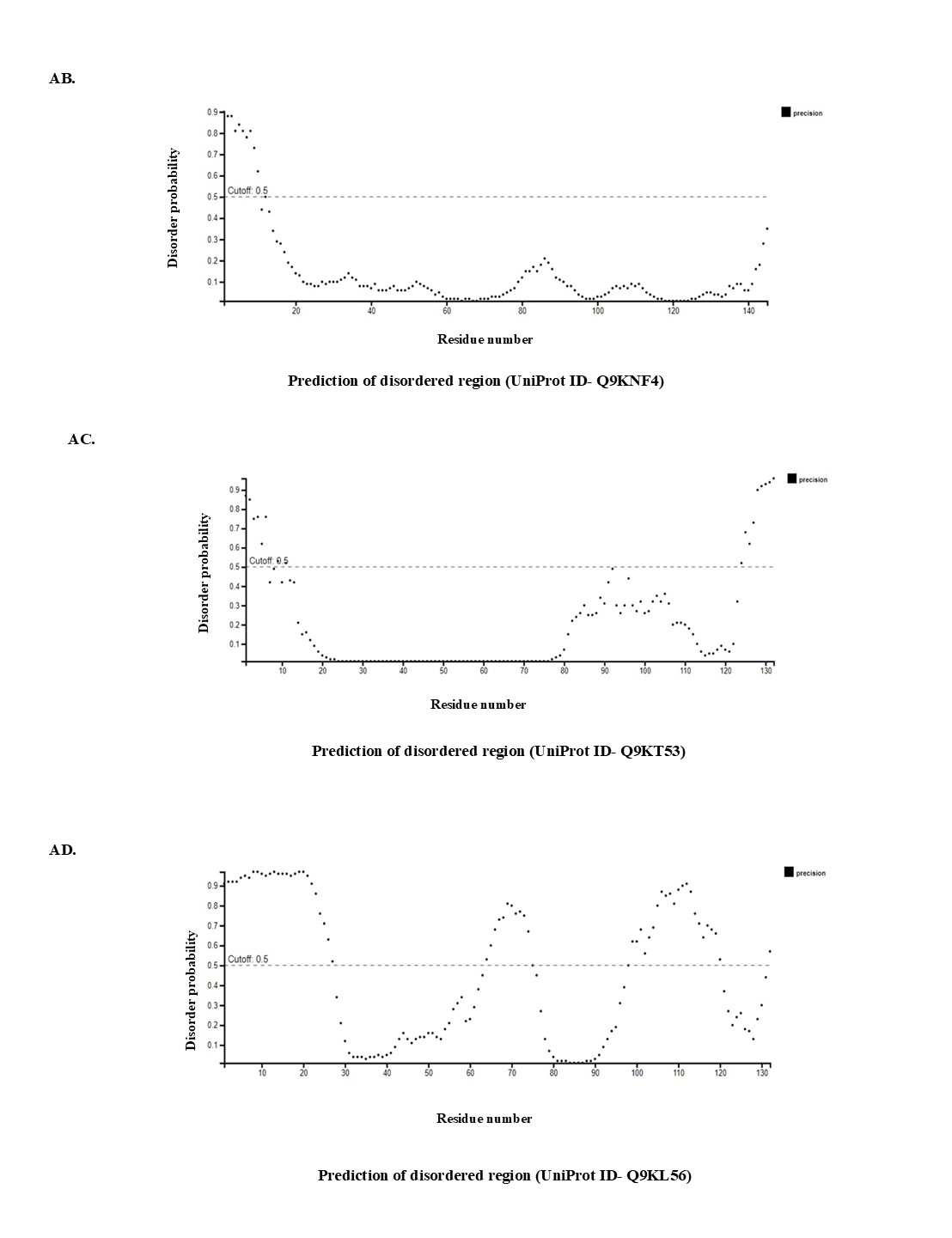


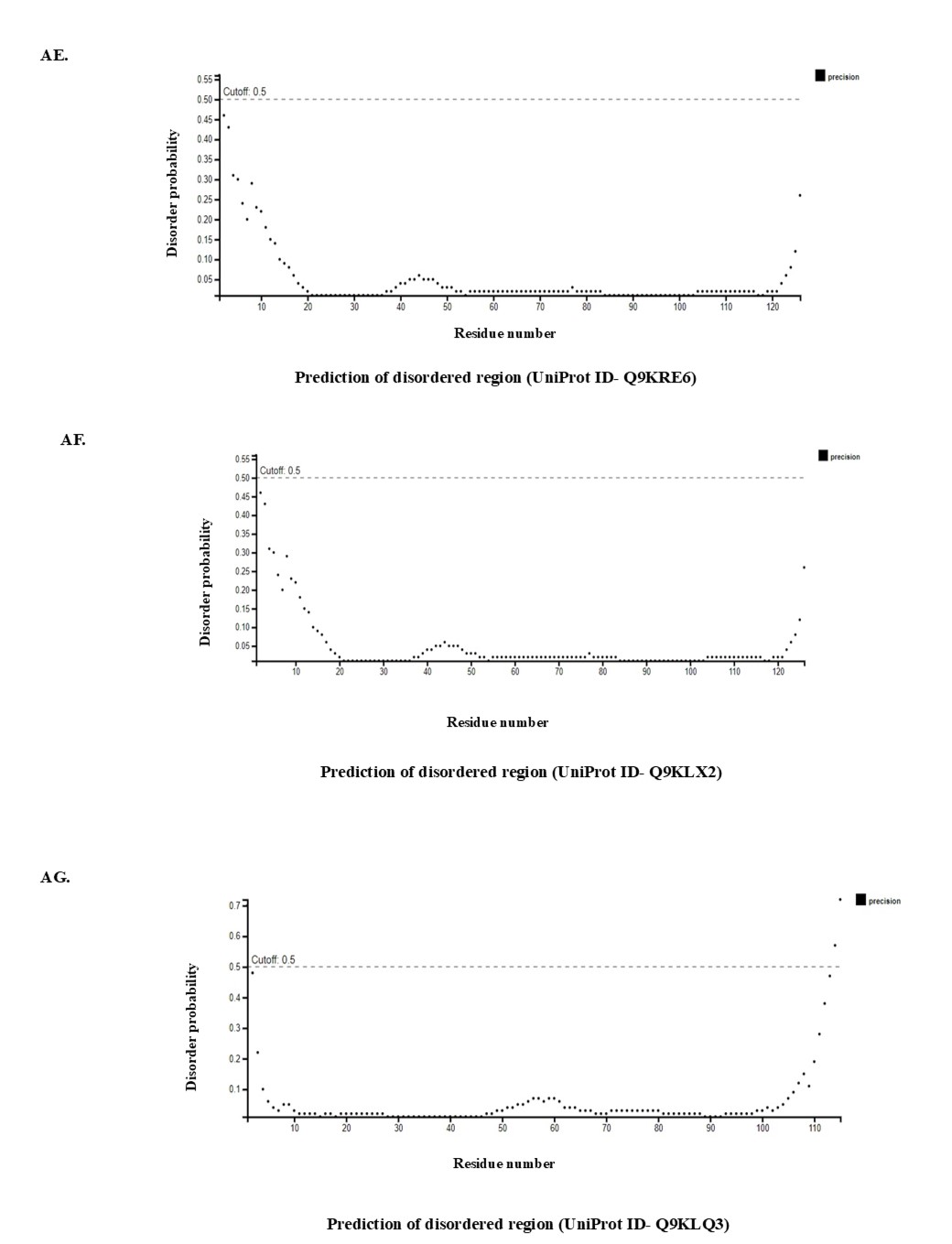


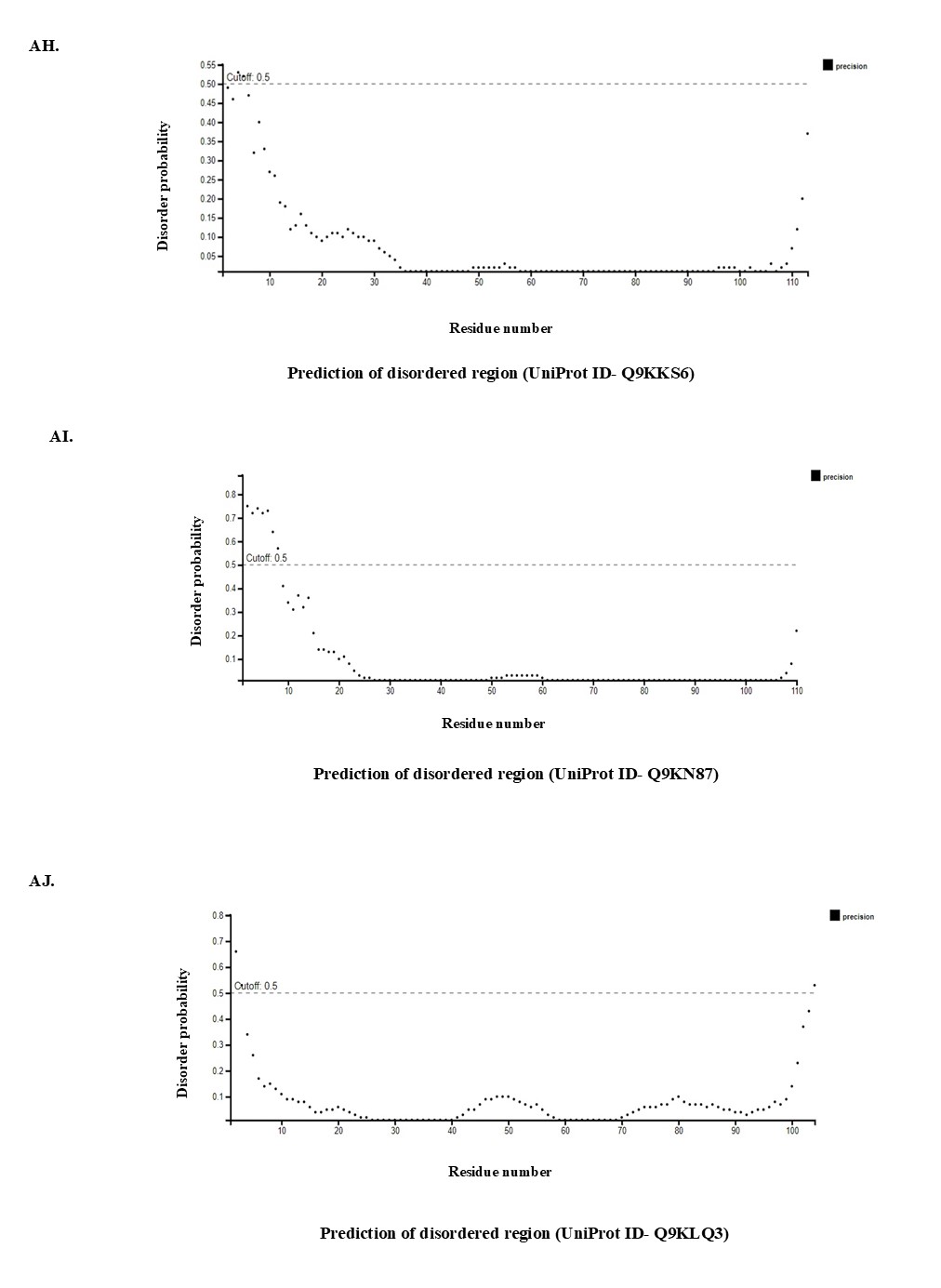


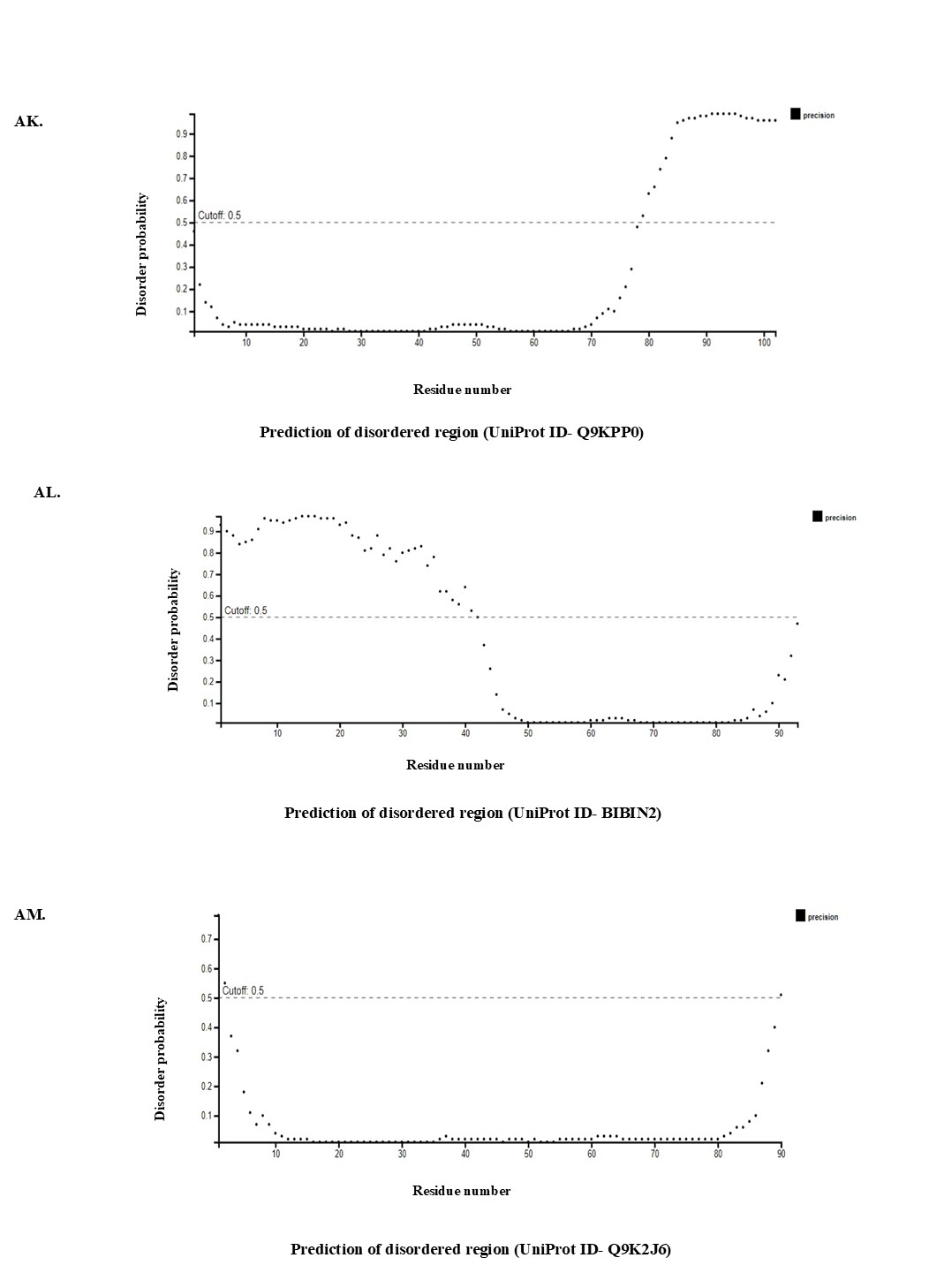


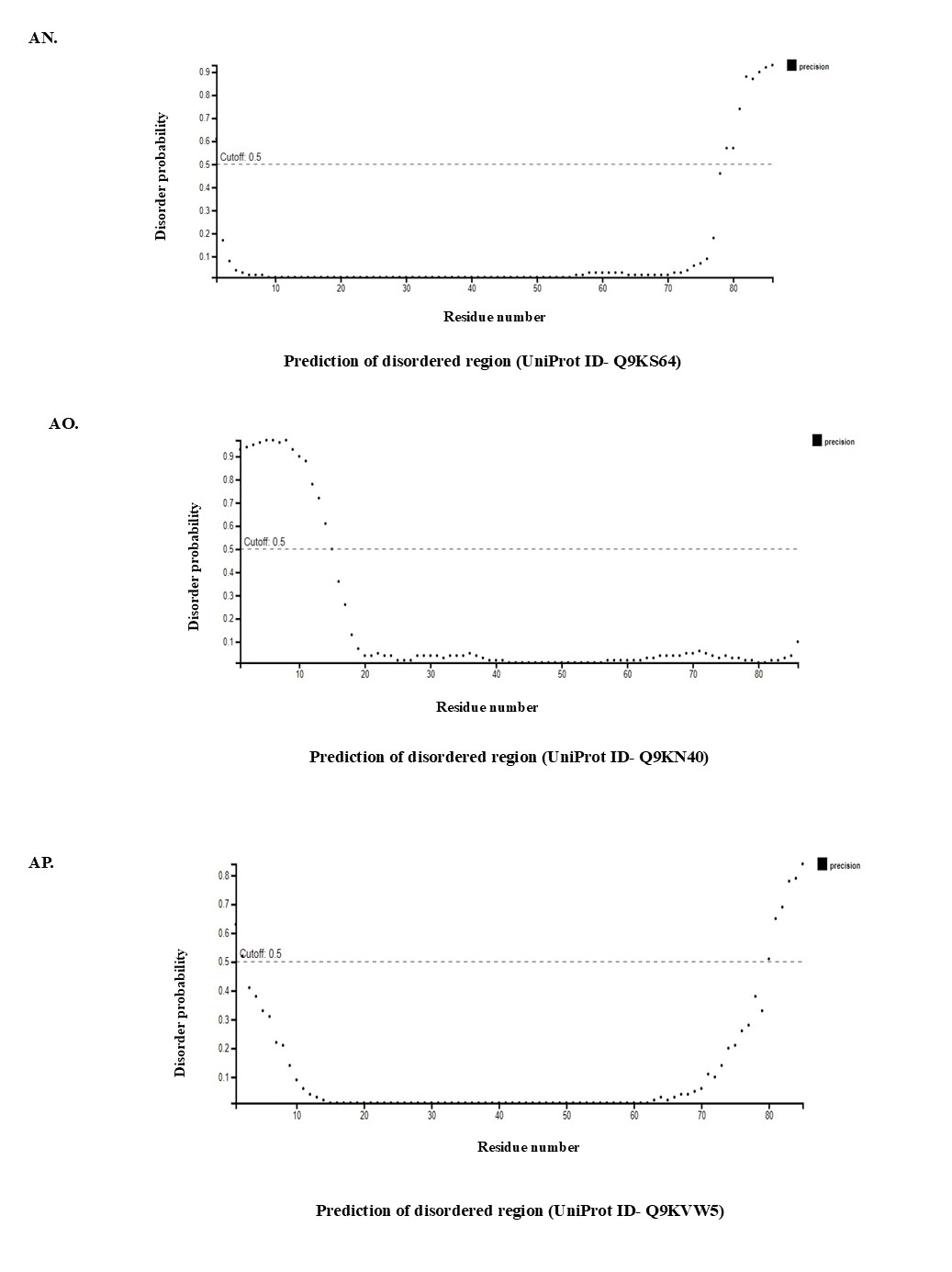


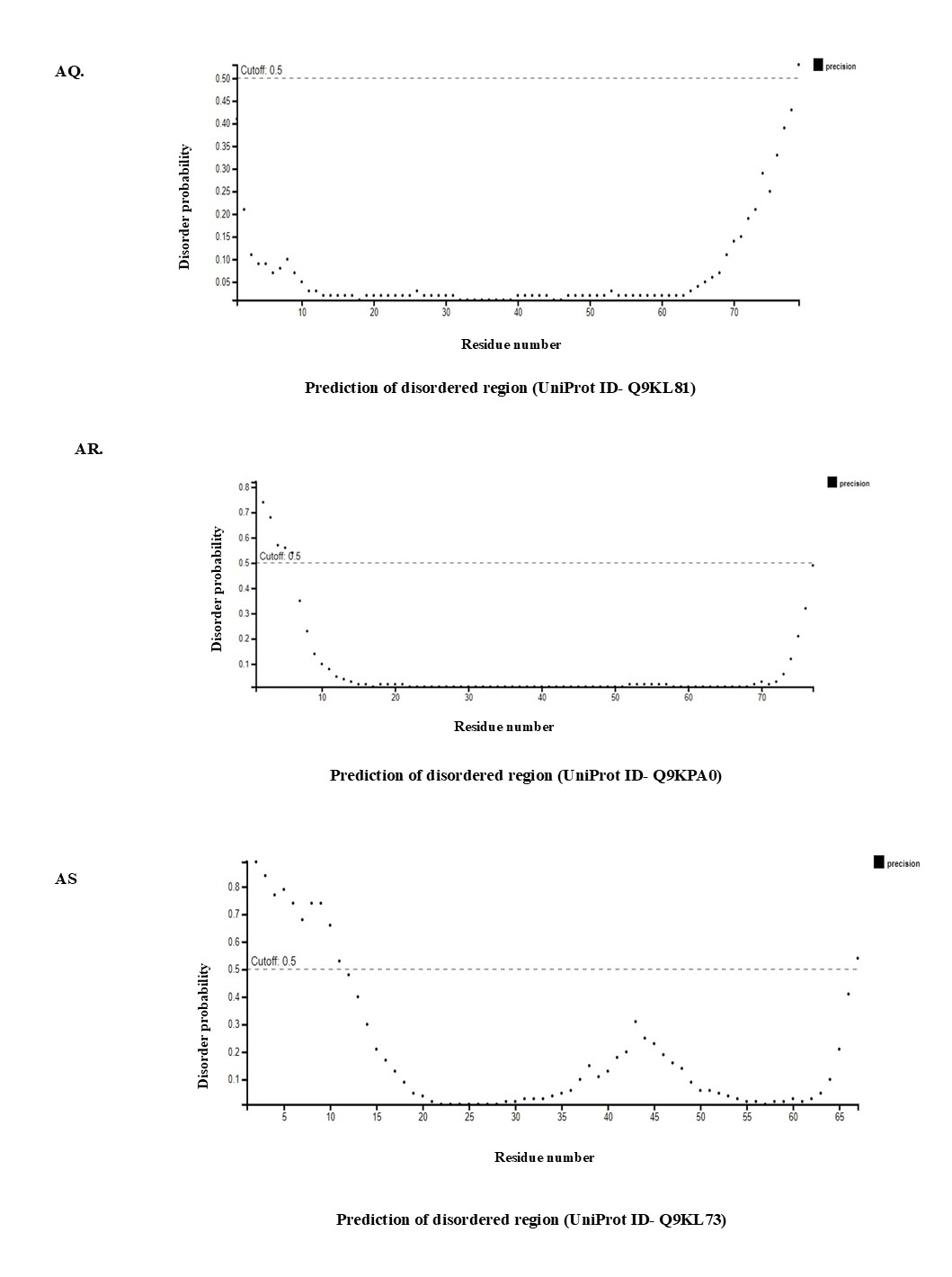


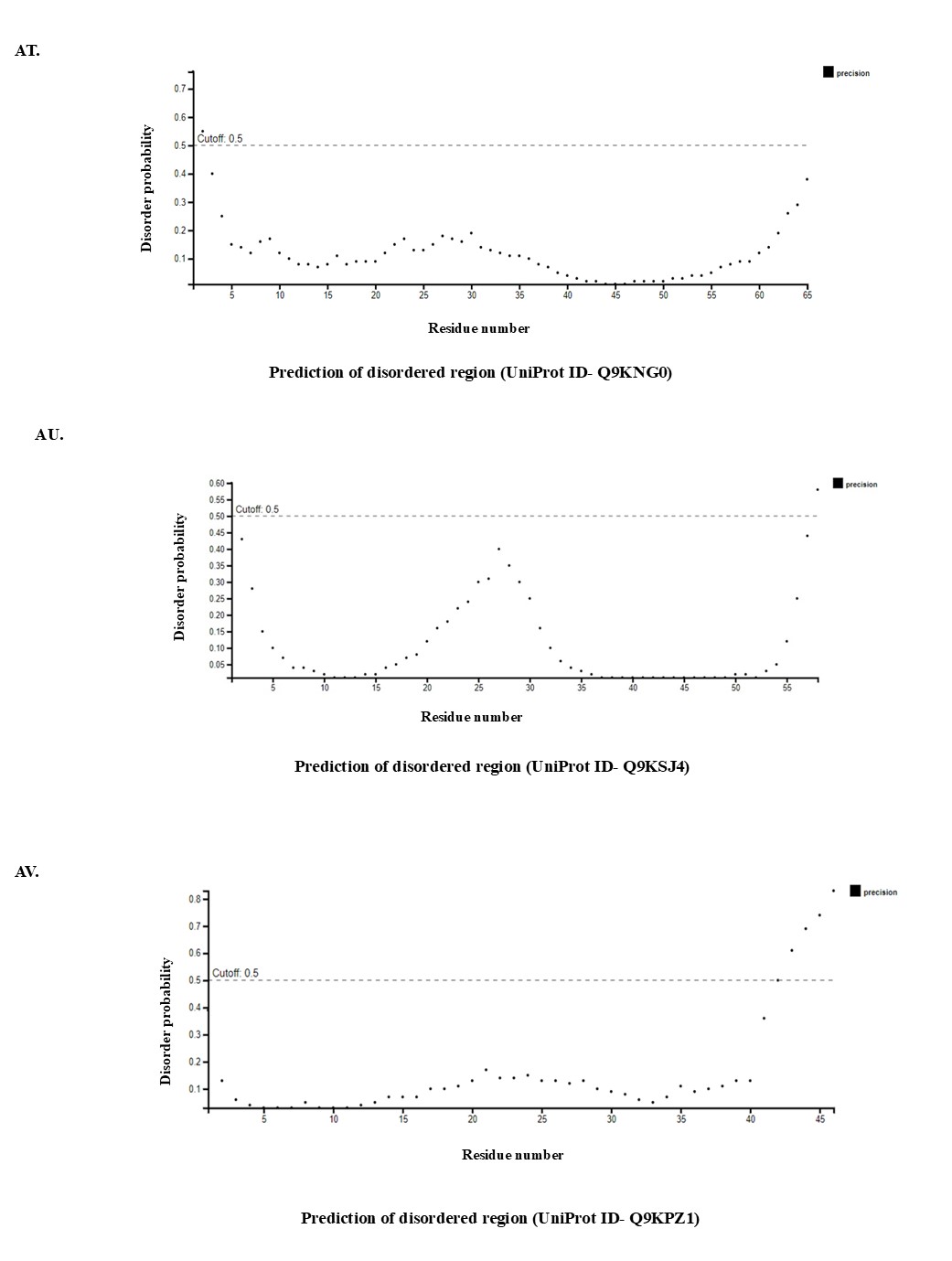


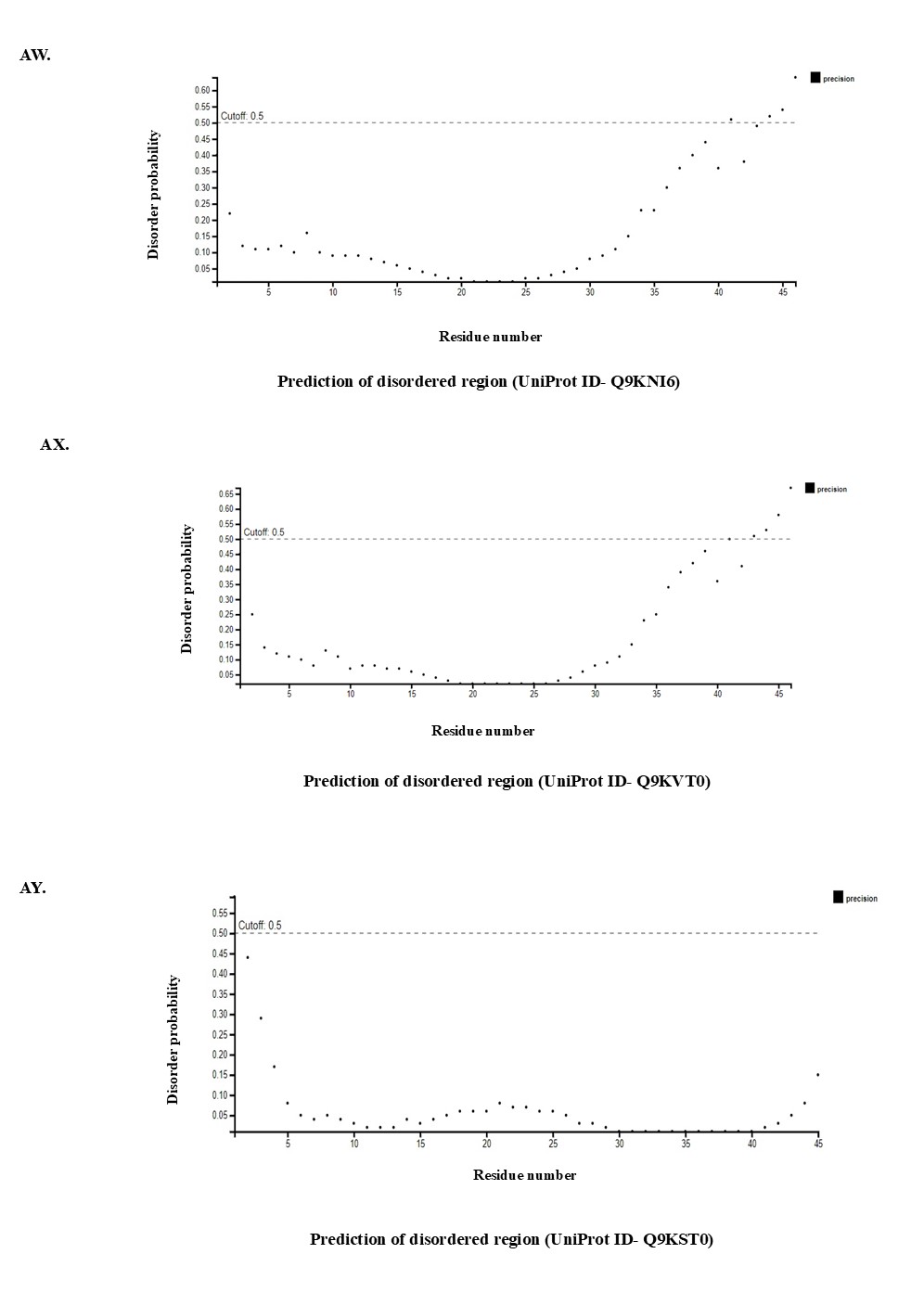

Supplement: S4 Fig — (DOCX) [file pone.0311301.s019.docx]
