# Supplementary material for: Comprehensive in silico analyses of fifty-one uncharacterized proteins from Vibrio cholerae
Source: PLoS One. 2024 Oct 4;19(10):e0311301. doi: 10.1371/journal.pone.0311301 (PMC11452002; doi:10.1371/journal.pone.0311301)
Supplement: S5 Fig — (DOCX) [file pone.0311301.s020.docx]

**Figure S5**

**Construction of 3-dimensional structures of uncharacterized proteins by ALphaFOLD-mmseq2 and validation of the structures.**


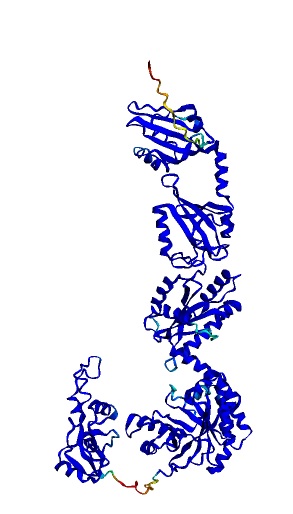


**A.**

**pLDDT value= 92.6**

**C**

**N**

**Model of the protein (UniProt ID- Q9KRD2) derived from AlphaFold-mmseq2**

**B.**


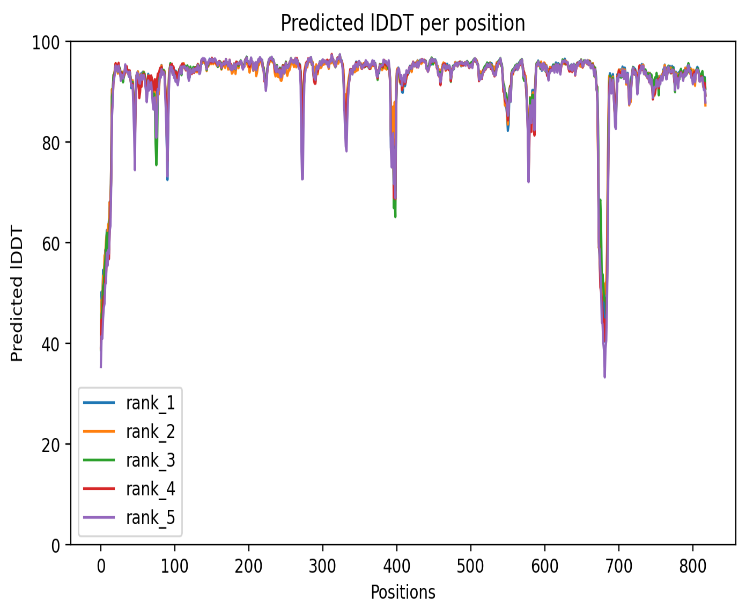

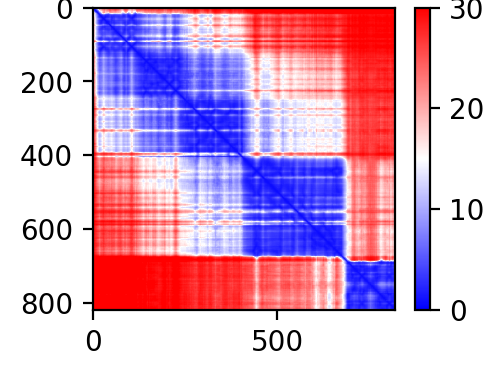


**C.**

**pLDDT graph for the model (UniProt ID-Q9KRD2)** **PAE graph for the model Q9KRD2**


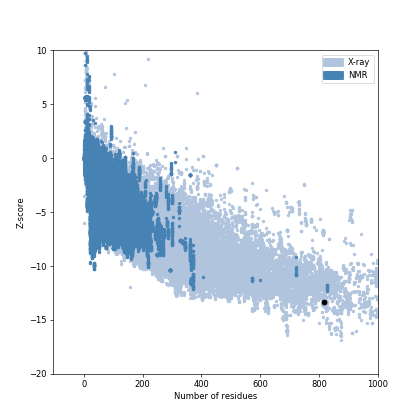


**Z-score= -13.31**

**D.**

**Overall model quality of the model structure of Q9KRD2 by ProSA**

**E.**


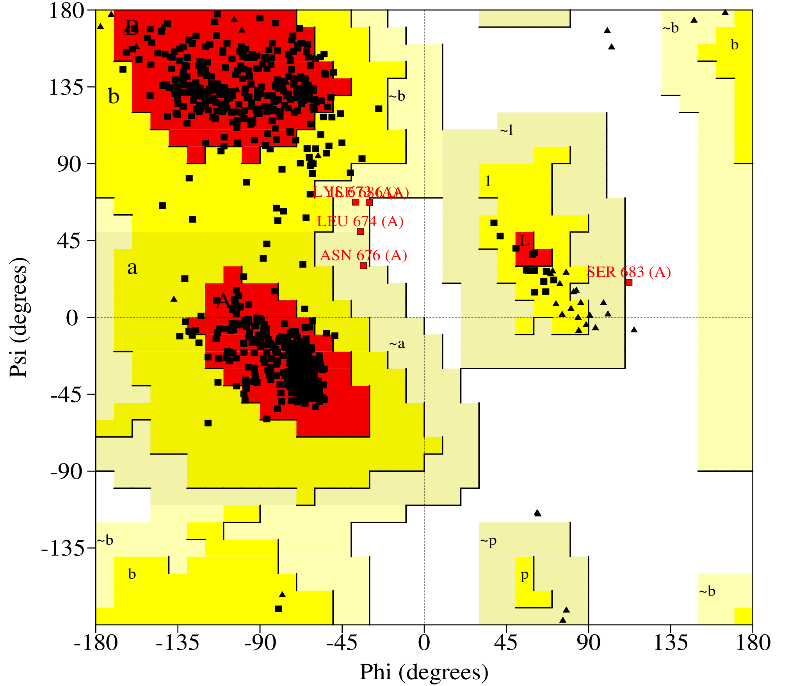


**Ramachandran plot for the model structure of Q9KRD2**

| Residues in most favoured region [A, B, L] | 676 | 91.7% |
| --- | --- | --- |
| Residues in additional allowed region [a,b,l,p] | 56 | 7.6% |
| Residues in generously allowed regions [~a, ~b, ~l, ~p] | 4 | 0.5% |
| Residues in disallowed regions | 1 | 0.1% |
| Total (Number of non-glycine and non-proline residues) | 737 | 100% |
| Number of end-residues (excl. Gly and Pro) | 2 |  |
| Number of Glycine | 48 |  |
| Number of Proline | 31 |  |
| Total number of residues | 818 |  |

**F.**

**Ramachandran plot statistics for the model structure of Q9KRD2**


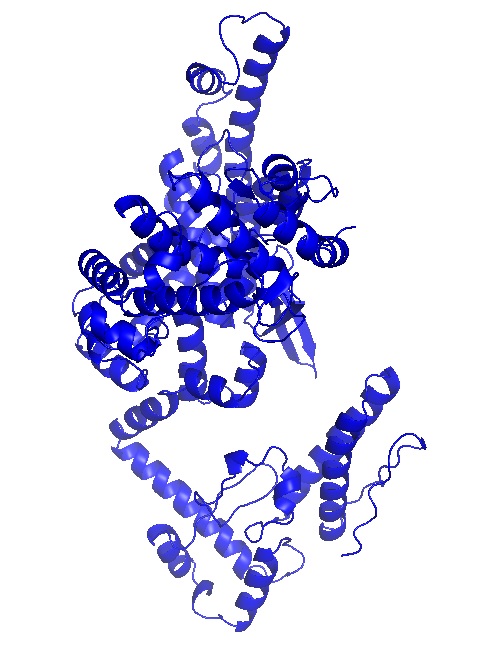


**N**

**C**

**pLDDT value= 92.1**

**G.**

**Model of the protein (UniProt ID- Q9KVG3) derived from AlphaFold-mmseq2**

**H.**


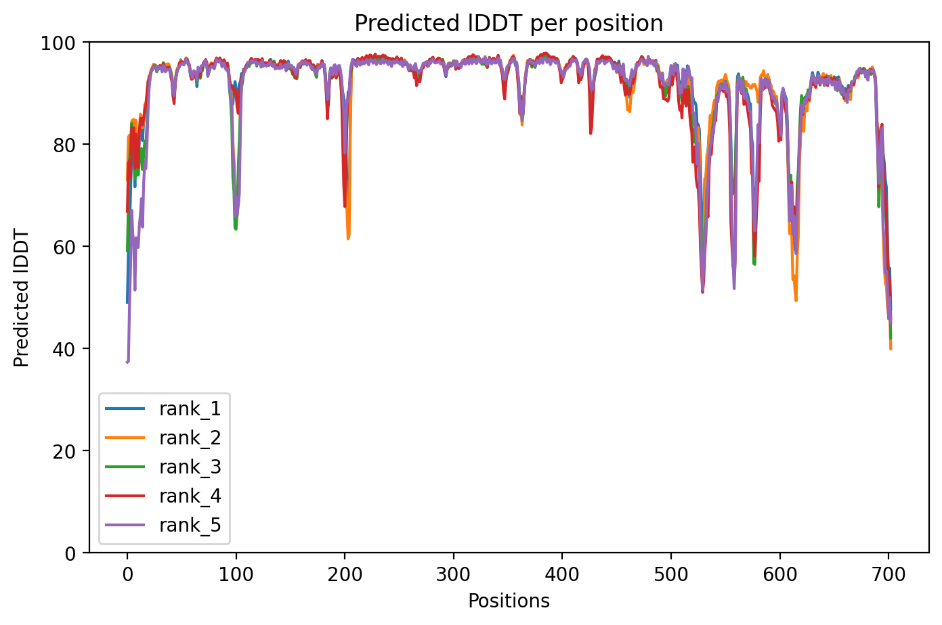

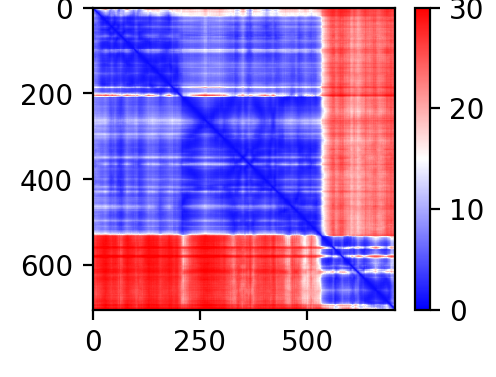


**I.**

**pLDDT graph for the model (UniProt ID-Q9KVG3) PAE graph for the model Q9KVG3**


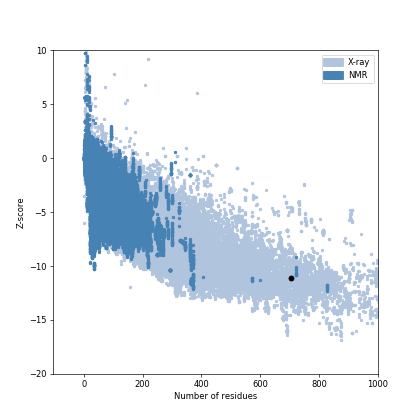


**J.**

**Z-score= -11.11**

**Overall model quality of the model structure of Q9KVG3 by ProSA**

**K.**


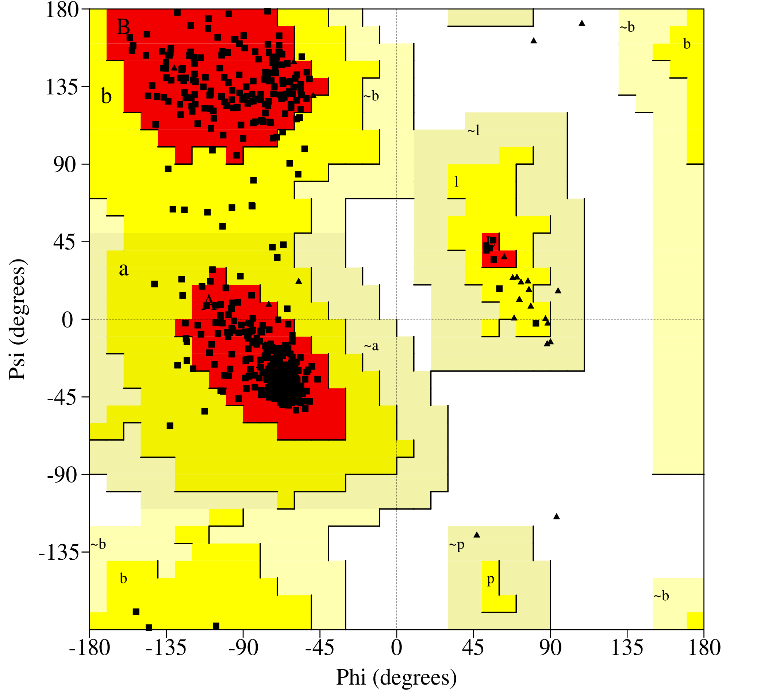


**Ramachandran plot for the model structure of Q9KVG3**

| Residues in most favoured region [A, B, L] | 610 | 94.6% |
| --- | --- | --- |
| Residues in additional allowed region [a,b,l,p] | 35 | 5.4% |
| Residues in generously allowed regions [~a, ~b, ~l, ~p] | 0 | 0.0% |
| Residues in disallowed regions | 0 | 0.0% |
| Total (Number of non-glycine and non-proline residues) | 645 | 100% |
| Number of end-residues (excl. Gly and Pro) | 1 |  |
| Number of Glycine | 38 |  |
| Number of Proline | 19 |  |
| Total number of residues | 703 |  |

**L.**

**Ramachandran plot statistics for the model structure of Q9KVG3**


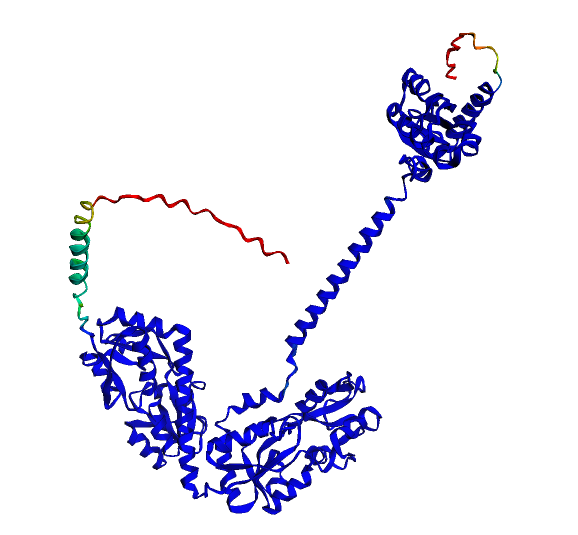


**M.**

**pLDDT value= 91.8**

**N**

**C**

**Model of the protein (UniProt ID- Q9KT38) derived from AlphaFold-mmseq2**

**N.**


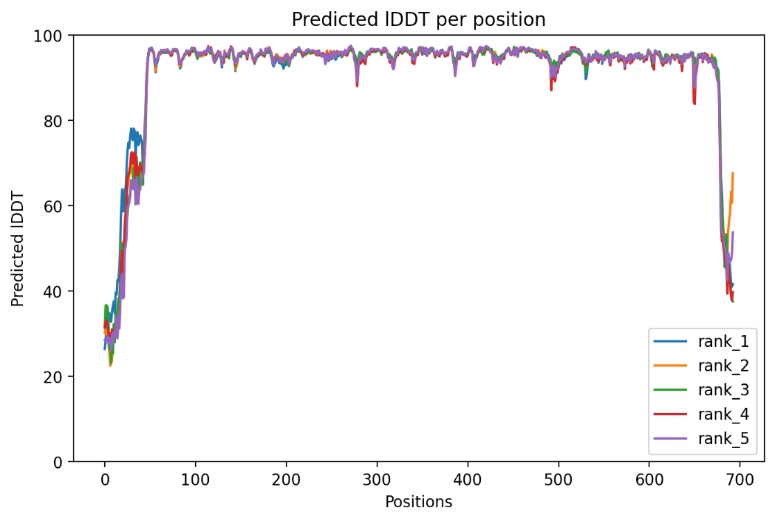
 ***
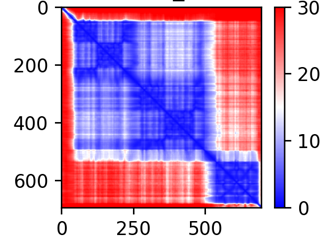
***

**O.**

**pLDDT graph for the model (UniProt ID-Q9KT38) PAE graph for the model Q9KT38**


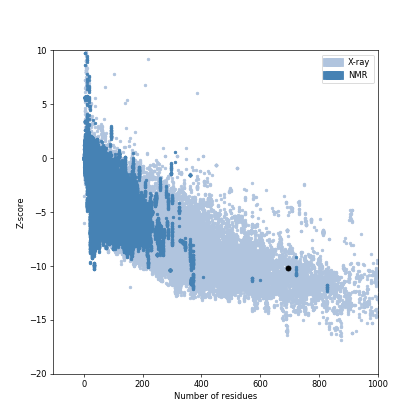


**Z-score= -10.22**

**P.**

**Overall model quality of the model structure of Q9KT38 by ProSA**

**Q.**


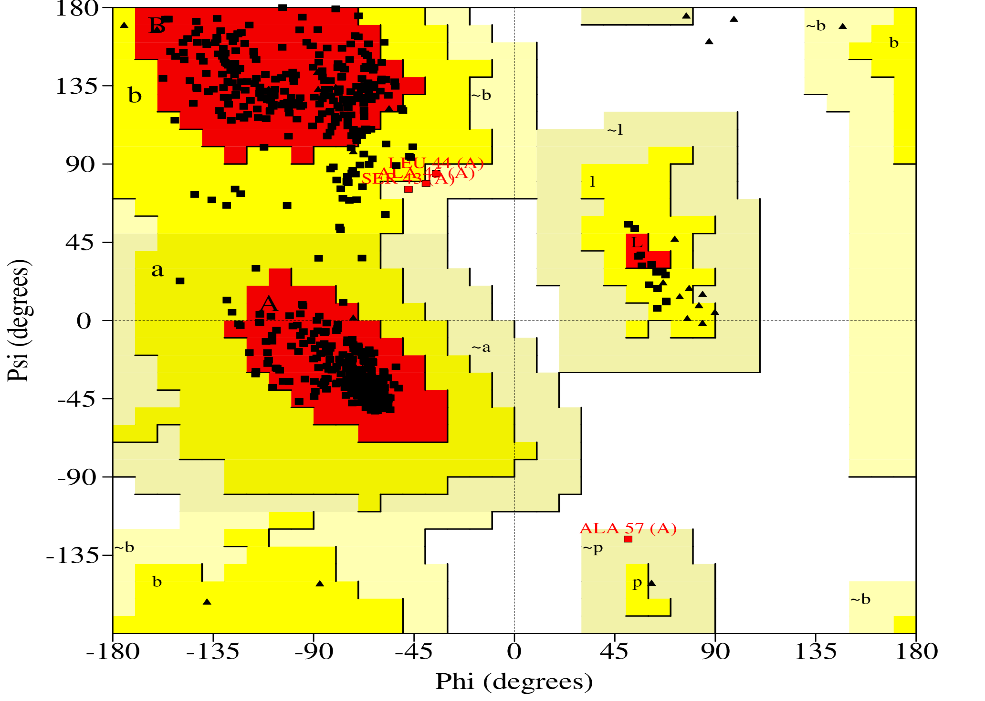


**Ramachandran plot for the model structure of Q9KT38**

| Residues in most favoured region [A, B, L] | 580 | 90.8% |
| --- | --- | --- |
| Residues in additional allowed region [a,b,l,p] | 55 | 8.6% |
| Residues in generously allowed regions [~a, ~b, ~l, ~p] | 4 | 0.6% |
| Residues in disallowed regions | 0 | 0.0% |
| Total (Number of non-glycine and non-proline residues) | 639 | 100% |
| Number of end-residues (excl. Gly and Pro) | 2 |  |
| Number of Glycine | 32 |  |
| Number of Proline | 20 |  |
| Total number of residues | 693 |  |

**R.**

**Ramachandran plot statistics for the model structure of Q9KT38**

**S.**

**pLDDT value= 94.9**


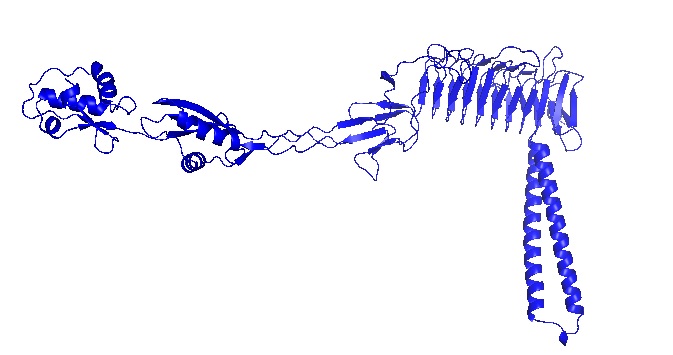


**C**

**N**

**Model of the protein (UniProt ID- Q9KKL8) derived from AlphaFold-mmseq2**

**T.**


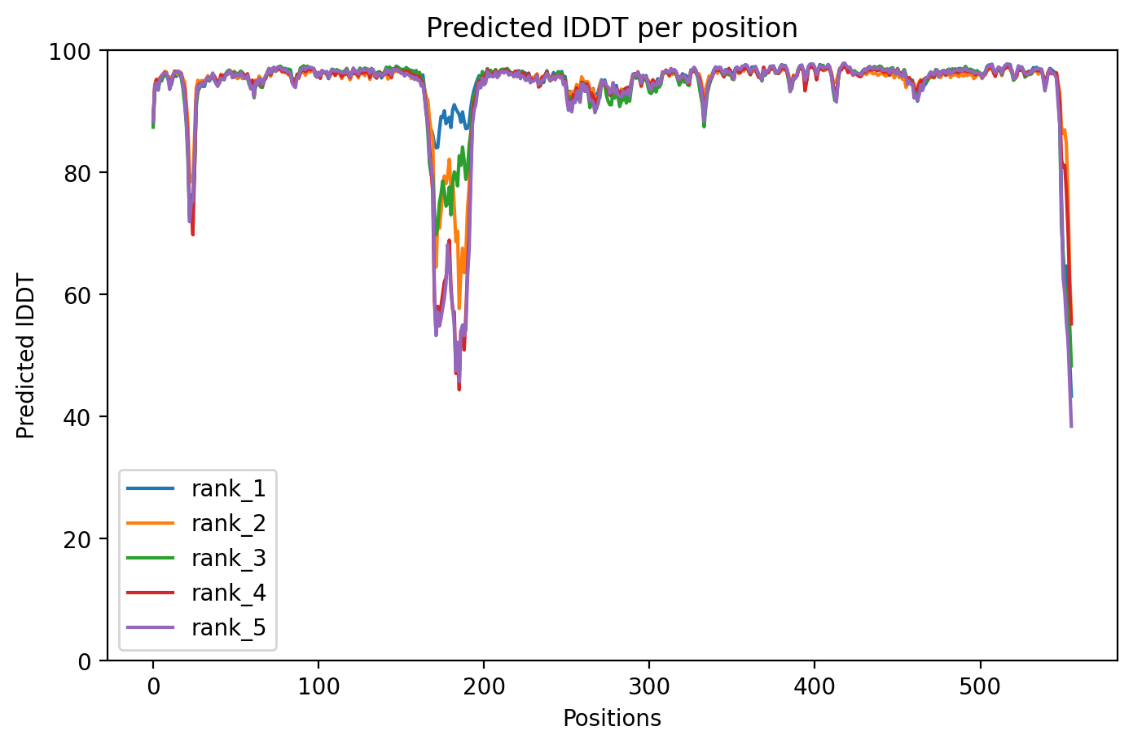


**pLDDT graph for the model (UniProt ID-Q9KKL8)**

***
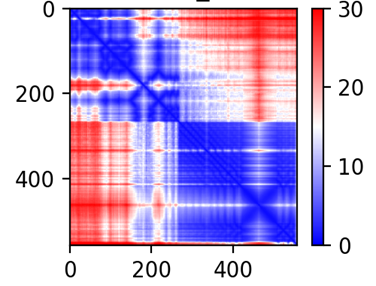
***

**U.**

**PAE graph for the model Q9KKL8**


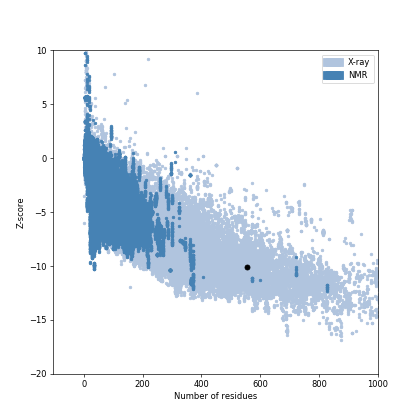


**V.**

**Z-score= -10.06**

**Overall model quality of the model structure of Q9KKL8 by ProSA**

**W.**


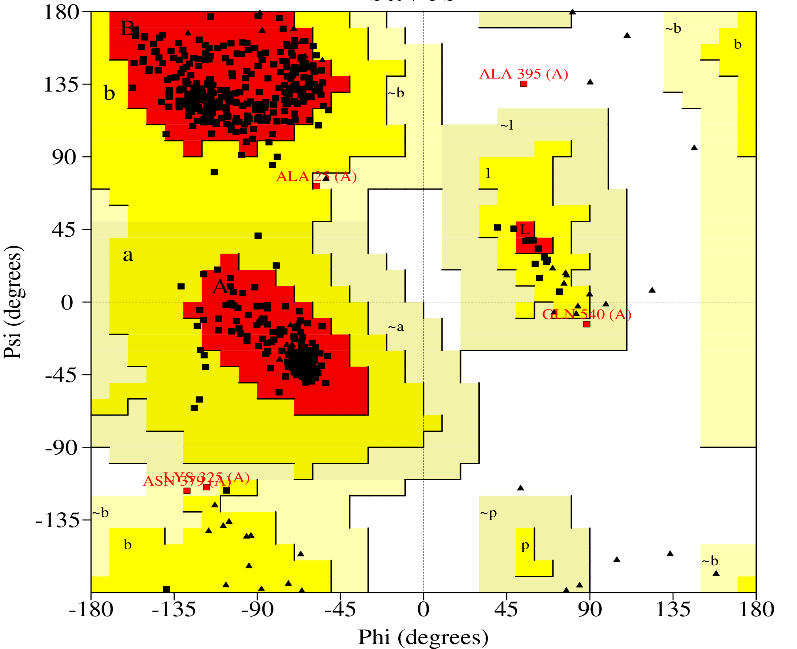


**Ramachandran plot for the model structure of Q9KKL8**

| Residues in most favoured region [A, B, L] | 454 | 93.6% |
| --- | --- | --- |
| Residues in additional allowed region [a,b,l,p] | 26 | 5.4% |
| Residues in generously allowed regions [~a, ~b, ~l, ~p] | 4 | 0.8% |
| Residues in disallowed regions | 1 | 0.2% |
| Total (Number of non-glycine and non-proline residues) | 485 | 100% |
| Number of end-residues (excl. Gly and Pro) | 2 |  |
| Number of Glycine | 51 |  |
| Number of Proline | 18 |  |
| Total number of residues | 556 |  |

**X.**

**Ramachandran plot statistics for the model structure of Q9KKL8**

**Y.**


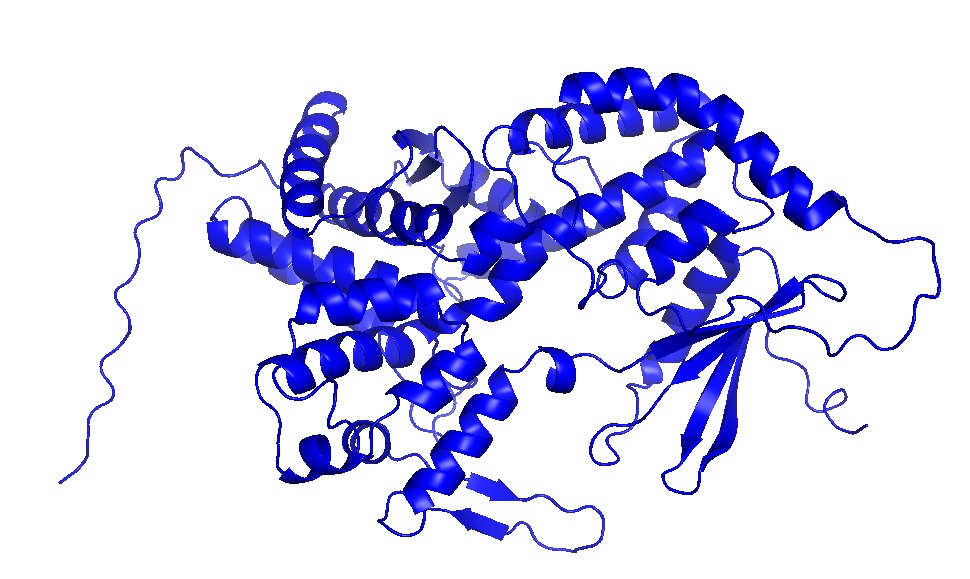


**pLDDT value= 94.2**

**N**

**C**

**Model of the protein (UniProt ID- Q9KQX5) derived from AlphaFold-mmseq2**

**Z.**


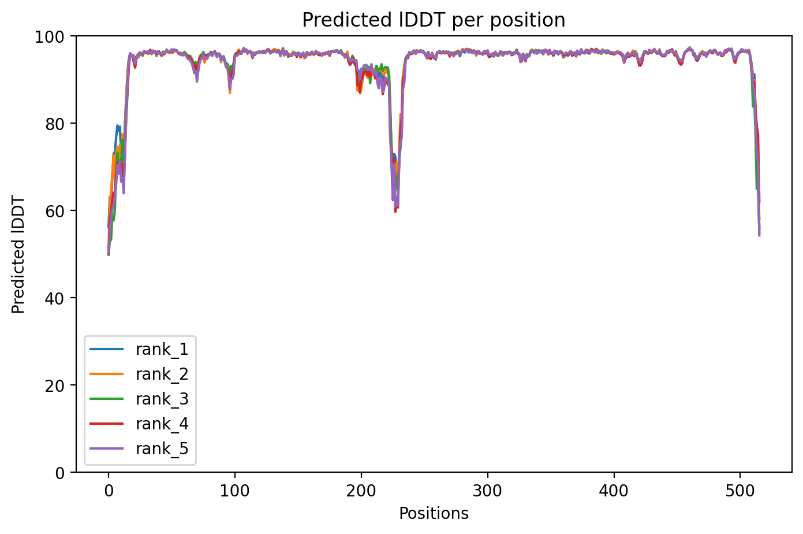
 ***
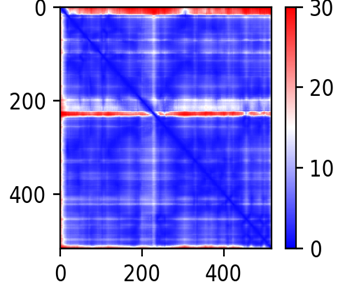
***

**AA.**

**pLDDT graph for the model (UniProt ID-Q9KQX5) PAE graph for the model Q9KQX5**


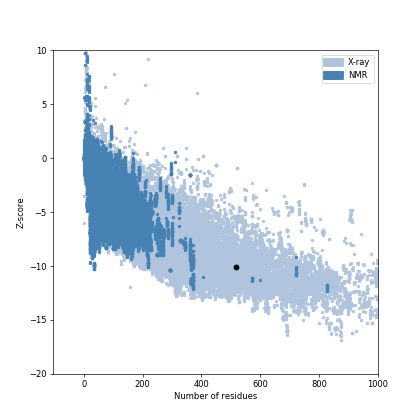


**AB.**

**Z-score= -10.12**

**Overall model quality of the model structure of Q9KQX5 by ProSA**

**AC.**


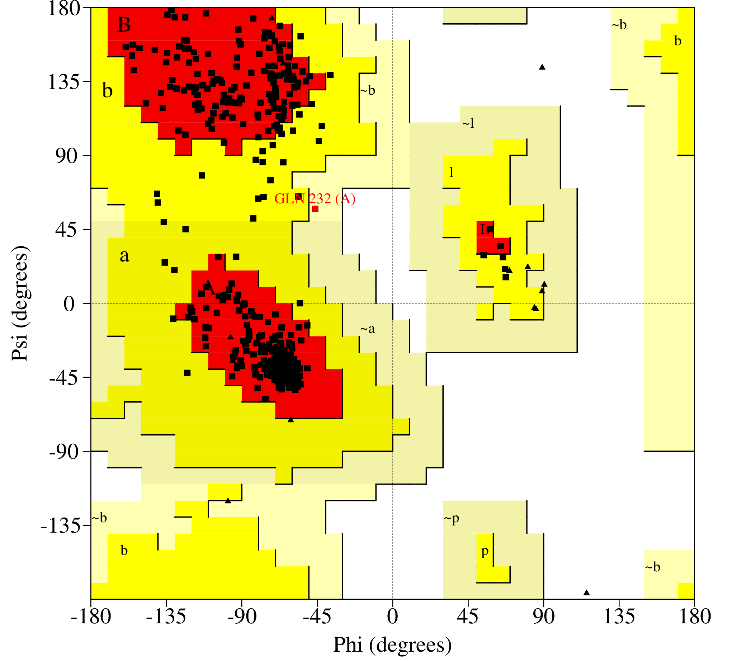


**Ramachandran plot for the model structure of of Q9KQX5**

| Residues in most favoured region [A, B, L] | 435 | 92.2% |
| --- | --- | --- |
| Residues in additional allowed region [a,b,l,p] | 36 | 7.6% |
| Residues in generously allowed regions [~a, ~b, ~l, ~p] | 1 | 0.2% |
| Residues in disallowed regions | 0 | 0.0% |
| Total (Number of non-glycine and non-proline residues) | 472 | 100% |
| Number of end-residues (excl. Gly and Pro) | 2 |  |
| Number of Glycine | 18 |  |
| Number of Proline | 24 |  |
| Total number of residues | 516 |  |

**AD.**

**Ramachandran plot statistics for the model structure of Q9KQX5**


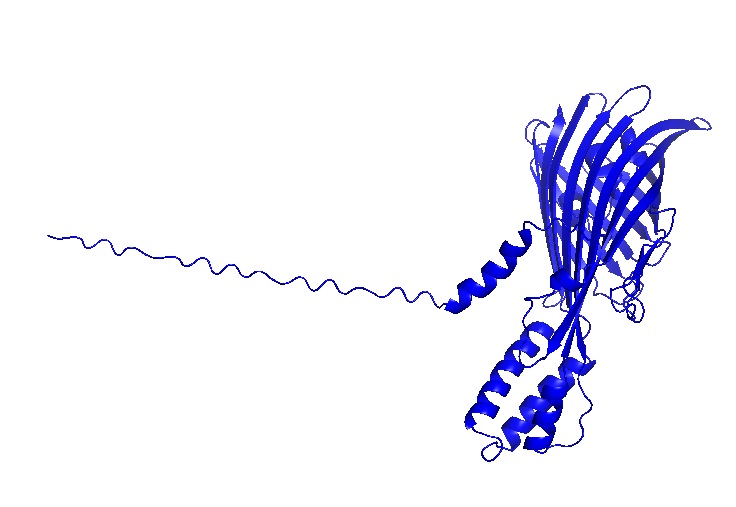


**AE.**

**N**

**C**

**pLDDT value= 88.6**

**Model of the protein (UniProt ID- Q9KLK5) derived from AlphaFold-mmseq2**

**AF.**


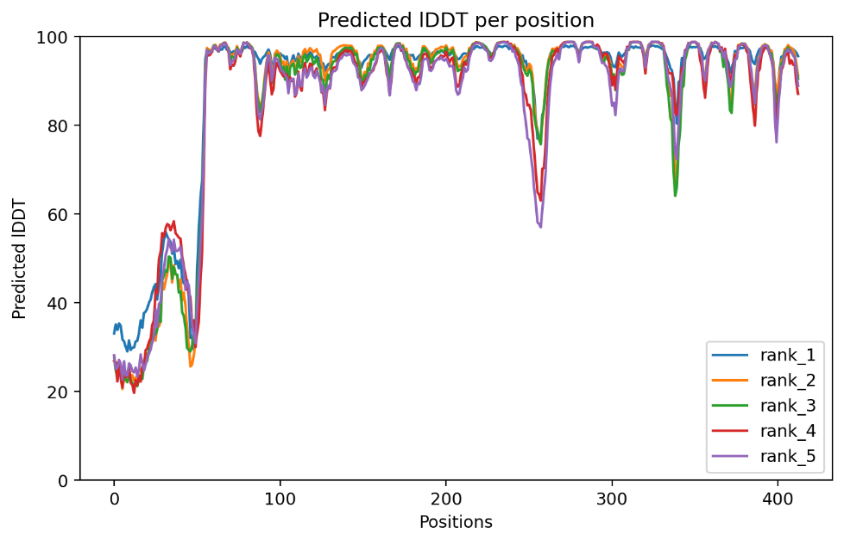
 ***
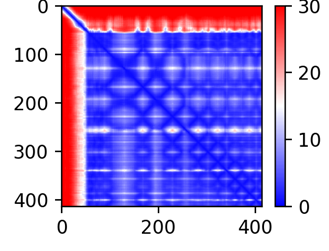
***

**AG.**

**pLDDT graph for the model (UniProt ID-Q9KLK5) PAE graph for the model Q9KLK5**


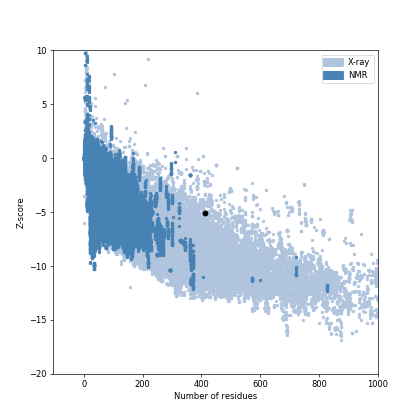


**AH.**

**Z-score= -5.09**

**Overall model quality of the model structure of Q9KLK5 by ProSA**

**AI.**


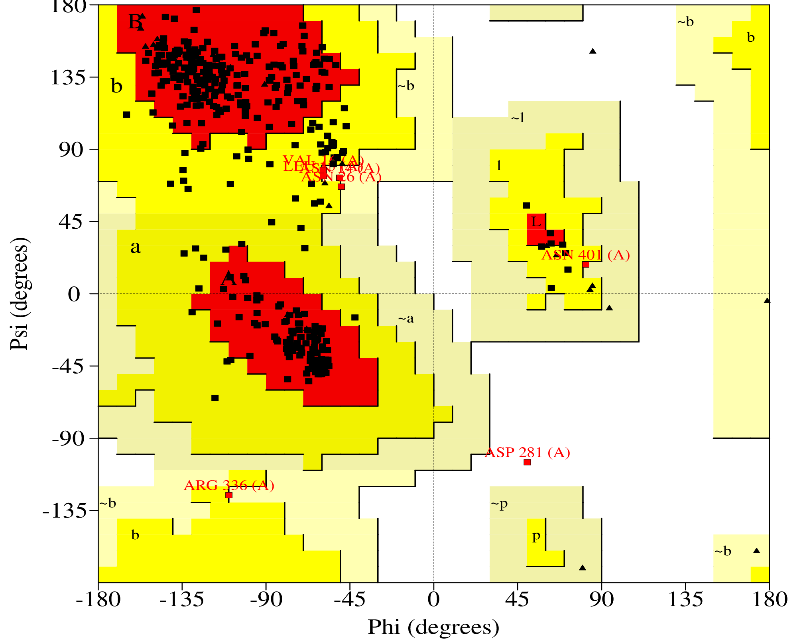


**Ramachandran plot for the model structure of Q9KLK5**

| Residues in most favoured region [A, B, L] | 305 | 82.7% |
| --- | --- | --- |
| Residues in additional allowed region [a, b, l, p] | 57 | 15.4% |
| Residues in generously allowed regions [~a, ~b, ~l, ~p] | 6 | 1.6% |
| Residues in disallowed regions | 1 | 0.3% |
| Total (Number of non-glycine and non-proline residues) | 369 | 100% |
| Number of end-residues (excl. Gly and Pro) | 2 |  |
| Number of Glycine | 35 |  |
| Number of Proline | 7 |  |
| Total number of residues | 413 |  |

**AJ.**

**Ramachandran plot statistics for the model structure of Q9KLK5**


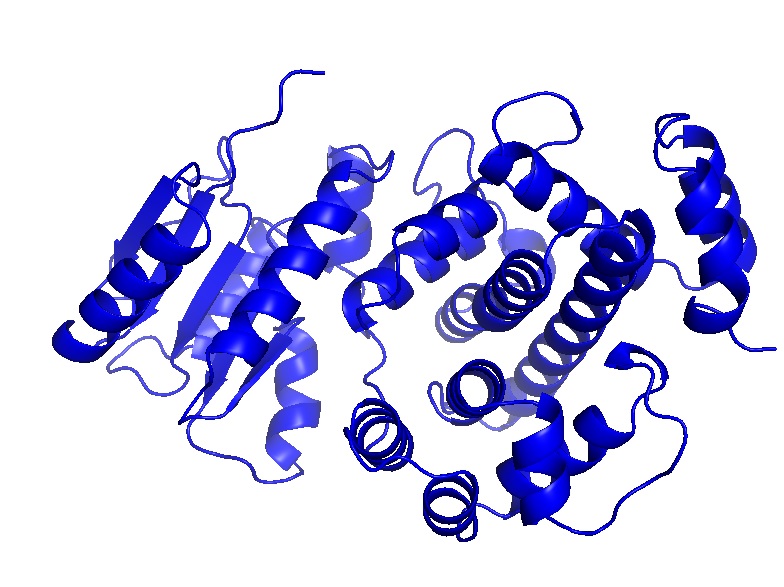


**AK.**

**pLDDT value= 95.2**

**C**

**N**

**Model of the protein (UniProt ID- Q9KT24) derived from AlphaFold-mmseq2**


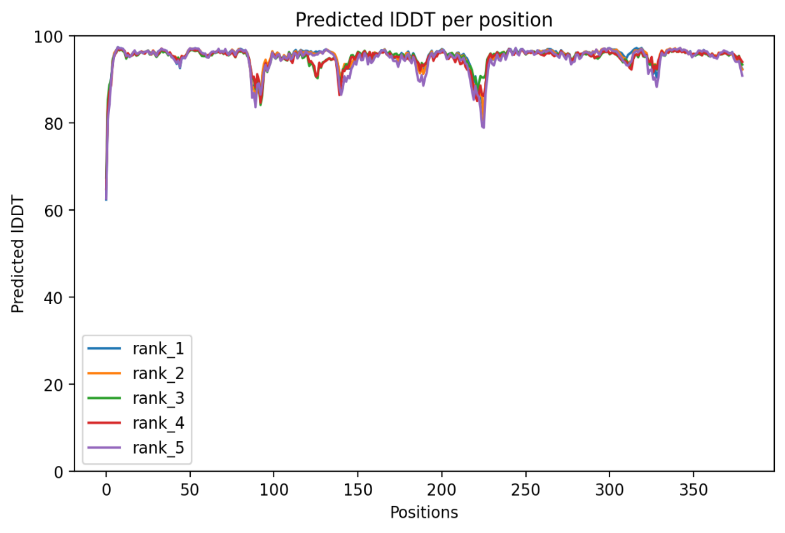
 ***
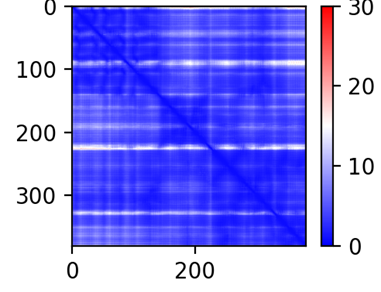
***

**AL.**

**AK.**

**pLDDT graph for the model (UniProt ID-Q9KT24) PAE graph for the model Q9KT24**


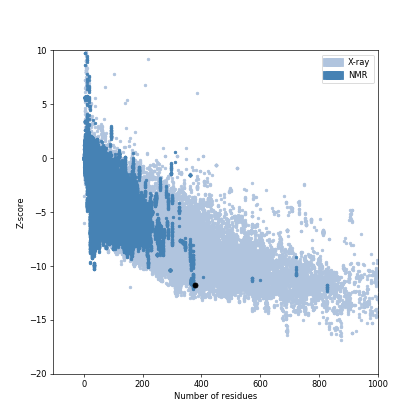


**AM.**

**Z-score= -11.75**

**Overall model quality of the model structure of Q9KT24 by ProSA**

**AN.**


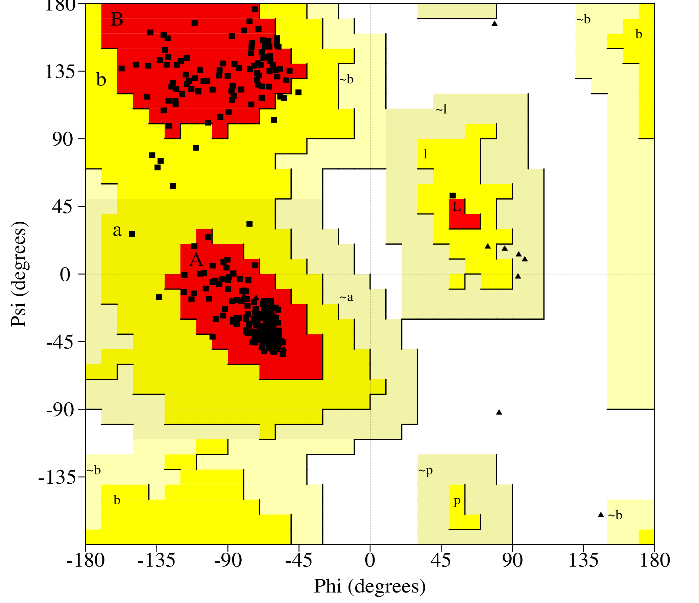


**Ramachandran plot for the model structure of Q9KT24**

| Residues in most favoured region [A, B, L] | 333 | 96.5% |
| --- | --- | --- |
| Residues in additional allowed region [a, b, l, p] | 12 | 3.5% |
| Residues in generously allowed regions [~a, ~b, ~l, ~p] | 0 | 0.0% |
| Residues in disallowed regions | 0 | 0.0% |
| Total (Number of non-glycine and non-proline residues) | 345 | 100% |
| Number of end-residues (excl. Gly and Pro) | 2 |  |
| Number of Glycine | 16 |  |
| Number of Proline | 17 |  |
| Total number of residues | 380 |  |

**AO.**

**Ramachandran plot statistics for the model structure of Q9KT24**

**AP.**


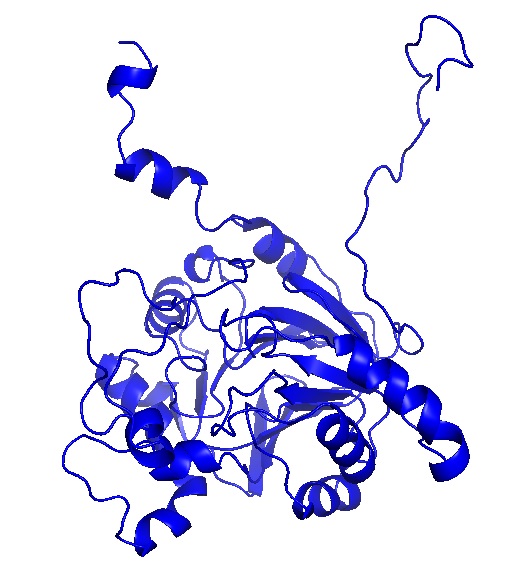


**pLDDT value= 97.9**

**C**

**N**

**Model of the protein (UniProt ID- Q9KMS2) derived from AlphaFold-mmseq2**

**AQ.**


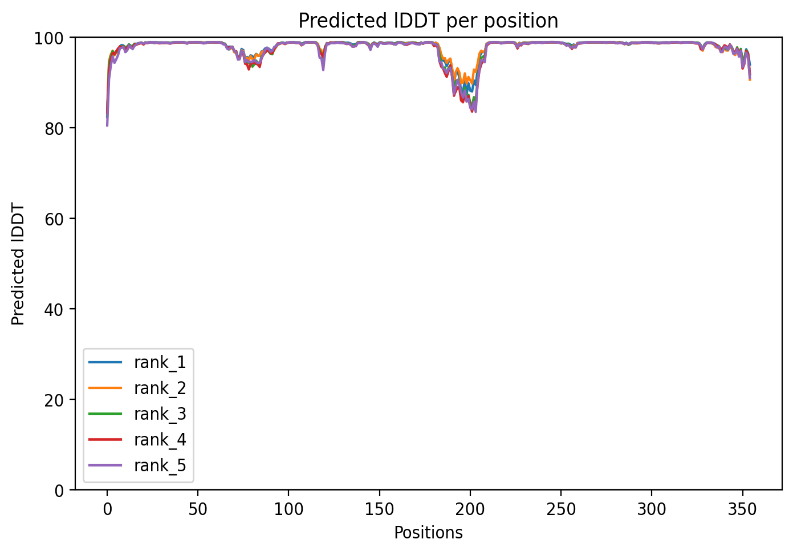
 ***
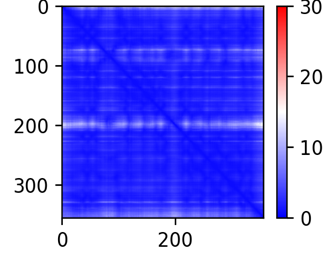
***

**AR.**

**pLDDT graph for the model (UniProt ID-Q9KMS2) PAE graph for the model Q9KMS2**


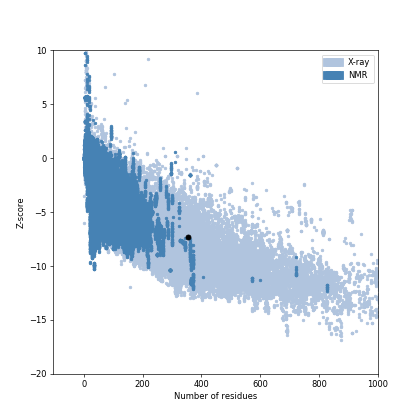


**AR.**

**Z-score= -11.75**

**Overall model quality of the model structure of Q9KMS2 by ProSA**

**AS.**


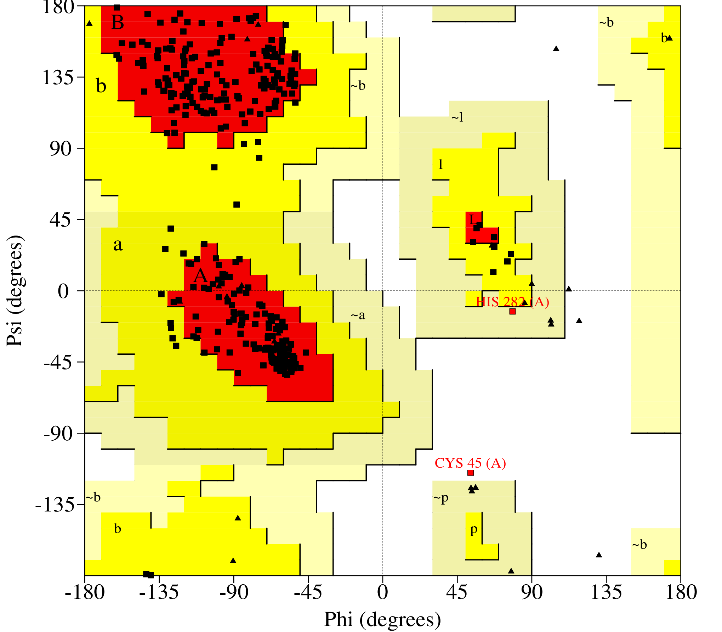


**Ramachandran plot for the model structure of Q9KMS2**

| Residues in most favoured region [A, B, L] | 289 | 92.6% |
| --- | --- | --- |
| Residues in additional allowed region [a, b, l, p] | 21 | 6.7% |
| Residues in generously allowed regions [~a, ~b, ~l, ~p] | 1 | 0.3% |
| Residues in disallowed regions | 1 | 0.3% |
| Total (Number of non-glycine and non-proline residues) | 312 | 100% |
| Number of end-residues (excl. Gly and Pro) | 2 |  |
| Number of Glycine | 26 |  |
| Number of Proline | 15 |  |
| Total number of residues | 355 |  |

**AT.**

**Ramachandran plot statistics for the model structure of Q9KMS2**

**AU.**

**C**


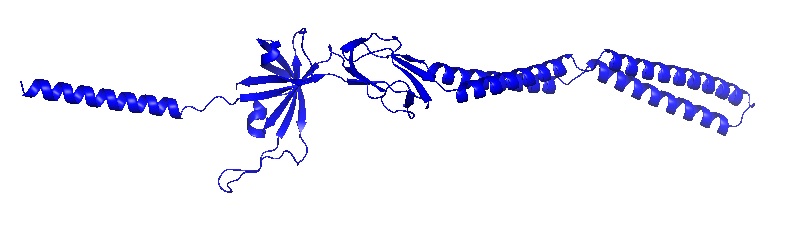


**pLDDT value= 95.6**

**N**

**Model of the protein (UniProt ID- Q9KRM9) derived from AlphaFold-mmseq2**

**AW.**

**AV.**

***
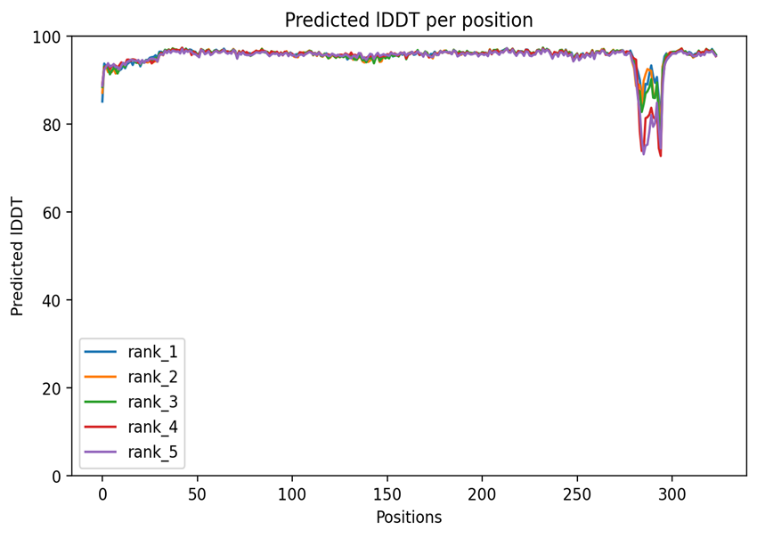
***
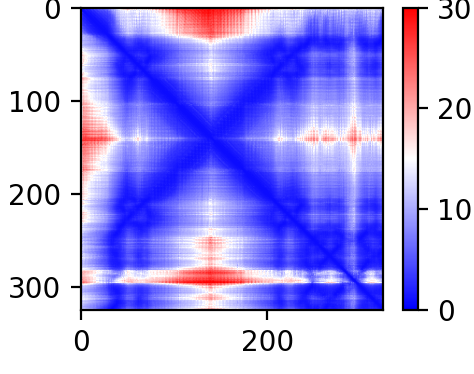


**pLDDT graph for the model (UniProt ID-Q9KRM9) PAE graph for the model Q9KRM9**


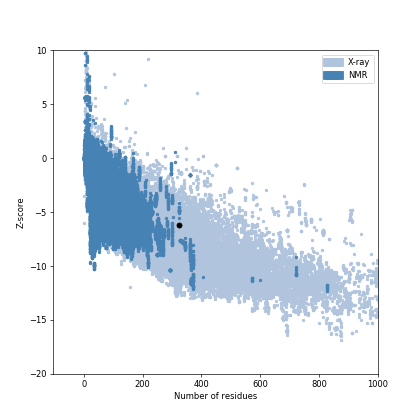


**AX.**

**Z-score= -6.18**

**Overall model quality of the model structure of Q9KRM9 by ProSA**


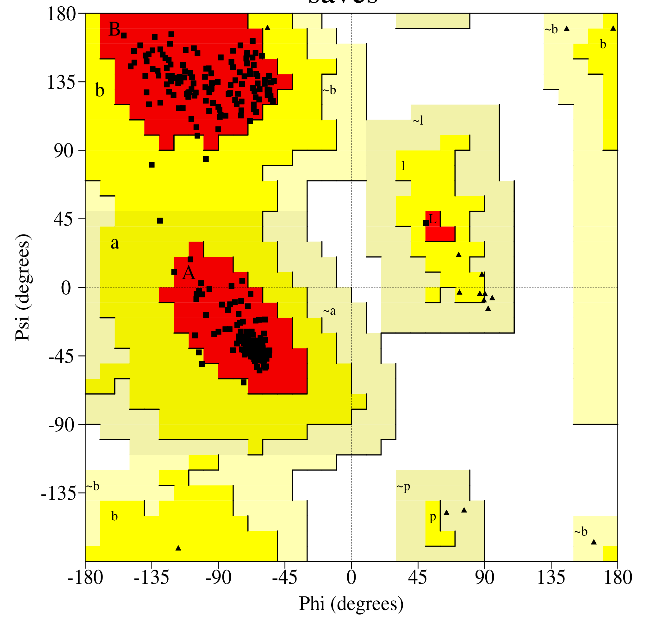


**AY.**

**Ramachandran plot for the model structure of Q9KRM9**

| Residues in most favoured region [A, B, L] | 285 | 97.3% |
| --- | --- | --- |
| Residues in additional allowed region [a, b, l, p] | 8 | 2.7% |
| Residues in generously allowed regions [~a, ~b, ~l, ~p] | 0 | 0.0% |
| Residues in disallowed regions | 0 | 0.0% |
| Total (Number of non-glycine and non-proline residues) | 293 | 100% |
| Number of end-residues (excl. Gly and Pro) | 2 |  |
| Number of Glycine | 20 |  |
| Number of Proline | 9 |  |
| Total number of residues | 324 |  |

**AZ.**

**Ramachandran plot statistics for the model structure of Q9KRM9**


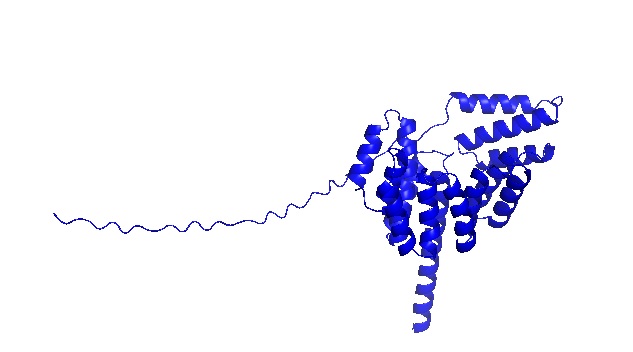


**BA.**

**pLDDT value= 93.8**

**C**

**N**

**Model of the protein (UniProt ID- Q9KU75) derived from AlphaFold-mmseq2**

**BB.**


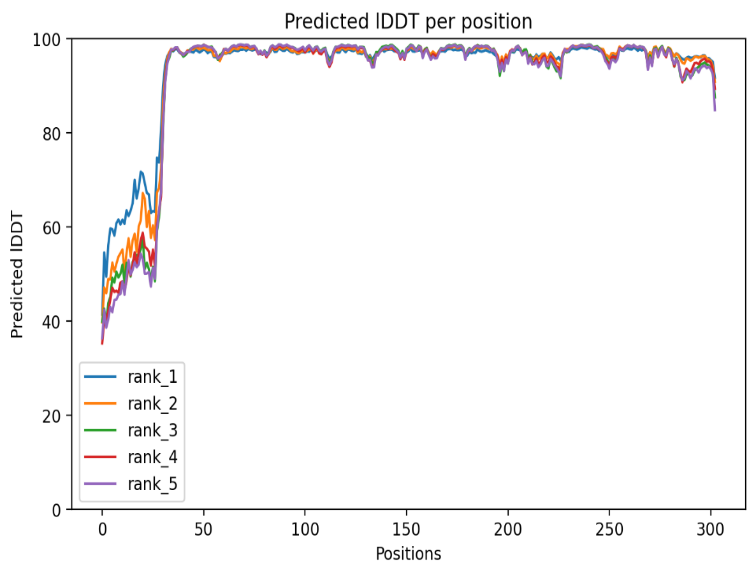
 ***
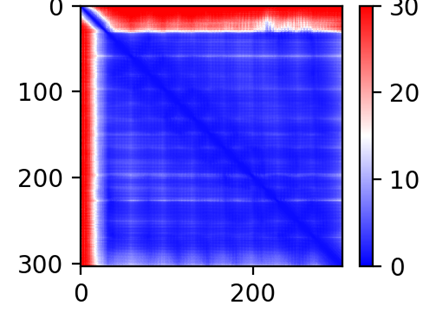
***

**BC.**

**pLDDT graph for the model (UniProt ID-Q9KU75) PAE graph for the model Q9KU75**


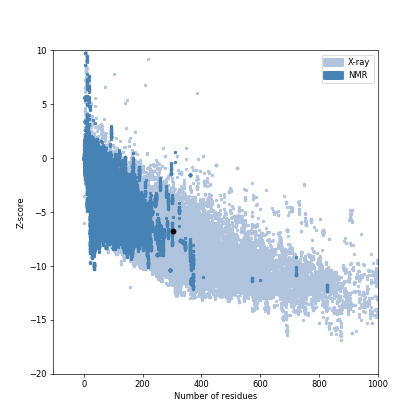


**BD.**

**Z-score= -6.79**

**Overall model quality of the model structure of Q9KU75 by ProSA**


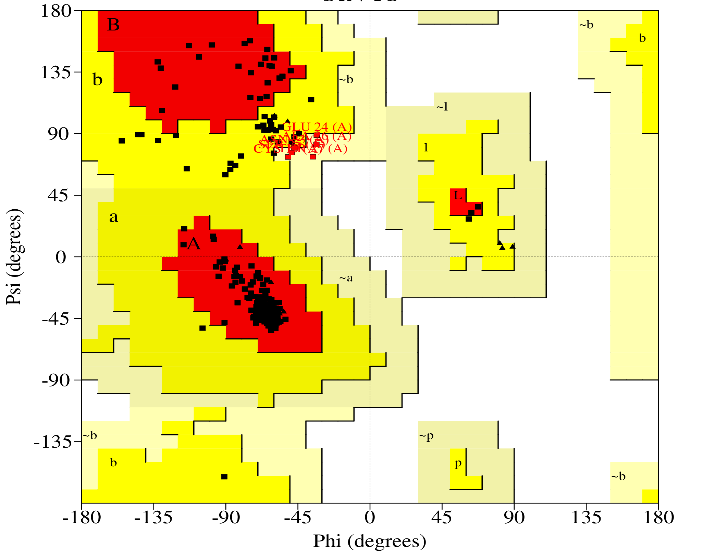


**BE.**

**Ramachandran plot for the model structure of Q9KU75**

| Residues in most favoured region [A, B, L] | 236 | 85.5% |
| --- | --- | --- |
| Residues in additional allowed region [a, b, l, p] | 34 | 12.3% |
| Residues in generously allowed regions [~a, ~b, ~l, ~p] | 6 | 2.2% |
| Residues in disallowed regions | 0 | 0.0% |
| Total (Number of non-glycine and non-proline residues) | 276 | 100% |
| Number of end-residues (excl. Gly and Pro) | 2 |  |
| Number of Glycine | 13 |  |
| Number of Proline | 12 |  |
| Total number of residues | 303 |  |

**BF.**

**Ramachandran plot statistics for the model structure of Q9KU75**


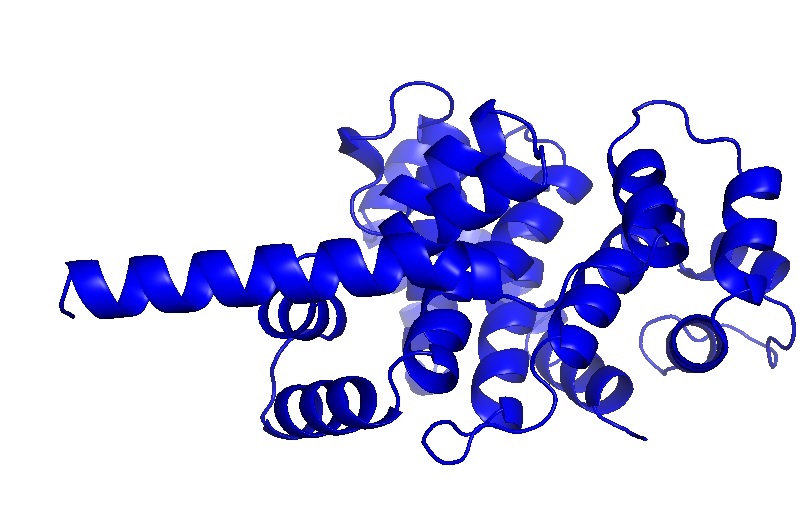


**BG.**

**C**

**N**

**pLDDT value= 94.2**

**Model of the protein (UniProt ID- Q9KND1) derived from AlphaFold-mmseq2**


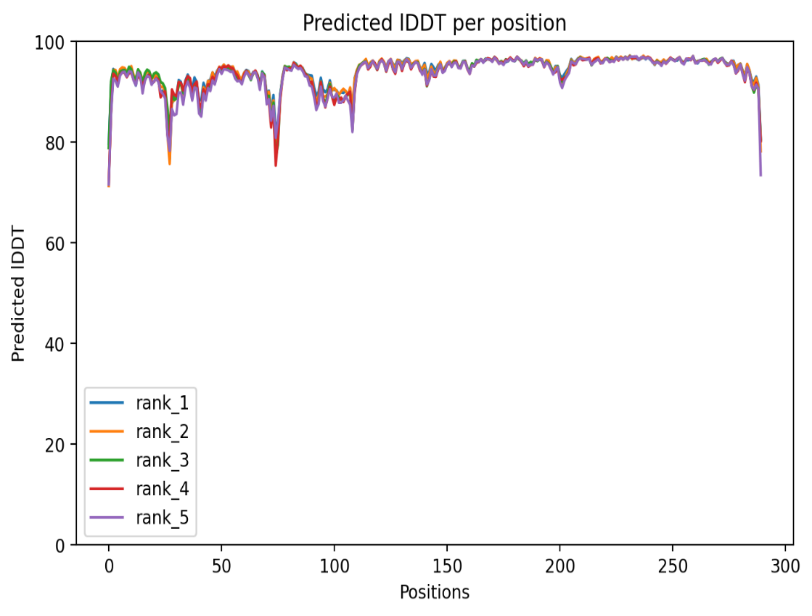
 ***
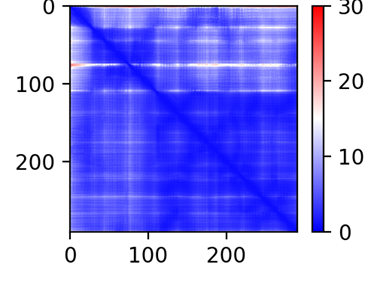
***

**BI.**

**BH.**

**pLDDT graph for the model (UniProt ID-Q9KND1) PAE graph for the model Q9KND1**


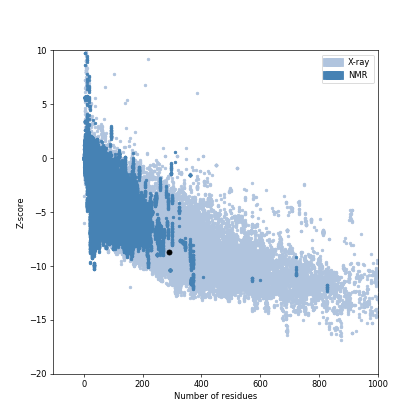


**BJ.**

**Z-score= -8.72**

**Overall model quality of the model structure of Q9KND1 by ProSA**

**BK.**


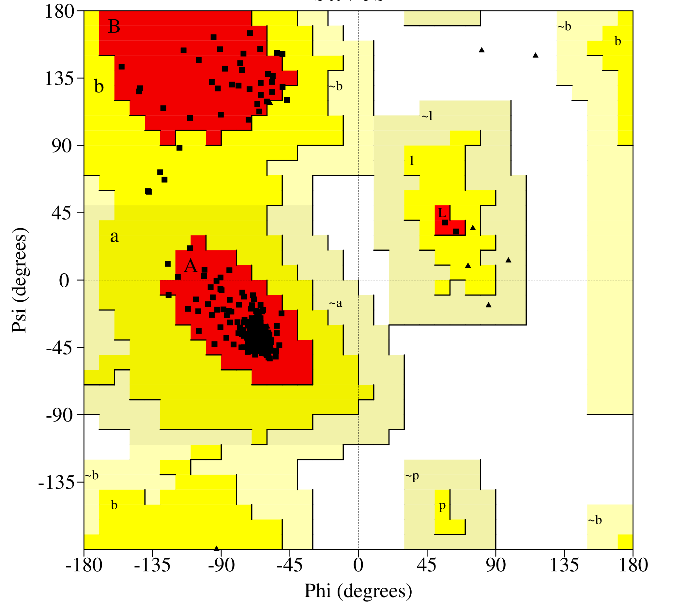


**Ramachandran plot for the model structure of Q9KND1**

**BL.**

| Residues in most favoured region [A, B, L] | 256 | 96.6% |
| --- | --- | --- |
| Residues in additional allowed region [a, b, l, p] | 9 | 3.4% |
| Residues in generously allowed regions [~a, ~b, ~l, ~p] | 0 | 0.0% |
| Residues in disallowed regions | 0 | 0.0% |
| Total (Number of non-glycine and non-proline residues) | 265 | 100% |
| Number of end-residues (excl. Gly and Pro) | 2 |  |
| Number of Glycine | 12 |  |
| Number of Proline | 11 |  |
| Total number of residues | 290 |  |

**Ramachandran plot statistics for the model structure of Q9KND1**

**BM.**


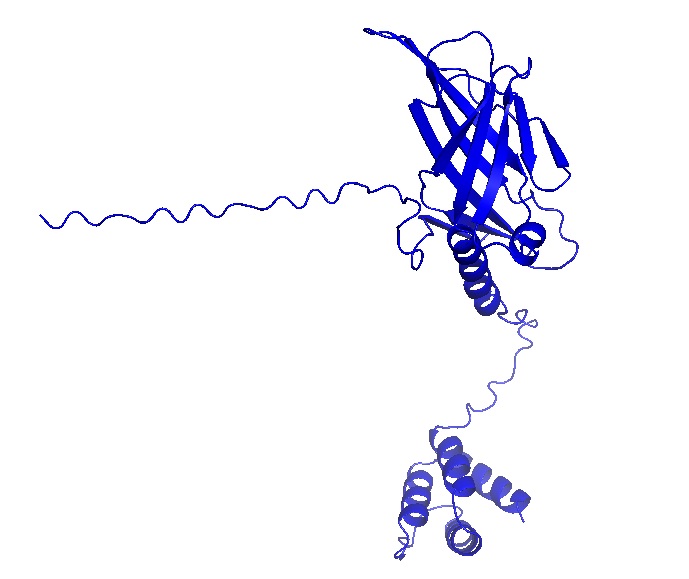


**C**

**pLDDT value= 84.8**

**N**

**Model of the protein (UniProt ID- Q9KTC9) derived from AlphaFold-mmseq2**


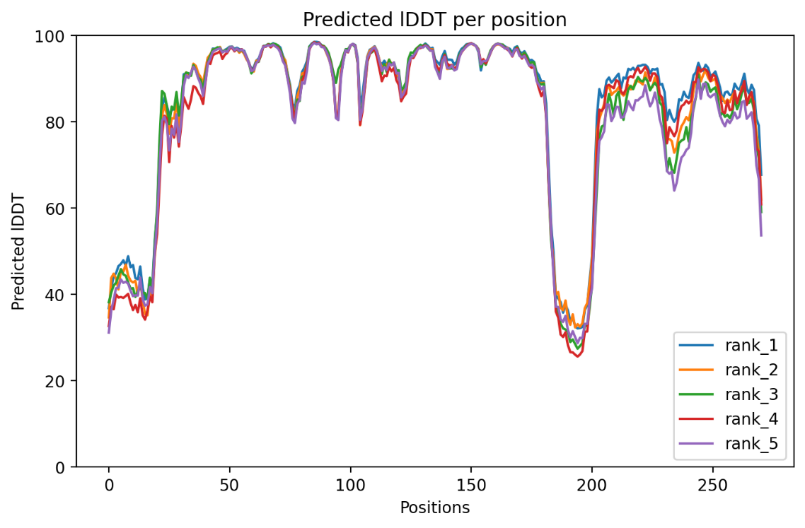
 **
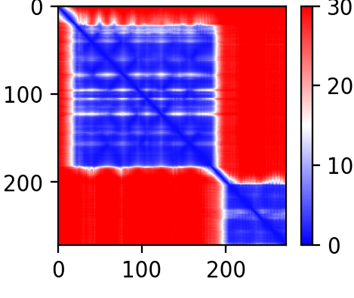
**

**BO.**

**BN.**

**pLDDT graph for the model (UniProt ID-Q9KTC9) PAE graph for the model Q9KTC9**


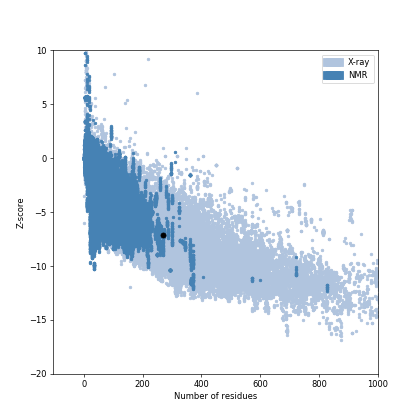


**BP.**

**Z-score= -7.17**

**Overall model quality of the model structure of Q9KTC9 by ProSA**

**BQ.**


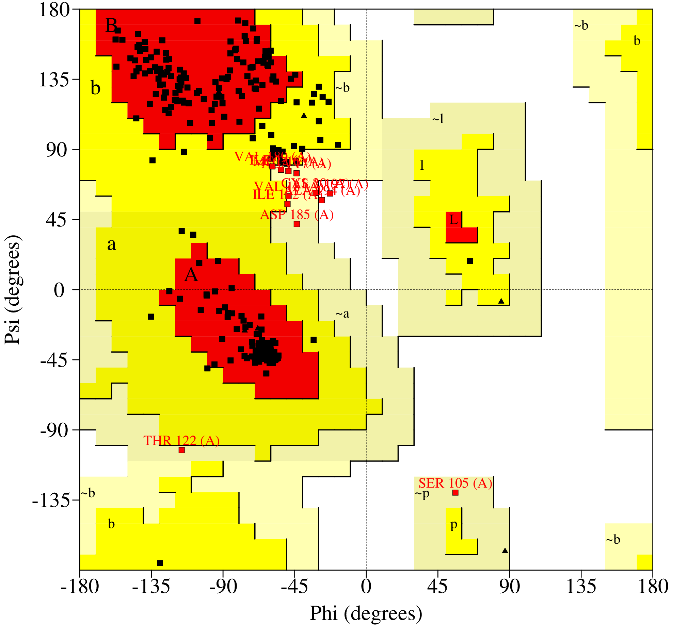


**Ramachandran plot for the model structure of Q9KTC9**

**BR.**

| Residues in most favoured region [A, B, L] | 206 | 81.7% |
| --- | --- | --- |
| Residues in additional allowed region [a, b, l, p] | 34 | 13.5% |
| Residues in generously allowed regions [~a, ~b, ~l, ~p] | 10 | 4.0% |
| Residues in disallowed regions | 2 | 0.8% |
| Total (Number of non-glycine and non-proline residues) | 252 | 199% |
| Number of end-residues (excl. Gly and Pro) | 2 |  |
| Number of Glycine | 6 |  |
| Number of Proline | 11 |  |
| Total number of residues | 271 |  |

**Ramachandran plot statistics for the model structure of Q9KTC9**

**BS.**


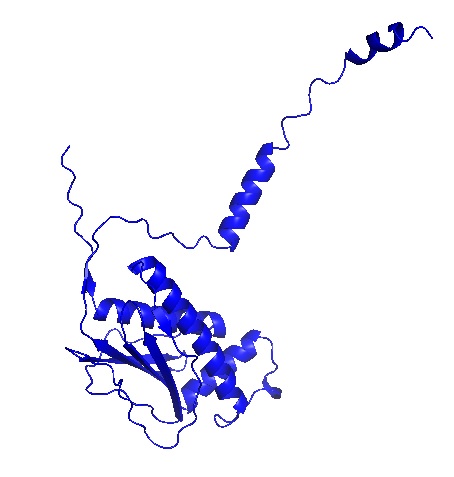


**N**

**C**

**pLDDT value= 84.6**

**Model of the protein (UniProt ID- Q9KSQ9) derived from AlphaFold-mmseq2**


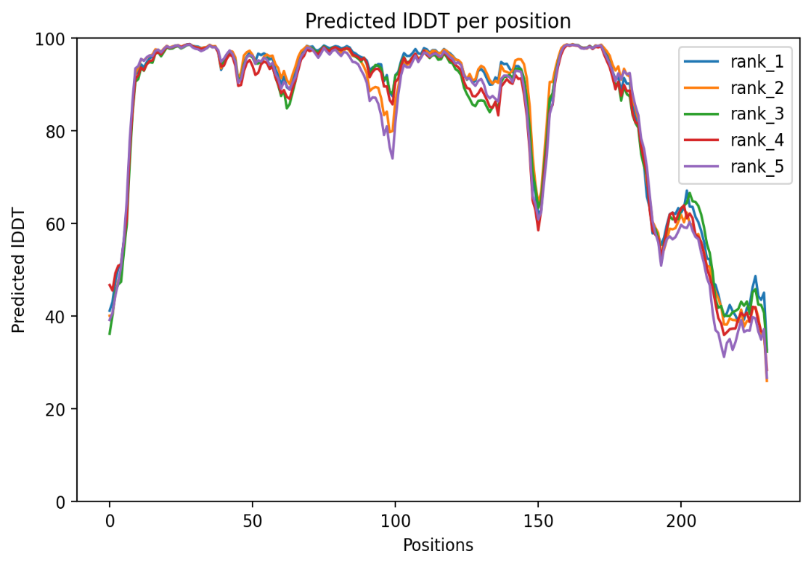
 **
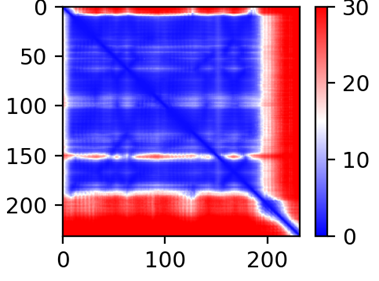
**

**BU.**

**BT.**

**pLDDT graph for the model (UniProt ID-Q9KSQ9) PAE graph for the model Q9KSQ9**


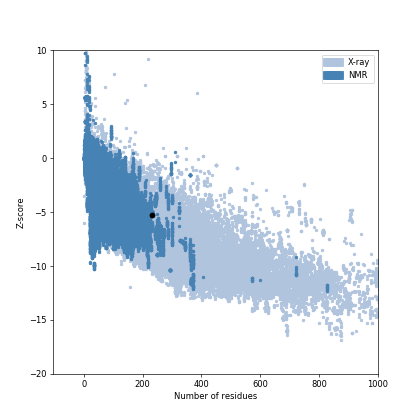


**Z-score= -5.23**

**BV.**

**Overall model quality of the model structure of Q9KSQ9 by ProSA**

**BW.**


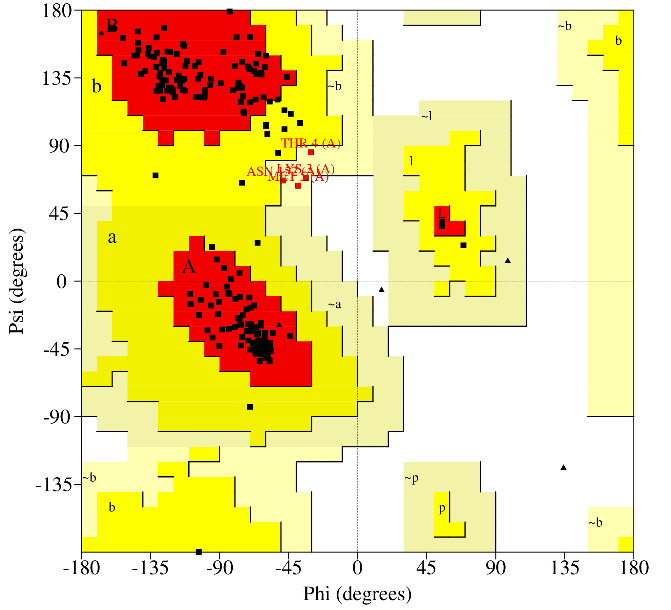


**Ramachandran plot for the model structure of Q9KSQ9**

| Residues in most favoured region [A, B, L] | 194 | 90.7% |
| --- | --- | --- |
| Residues in additional allowed region [a, b, l, p] | 16 | 7.5% |
| Residues in generously allowed regions [~a, ~b, ~l, ~p] | 4 | 1.9% |
| Residues in disallowed regions | 0 | 0.0% |
| Total (Number of non-glycine and non-proline residues) | 214 | 100% |
| Number of end-residues (excl. Gly and Pro) | 2 |  |
| Number of Glycine | 9 |  |
| Number of Proline | 6 |  |
| Total number of residues | 231 |  |

**BX.**

**Ramachandran plot statistics for the model structure of Q9KSQ9**


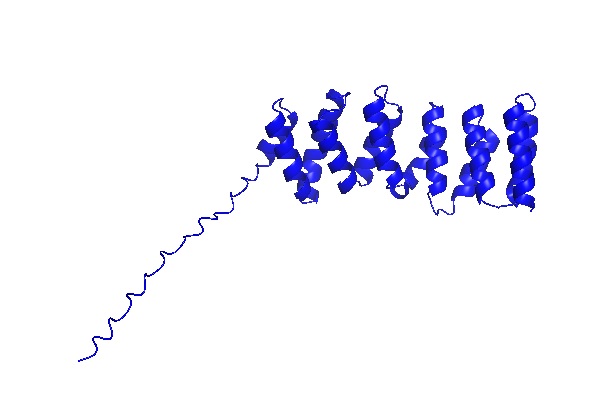


**BY.**

**N**

**C**

**pLDDT value= 88.9**

**Model of the protein (UniProt ID- Q9KS60) derived from AlphaFold-mmseq2**

**BZ.**


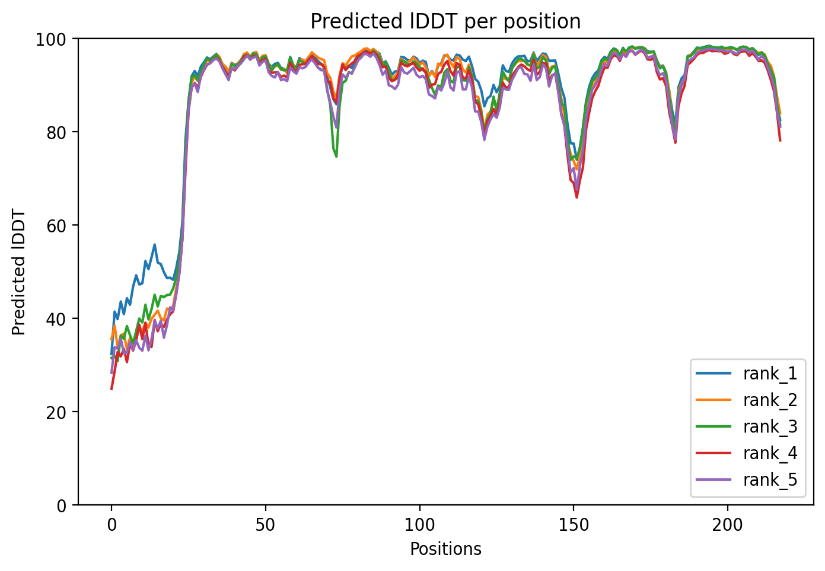

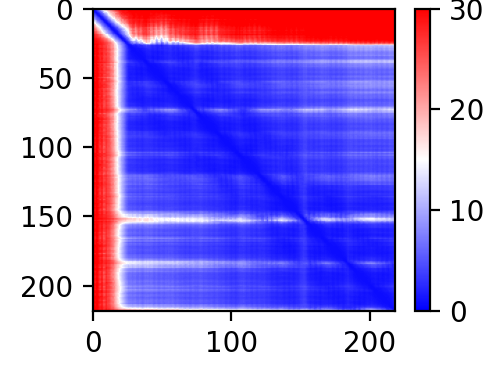


**CA.**

**pLDDT graph for the model (UniProt ID-Q9KS60) PAE graph for the model Q9KS60**


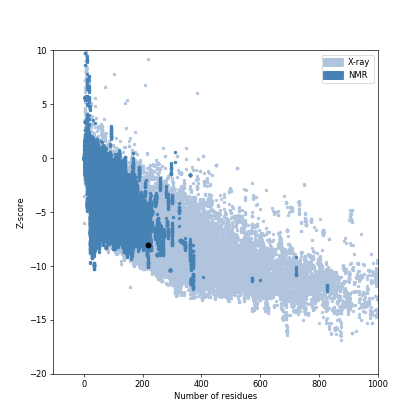


**CB.**

**Z-score= -8.02**

**Overall model quality of the model structure of Q9KS60 by ProSA**

**CC.**


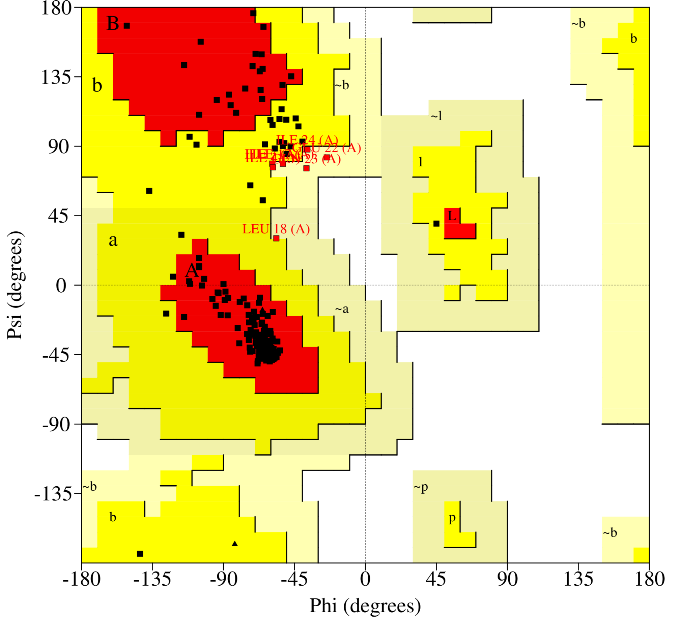


**Ramachandran plot for the model structure of Q9KS60**

| Residues in most favoured region [A, B, L] | 171 | 85.4% |
| --- | --- | --- |
| Residues in additional allowed region [a, b, l, p] | 23 | 11.4% |
| Residues in generously allowed regions [~a, ~b, ~l, ~p] | 7 | 3.5% |
| Residues in disallowed regions | 0 | 0.0% |
| Total (Number of non-glycine and non-proline residues) | 201 |  |
| Number of end-residues (excl. Gly and Pro) | 2 |  |
| Number of Glycine | 4 |  |
| Number of Proline | 11 |  |
| Total number of residues | 218 |  |

**CD.**

**Ramachandran plot statistics for the model structure of Q9KSQ9**

**CE.**

**C**


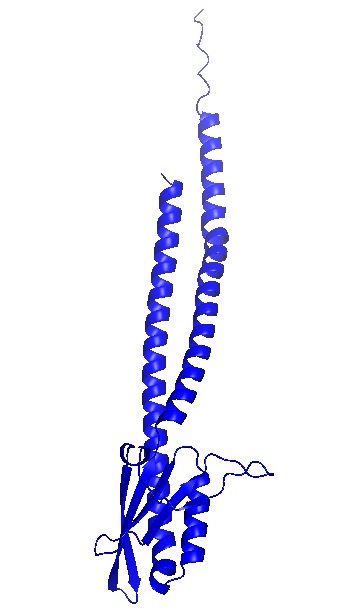


**N**

**pLDDT value= 91.1**

**Model of the protein (UniProt ID- Q9KKX0) derived from AlphaFold-mmseq2**

**CF.**


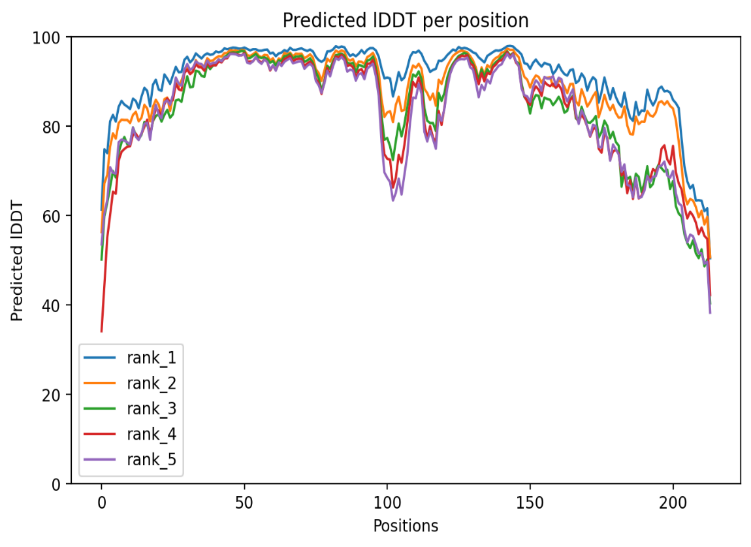
 **
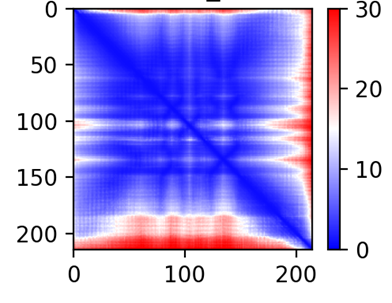
**

**CG.**

**pLDDT graph for the model (UniProt ID-Q9KKX0) PAE graph for the model Q9KKX0**


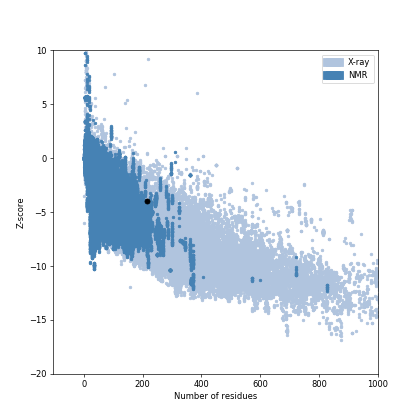


**CH.**

**Z-score= -3.94**

**Overall model quality of the model structure of Q9KKX0 by ProSA**


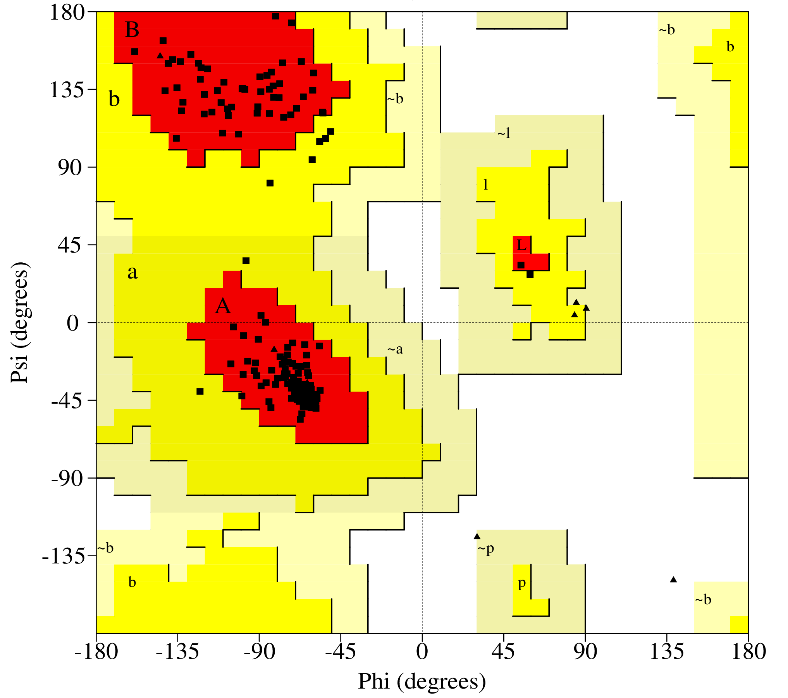


**CI.**

**Ramachandran plot for the model structure of Q9KKX0**

| Residues in most favoured region [A, B, L] | 188 | 95.9% |
| --- | --- | --- |
| Residues in additional allowed region [a, b, l, p] | 8 | 4.1% |
| Residues in generously allowed regions [~a, ~b, ~l, ~p] | 0 | 0.0% |
| Residues in disallowed regions | 0 | 0.0% |
| Total (Number of non-glycine and non-proline residues) | 196 | 100% |
| Number of end-residues (excl. Gly and Pro) | 2 |  |
| Number of Glycine | 8 |  |
| Number of Proline | 8 |  |
| Total number of residues | 214 |  |

**CJ.**

**Ramachandran plot statistics for the model structure of Q9KKX0**


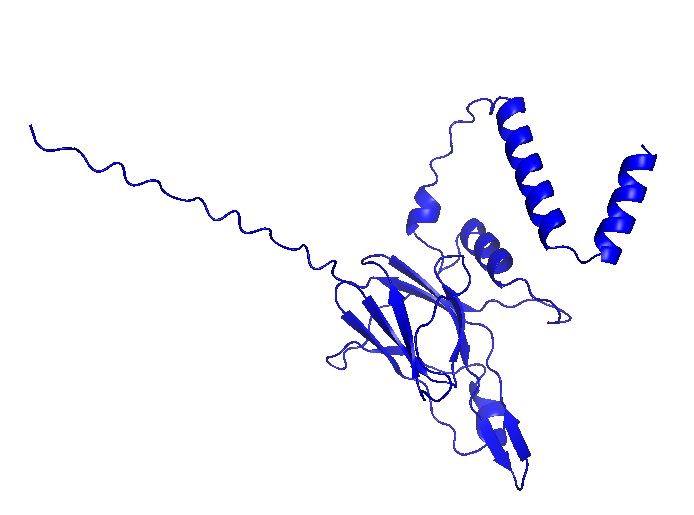


**CK.**

**N**

**C**

**pLDDT value= 89.1**

**Model of the protein (UniProt ID- Q9KND9) derived from AlphaFold-mmseq2**


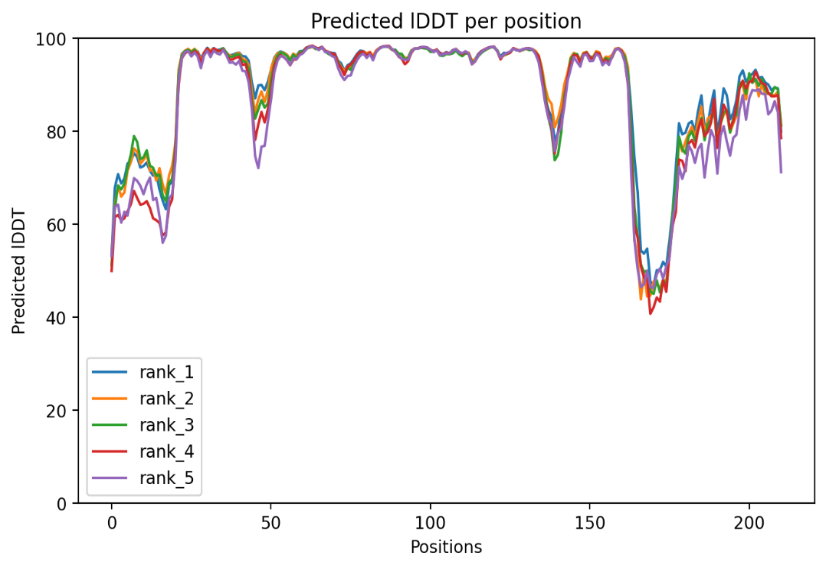
 **
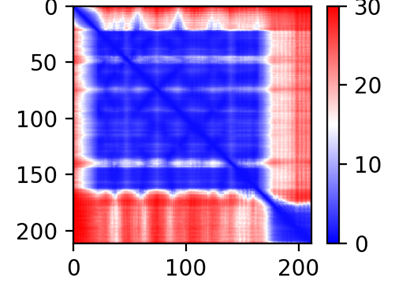
**

**CM.**

**CL.**

**pLDDT graph for the model (UniProt ID-Q9KND9) PAE graph for the model Q9KND9**


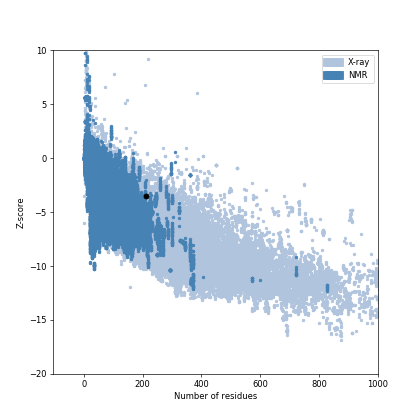


**CN.**

**Z-score= -3.52**

**Overall model quality of the model structure of Q9KND9 by ProSA**

**CO.**


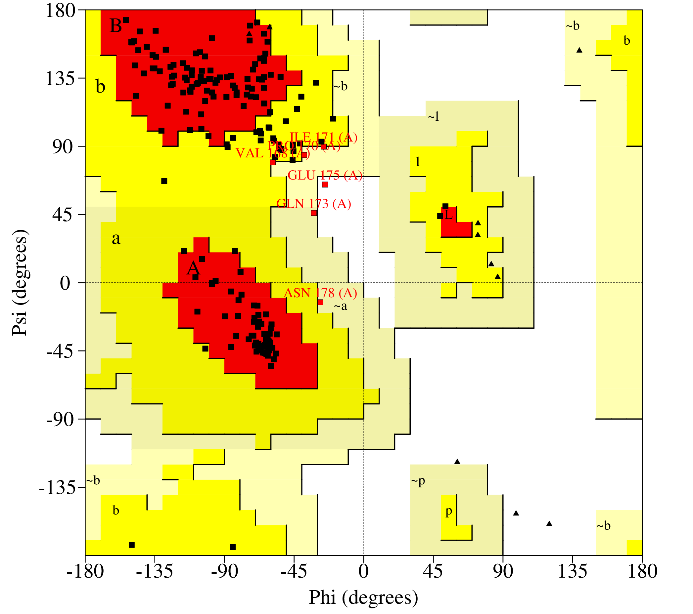


**Ramachandran plot for the model structure of Q9KND9**

| Residues in most favoured region [A, B, L] | 151 | 78.6% |
| --- | --- | --- |
| Residues in additional allowed region [a, b, l, p] | 36 | 18.8% |
| Residues in generously allowed regions [~a, ~b, ~l, ~p] | 4 | 2.1% |
| Residues in disallowed regions | 1 | 0.5% |
| Total (Number of non-glycine and non-proline residues) | 192 | 100% |
| Number of end-residues (excl. Gly and Pro) | 2 |  |
| Number of Glycine | 10 |  |
| Number of Proline | 7 |  |
| Total number of residues | 211 |  |

**CP.**

**Ramachandran plot statistics for the model structure of Q9KKX0**

**C**


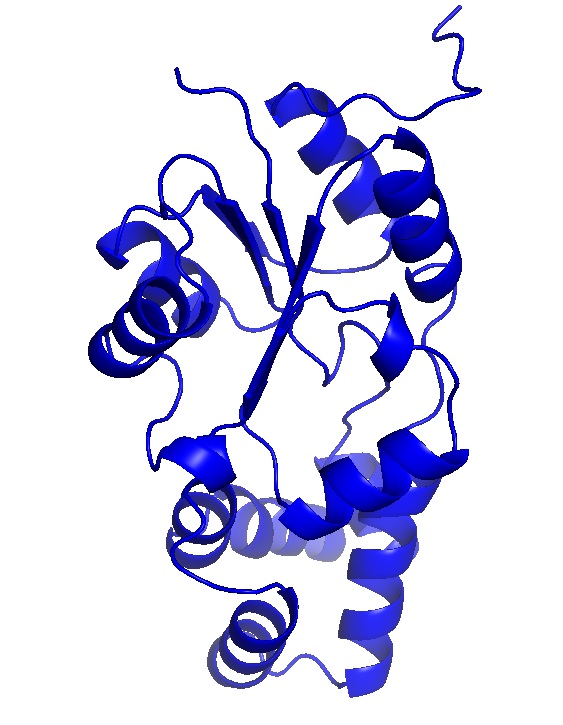


**CQ.**

**pLDDT value= 96.1**

**N**

**Model of the protein (UniProt ID- Q9KRJ5) derived from AlphaFold-mmseq2**

**CR.**


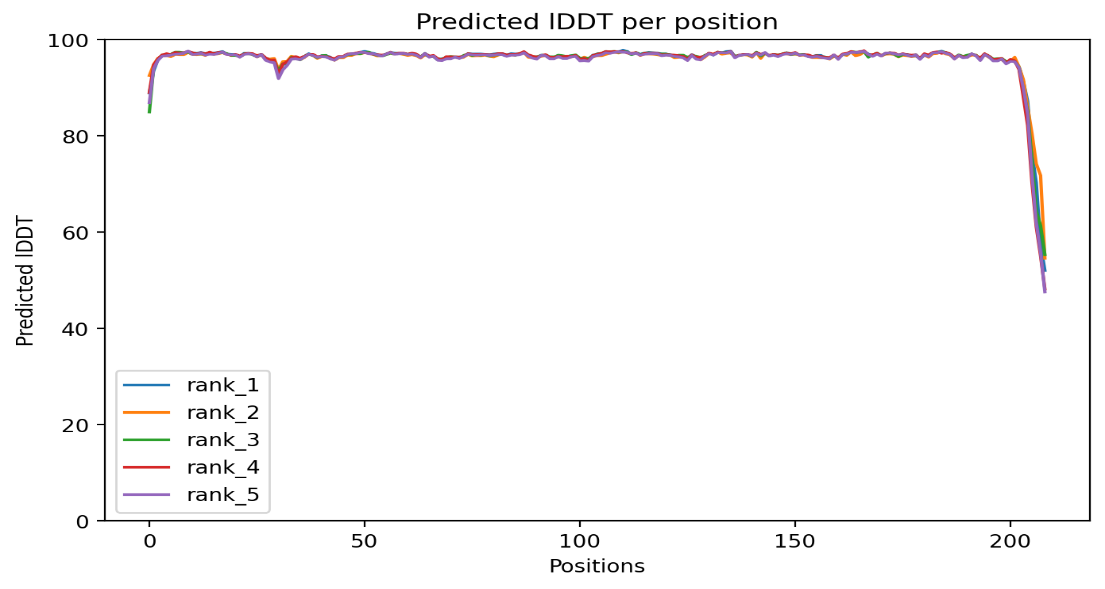
 **
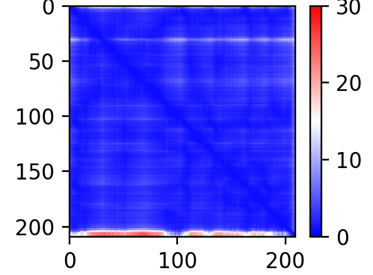
**

**CS.**

**pLDDT graph for the model (UniProt ID-Q9KRJ5) PAE graph for the model Q9K****RJ5**


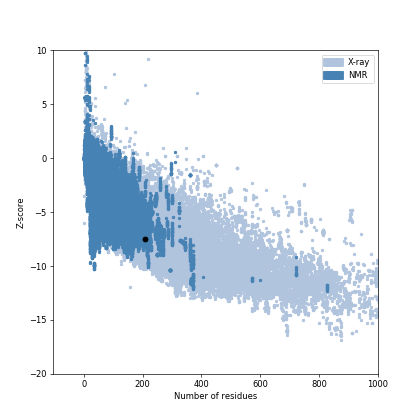


**CT.**

**Z-score= -7.51**

**Overall model quality of the model structure of Q9KRJ5 by ProSA**

**CU.**


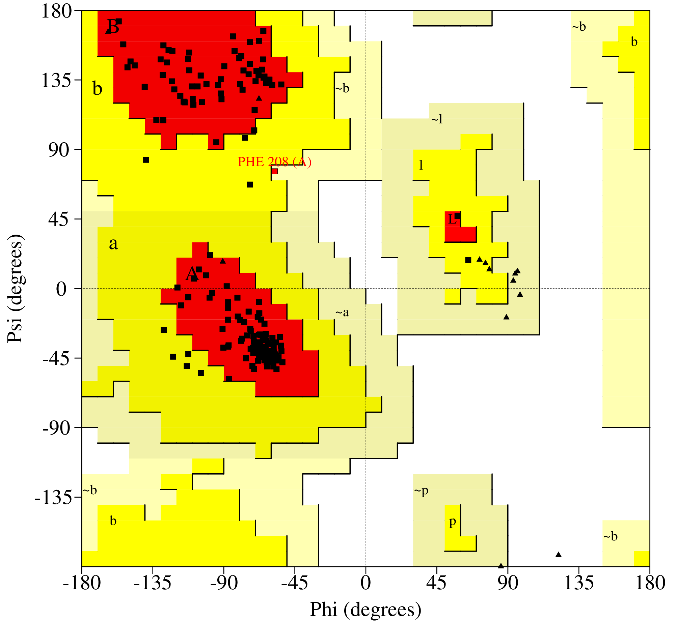


**Ramachandran plot for the model structure of Q9KRJ5**

| Residues in most favoured region [A, B, L] | 176 | 94.6% |
| --- | --- | --- |
| Residues in additional allowed region [a, b, l, p] | 9 | 4.8% |
| Residues in generously allowed regions [~a, ~b, ~l, ~p] | 1 | 0.5% |
| Residues in disallowed regions | 0 | 0.0% |
| Total (Number of non-glycine and non-proline residues) | 186 | 100% |
| Number of end-residues (excl. Gly and Pro) | 1 |  |
| Number of Glycine | 15 |  |
| Number of Proline | 7 |  |
| Total number of residues | 209 |  |

**CV.**

**Ramachandran plot statistics for the model structure of Q9KRJ5**

**C**


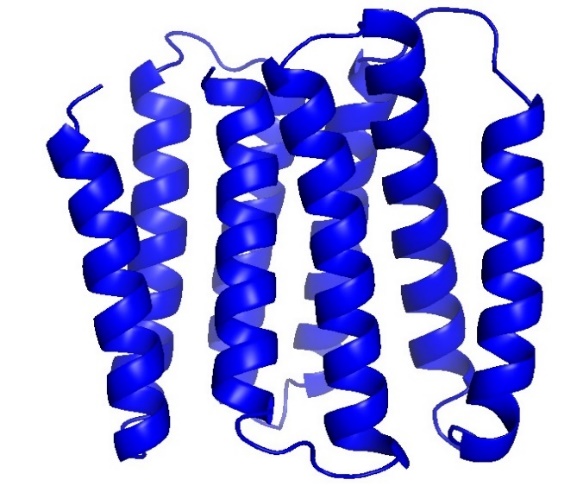


**CW.**

**pLDDT value= 94.9**

**N**

**Model of the protein (UniProt ID- Q9KVJ9) derived from AlphaFold-mmseq2**

**CZ.**


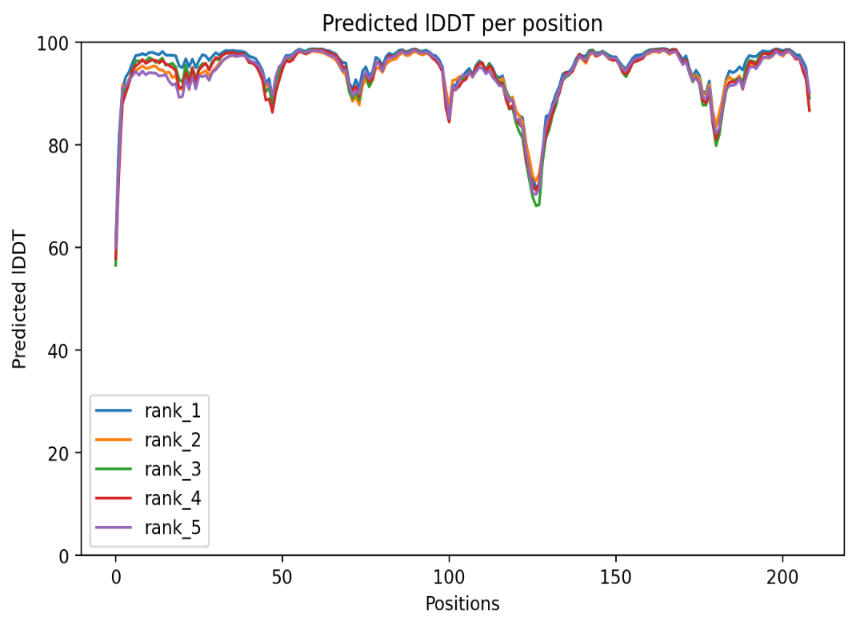
***
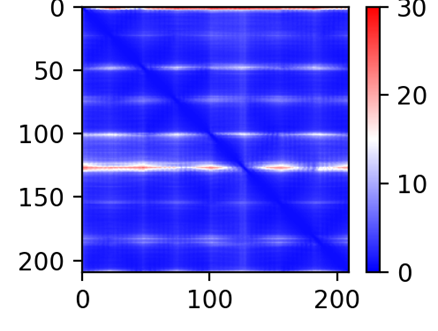
***

**DA.**

**pLDDT graph for the model (UniProt ID-Q9KVJ9) PAE graph for the model Q9KVJ9**


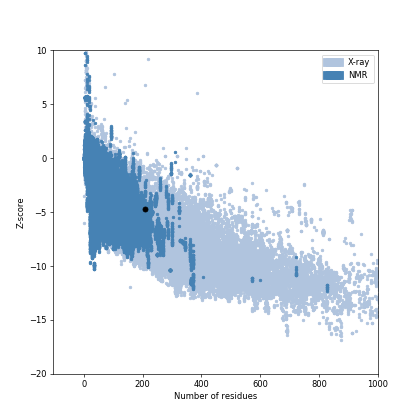


**DB.**

**Z-score= -4.7**

**Overall model quality of the model structure of Q9KVJ9 by ProSA**


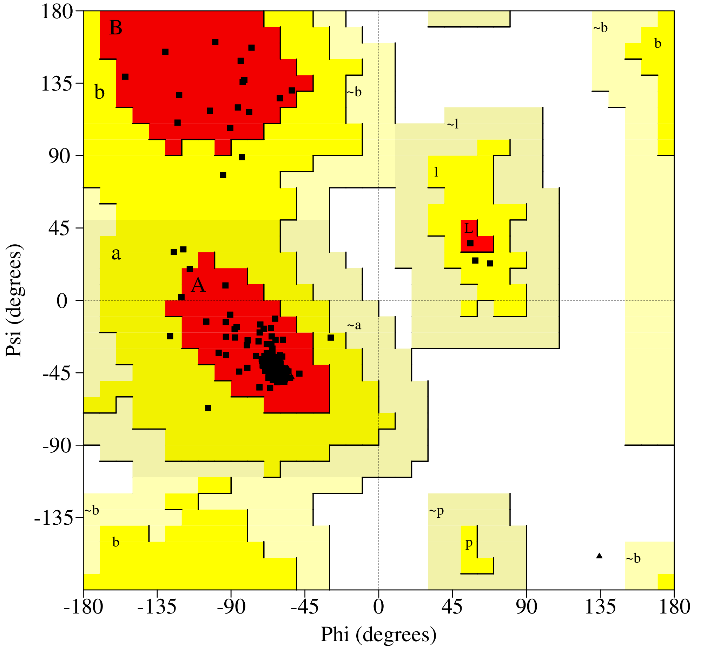


**DC.**

**Ramachandran plot for the model structure of Q9KVJ9**

| Residues in most favoured region [A, B, L] | 181 | 94.8% |
| --- | --- | --- |
| Residues in additional allowed region [a, b, l, p] | 10 | 5.2% |
| Residues in generously allowed regions [~a, ~b, ~l, ~p] | 0 | 0.0% |
| Residues in disallowed regions | 0 | 0.0% |
| Total (Number of non-glycine and non-proline residues) | 191 | 100% |
| Number of end-residues (excl. Gly and Pro) | 2 |  |
| Number of Glycine | 10 |  |
| Number of Proline | 6 |  |
| Total number of residues | 209 |  |

**DD.**

**Ramachandran plot statistics for the model structure of Q9KVJ9**


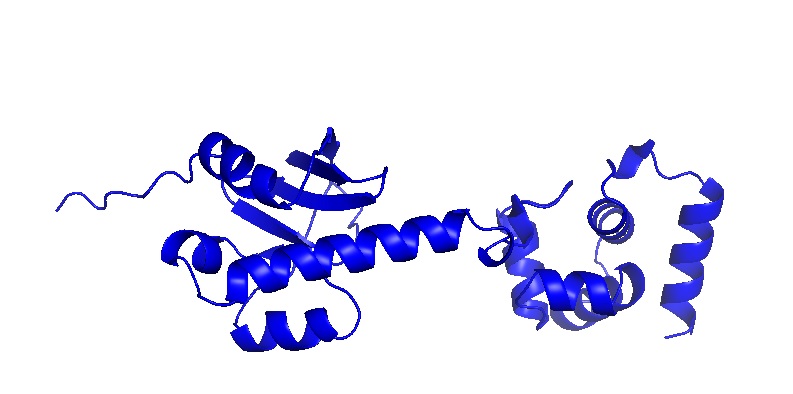


**DE.**

**pLDDT value= 91.4**

**C**

**N**

**Model of the protein (UniProt ID- Q9KSV3) derived from AlphaFold-mmseq2**


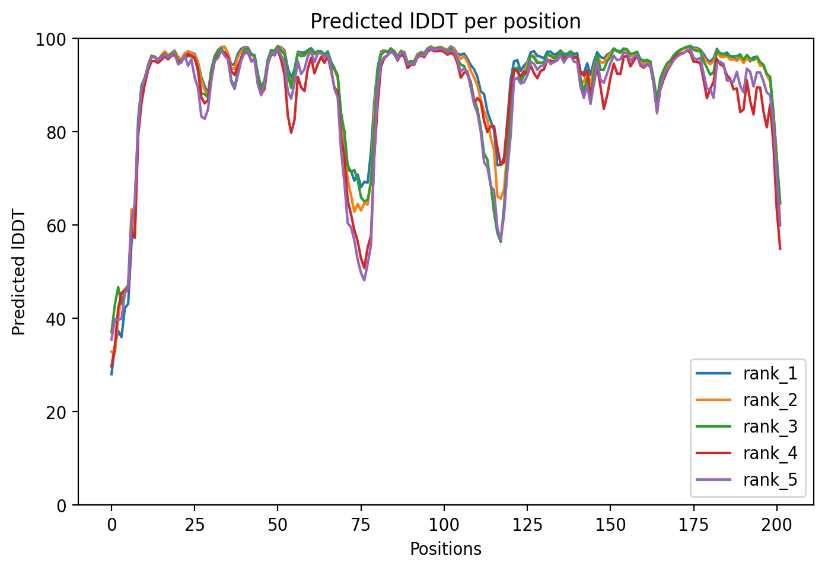

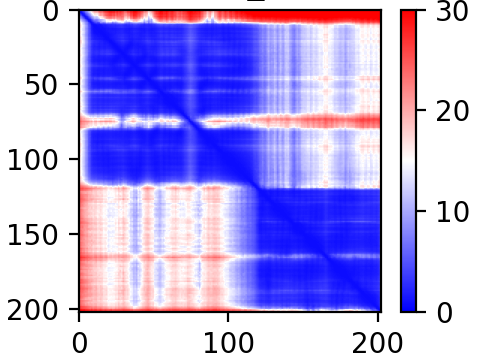


**DF.**

**DF.**

**pLDDT graph for the model (UniProt ID-Q9KSV3) PAE graph for the model Q9KSV3**


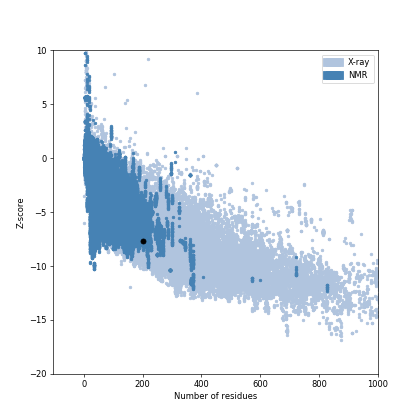


**DG.**

**Z-score= -7.71**

**Overall model quality of the model structure of Q9KSV3 by ProSA**


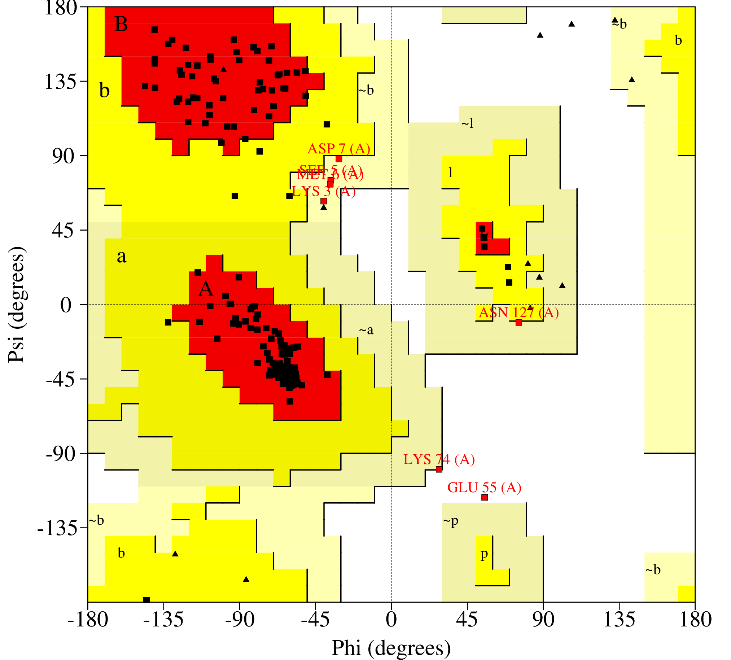


**DH.**

**Ramachandran plot for the model structure of Q9KSV3**

| Residues in most favoured region [A, B, L] | 166 | 91.2% |
| --- | --- | --- |
| Residues in additional allowed region [a, b, l, p] | 9 | 4.9% |
| Residues in generously allowed regions [~a, ~b, ~l, ~p] | 6 | 3.3% |
| Residues in disallowed regions | 1 | 0.5% |
| Total (Number of non-glycine and non-proline residues) | 182 | 100% |
| Number of end-residues (excl. Gly and Pro) | 2 |  |
| Number of Glycine | 13 |  |
| Number of Proline | 5 |  |
| Total number of residues | 202 |  |

**DI.**

**Ramachandran plot statistics for the model structure of Q9KSV3**


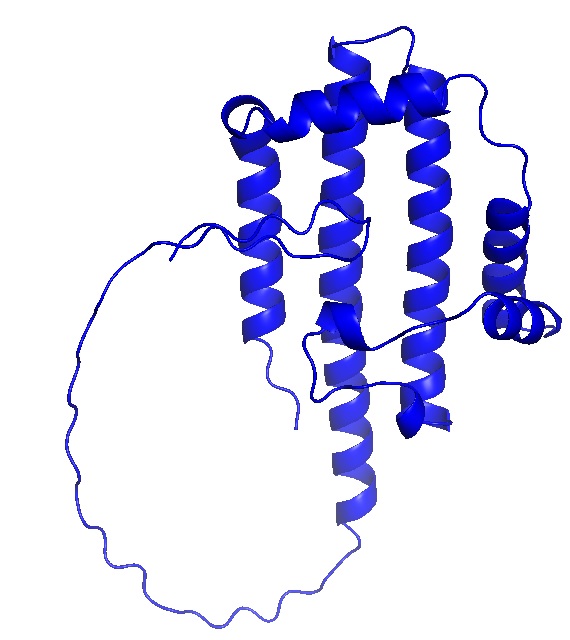


**DJ.**

**pLDDT value= 84.1**

**N**

**C**

**Model of the protein (UniProt ID- Q9KSV6) derived from AlphaFold-mmseq2**

**DK.**


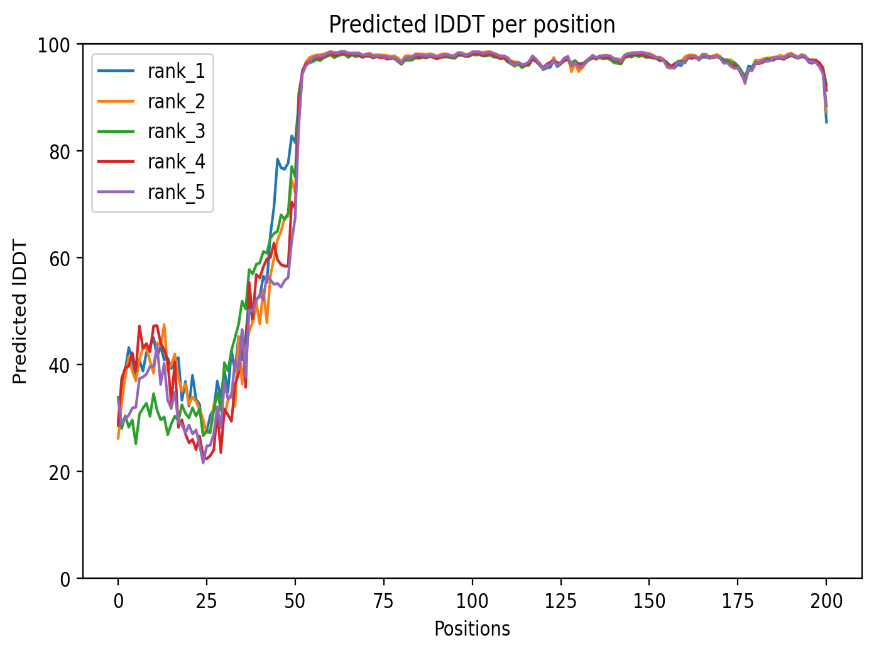

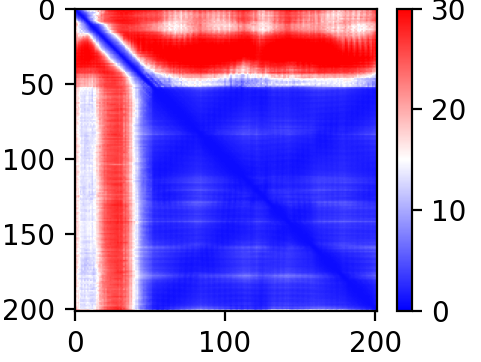


**DL.**

**pLDDT graph for the model (UniProt ID-Q9KSV6) PAE graph for the model Q9KSV6**


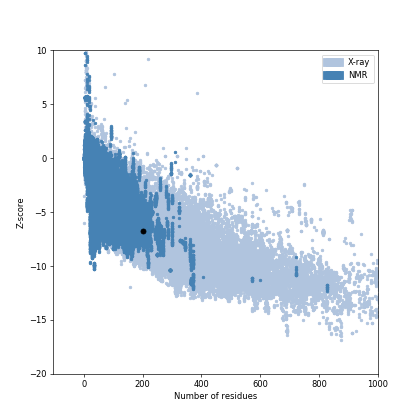


**DM.**

**Z-score= -6.76**

**Overall model quality of the model structure of Q9KSV6 by ProSA**

**DN.**


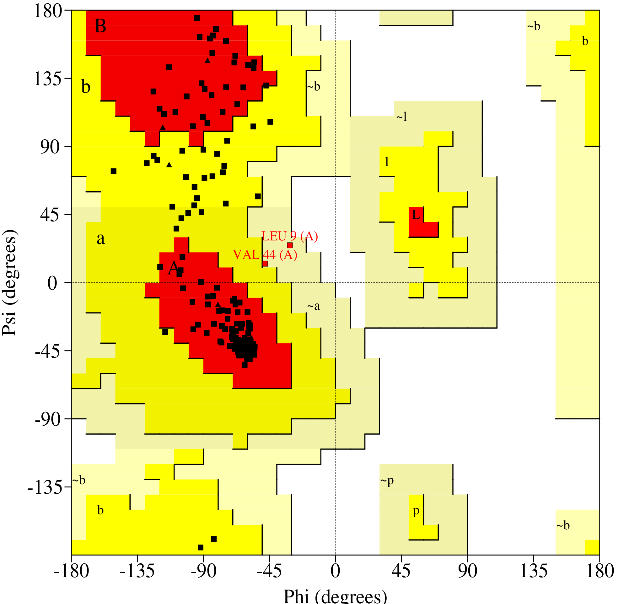


**Ramachandran plot for the model structure of Q9KSV6**

| Residues in most favoured region [A, B, L] | 157 | 84.4% |
| --- | --- | --- |
| Residues in additional allowed region [a, b, l, p] | 27 | 14.5% |
| Residues in generously allowed regions [~a, ~b, ~l, ~p] | 2 | 1.1% |
| Residues in disallowed regions | 0 | 0.0% |
| Total (Number of non-glycine and non-proline residues) | 186 | 100% |
| Number of end-residues (excl. Gly and Pro) | 2 |  |
| Number of Glycine | 6 |  |
| Number of Proline | 7 |  |
| Total number of residues | 201 |  |

**DO.**

**Ramachandran plot statistics for the model structure of Q9KSV6**

**DP.**

**pLDDT value= 95**

**N**

**C**

**Model of the protein (UniProt ID- Q9KND3) derived from AlphaFold-mmseq2**

**DQ.**

**DR.**

**pLDDT graph for the model (UniProt ID-Q9KND3) PAE graph for the model Q9KND3**

**DS.**

**Z-score= -6.96**

**Overall model quality of the model structure of Q9KND3 by ProSA**

**DT.**

**Ramachandran plot for the model structure of Q9KND3**

| Residues in most favoured region [A, B, L] | 157 | 84.4% |
| --- | --- | --- |
| Residues in additional allowed region [a, b, l, p] | 27 | 14.5% |
| Residues in generously allowed regions [~a, ~b, ~l, ~p] | 2 | 1.1% |
| Residues in disallowed regions | 0 | 0.0% |
| Total (Number of non-glycine and non-proline residues) | 186 | 100% |
| Number of end-residues (excl. Gly and Pro) | 2 |  |
| Number of Glycine | 6 |  |
| Number of Proline | 7 |  |
| Total number of residues | 201 |  |

**DU.**

**Ramachandran plot statistics for the model structure of Q9KND3**

**DV.**

**pLDDT value= 92.7**

**C**

**N**

**Model of the protein (UniProt ID- Q9KP29) derived from AlphaFold-mmseq2**

**DX.**

**DW.**

**pLDDT graph for the model (UniProt ID-Q9KP29) PAE graph for the model Q9KP29**

**DY.**

**Z-score= -4.21**

**Overall model quality of the model structure of Q9KP29 by ProSA**

**DZ.**

**Ramachandran plot for the model structure of Q9KP29**

| Residues in most favoured region [A, B, L] | 133 | 80.6% |
| --- | --- | --- |
| Residues in additional allowed region [a, b, l, p] | 27 | 16.4% |
| Residues in generously allowed regions [~a, ~b, ~l, ~p] | 4 | 2.4% |
| Residues in disallowed regions | 1 | 0.6% |
| Total (Number of non-glycine and non-proline residues) | 165 | 100% |
| Number of end-residues (excl. Gly and Pro) | 2 |  |
| Number of Glycine | 10 |  |
| Number of Proline | 6 |  |
| Total number of residues | 183 |  |

**EA.**

**Ramachandran plot statistics for the model structure of Q9KP29**

**EB.**

**C**

**pLDDT value= 86.8**

**N**

**Model of the protein (UniProt ID- Q9KMX1) derived from AlphaFold-mmseq2**

**EC.**

**ED.**

**pLDDT graph for the model (UniProt ID-Q9KMX1) PAE graph for the model Q9KMX1**

**EE.**

**Z-score= -2.89**

**Overall model quality of the model structure of Q9KMX1 by ProSA**

**EF.**

**Ramachandran plot for the model structure of Q9KMX1**

| Residues in most favoured region [A, B, L] | 127 | 83.0% |
| --- | --- | --- |
| Residues in additional allowed region [a, b, l, p] | 15 | 9.8% |
| Residues in generously allowed regions [~a, ~b, ~l, ~p] | 11 | 7.2% |
| Residues in disallowed regions | 0 | 0.0% |
| Total (Number of non-glycine and non-proline residues) | 153 | 100% |
| Number of end-residues (excl. Gly and Pro) | 2 |  |
| Number of Glycine | 20 |  |
| Number of Proline | 7 |  |
| Total number of residues | 182 |  |

**EG.**

**Ramachandran plot for the model structure of Q9KMX1**

**EH.**

**C**

**N**

**pLDDT value= 85.8**

**Model of the protein (UniProt ID- Q9KTE5) derived from AlphaFold-mmseq2**

**EI.**

**EJ.**

**pLDDT graph for the model (UniProt ID-Q9KTE5) PAE graph for the model Q9KTE5**

**EK.**

**Z-score= -6.2**

**Overall model quality of the model structure of Q9KTE5 by ProSA**

**EL.**

**Ramachandran plot for the model structure of Q9KTE5**

| Residues in most favoured region [A, B, L] | 132 | 95.0% |
| --- | --- | --- |
| Residues in additional allowed region [a, b, l, p] | 6 | 4.3% |
| Residues in generously allowed regions [~a, ~b, ~l, ~p] | 1 | 0.7% |
| Residues in disallowed regions | 0 | 0.0% |
| Total (Number of non-glycine and non-proline residues) | 139 | 100% |
| Number of end-residues (excl. Gly and Pro) | 1 |  |
| Number of Glycine | 9 |  |
| Number of Proline | 7 |  |
| Total number of residues | 156 |  |

**EM.**

**Ramachandran plot statistics for the model structure of Q9KTE5**

**EN.**

**C**

**pLDDT value= 84.3**

**N**

**Model of the protein (UniProt ID- Q9KPD6) derived from AlphaFold-mmseq2**

**EO.**

**EP.**

**pLDDT graph for the model (UniProt ID-Q9KPD6) PAE graph for the model Q9KPD6**

**EQ.**

**Z-score= -4.84**

**Overall model quality of the model structure of Q9KPD6 by ProSA**

**ER.**

**Ramachandran plot for the model structure of Q9KPD6**

| Residues in most favoured region [A, B, L] | 115 | 86.5% |
| --- | --- | --- |
| Residues in additional allowed region [a, b, l, p] | 17 | 12.8% |
| Residues in generously allowed regions [~a, ~b, ~l, ~p] | 0 | 0.0% |
| Residues in disallowed regions | 1 | 0.8% |
| Total (Number of non-glycine and non-proline residues) | 133 | 100.0% |
| Number of end-residues (excl. Gly and Pro) | 2 |  |
| Number of Glycine | 4 |  |
| Number of Proline | 9 |  |
| Total number of residues | 148 |  |

**ES.**

**Ramachandran plot statistics for the model structure of Q9KPD6**

**ET.**

**C**

**pLDDT value= 83.7**

**N**

**Model of the protein (UniProt ID- Q9KPA3) derived from AlphaFold-mmseq2**

**EV.**

**EU.**

**pLDDT graph for the model (UniProt ID-Q9KPA3) PAE graph for the model Q9KPA3**

**EW.**

**Z-score= -4.03**

**Overall model quality of the model structure of Q9KPA3 by ProSA**

**EX.**

**Ramachandran plot for the model structure of Q9KPA3**

| Residues in most favoured region [A, B, L] | 121 | 90.3% |
| --- | --- | --- |
| Residues in additional allowed region [a, b, l, p] | 8 | 6.0% |
| Residues in generously allowed regions [~a, ~b, ~l, ~p] | 5 | 3.7% |
| Residues in disallowed regions | 0 | 0.0% |
| Total (Number of non-glycine and non-proline residues) | 134 | 100.0% |
| Number of end-residues (excl. Gly and Pro) | 2 |  |
| Number of Glycine | 7 |  |
| Number of Proline | 5 |  |
| Total number of residues | 148 |  |

**EY.**

**Ramachandran plot statistics for the model structure of Q9KPA3**

**EZ.**

**pLDDT value= 86.9**

**C**

**N**

**Model of the protein (UniProt ID- Q9KNF4) derived from AlphaFold-mmseq2**

**FA.**

**FB.**

**pLDDT graph for the model (UniProt ID-Q9KNF4) PAE graph for the model Q9KNF4**

**FC.**

**Z-score= -4.74**

**Overall model quality of the model structure of Q9KNF4 by ProSA**

**FD.**

**Ramachandran plot for the model structure of Q9KNF4**

| Residues in most favoured region [A, B, L] | 110 | 83.3% |
| --- | --- | --- |
| Residues in additional allowed region [a, b, l, p] | 14 | 10.6% |
| Residues in generously allowed regions [~a, ~b, ~l, ~p] | 8 | 6.1% |
| Residues in disallowed regions | 0 | 0.0% |
| Total (Number of non-glycine and non-proline residues) | 132 | 100% |
| Number of end-residues (excl. Gly and Pro) | 2 |  |
| Number of Glycine | 6 |  |
| Number of Proline | 5 |  |
| Total number of residues | 145 |  |

**FE.**

**Ramachandran plot statistics for the model structure of Q9KNF4**

**FF.**

**C**

**pLDDT value= 89.5**

**N**

**Model of the protein (UniProt ID- Q9KT53) derived from AlphaFold-mmseq2**

**FG.**

**FH.**

**pLDDT graph for the model (UniProt ID-Q9KT53) PAE graph for the model Q9KT53**

**FI.**

**Z-score= -2,47**

**Overall model quality of the model structure of Q9KT53 by ProSA**

**FJ.**

**Ramachandran plot for the model structure of Q9KT53**

| Residues in most favoured region [A, B, L] | 105 | 89.7% |
| --- | --- | --- |
| Residues in additional allowed region [a, b, l, p] | 8 | 6.8% |
| Residues in generously allowed regions [~a, ~b, ~l, ~p] | 4 | 3.4% |
| Residues in disallowed regions | 0 | 0.0% |
| Total (Number of non-glycine and non-proline residues) | 117 | 100% |
| Number of end-residues (excl. Gly and Pro) | 2 |  |
| Number of Glycine | 5 |  |
| Number of Proline | 8 |  |
| Total number of residues | 132 |  |

**FK.**

**Ramachandran plot statistics for the model structure of Q9KT53**

**FL.**

**C**

**pLDDT value= 56.1**

**N**

**Model of the protein (UniProt ID- Q9KT56) derived from AlphaFold-mmseq2**

**FN.**

**FM.**

**pLDDT graph for the model (UniProt ID-Q9KT56) PAE graph for the model Q9KT56**

**FO.**

**Z-score= -1.38**

**Overall model quality of the model structure of Q9KT56 by ProSA**

**FP.**

**Ramachandran plot for the model structure of Q9KT56**

| Residues in most favoured region [A, B, L] | 54 | 47.4% |
| --- | --- | --- |
| Residues in additional allowed region [a, b, l, p] | 32 | 28.1% |
| Residues in generously allowed regions [~a, ~b, ~l, ~p] | 28 | 24.6% |
| Residues in disallowed regions | 0 | 0.0% |
| Total (Number of non-glycine and non-proline residues) | 114 | 100% |
| Number of end-residues (excl. Gly and Pro) | 2 |  |
| Number of Glycine | 6 |  |
| Number of Proline | 10 |  |
| Total number of residues | 132 |  |

**FQ.**

**Ramachandran plot statistics for the model structure of Q9KT56**

**FR.**

**C**

**pLDDT value= 81.2**

**N**

**Model of the protein (UniProt ID- Q9KRE6) derived from AlphaFold-mmseq2**

**FS.**

**FT.**

**pLDDT graph for the model (UniProt ID-Q9KRE6) PAE graph for the model Q9KRE6**

**FU.**

**Z-score= -3.2**

**Overall model quality of the model structure of Q9KRE6 by ProSA**

**FV.**

**Ramachandran plot for the model structure of Q9KRE6**

| Residues in most favoured region [A, B, L] | 89 | 77.4% |
| --- | --- | --- |
| Residues in additional allowed region [a, b, l, p] | 22 | 19.1% |
| Residues in generously allowed regions [~a, ~b, ~l, ~p] | 4 | 3.5% |
| Residues in disallowed regions | 0 | 0.0% |
| Total (Number of non-glycine and non-proline residues) | 115 | 100% |
| Number of end-residues (excl. Gly and Pro) | 2 |  |
| Number of Glycine | 5 |  |
| Number of Proline | 4 |  |
| Total number of residues | 126 |  |

**FW.**

**Ramachandran plot statistics for the model structure of Q9KRE6**

**FX.**

**pLDDT value= 95**

**C**

**N**

**Model of the protein (UniProt ID- Q9KLX2) derived from AlphaFold-mmseq2**

**FY.**

**FZ.**

**pLDDT graph for the model (UniProt ID-Q9KLX2) PAE graph for the model Q9KLX2**

**GA.**

**Z-score= -3.88**

**Overall model quality of the model structure of Q9KLX2 by ProSA**

**GB.**

**Ramachandran plot for the model structure of Q9KLX2**

| Residues in most favoured region [A, B, L] | 104 | 94.5% |
| --- | --- | --- |
| Residues in additional allowed region [a, b, l, p] | 6 | 5.5% |
| Residues in generously allowed regions [~a, ~b, ~l, ~p] | 0 | 0.0% |
| Residues in disallowed regions | 0 | 0.0% |
| Total (Number of non-glycine and non-proline residues) | 110 | 100% |
| Number of end-residues (excl. Gly and Pro) | 1 |  |
| Number of Glycine | 7 |  |
| Number of Proline | 4 |  |
| Total number of residues | 122 |  |

**GC.**

**Ramachandran plot statistics for the model structure of Q9KLX2**

**GD.**

**N**

**pLDDT value= 89.2**

**C**

**Model of the protein (UniProt ID- Q9KLQ3) derived from AlphaFold-mmseq2**

**GF.**

**GE.**

**pLDDT graph for the model (UniProt ID-Q9KLQ3) PAE graph for the model Q9KLQ3**

**GG.**

**Z-score= -2.95**

**Overall model quality of the model structure of Q9KLQ3 by ProSA**

**GH.**

**Ramachandran plot for the model structure of Q9KLQ3**

| Residues in most favoured region [A, B, L] | 106 | 97.2% |
| --- | --- | --- |
| Residues in additional allowed region [a, b, l, p] | 3 | 2.8% |
| Residues in generously allowed regions [~a, ~b, ~l, ~p] | 0 | 0.0% |
| Residues in disallowed regions | 0 | 0.0% |
| Total (Number of non-glycine and non-proline residues) | 109 | 100% |
| Number of end-residues (excl. Gly and Pro) | 2 |  |
| Number of Glycine | 2 |  |
| Number of Proline | 2 |  |
| Total number of residues | 115 |  |

**GI.**

**Ramachandran plot statistics for the model structure of Q9KLQ3**

**GJ.**

**C**

**pLDDT value= 72.5**

**N**

**Model of the protein (UniProt ID- Q9KKS6) derived from AlphaFold-mmseq2**

**GK.**

**GL.**

**pLDDT for the model (UniProt ID-Q9KKS6) PAE graph for the model Q9KKS6**

**GM.**

**Z-score= -3.74**

**Overall model quality of the model structure of Q9KKS6 by ProSA**

**GN.**

**Ramachandran plot for the model structure of Q9KKS6**

| Residues in most favoured region [A, B, L] | 64 | 63.4% |
| --- | --- | --- |
| Residues in additional allowed region [a, b, l, p] | 20 | 19.8% |
| Residues in generously allowed regions [~a, ~b, ~l, ~p] | 13 | 12.9% |
| Residues in disallowed regions | 4 | 4.0% |
| Total (Number of non-glycine and non-proline residues) | 101 | 100% |
| Number of end-residues (excl. Gly and Pro) | 2 |  |
| Number of Glycine | 8 |  |
| Number of Proline | 2 |  |
| Total number of residues | 113 |  |

**GO.**

**Ramachandran plot statistics for the model structure of Q9KKS6**

**GP.**

**N**

**pLDDT value= 90.8**

**C**

**Model of the protein (UniProt ID- Q9KN87) derived from AlphaFold-mmseq2**

**GQ.**

**GR.**

**pLDDT for the model (UniProt ID-Q9KN87) PAE graph for the model Q9KN87**

**GS.**

**Z-score= -5.03**

**Overall model quality of the model structure of Q9KN87 by ProSA**

**GT.**

**Ramachandran plot for the model structure of Q9KN87**

| Residues in most favoured region [A, B, L] | 82 | 82.0% |
| --- | --- | --- |
| Residues in additional allowed region [a, b, l, p] | 10 | 10.0% |
| Residues in generously allowed regions [~a, ~b, ~l, ~p] | 8 | 8.0% |
| Residues in disallowed regions | 0 | 0.0% |
| Total (Number of non-glycine and non-proline residues) | 100 | 100% |
| Number of end-residues (excl. Gly and Pro) | 2 |  |
| Number of Glycine | 3 |  |
| Number of Proline | 5 |  |
| Total number of residues | 110 |  |

**GU.**

**Ramachandran plot statistics for the model structure of Q9KN87**

**GV.**

**pLDDT value= 73.4**

**N**

**C**

**Model of the protein (UniProt ID- Q9KU58) derived from AlphaFold-mmseq2**

**GX.**

**GW.**

**pLDDT for the model (UniProt ID-Q9KU58) PAE graph for the model Q9KU58**

**GY.**

**Z-score= -2.66**

**Overall model quality of the model structure of Q9KU58 by ProSA**

**GZ.**

**Ramachandran plot for the model structure of Q9KU58**

| Residues in most favoured region [A, B, L] | 73 | 79.3% |
| --- | --- | --- |
| Residues in additional allowed region [a, b, l, p] | 9 | 9.8% |
| Residues in generously allowed regions [~a, ~b, ~l, ~p] | 10 | 10.9% |
| Residues in disallowed regions | 0 | 0.0% |
| Total (Number of non-glycine and non-proline residues) | 92 | 100% |
| Number of end-residues (excl. Gly and Pro) | 2 |  |
| Number of Glycine | 4 |  |
| Number of Proline | 6 |  |
| Total number of residues | 104 |  |

**HA.**

**Ramachandran plot statistics for the model structure of Q9KU58**

**C**

**HB.**

**pLDDT value= 87.9**

**N**

**Model of the protein (UniProt ID- Q9KPP0) derived from AlphaFold-mmseq2**

**HC.**

**HD.**

**pLDDT for the model (UniProt ID-Q9KPP0) PAE graph for the model Q9KPP0**

**HE.**

**Z-score= -3.98**

**Overall model quality of the model structure of Q9KPP0 by ProSA**

**HF.**

**Ramachandran plot for the model structure of Q9KPP0**

| Residues in most favoured region [A, B, L] | 73 | 78.5% |
| --- | --- | --- |
| Residues in additional allowed region [a, b, l, p] | 20 | 21.5% |
| Residues in generously allowed regions [~a, ~b, ~l, ~p] | 0 | 0.0% |
| Residues in disallowed regions | 0 | 0.0% |
| Total (Number of non-glycine and non-proline residues) | 93 | 100% |
| Number of end-residues (excl. Gly and Pro) | 2 |  |
| Number of Glycine | 4 |  |
| Number of Proline | 3 |  |
| Total number of residues | 102 |  |

**HG.**

**Ramachandran plot statistics for the model structure of Q9KU58**

**HH.**

**pLDDT value= 67.9**

**C**

**N**

**Model of the protein (UniProt ID- B1B1N2) derived from AlphaFold-mmseq2**

**HI.**

**HJ.**

**pLDDT for the model (UniProt ID-B1B1N2) PAE graph for the model B1B1N2**

**HK.**

**Z-score= -2.35**

**Overall model quality of the model structure of B1B1N2 by ProSA**

**HL.**

**Ramachandran plot for the model structure of B1B1N2**

| Residues in most favoured region [A, B, L] | 50 | 60.2% |
| --- | --- | --- |
| Residues in additional allowed region [a, b, l, p] | 17 | 20.5% |
| Residues in generously allowed regions [~a, ~b, ~l, ~p] | 13 | 15.7% |
| Residues in disallowed regions | 3 | 3.6% |
| Total (Number of non-glycine and non-proline residues) | 83 | 100% |
| Number of end-residues (excl. Gly and Pro) | 2 |  |
| Number of Glycine | 6 |  |
| Number of Proline | 2 |  |
| Total number of residues | 93 |  |

**HM.**

**Ramachandran plot statistics for the model structure of B1B1N2**

**HN.**

**C**

**pLDDT value= 88.2**

**N**

**Model of the protein (UniProt ID- Q9K2J6) derived from AlphaFold-mmseq2**

**HP.**

**HO.**

**pLDDT for the model (UniProt ID-Q9K2J6) PAE graph for the model Q9K2J6**

**HQ.**

**Z-score= -1.67**

**Overall model quality of the model structure of Q9K2J6 by ProSA**

**HR.**

**Ramachandran plot for the model structure of Q9K2J6**

| Residues in most favoured region [A, B, L] | 76 | 89.4% |
| --- | --- | --- |
| Residues in additional allowed region [a, b, l, p] | 8 | 9.4% |
| Residues in generously allowed regions [~a, ~b, ~l, ~p] | 1 | 1.2% |
| Residues in disallowed regions | 0 | 0.0% |
| Total (Number of non-glycine and non-proline residues) | 85 | 100% |
| Number of end-residues (excl. Gly and Pro) | 2 |  |
| Number of Glycine | 1 |  |
| Number of Proline | 2 |  |
| Total number of residues | 90 |  |

**HS.**

**Ramachandran plot statistics for the model structure of Q9K2J6**

**HT.**

**C**

**pLDDT value= 88.5**

**N**

**Model of the protein (UniProt ID- Q9KS64) derived from AlphaFold-mmseq2**

**HU.**

**HV.**

**pLDDT for the model (UniProt ID-Q9KS64) PAE graph for the model Q9KS64**

**HW.**

**Z-score= -6.64**

**Overall model quality of the model structure of Q9KS64 by ProSA**

**HX.**

**Ramachandran plot for the model structure of Q9KS64**

| Residues in most favoured region [A, B, L] | 61 | 81.3% |
| --- | --- | --- |
| Residues in additional allowed region [a, b, l, p] | 8 | 10.7% |
| Residues in generously allowed regions [~a, ~b, ~l, ~p] | 4 | 5.3% |
| Residues in disallowed regions | 2 | 2.7% |
| Total (Number of non-glycine and non-proline residues) | 75 | 100% |
| Number of end-residues (excl. Gly and Pro) | 2 |  |
| Number of Glycine | 4 |  |
| Number of Proline | 5 |  |
| Total number of residues | 86 |  |

**HY.**

**Ramachandran plot statistics for the model structure of Q9KS64**

**HZ.**

**C**

**pLDDT value= 84.2**

**N**

**Model of the protein (UniProt ID- Q9KN40) derived from AlphaFold-mmseq2**

**IA.**

**IB.**

**pLDDT for the model (UniProt ID-Q9KN40) PAE graph for the model Q9KN40**

**IC.**

**Z-score= -3.91**

**Overall model quality of the model structure of Q9KN40 by ProSA**

**ID.**

**Ramachandran plot for the model structure of Q9KN40**

| Residues in most favoured region [A, B, L] | 60 | 75.9% |
| --- | --- | --- |
| Residues in additional allowed region [a, b, l, p] | 6 | 7.6% |
| Residues in generously allowed regions [~a, ~b, ~l, ~p] | 13 | 16.5% |
| Residues in disallowed regions | 0 | 0.0% |
| Total (Number of non-glycine and non-proline residues) | 79 | 100% |
| Number of end-residues (excl. Gly and Pro) | 2 |  |
| Number of Glycine | 4 |  |
| Number of Proline | 1 |  |
| Total number of residues | 86 |  |

**IE.**

**Ramachandran plot statistics for the model structure of Q9KS40**

**IF.**

**N**

**pLDDT value= 79.4**

**C**

**Model of the protein (UniProt ID- Q9KVW5) derived from AlphaFold-mmseq2**

**IH.**

**IG.**

**pLDDT for the model (UniProt ID-Q9KVW5) PAE graph for the model Q9KVW5**

**II.**

**Z-score= -0.56**

**Overall model quality of the model structure of Q9KVW5 by ProSA**

**IJ.**

**Ramachandran plot for the model structure of Q9KVW5**

| Residues in most favoured region [A, B, L] | 60 | 75.9% |
| --- | --- | --- |
| Residues in additional allowed region [a, b, l, p] | 6 | 7.6% |
| Residues in generously allowed regions [~a, ~b, ~l, ~p] | 13 | 16.5% |
| Residues in disallowed regions | 0 | 0.0% |
| Total (Number of non-glycine and non-proline residues) | 79 | 100% |
| Number of end-residues (excl. Gly and Pro) | 2 |  |
| Number of Glycine | 4 |  |
| Number of Proline | 1 |  |
| Total number of residues | 86 |  |

**IK.**

**Ramachandran plot statistics for the model structure of Q9KVW5**

**IL.**

**N**

**pLDDT value= 47.3**

**C**

**Model of the protein (UniProt ID- Q9KKL81 derived from AlphaFold-mmseq2**

**IM.**

**IN.**

**pLDDT for the model (UniProt ID-Q9KL81) PAE graph for the model Q9KL81**

**IO.**

**Z-score= -1.17**

**Overall model quality of the model structure of Q9KL81 by ProSA**

**IP.**

**Ramachandran plot for the model structure of Q9KL81**

| Residues in most favoured region [A, B, L] | 66 | 91.7% |
| --- | --- | --- |
| Residues in additional allowed region [a, b, l, p] | 5 | 6.9% |
| Residues in generously allowed regions [~a, ~b, ~l, ~p] | 1 | 1.4% |
| Residues in disallowed regions | 0 | 0.0% |
| Total (Number of non-glycine and non-proline residues) | 72 | 100% |
| Number of end-residues (excl. Gly and Pro) | 2 |  |
| Number of Glycine | 0 |  |
| Number of Proline | 5 |  |
| Total number of residues | 79 |  |

**IQ.**

**Ramachandran plot statistics for the model structure of Q9KL81**

**IR.**

**C**

**N**

**pLDDT value= 88.1**

**Model of the protein (UniProt ID- Q9KPA0 derived from AlphaFold-mmseq2**

**IS.**

**IT.**

**pLDDT for the model (UniProt ID-Q9KPA0) PAE graph for the model Q9KPA0**

**IU.**

**Z-score= -4.23**

**Overall model quality of the model structure of Q9KPA0 by ProSA**

**IV.**

**Ramachandran plot for the model structure of Q9KPA0**

| Residues in most favoured region [A, B, L] | 60 | 89.6% |
| --- | --- | --- |
| Residues in additional allowed region [a, b, l, p] | 6 | 9.0% |
| Residues in generously allowed regions [~a, ~b, ~l, ~p] | 0 | 0.0% |
| Residues in disallowed regions | 1 | 1.5% |
| Total (Number of non-glycine and non-proline residues) | 67 | 100% |
| Number of end-residues (excl. Gly and Pro) | 2 |  |
| Number of Glycine | 5 |  |
| Number of Proline | 3 |  |
| Total number of residues | 77 |  |

**IW.**

**Ramachandran plot statistics for the model structure of Q9KPA0**

**IX.**

**pLDDT value= 84**

**C**

**N**

**Model of the protein (UniProt ID- Q9KL73 derived from AlphaFold-mmseq2**

**IZ.**

**IY.**

**pLDDT for the model (UniProt ID-Q9KL73) PAE graph for the model Q9KL73**

**JA.**

**Z-score= -3.83**

**Overall model quality of the model structure of Q9KL73 by ProSA**

**JB.**

**Overall model quality of the model structure of Q9KL73 by ProSA**

**JC.**

| Residues in most favoured region [A, B, L] | 50 | 82.0% |
| --- | --- | --- |
| Residues in additional allowed region [a, b, l, p] | 4 | 6.6% |
| Residues in generously allowed regions [~a, ~b, ~l, ~p] | 6 | 9.8% |
| Residues in disallowed regions | 1 | 1.6% |
| Total (Number of non-glycine and non-proline residues) | 61 | 100% |
| Number of end-residues (excl. Gly and Pro) | 2 |  |
| Number of Glycine | 3 |  |
| Number of Proline | 1 |  |
| Total number of residues | 67 |  |

**Ramachandran plot statistics for the model structure of Q9KL73**

**JD.**

**N**

**pLDDT value= 69.7**

**C**

**Model of the protein (UniProt ID- Q9KNG0 derived from AlphaFold-mmseq2**

**JE.**

**JF.**

**pLDDT for the model (UniProt ID-Q9KNG0) PAE graph for the model Q9KNG0**

**JG.**

**Z-score= -3.75**

**Overall model quality of the model structure of Q9KNG0 by ProSA**

**JH.**

**Overall model quality of the model structure of Q9KNG0 by ProSA**

| Residues in most favoured region [A, B, L] | 52 | 86.7% |
| --- | --- | --- |
| Residues in additional allowed region [a, b, l, p] | 5 | 8.3% |
| Residues in generously allowed regions [~a, ~b, ~l, ~p] | 2 | 3.3% |
| Residues in disallowed regions | 1 | 1.7% |
| Total (Number of non-glycine and non-proline residues) | 60 | 100% |
| Number of end-residues (excl. Gly and Pro) | 2 |  |
| Number of Glycine | 2 |  |
| Number of Proline | 1 |  |
| Total number of residues | 65 |  |

**JI.**

**Ramachandran plot statistics for the model structure of Q9KNG0**

**JJ.**

**pLDDT value= 79**

**C**

**N**

**Model of the protein (UniProt ID- Q9KSJ4 derived from AlphaFold-mmseq2**

**JK.**

**JL.**

**pLDDT for the model (UniProt ID-Q9KSJ4) PAE graph for the model Q9KSJ4**

**JM.**

**Z-score= -3.38**

**Overall model quality of the model structure of Q9KSJ4 by ProSA**

**JM.**

**Overall model quality of the model structure of Q9KSJ4 by ProSA**

| Residues in most favoured region [A, B, L] | 48 | 88.9% |
| --- | --- | --- |
| Residues in additional allowed region [a, b, l, p] | 6 | 11.1% |
| Residues in generously allowed regions [~a, ~b, ~l, ~p] | 0 | 0.0% |
| Residues in disallowed regions | 0 | 0.0% |
| Total (Number of non-glycine and non-proline residues) | 54 | 100% |
| Number of end-residues (excl. Gly and Pro) | 2 |  |
| Number of Glycine | 0 |  |
| Number of Proline | 2 |  |
| Total number of residues | 58 |  |

**JN.**

**Ramachandran plot statistics for the model structure of Q9KSJ4**

**JO.**

**C**

**pLDDT value= 74.7**

**N**

**Model of the protein (UniProt ID- Q9KPZ1 derived from AlphaFold-mmseq2**

**JQ.**

**JP.**

**pLDDT for the model (UniProt ID-Q9KPZ1) PAE graph for the model Q9KPZ1**

**JR.**

**Z-score= 0.25**

**Overall model quality of the model structure of Q9KPZ1 by ProSA**

**JS.**

**Overall model quality of the model structure of Q9KPZ1 by ProSA**

| Residues in most favoured region [A, B, L] | 36 | 97.3% |
| --- | --- | --- |
| Residues in additional allowed region [a, b, l, p] | 1 | 2.7% |
| Residues in generously allowed regions [~a, ~b, ~l, ~p] | 0 | 0.0% |
| Residues in disallowed regions | 0 | 0.0% |
| Total (Number of non-glycine and non-proline residues) | 37 | 100% |
| Number of end-residues (excl. Gly and Pro) | 2 |  |
| Number of Glycine | 6 |  |
| Number of Proline | 1 |  |
| Total number of residues | 46 |  |

**JT.**

**Ramachandran plot statistics for the model structure of Q9KPZ1**

**JU.**

**pLDDT value= 72.4**

**C**

**N**

**Model of the protein (UniProt ID- Q9KNI6 derived from AlphaFold-mmseq2**

**JV.**

**JW.**

**pLDDT for the model (UniProt ID-Q9KNI6) PAE graph for the model Q9KNI6**

**JX.**

**Z-score= -1.74**

**Overall model quality of the model structure of Q9KNI6 by ProSA**

**JY.**

**Overall model quality of the model structure of Q9KNI6 by ProSA**

| Residues in most favoured region [A, B, L] | 41 | 100% |
| --- | --- | --- |
| Residues in additional allowed region [a, b, l, p] | 0 | 0.0% |
| Residues in generously allowed regions [~a, ~b, ~l, ~p] | 0 | 0.0% |
| Residues in disallowed regions | 0 | 0.0% |
| Total (Number of non-glycine and non-proline residues) | 41 | 100% |
| Number of end-residues (excl. Gly and Pro) | 2 |  |
| Number of Glycine | 3 |  |
| Number of Proline | 0 |  |
| Total number of residues | 46 |  |

**JZ**

**Ramachandran plot statistics for the model structure of Q9KNI6**

**KA.**

**C**

**pLDDT value= 70**

**N**

**Model of the protein (UniProt ID- Q9KVT0 derived from AlphaFold-mmseq2**

**KB.**

**KC.**

**pLDDT for the model (UniProt ID-Q9KVT0) PAE graph for the model Q9KVT0**

**KD.**

**Z-score= -1.6**

**Overall model quality of the model structure of Q9KVT0 by ProSA**

**KE.**

**Overall model quality of the model structure of Q9KVT0 by ProSA**

| Residues in most favoured region [A, B, L] | 42 | 100% |
| --- | --- | --- |
| Residues in additional allowed region [a, b, l, p] | 0. | 0.0% |
| Residues in generously allowed regions [~a, ~b, ~l, ~p] | 0 | 0.0% |
| Residues in disallowed regions | 0 | 0.0% |
| Total (Number of non-glycine and non-proline residues) | 42 | 100% |
| Number of end-residues (excl. Gly and Pro) | 2 |  |
| Number of Glycine | 2 |  |
| Number of Proline | 0 |  |
| Total number of residues | 46 |  |

**KF.**

**Ramachandran plot statistics for the model structure of Q9KVT0**

**KF.**

**pLDDT value= 67.4**

**C**

**N**

**Model of the protein (UniProt ID- Q9KST0 derived from AlphaFold-mmseq2**

**KH.**

**KG.**

**pLDDT for the model (UniProt ID-Q9KST0) PAE graph for the model Q9KST0**

**KI.**

**Z-score= -0.36**

**Overall model quality of the model structure of Q9KST0 by ProSA**

**KJ.**

**Overall model quality of the model structure of Q9KST0 by ProSA**

| Residues in most favoured region [A, B, L] | 33 | 84.6% |
| --- | --- | --- |
| Residues in additional allowed region [a, b, l, p] | 2 | 5.1% |
| Residues in generously allowed regions [~a, ~b, ~l, ~p] | 2 | 5.1% |
| Residues in disallowed regions | 2 | 5.1% |
| Total (Number of non-glycine and non-proline residues) | 39 | 100% |
| Number of end-residues (excl. Gly and Pro) | 2 |  |
| Number of Glycine | 1 |  |
| Number of Proline | 3 |  |
| Total number of residues | 45 |  |

**KK.**

**Ramachandran plot statistics for the model structure of Q9KST0**
