# Supplementary material for: Comprehensive in silico analyses of fifty-one uncharacterized proteins from Vibrio cholerae
Source: PLoS One. 2024 Oct 4;19(10):e0311301. doi: 10.1371/journal.pone.0311301 (PMC11452002; doi:10.1371/journal.pone.0311301)
Supplement: S7 Fig — (DOCX) [file pone.0311301.s022.docx]

**Figure S7**

**Graphical representation of discontinuous B-cell epitope present within 24 uncharacterized proteins**

***
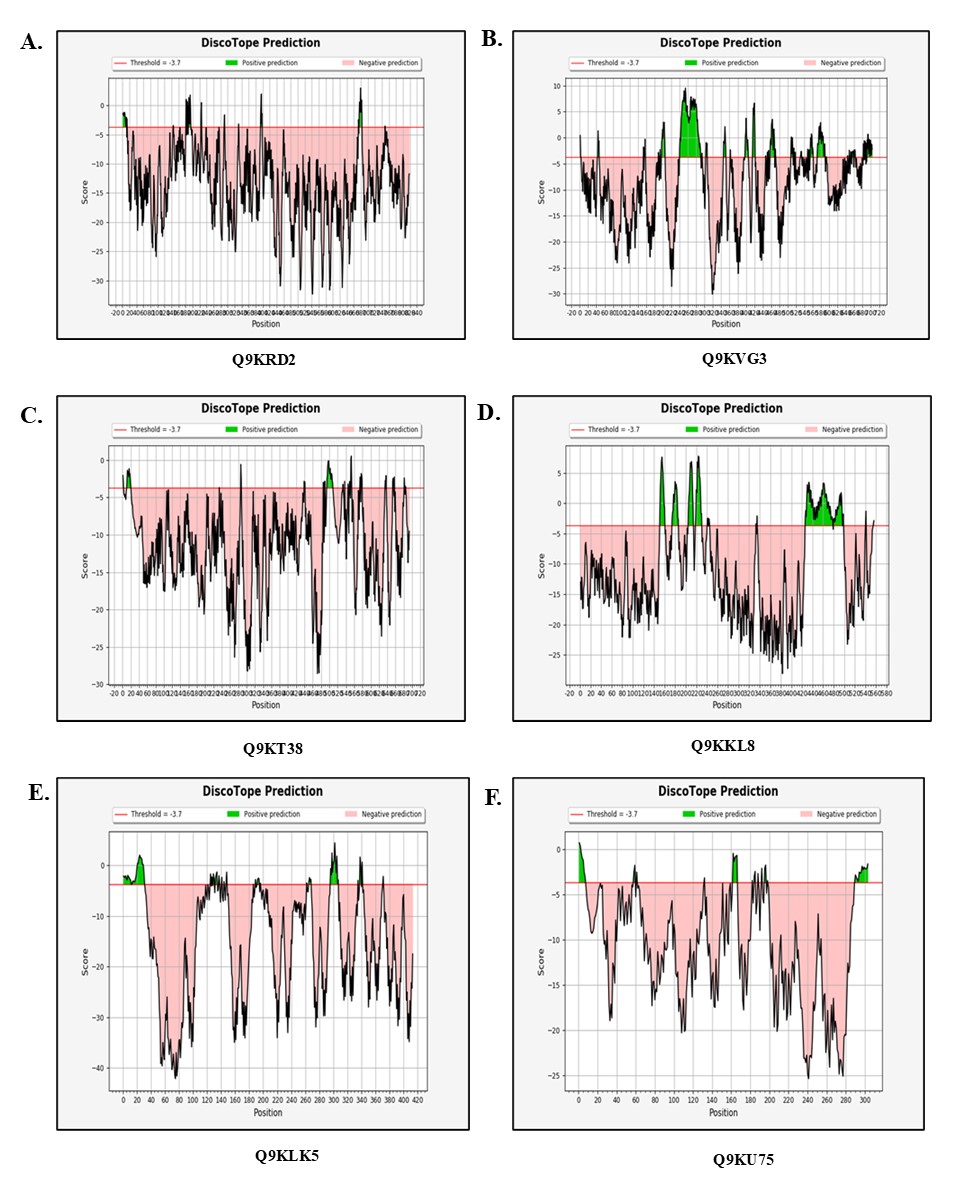
***

***
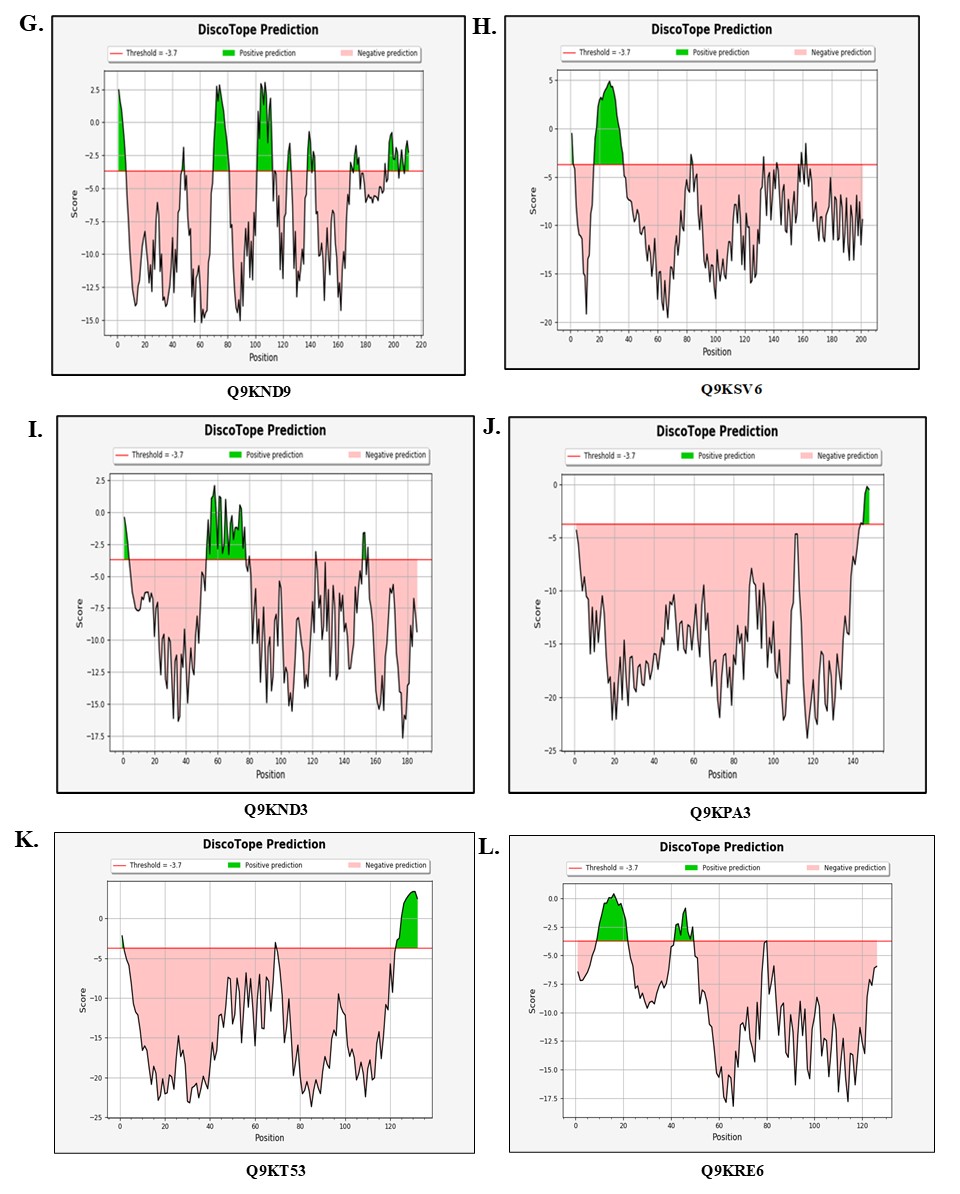
***

***
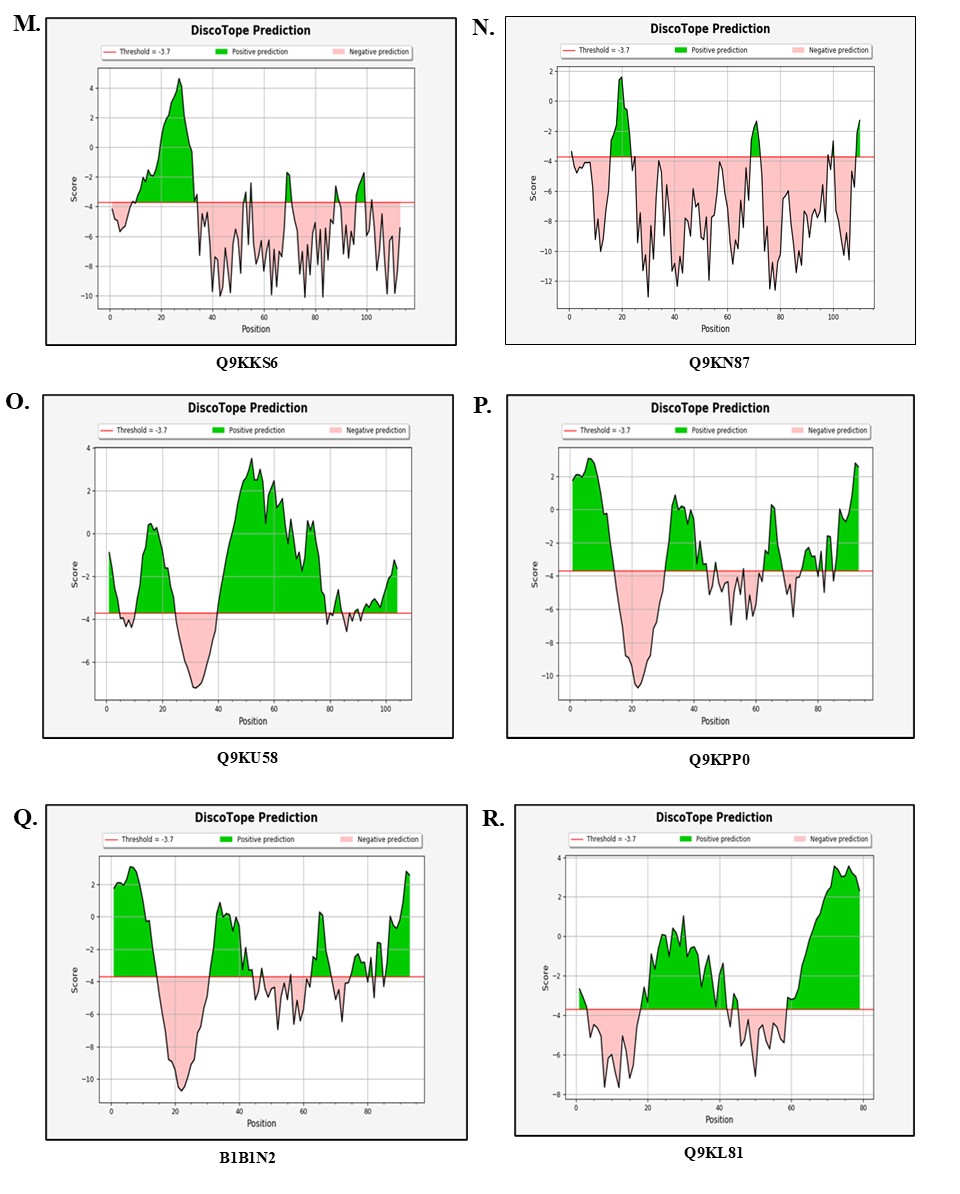
***

***
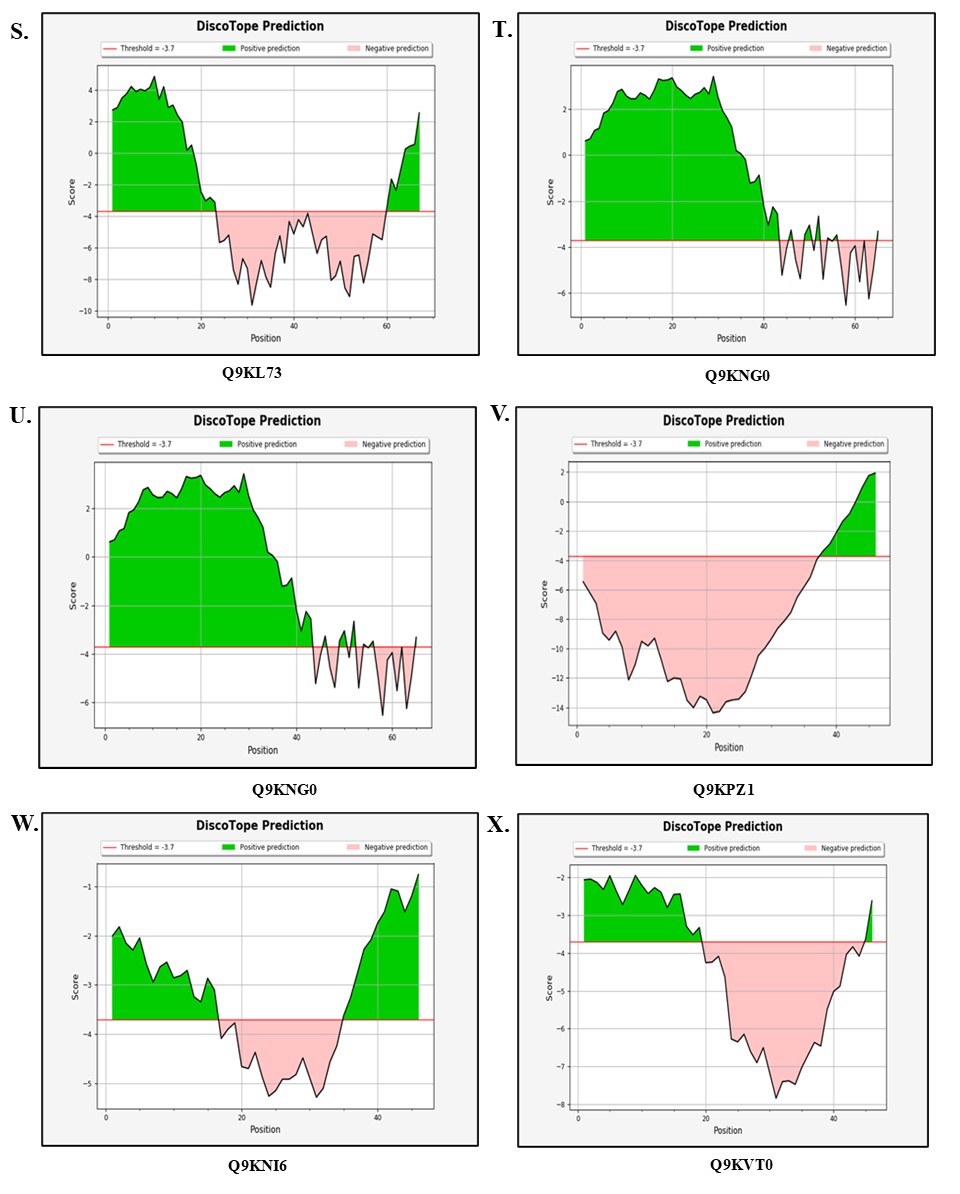
***
